# Programmable dynamic covalent nanoparticle building blocks with complementary reactivity†
†Electronic supplementary information (ESI) available: Synthetic procedures and characterization data for all compounds and nanoparticles (*in situ* and *ex situ* NMR, TEM, TGA, LDI-MS); stability tests on AuNP-**6**; kinetic analysis experimental methods and data for all experimental replicates; nanoparticle assembly experimental methods and monitoring by UV-Vis absorption spectroscopy, dynamic light scattering and TEM; assembly control experiments. The research data underpinning this publication can be accessed at DOI: 10.17630/184e09e0-2192-44f0-936f-6acbabc3ab7b. See DOI: 10.1039/c9sc04195h


**DOI:** 10.1039/c9sc04195h

**Published:** 2019-11-14

**Authors:** Nicolas Marro, Flavio della Sala, Euan R. Kay

**Affiliations:** a EaStCHEM School of Chemistry , University of St Andrews , North Haugh , St Andrews , KY16 9ST , UK . Email: ek28@st-andrews.ac.uk

## Abstract

Nanoparticle-based devices, materials and technologies will demand a new era of synthetic chemistry where predictive principles familiar in the molecular regime are extended to nanoscale building blocks. Typical covalent strategies for modifying nanoparticle-bound species rely on kinetically controlled reactions optimised for efficiency but with limited capacity for selective and divergent access to a range of product constitutions. In this work, monolayer-stabilized nanoparticles displaying complementary dynamic covalent hydrazone exchange reactivity undergo distinct chemospecific transformations by selecting appropriate combinations of ‘nucleophilic’ or ‘electrophilic’ nanoparticle-bound monolayers with nucleophilic or electrophilic molecular modifiers. Thermodynamically governed reactions allow modulation of product compositions, spanning mixed-ligand monolayers to exhaustive exchange. High-density nanoparticle-stabilizing monolayers facilitate *in situ* reaction monitoring by quantitative ^19^F NMR spectroscopy. Kinetic analysis reveals that hydrazone exchange rates are moderately diminished by surface confinement, and that the magnitude of this effect is dependent on mechanistic details: surface-bound electrophiles react intrinsically faster, but are more significantly affected by surface immobilization than nucleophiles. Complementary nanoparticles react with each other to form robust covalently connected binary aggregates. Endowed with the adaptive characteristics of the dynamic covalent linking process, the nanoscale assemblies can be tuned from extended aggregates to colloidally stable clusters of equilibrium sizes that depend on the concentration of a monofunctional capping agent. Just two ‘dynamic covalent nanoparticles’ with complementary thermodynamically governed reactivities therefore institute a programmable toolkit offering flexible control over nanoparticle surface functionalization, and construction of adaptive assemblies that selectively combine several nanoscale building blocks.

## Abstract 2

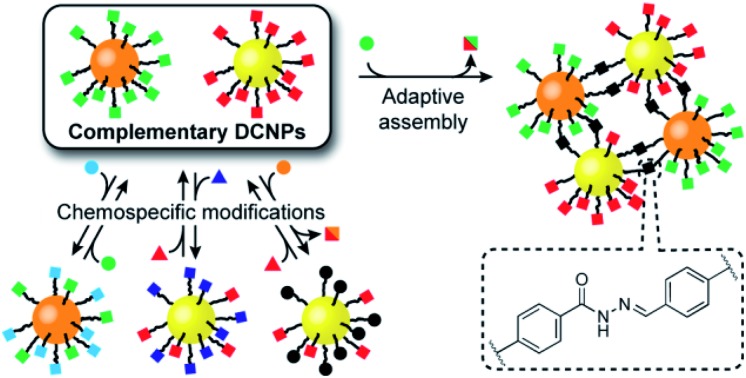
A toolkit of two complementary dynamic covalent nanoparticles enables programmable and reversible nanoparticle functionalization and construction of adaptive binary assemblies.

## Introduction

Generalizable principles of chemical reactivity are the bedrock of molecular and macromolecular synthetic chemistry methods. Similarly predictable strategies for modifying surface-bound species will be critical to exploiting the full technological potential of nanomaterials.^1^ Ideally, a relatively small set of nanoparticle ‘synthons’ would be amenable to efficient derivatization *via* specific and operationally simple synthetic methods that are independent of the underlying core. These would enable divergent post-synthesis routes for nanoparticle functionalization, property tuning, assembly, and integration with a range of other components.

Oligonucleotide-stabilized colloidal nanoparticles continue to set the benchmark,^2^ but are inherently restricted by relatively limited structural diversity and by the environmental conditions required to maintain structural and functional integrity of the surface-bound biomolecules. Numerous reports have explored covalent ligation to either biological or abiotic nanoparticle-bound molecules,1a,1c,1g and significant innovations continue to bring the prospect of generalizable nanoparticle building block strategies ever closer.^3^ Yet these tend to be optimized on a case-by-case basis and it is not yet clear how, or even whether, the predictive concepts of molecular synthetic chemistry can be extended to nanoscale entities. In contrast to the decades-long accumulation of knowledge from physical-organic studies on solution-phase molecular substrates, we lack a comparable systematic knowhow regarding chemical reactivity at nanoscale surfaces. Matching this level of structure–reactivity understanding will require nanoparticle platforms with features we take for granted in modern molecular synthetic methods, including chemospecific complementary and orthogonal reactivities, allied with real-time *in situ* characterization of nanoparticle-bound molecular structure. The heterogeneous, polydisperse and multi-component nature of nanoparticle systems presents a considerable barrier to achieving these ideals.

**Fig. 1:**
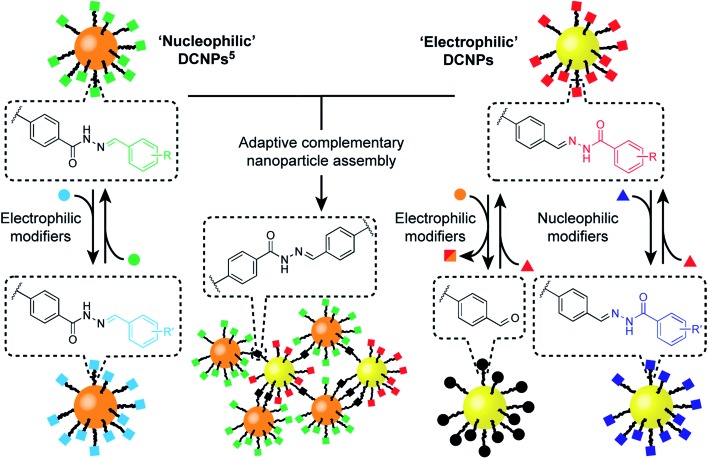
Functionalization and assembly of a pair of complementary hydrazone-based dynamic covalent nanoparticles (DCNPs) *via* chemospecific reversible covalent modifications. Starting from a toolkit of DCNPs bearing ‘nucleophilic’ (orange cores) and ‘electrophilic’ (yellow cores) hydrazones (squares), distinct transformations are triggered by combination with either electrophilic (circles) or nucleophilic (triangles) molecular modifiers, providing direct access to modified nanoparticle-bound hydrazone structures, or unconjugated aldehydes. In combination, complementary DCNPs react with each other to produce binary nanoparticle aggregates that are structurally adaptive to assembly reaction conditions.

We have recently implemented dynamic covalent reactions in nanoparticle-bound settings^4^ as a flexible means to reversibly alter the constitution of nanoparticle-stabilizing molecular monolayers,^5^ to tune the composition of mixed-ligand monolayers – and consequently nanoparticle properties^6^ – and to direct covalent assembly–disassembly of nanoparticle aggregates.^7^ Meanwhile, others have exploited reversible covalent reactions to achieve nanoparticle-bound monolayer constitutions that adapt to noncovalently bound templates,^8^ or to stabilize nanoparticle assemblies and surface attachment.^9^ Non-symmetrical dynamic covalent linkages (*e.g.* hydrazones, imines or boronate esters) are ideal for chemoselective functionalization strategies. However, attaching only one end of each dynamic covalent bond to the nanoparticle surface (in the case of our hydrazone-functionalized monolayers,^5^ the nucleophilic hydrazide portion, Fig. 1, ‘nucleophilic DCNPs’) restricts the range of accessible modifications. Generalizable synthetic strategies require a suite of building blocks with chemospecific reactivities that allow for complementary or orthogonal combinations.

Here we report the development of an ‘electrophilic’ nanoparticle partner for our first-generation ‘nucleophilic’ hydrazone-functionalized nanoparticles (Fig. 1). Distinct chemospecific monolayer modifications can be achieved by selecting either nucleophilic or electrophilic exchange units, and product compositions can be varied by simple stoichiometric tuning of the thermodynamically governed transformations, altogether amounting to comprehensive control over nanoparticle-bound monolayer constitution. We show that this platform allows structure–reactivity relationships for interfacial processes to be established, revealing mechanistic insights regarding reactivity in the nanoparticle-bound environment. Furthermore, this complementary pair of metallic nanoparticle building blocks opens the door to constructing covalently linked nanoparticle assemblies composed of two different nanoscale constituents. The combination of synthetic programmability and predictive understanding of molecular reactivity, allied with the flexibility and versatility of dynamic covalent reactivity using abiotic structures, suggests a general strategy for functionalizing and modifying nanoparticles with molecular precision, and for the programmable assembly of nano-sized objects to create new nanomaterials.

## Design, synthesis and structural characterization of ‘electrophilic’ hydrazone dynamic covalent nanoparticles

**Scheme 1:**
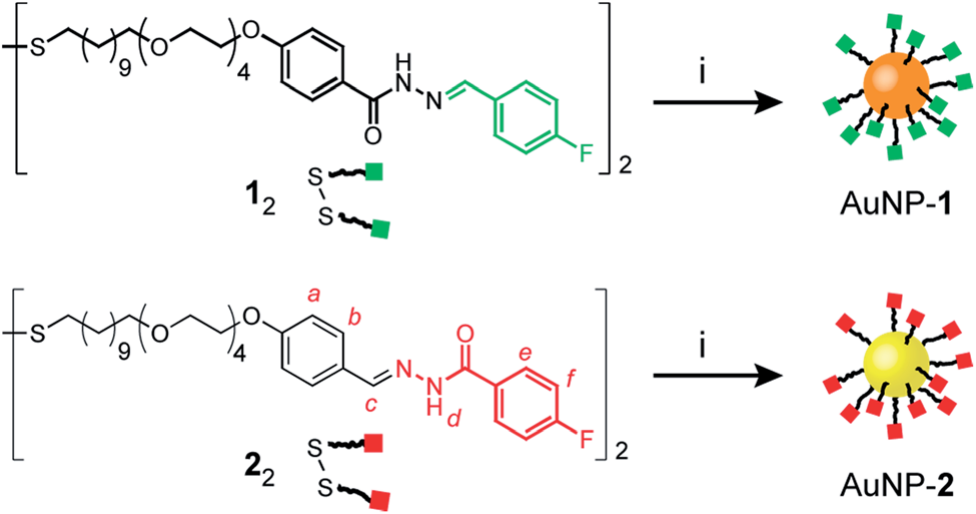
Preparation of complementary hydrazone-terminated dynamic covalent nanoparticles AuNP-**1** (orange core) and AuNP-**2** (yellow core). Green squares represent ‘nucleophilic’ and red squares ‘electrophilic’ nanoparticle-bound hydrazones. Reagents and conditions: (i) PPh_3_AuCl, *t*-BuNH_2_·BH_3_, THF/DMF (8 : 1 v/v), 50 °C to r.t., 6 h.

Mirroring our approach previously adopted for ‘nucleophilic’ hydrazone-functionalized gold nanoparticles (*e.g.* AuNP-**1**, Scheme 1, orange core),^5^ we sought to prepare nanoparticles stabilized by a single-component monolayer bearing electrophilic carbonyl functionality at the periphery. The incorporation of redox-sensitive carbonyl functionality at the surface of metal nanoparticles is not straightforward, however. The reducing conditions commonly used to prepare metallic nanoparticles are generally incompatible with aldehydes or ketones, while the efficiency of noble metal nanoparticles as redox catalysts can result in aldehyde oxidation.^10^ Previous reports of carbonyl-functionalized gold nanoparticles have exploited ligand-exchange protocols to incorporate surface-bound aldehydes or ketones only as minor monolayer components alongside non-reactive stabilizing ligands,^8^,9a,d,^11^ or using polymeric surface stabilizers.9d,^12^ To maximise the density of modifiable reactive sites, we chose to introduce aldehydes in the form of hydrazone-protected ligands as the only nanoparticle-bound species (Scheme 1, red squares). We previously demonstrated that hydrazone-stabilized AuNP-**1** could be prepared in a single synthetic step while avoiding hydrazone reduction.^5^ A similar protocol using disulfide ligand precursor **2**_2_ provided AuNP-**2** (Scheme 1, yellow core), where now the electrophilic carbonyl-derived end of the hydrazone bond is tethered to the nanoparticle surface *via* an alkyl-tetra(ethylene glycol) linker and strong gold–thiyl bond.

**Fig. 2:**
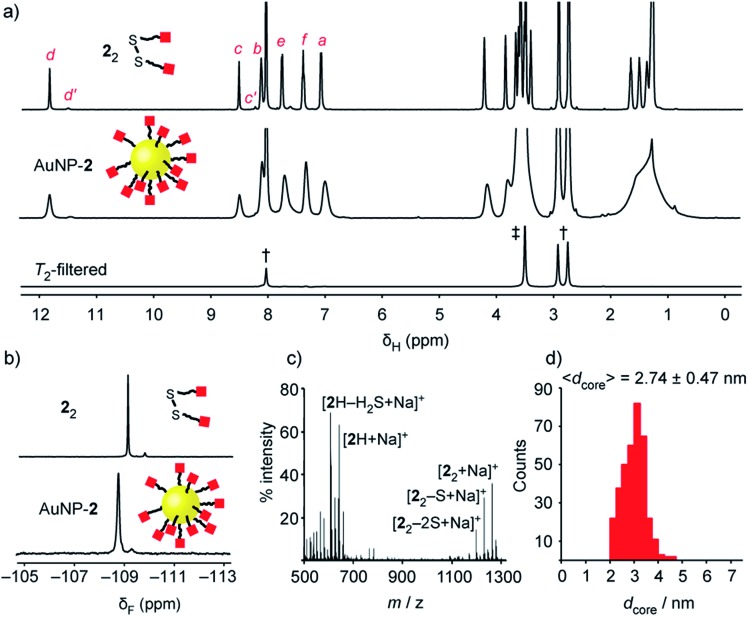
Characterization of ‘electrophilic’ DCNP AuNP-**2**. (a) ^1^H NMR spectra ([D_7_]DMF, 500 MHz, 295 K): **2**_2_ (top), AuNP-**2** (middle), *T*_2_-filtered spectrum recorded on AuNP-**2** using the CPMG-z pulse sequence^13^ (bottom). All sharp signals can be assigned to residual non-deuterated solvents († = DMF: *δ* 2.75 (s), 2.92 (s), 8.02 (s) ppm; ‡ = H_2_O: *δ* 3.50 (s) ppm). (b) Partial ^19^F NMR spectra ([D_7_]DMF, 470 MHz, 295 K, internal standard: CFCl_3_): **2**_2_ (top), AuNP-**2** (bottom). (c) LDI-MS of AuNP-**2**. (d) Size distribution of a representative batch of AuNP-**2** ( (〈*d*_core_〉 = 2.74 ± 0.47 nm). Full sweep-width NMR spectra, TEM images and size distributions of several nanoparticle batches can be found in the ESI. = 2.74 ± 0.47 nm). Full sweep-width NMR spectra, TEM images and size distributions of several nanoparticle batches can be found in the ESI.†

Nanoparticles were isolated from the reaction mixture by precipitation with a non-solvent. Several rounds of washing (re-dispersion, sonication, centrifugation and careful decanting of the supernatant) removed all non-surface-bound molecular species, and the purified sample freeze dried to provide analytically pure AuNP-**2**. Transmission electron microscopy (TEM) of several batches revealed that this procedure reproducibly yields spherical nanoparticles of mean diameter (. Transmission electron microscopy (TEM) of several batches revealed that this procedure reproducibly yields spherical nanoparticles of mean diameter (〈*d*_core_〉) in the range 2.7–3.0 nm, with relatively narrow monomodal size distributions (dispersities ≤ 17%, ) in the range 2.7–3.0 nm, with relatively narrow monomodal size distributions (dispersities ≤ 17%, Fig. 2d and S7†).

Colloidally stable solutions of AuNP-**2** could be prepared in DMF at high concentrations, on the order of 5 mM in terms of nanoparticle-bound ligands,^14^ allowing the structural and compositional integrity of the single-component monolayer to be verified by nuclear magnetic resonance (NMR) spectroscopy. The ^1^H NMR spectrum of AuNP-**2** shows characteristically broad resonances, which nonetheless can be matched with the resonances for molecular precursor **2**_2_ (Fig. 2a). No additional signals can be observed that would indicate degradation of hydrazone **2**, or incorporation of other species in the surface-bound monolayer. Likewise, the ^19^F NMR spectrum of AuNP-**2** revealed only resonances for the hydrazone ligand **2** (Fig. 2b).^15^ Spectral simplicity, excellent chemical shift dispersity and ease of quantification make ^19^F NMR a valuable tool for establishing molecular structure and *in situ* reaction tracking for nanoparticle-bound species (*vide infra*). Taking advantage of short relaxation times for nanoparticle-bound molecules, the CPMG-z pulse sequence provides a *T*_2_-filtered ^1^H spectrum with suppression of all signals for molecular-sized species.^13^ Observing only signals for residual non-deuterated solvents thus confirms the complete removal of all non-surface-bound impurities (Fig. 2a and S10†). The monolayer composition was further probed by oxidative release of surface-bound species using iodine. Analysis of the resulting supernatant by ^19^F NMR in the presence of an internal standard revealed only signals corresponding to the products of surface-released hydrazone **2** (Fig. S11†). This experiment also verified excellent quantitative agreement between the concentration of surface-bound hydrazones measured from the broad nanoparticle-bound spectrum and measured from the sharp bulk solution signals following surface release (Table S5†). Further confirmation of the monolayer constitution was provided by laser desorption ionization mass spectrometry (LDI-MS), which revealed mass ions for **2**H, and disulfide **2**_2_, along with their characteristic fragmentation patterns (Fig. 2c and S13†).

## Chemospecific dynamic covalent modifications of ‘electrophilic’ nanoparticle-bound hydrazone monolayers

**Fig. 3:**
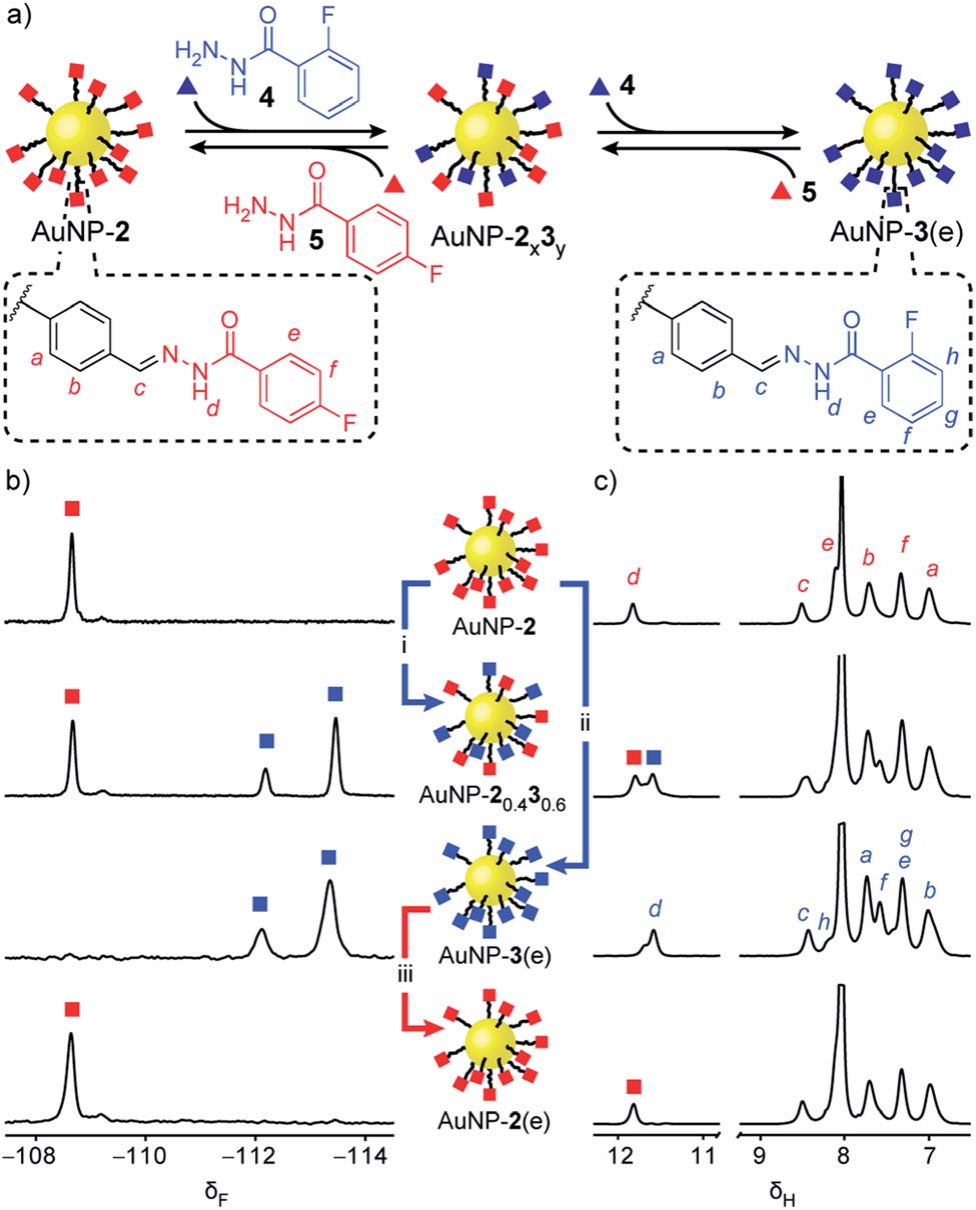
Reversible dynamic covalent modification of ‘electrophilic’ DCNPs (surface-bound hydrazones: squares) using nucleophilic molecular modifiers (hydrazides: triangles). (a) Schematic representation of the interconversion between AuNP-**2**, AuNP-**3** and intermediate mixed-ligand monolayer compositions AuNP-**2**_x_**3**_y_. General conditions: AuNP-**2** (5 mM), CF_3_CO_2_H (20 mM), DMF/D_2_O (9 : 1 v/v), rt. (b) Partial ^19^F NMR spectra ([D_7_]DMF, 470 MHz, 295 K, internal standard: CFCl_3_). (c) Partial ^1^H NMR spectra ([D_7_]DMF, 500 MHz, 295 K). Spectra from top to bottom: AuNP-**2** (direct synthesis); AuNP-**2**_0.4_**3**_0.6_ (conditions (i): 1.0 eq. **4**, 1 h); AuNP-**3**(e) (conditions (ii): 25 eq. **4**, 1.2 h); AuNP-**2**(e) (conditions (iii): 25 eq. **5**, 2.2 h). Full sweep-width spectra and pre-purification spectra can be found in Fig. S15–S17 and S21.†

‘Electrophilic’ nanoparticle-bound hydrazones (*e.g.* AuNP-**2** and AuNP-**3**: Fig. 3, red and blue squares respectively) can be readily interconverted on treatment with an appropriate nucleophilic modifier (*e.g.* hydrazides: Fig. 3, triangles). A colloidally stable solution of AuNP-**2** in 10% v/v D_2_O/DMF (5 mM in terms of nanoparticle-bound **2**)^14^ was treated with 2-fluorobenzoyl hydrazide **4** (5 mM) and CF_3_CO_2_H (20 mM) at room temperature. Monitoring by ^19^F NMR spectroscopy revealed sharp signals indicating a steady release of 4-fluorobenzoyl hydrazide **5** from the nanoparticle-bound monolayer, and concomitant consumption of hydrazide **4**. Meanwhile, broad nanoparticle-bound signals confirmed the quantitative replacement of hydrazones **2** for **3** on the nanoparticle surface (Fig. S15†). No further changes were observed after 1 hour. At this point, area deconvolution of either the broad signals for nanoparticle-bound hydrazones or the sharp signals for hydrazides released in bulk solution both indicated a nanoparticle-bound mixed-ligand monolayer comprising 58% hydrazone **3** and 42% hydrazone **2** (AuNP-**2**_0.4_**3**_0.6_ Table S7†). Incubating AuNP-**2** with a higher concentration of hydrazide **4** (25 mM, 5 molar equivalents), again with CF_3_CO_2_H (20 mM) at room temperature, shifted the equilibrium endpoint as expected, giving a surface-bound monolayer composition comprising 88% hydrazone **3** with only 12% hydrazone **2** remaining (AuNP-**2**_0.1_**3**_0.9_ Fig. S16c†). Exhaustive exchange could be achieved directly from AuNP-**2** in the presence of 25 molar equivalents hydrazide **4** under otherwise identical conditions, to produce single-component monolayer-stabilized nanoparticles AuNP-**3**(e) after 1.2 h (Fig. S16e†).^16^,^17^

In each case, the product nanoparticles were isolated and purified by multiple cycles of precipitation and washing. Pleasingly, subsequent analysis revealed the surface-bound monolayer composition is unchanged by the purification procedure, demonstrating the kinetic stability of the exchangeable hydrazone links in the absence of an acid catalyst. Comparison of the *in situ*^19^F and ^1^H NMR spectra of the purified samples (Fig. 3b and c and S16–S17†) evidences the single-component composition of the exhaustively exchanged monolayer on AuNP-**3**(e) and shows that mixed-ligand monolayers produce spectra that are simple superpositions of the two single-component monolayer spectra. Release of surface-bound species by oxidative ligand desorption confirmed the same monolayer compositions and the absence of any unfunctionalized aldehydes (Table S7 and Fig. S19–S20†). The reversibility of the dynamic covalent exchange process meant that AuNP-**2** could be regenerated by treating AuNP-**3**(e) with hydrazide **5**. Both ^19^F NMR and ^1^H NMR analysis *in situ* (AuNP-**2**(e),^16^Fig. 3b and c and S21†) and following oxidative ligand desorption (Fig. S22†) indicated a homogeneous monolayer of hydrazone **2**.

Remarkable structural and functional integrity was observed following the dynamic covalent exchange process. TEM imaging indicated no significant differences in the size or shape distribution of AuNP-**3**(e) or AuNP-**2**(e) compared to the starting AuNP-**2** (Fig. S23†). Likewise, thermal analysis and quantitative ^19^F NMR measurements confirmed that there was no loss of dynamic covalent functionality either by ligand desorption or irreversible covalent modification following the back-and-forth exchange between AuNP-**2** and AuNP-**2**(e) *via* AuNP-**3**(e) (Table S11†).

To compare post-synthesis monolayer modification by dynamic covalent exchange to *de novo* nanoparticle synthesis, AuNP-**3** were also prepared directly from the corresponding disulfide pro-ligand **3**_2_. Using the same synthetic conditions and purification procedure as for AuNP-**2**, the nanoparticles produced in this case were significantly larger (AuNP-**3**, , 〈*d*_core_〉 = 3.4–3.8 nm for four independent batches, Fig. S14 = 3.4–3.8 nm for four independent batches, Fig. S14†), highlighting the unpredictable and sensitive relationship between ligand molecular structure and metal nanoparticle nucleation/growth processes.1h,^18^ Analysis by ^1^H and ^19^F NMR spectroscopy indicated an almost identical hydrazone-terminated surface-bound monolayer on AuNP-**3** and AuNP-**3**(e) (Fig. S17†), with one exception that the material produced by direct synthesis retained some *tert*-butylamine impurity from the reducing agent. This comparison illustrates the significant advantages of a divergent ‘building block’ approach to functionalized nanoparticles. *De novo* synthesis requires preparing each new alkyl thiol ligand (required in large excess and entailing a seven-step synthetic procedure in this case) and empirical re-optimization of nanoparticle preparation and purification protocols. Instead, post-synthesis modification allows a set of nanoparticles with precisely the same core features (*e.g.* shape and size distribution) but bearing different monolayer structures to be efficiently prepared using standardized synthetic procedures.

**Fig. 4:**
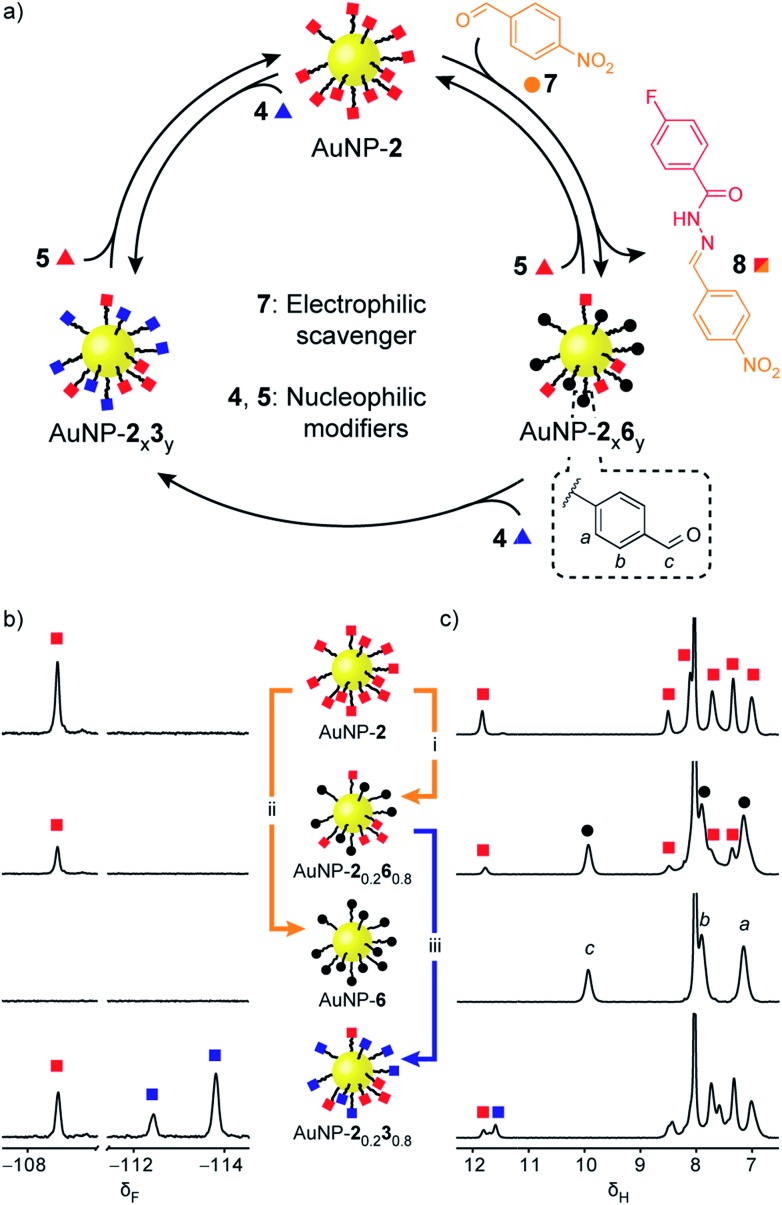
Reversible dynamic covalent modification of ‘electrophilic’ DCNPs (hydrazones: squares) using electrophilic molecular scavengers (aldehydes: circles) and nucleophilic modifiers (hydrazides: triangles). (a) Schematic representation of the interconversion between AuNP-**2**, AuNP-**6** and intermediate mixed-ligand monolayer compositions of hydrazones and aldehydes (AuNP-**2**_x_**6**_y_), or two different hydrazones (AuNP-**2**_x_**3**_y_). (b) Partial ^19^F NMR spectra ([D_7_]DMF, 470 MHz, 295 K, internal standard: CFCl_3_). (c) Partial ^1^H NMR spectra ([D_7_]DMF, 500 MHz, 295 K). Spectra from top to bottom: AuNP-**2** (direct synthesis); AuNP-**2**_0.2_**6**_0.8_ (conditions (i): 50 eq. **7**, 20 mM CF_3_CO_2_H, 45 °C, 6 h); AuNP-**6** (conditions (ii): 50 eq. **7**, 20 mM CF_3_CO_2_H, 45 °C, 26 h); AuNP-**2**_0.2_**3**_0.8_ prepared from AuNP-**2**_0.2_**6**_0.8_ (conditions (iii): 1 eq. **4** relative to **6**, 20 mM CF_3_CO_2_H, rt, 4.4 h). Full sweep-width spectra, pre-purification spectra, and post-purification characterization by oxidative ligand desorption can be found in Fig. S26–S29 and S31–S34.†

Rather than introducing nucleophilic molecular modifiers, reaction with an electrophilic exchange unit effects a chemically distinct modification to give nanoparticle-bound monolayers bearing unfunctionalized aldehydes **6** (Fig. 4, black circles). Treating AuNP-**2** with 4-nitrobenzaldehyde (**7**, Fig. 4, orange circles) trapped the hydrazide ‘protecting group’ as hydrazone **8**, producing AuNP-**2**_x_**6**_y_. Exchange with 50 molar equivalents **7** resulted in exhaustive hydrolysis to give AuNP-**6** after 26 h (Fig. 4b and c, S25–S27†). Likewise, mixed-ligand monolayers were readily accessible *via* two routes. Removing the acid catalyst kinetically trapped non-equilibrium monolayer compositions – for example AuNP-**2**_0.2_**6**_0.8_was isolated after 6 h in the presence of 50 equivalents **7** (Fig. 4b and c and S29†). Alternatively, the composition of AuNP-**2**_x_**6**_y_ mixed-ligand monolayers could be controlled by adjusting the stoichiometry of trapping aldehyde **7** and allowing the exchange to reach thermodynamic equilibrium (Fig. S30 and S31, Tables S14 and S15†). Nanoparticle-bound aldehydes exhibited remarkable stability, and importantly, no evidence of aldehyde oxidation or other decomposition reactions was observed even at elevated temperatures under the acidic conditions required for dynamic covalent exchange reactions (Fig. S35, S36 and Table S19†). We demonstrated that the aldehyde-functionalized monolayers retained their reactivity by treating AuNP-**2**_0.2_**6**_0.8_ with hydrazide **4** (0.8 molar equivalents; equimolar with respect to nanoparticle-bound **6**) to produce mixed hydrazone monolayer AuNP-**2**_0.2_**3**_0.8_ (Fig. 4b and c). Timecourse monitoring of this reaction (Fig. S32, Table S16†) revealed that in fact some hydrazide **5** is released from the monolayer at early time points, likely as a result of direct transimination of hydrazone **2** to **3** (*vide infra*). Over time, however, the system evolves to an equilibrium endpoint where all aldehydes are converted to hydrazones with the composition that exactly reflects the stoichiometric ratio of hydrazide components added.

The combined capabilities to directly exchange hydrazone functionality and to selectively reveal controlled quantities of reactive aldehydes now present opportunities for a wide diversity of reversible and irreversible covalent reactions to produce polyfunctional monolayers that combine several structurally diverse constituents in precisely controlled ratios. To do so reliably, however, demands an in-depth understanding of molecular reactivity in the nanoparticle-bound environment.

## Kinetic analysis of hydrazone exchange in complementary nanoparticle-bound monolayers

**Fig. 5:**
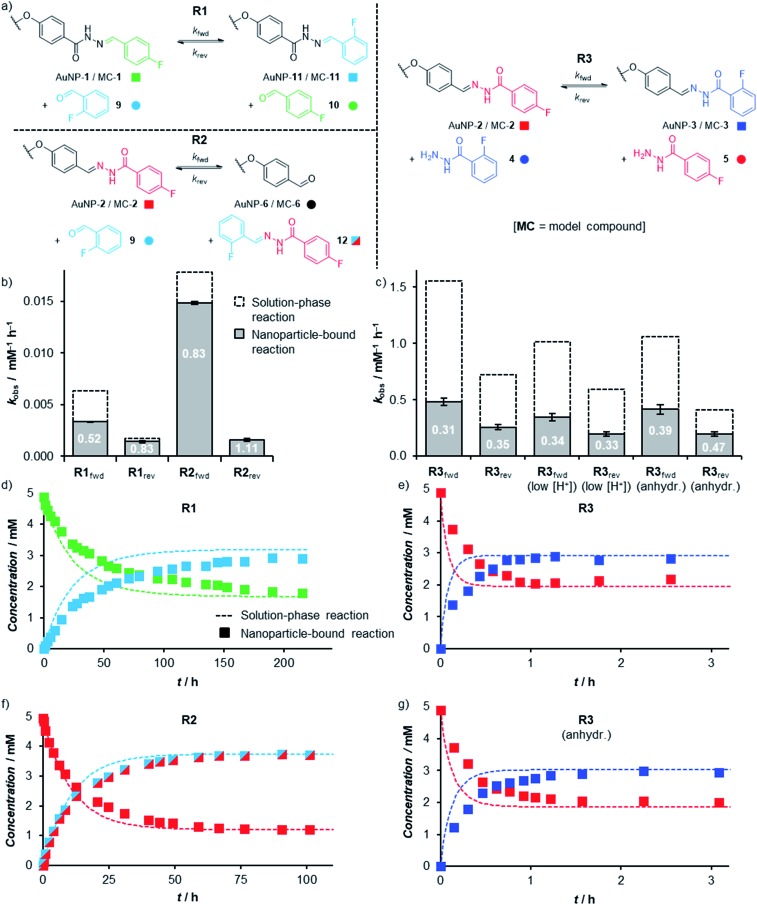
(a) The three hydrazone exchange processes **R1–R3** investigated in bulk solution (starting materials MC-**1** or MC-**2**) and on nanoparticle-bound monolayers (starting materials AuNP-**1** or AuNP-**2**). (b and c) Observed rate constants and inhibition factors [*k*(NP-bound)/*k*(bulk)] for hydrazone exchange reactions with electrophilic modifiers (b, **R1**, **R2**) and nucleophilic modifiers (c, **R3**) taking place in bulk solution (dashed columns) and within nanoparticle-bound monolayers (filled columns). Each value is the mean of triplicate experiments; for numerical values, see Tables S20 and S21.† (d–g) Representative kinetic profiles comparing hydrazone exchange reactions performed on nanoparticle-bound monolayers (filled symbols), and bulk solution substrates (dashed lines): (d) **R1**; (e) **R3**; (f) **R2**; (g) **R3** (anhydr.).

The ability to achieve high concentrations of nanoparticle-bound reactive groups in colloidal solution enables the application of non-destructive analytical techniques that can attain molecular sensitivity. Using ^19^F NMR spectroscopy, concentrations of both unbound solution-phase and nanoparticle-bound species could each be quantified against an internal standard of known concentration to track surface-confined dynamic covalent reactions *in situ*. Thereby kinetic profiles were established for transformations of both ‘nucleophilic’ (AuNP-**1**, reaction **R1**) and ‘electrophilic’ (AuNP-**2**, reactions **R2** and **R3**) dynamic covalent nanoparticles, reacting with electrophilic (reactions **R1** and **R2**) or nucleophilic (reaction **R3**) modifiers (Fig. 5, Tables S20 and S21†).^19^ The reaction of corresponding model compounds (MC-**1** and MC-**2**) in bulk solution provided the crucial comparison between nanoparticle-bound and unbound reactivity. All reactions were performed at a concentration of *ca.* 5 mM in starting hydrazone in DMF, and when present, a large excess of water (10% v/v ≈ 1000 equivalents). Examining hydrazone exchange in the presence of an equimolar quantity of modifier and an excess of CF_3_CO_2_H, nearly every nanoparticle-bound reaction is significantly slower than its bulk-solution analogue (compare filled and unfilled bars, Fig. 5b and c; and compare data points with lines, Fig. 5d–g), although the rate constants remain within the same order of magnitude (Tables S20 and S21†). This was in-line with our expectations for reaction in the monolayer environment,^5^ where steric crowding may be expected on account of the spatial proximity of neighbouring ligands. Notably, however, the magnitude of kinetic retardation varies between nanoparticle substrates and reaction conditions, providing instructive mechanistic insights (*vide infra*). Small differences in equilibrium position between surface-confined and bulk-solution scenarios observed in some cases can likewise be expected on account of different intermolecular interactions experienced in each environment.

In bulk solution, hydrazone exchange with electrophilic modifiers (**R1**, **R2**) is intrinsically much slower than reaction in the presence of just 1 equivalent of a nucleophilic hydrazide modifier (**R3**),^20^ suggesting that under these conditions, exchange *via* a direct transimination mechanism is kinetically more significant than the alternative hydrolysis–condensation pathway.^21^ The same relationship is maintained in the nanoparticle-bound environment: reaction between AuNP-**2** and 1 equivalent hydrazide **4** (reaction **R3**) reaches equilibrium within 1 h (Fig. 5e), whereas the analogous process for nucleophilic AuNP-**1** with 1 equivalent **9** (reaction **R1**) has not fully equilibrated even after 10 days at room temperature (Fig. 5d). The kinetic profiles for each reaction were fitted to give observed second order rate constants in each direction (*k*_fwd_ and *k*_rev_, Fig. 5b and c, Tables S20 and S21†).^22^,^23^ Initial rate constants were estimated from the linear portion of the reaction progress plots (Tables S22–S32†) and showed good agreement with the rate constants fitted to the full time-course data, suggesting that there is no significant change in reaction rate or mechanism as the reactions proceed.^24^

The efficiency of the transimination mechanism was confirmed by reaction in the absence of water, where the exchange of AuNP-**2** with 1 equivalent hydrazide **4** still reached equilibrium within 2.5 h (Fig. 5g), corresponding to a reduction in the forward and reverse second-order rate constants by only modest factors of 0.86 and 0.76 over reaction in the presence of water.^25^ The reactivity difference for AuNP-**2** over AuNP-**1** is further enhanced by electronic factors: the electron-rich 4-alkoxybenzylidine hydrazone of AuNP-**2** being significantly more reactive than the electron-poor 2-fluoro or 4-fluorobenzylidine hydrazones of AuNP-**1** (*e.g.* compare **R2_fwd_** and **R1_fwd_**); curiously this electronic effect appears to be more significant for reaction in the nanoparticle-bound environment compared to bulk solution.^20^

It is notable that the faster reaction with a nucleophilic modifier experiences a more significant rate reduction on transfer from bulk solution to the nanoparticle surface (compare filled and unfilled bars, Fig. 5b and c). Several effects could contribute to the difference in reaction rates between the two environments, including: (1) reduced efficiency of nucleophilic attack at a sterically crowded and conformationally restricted surface-bound electrophile;24a–c,^26^ (2) alteration of p*K*_a_ for key intermediates in the surface-bound monolayer;^27^ (3) differential rates for proton transfer steps involving surface-bound *versus* bulk solution intermediates.^28^ Our results suggest that the reduced efficiency of nucleophilic attack is an important factor, so that pathways involving the sterically bulkier hydrazide nucleophiles (reaction **R3**) are more significantly affected by surface confinement.^29^ When water is the only nucleophile available, relatively small surface inhibitions are observed in most cases (reactions **R1_fwd_**, **R1_rev_**, **R2_fwd_**). Furthermore, a negligible difference in surface-bound *versus* bulk-solution rate constants for **R2_rev_** is consistent with the fact that the rate-determining nucleophilic attack of water takes place on a solution-phase molecular species (**12**), irrespective of whether the initial substrate was MC-**2** or AuNP-**2**. A final point of interest is that the effect of surface confinement is greatest when the transimination and hydrolysis–condensation mechanisms operate simultaneously (compare **R3** with **R3** (anhydr.) and with **R2**). As transimination is the kinetically dominant pathway rather than attack by water nucleophiles (*vide supra*), this suggests that water also plays another role in the rate difference between nanoparticle-bound and bulk-solution reactions by directly affecting the transimination pathway. This would be consistent with a more tightly packed monolayer in more polar solvent environments, which would further hinder the nanoparticle-bound over the bulk-solution reaction when water is present. Alternatively, water may be involved in catalysing key proton transfer steps in bulk solution,^30^ but may be less efficient in performing the same role for intermediates confined within the monolayer.

From a practical perspective, we were pleased to observe that dynamic covalent modification of electrophilic nanoparticle-bound monolayers can be achieved under even milder conditions. The rapid reaction between AuNP-**2** and **4** still attained equilibrium in under 1.5 h in the presence of only 5 mM CF_3_CO_2_H (1 equivalent relative to hydrazone, Fig. S37†). No change in the surface-associated inhibition in reaction rate was observed (factors of 0.34 and 0.33 for the forward and reverse reactions, **R3_f_**(low [H^+^]) and **R3_r_**(low [H^+^]) respectively, Table S21†), indicating no change to the rate determining step(s) at this lower acid concentration.

Comparative information regarding reactivity in extended 2D self-assembled monolayers is limited to isolated studies on specialized systems, many of which do not allow for direct comparison to solution-phase analogues.^31^ This reflects the paucity of analytical techniques that can achieve the sensitivity required for molecular-level analysis of the extremely low concentrations of surface-bound species in heterogeneous systems. By contrast, colloidally stable nanoparticles can be considered as ‘pseudomolecular’ species. Their large surface-to-volume ratios provide surface-confined environments in sufficiently high concentration to allow application of solution-phase molecular analytical techniques. From a fundamental perspective, therefore, monolayer-stabilized nanoparticles have a critical role to play as model systems capable of providing new insight on molecular interactions and reactivity in surface-confined environments.

## Adaptive assembly of complementary dynamic covalent nanoparticle building blocks

A complementary pair of reactive nanoparticles presents the opportunity for modifications not only using molecular reagents of appropriate reactivity, but also direct reaction between nanoparticles to create multi-component assemblies. The resulting aggregates are constructed *via* robust covalent connections, but are at the same time endowed with the responsive characteristics of dynamic covalent chemistry, leading to adaptive and reconfigurable behaviours.

**Fig. 6:**
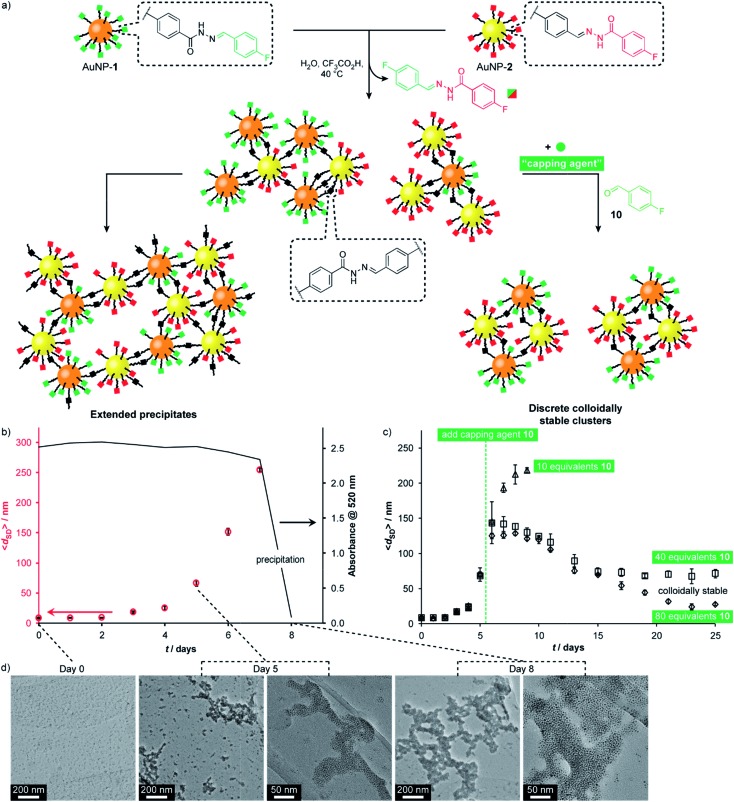
(a) Assembly of complementary nucleophilic (AuNP-**1**) and electrophilic (AuNP-**2**) dynamic covalent nanoparticles to produce extended networks (b and d) or discrete colloidally stable clusters (c). (b) Average hydrodynamic diameter () dynamic covalent nanoparticles to produce extended networks (b and d) or discrete colloidally stable clusters (c). (b) Average hydrodynamic diameter (〈*d*_SD_〉) for complementary assembly, as measured by DLS, and absorbance at 520 nm over time. (c) Average hydrodynamic diameter (〈) for complementary assembly, as measured by DLS, and absorbance at 520 nm over time. (c) Average hydrodynamic diameter (〉) for complementary assembly, as measured by DLS, and absorbance at 520 nm over time. (c) Average hydrodynamic diameter (〈*d*_SD_〉), as measured by DLS, showing the effect of introducing different concentrations of monofunctional capping agent ), as measured by DLS, showing the effect of introducing different concentrations of monofunctional capping agent **10** at day 5. (d) Representative TEM images at 0, 5 and 8 days. Conditions (all experiments): 0.15 mM overall surface-bound hydrazones, 9 : 1 v/v DMF/H_2_O, 20 mM CF_3_COOH, 40 °C. Full UV-Vis absorption spectra, an expansion of solvodynamic sizes observed over days 1–4, and additional TEM images are reported in the ESI.†

Having firmly established reversible hydrazone formation within the monolayers of ‘nucleophilic’ AuNP-**1** and ‘electrophilic’ AuNP-**2**, we investigated the reaction between this complementary pair of dynamic covalent nanoparticles to directly assemble binary nanoparticle aggregates (Fig. 6). A 1 : 1 mixture of AuNP-**1** and AuNP-**2**, each at 0.075 mM (in terms of hydrazone ligand),^14^ in H_2_O/DMF (10% v/v) was treated with CF_3_CO_2_H (20 mM). The red-brown solution showed little change by eye until day 8, when complete precipitation was observed, leaving a colourless supernatant. Monitoring by dynamic light scattering (DLS, Fig. 6b and S48†) revealed intermediate stages in the assembly process. The average solvodynamic size measured by DLS () revealed intermediate stages in the assembly process. The average solvodynamic size measured by DLS (〈*d*_SD_〉) showed no significant change over the first two days (〈) showed no significant change over the first two days (〉) showed no significant change over the first two days (〈*d*_SD_〉 ≈ 9 nm). Rapid growth of colloidally stable aggregates was then observed, reaching 〈 ≈ 9 nm). Rapid growth of colloidally stable aggregates was then observed, reaching 〉 ≈ 9 nm). Rapid growth of colloidally stable aggregates was then observed, reaching 〈*d*_SD_〉 = 254 nm at day 7, after which complete precipitation occurred by day 8. Monitoring the concentration of nanoparticles in solution by UV-Vis absorption at the surface plasmon resonance ( = 254 nm at day 7, after which complete precipitation occurred by day 8. Monitoring the concentration of nanoparticles in solution by UV-Vis absorption at the surface plasmon resonance (*λ*_ma*x*_ = 520 nm) indicated that the clusters formed over days 1–7 remained colloidally stable; whereas virtually no material remained in suspension following the rapid precipitation process between days 7 and 8 (Fig. 6b and S41†).

TEM imaging of dried samples prepared from the suspension at day 5 (TEM imaging of dried samples prepared from the suspension at day 5 (〈*d*_SD_〉 = 67 nm) revealed a mixture of small clusters, larger aggregates, and a few isolated nanoparticles, whereas the suspension produced on sonication of the solid sample at day 8 revealed almost quantitative assembly of both types of nanoparticle into extended binary aggregates ( = 67 nm) revealed a mixture of small clusters, larger aggregates, and a few isolated nanoparticles, whereas the suspension produced on sonication of the solid sample at day 8 revealed almost quantitative assembly of both types of nanoparticle into extended binary aggregates (Fig. 6d).^32^,^33^ Importantly, control experiments omitting either the acid catalyst or one of the complementary nanoparticles showed no change in solvodynamic diameter, UV-Vis absorption or appearance when imaged by electron microscopy (Fig. S42–S44 and S49–S51†), indicating that it is the direct reaction between complementary DCNPs that is responsible for the assembly process.

Directing nanoparticle assembly through specific and reversible molecular processes presents the opportunity to influence the nanoscale aggregation outcome by applying appropriate molecular triggers. For example, a monotopic capping agent such as 4-fluorobenzaldehyde (**10**) could modify the complementary binary assembly process in a concentration-dependent manner (Fig. 6c). On adding 10 equivalents of **10** to the equilibrating system at day 5, colloidally stable assemblies reached a smaller maximum size ( to the equilibrating system at day 5, colloidally stable assemblies reached a smaller maximum size (〈*d*_SD_〉 = 218 nm) and persisted in solution longer before precipitation occurred at day 9. Adding higher concentrations of monotopic = 218 nm) and persisted in solution longer before precipitation occurred at day 9. Adding higher concentrations of monotopic **10** prevented precipitation entirely. Instead, the system evolved through a maximum in aggregate size to reach an equilibrium endpoint characterized by persistent colloidally stable aggregates with narrow size distributions that are dependent on the concentration of monotopic unit (40 eq. **10**: : 〈*d*_SD_〉 ≈ 70 nm; 80 eq. ≈ 70 nm; 80 eq. **10**: : 〈*d*_SD_〉 ≈ 30 nm). In both these experiments, UV-Vis absorption spectroscopy confirmed that virtually all material remained in colloidal suspension even after 25 days (Fig. S46 and S47 ≈ 30 nm). In both these experiments, UV-Vis absorption spectroscopy confirmed that virtually all material remained in colloidal suspension even after 25 days (Fig. S46 and S47†).

Controlling nanoparticle assembly is vital for interfacing nanomaterials with existing technologies,1*e* yet few strategies are available for selectively combining two or more nanoscale components, far less with control over aspects of aggregate morphology.2f–i,9a,d,^34^ Complementary DCNPs are programmed to selectively produce binary aggregates bestowed with the responsive properties of the dynamic covalent molecular process. Adaptive assemblies of molecular building blocks have previously been achieved by introducing terminating units to control the degree of polymerization for dynamic polymers produced by noncovalent^35^ or dynamic covalent bonds.35b,^36^ Using DCNPs, remarkably, the same principle can be applied to direct nanometer-scale self-assemblies of building blocks *ca.* 250 kDa in size, using only a structurally simple low molecular weight (<150 Da) molecular unit. We can thus now look forward to the same level of programmable control over nanoparticle assembly as currently achieved for the assembly of molecular building blocks.

## Conclusions

We have shown that a pair of dynamic covalent nanoparticles with complementary reactivity institutes a programmable toolkit of nanoscale building blocks offering comprehensive constitutional control over both molecular structure and composition of nanoparticle-bound molecular monolayers. ‘Electrophilic’ hydrazone-functionalized DCNPs react with nucleophilic molecular modifiers to rapidly exchange surface-bound hydrazones. Alternatively, reaction with electrophilic molecular scavengers reveals surface-bound aldehydes. Products spanning the full range of mixed-ligand compositions to exhaustive exchange between single-component monolayers are readily accessible by either tuning the stoichiometry of the thermodynamically governed exchange processes, or simply removing the acid catalyst to kinetically arrest the reaction. Likewise, electrophilic modifications of ‘nucleophilic’ DCNPs are achieved using similar equilibrium-controlled processes.

In place of molecular modifiers, complementary dynamic covalent nanoparticles are also programmed to react directly with each other, producing aggregates comprising two different nanoparticle constituents that are linked by strong covalent bonds, which can subsequently be rendered kinetically stable. The adaptive and reversible characteristics of the underlying molecular-level process are conferred on these nanoscale assemblies so that product constitution can again be manipulated by changes to the reaction conditions, as demonstrated by concentration-dependent control of cluster size by a monofunctional molecular capping agent.

Unrestricted by the structural complexity and environmental constraints of biomolecules or abiotic noncovalent systems, this toolkit opens the door to a wide array of customizable nanoparticle-bound molecular structures and chemical reactivity. In contrast to alternative approaches, dynamic covalent modification avoids the considerable synthetic effort required to prepare each surface-stabilizing ligand in its entirety, and the ensuing empirical re-optimization of nanoparticle synthesis and purification protocols. By separating the nanoparticle preparation and modification steps, nanoparticle size and shape distributions are unaffected by the target monolayer ligand structures, and functional groups that would be unstable under other preparation routes can be introduced.

In the absence of the acid catalyst required for dynamic covalent exchange, nanoparticle-bound hydrazones are kinetically stable, allowing the nanoparticle products to be rigorously purified, stored, and re-suspended, even at low concentrations, with no change to the monolayer composition. Together with the high density of surface-bound reactive sites, this allows for the application of non-destructive analytical techniques that can achieve quantitative *in situ* molecular structural analysis and real-time reaction monitoring. With this platform it is therefore possible to begin to establish mechanistic understanding and structure–reactivity relationships for reactions in the unconventional nanoparticle-bound environment. General reactivity trends were found to translate from bulk solution into the interfacial setting. However, surface confinement has a more significant kinetic effect on the exchange reactions of ‘electrophilic’ hydrazone-based dynamic covalent nanoparticles than their ‘nucleophilic’ counterparts. Nevertheless, ‘electrophilic’ surface-bound monolayers are modified rapidly, even under anhydrous conditions and at low concentrations of acid catalyst, *via* a direct transimination pathway. With this level of mechanistic insight, one can anticipate a set of rational design rules for divergent chemospecific modifications of nanoparticle-bound species, and the programmable integration of multiple nanoscale and molecular components to access a range of polyfunctional products and materials, all from just a small number of nanoparticle starting points. Such capabilities will underpin the next generation of synthetic chemistry that combines chemically active nanoscale, supramolecular and molecular entities with equal proficiency.

## Conflicts of interest

There are no conflicts to declare.

## Supplementary Material

Supplementary information
