# Supplementary material for: Programmable dynamic covalent nanoparticle building blocks with complementary reactivity
Source: Chem Sci. 2019 Nov 14;11(2):372–83. doi: 10.1039/c9sc04195h (PMC7067244; doi:10.1039/c9sc04195h)
Supplement: Supplementary file 1 [file SC-011-C9SC04195H-s001.pdf]

Electronic Supplementary Material (ESI) for Chemical Science.
This journal is © The Royal Society of Chemistry 2019

Supporting Information

Programmable dynamic covalent nanoparticle building blocks with complementary reactivity

*Nicolas Marro, Flavio della Sala and Euan R. Kay**

1. General experimental procedures S2
2. Synthesis of organic compounds S3
3. Synthesis and characterisation of 'nucleophilic' 4-fluorobenzylidene terminated nanoparticles (AuNP-**1**) S13
4. Synthesis and characterisation of 'electrophilic' 4-fluorobenzoylhydrazide terminated nanoparticles (AuNP-**2**) S21
5. Synthesis and characterisation of 'electrophilic' 2-fluorobenzoylhydrazide terminated nanoparticles (AuNP-**3**) S30
6. Dynamic covalent modification of 'electrophilic' nanoparticles with nucleophilic modifiers S32
   - 6.1 Generation and characterization of AuNP-**3**(e) and mixed monolayer compositions AuNP-$\mathbf{2}_x\mathbf{3}_y$ S32
   - 6.2 Generation and characterization of AuNP-**2**(e) S42
   - 6.3 Verification of nanoparticle integrity following dynamic covalent modification S45
7. Dynamic covalent modification of 'electrophilic' nanoparticles with electrophilic modifiers S47
   - 7.1 Generation of single-component aldehyde-functionalized monolayers AuNP-**6** S47
   - 7.2 Generation of two-component hydrazone/aldehyde monolayers AuNP-$\mathbf{2}_x\mathbf{6}_y$ S52
   - 7.3 Stability tests on nanoparticle-bound aldehydes S62
8. Kinetic studies of dynamic covalent reactions S65
   - 8.1 Hydrazone exchange S65
   - 8.2 Hydrazone hydrolysis S73
9. Complementary nanoparticle assembly S77
   - 9.1 Monitoring by UV-Vis absorbance spectroscopy S78
   - 9.2 Monitoring by dynamic light scattering S81
   - 9.3 Monitoring by transmission electron microscopy S83
10. ^{1}H, ^{13}C and ^{19}F NMR spectra of organic compounds S86
11. References S103

1. General experimental procedures

Unless stated otherwise, all reagents were purchased from commercial sources and used without further purification. Dry *N*,*N*-dimethylformamide was purchased from Across Organics. All other dry solvents were obtained by means of a MBRAUN MB SPS-800TM solvent purification system, where solvents were passed through filter columns and dispensed under an argon atmosphere. Flash column chromatography was performed using Geduran® Si60 (40–63 µm, Merck, Germany) as stationary phase. Thin-layer chromatography (TLC) was performed on pre-coated silica gel plates (0.25 mm thick, 60F254, Merck, Germany) and observed under UV light (λ_{max} 254 nm) or visualized by staining with acidic ceric ammonium molybdate solution, followed by heating. Nanoparticle micrographs were obtained using a JEM 2010 transmission electron microscope (TEM). Samples were prepared by deposition of one drop of nanoparticle suspension on holey carbon films supported on a 300 mesh Cu grid (Agar Scientific®). Nanoparticle diameters were measured automatically using the software ImageJ. The images were first converted to black and white images using the "Threshold" function. The area of each nanoparticle was measured using the "Analyze particles" function. Particles on edges were excluded. UV-vis absorption spectroscopy was performed on a Thermo Scientific Evolution 220 UV-Visible Spectrophotometer using a quartz cuvette (10 mm path length). Dynamic light scattering (DLS) measurements were performed on a Malvern Zetasizer µV instrument using a glass cuvette (10 mm path length). Each data point is the average of three independent measurements made in series. In turn, each measurement is the average of 13–19 sequential scans. The solvodynamic sizes are reported as the mean size for distributions expressed as % number of particles (plots of the distributions expressed as both % number of particles and % particle volume). Size distributions were calculated by the instrument from the recorded intensity data using the appropriate values for viscosity, refractive index and dielectric constant estimated for binary mixtures. Such values have been calculated according to equations reported in the literature.[1] ^{1}H, ^{13}C, ^{19}F NMR spectra were recorded on Bruker Avance II 300, 400, 500 and 700 MHz instruments, at a constant temperature of 25 °C. ^{1}H Chemical shifts are reported in parts per million (ppm) from high to low field and referenced to the literature values for chemical shifts of residual non-deuterated solvent, with respect to tetramethylsilane.[2] ^{19}F Chemical shifts are referenced to $CFCl_3$ (0.00 ppm) as external standard. Standard abbreviations indicating multiplicity are used as follows: bs (broad singlet), d (doublet), m (multiplet), q (quartet), s (singlet), t (triplet), J (coupling constant). All spectra were analyzed using MestReNova (Version 10.0.2). All melting points were determined using a Stuart SMP30 Melting Point Apparatus and are reported uncorrected. Freeze drying was achieved using a Christ Alpha 1–2 LD Freeze dryer (Martin Christ GmbH, Osterode am Harz, Germany) at −54 °C, 0.15 mbar vacuum for ca. 15–20 h until complete dryness. Laser desorption ionization mass spectrometry (LDI-MS) was performed by applying a drop of nanoparticle solution (0.5 µL) to a MALDI target followed by air drying. The spectrum was then acquired using a 4800 MALDI TOF/TOF analyser (ABSciex, Foster City, CA) equipped with a Nd:YAG 355 nm laser and calibrated using a mixture of peptides. No external matrix was required for gold nanoparticle samples, which are capable of absorbing the laser excitation energy directly. For molecular samples, 2,5-dihydroxybenzoic acid or *trans*-2-[3-(4-*tert*-butylphenyl)-2-methyl-2-propenylidene] malononitrile were used to promote ionisation. Samples were analysed in positive and negative MS mode over the appropriate m/z range.

2. Synthesis of organic compounds

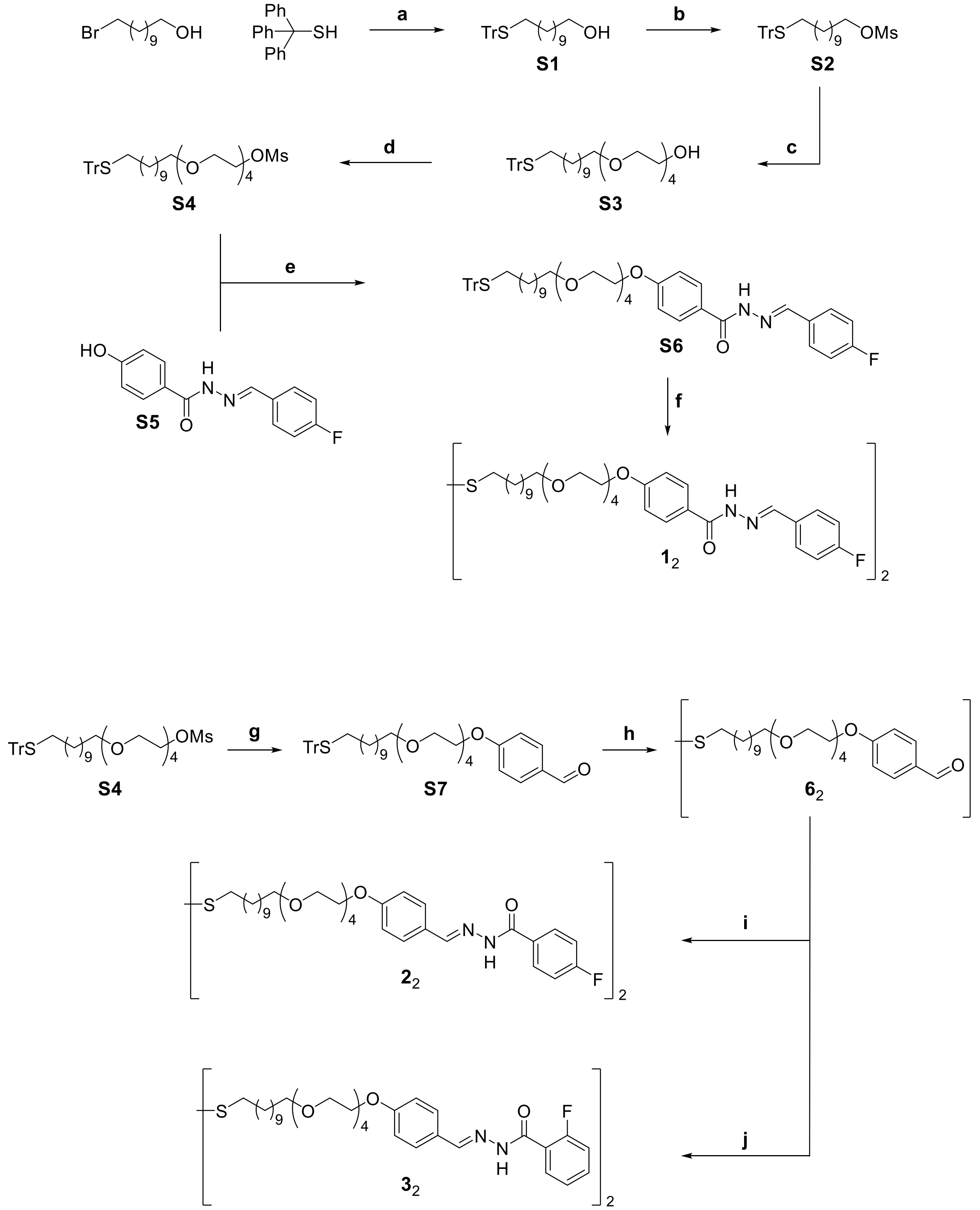

Scheme S1. Synthetic route to disulfide pro-ligands **1**$_2$, **2**$_2$, **3**$_2$. Reagents and conditions: a) NaOH, H_2O, 1:1 *v*/*v* EtOH/toluene, rt, 8 h, 91%; b) MsCl, Et_3N, CH_2Cl_2, 0 °C / rt, 3.5 h, 94%; c) tetraethylene glycol, NaOH, H_2O, 90 °C, 18 h, 88%; d) MsCl, Et_3N, CH_2Cl_2, 0 °C / rt, 4 h, 79%; e) K_2CO_3, KI, DMF, 80 °C, 18 h, 60%; f) I_2, 4:1 *v*/*v* CH_2Cl_2/MeOH, rt, 1.5 h, 86%; g) 4-hydroxybenzaldehyde, K_2CO_3, KI, DMF, 80 °C, 14 h, 80%; h) I_2, 3:1 *v*/*v* CH_2Cl_2/MeOH, rt, 1 h, 85%; i) 4-fluorobenzohydrazide, 3:1 *v*/*v* CH_2Cl_2/MeOH, AcOH, rt, 15 h, 88%; j) 2-fluorobenzohydrazide, 3:1 *v*/*v* CH_2Cl_2/MeOH, AcOH, rt, 8 h, 87%.

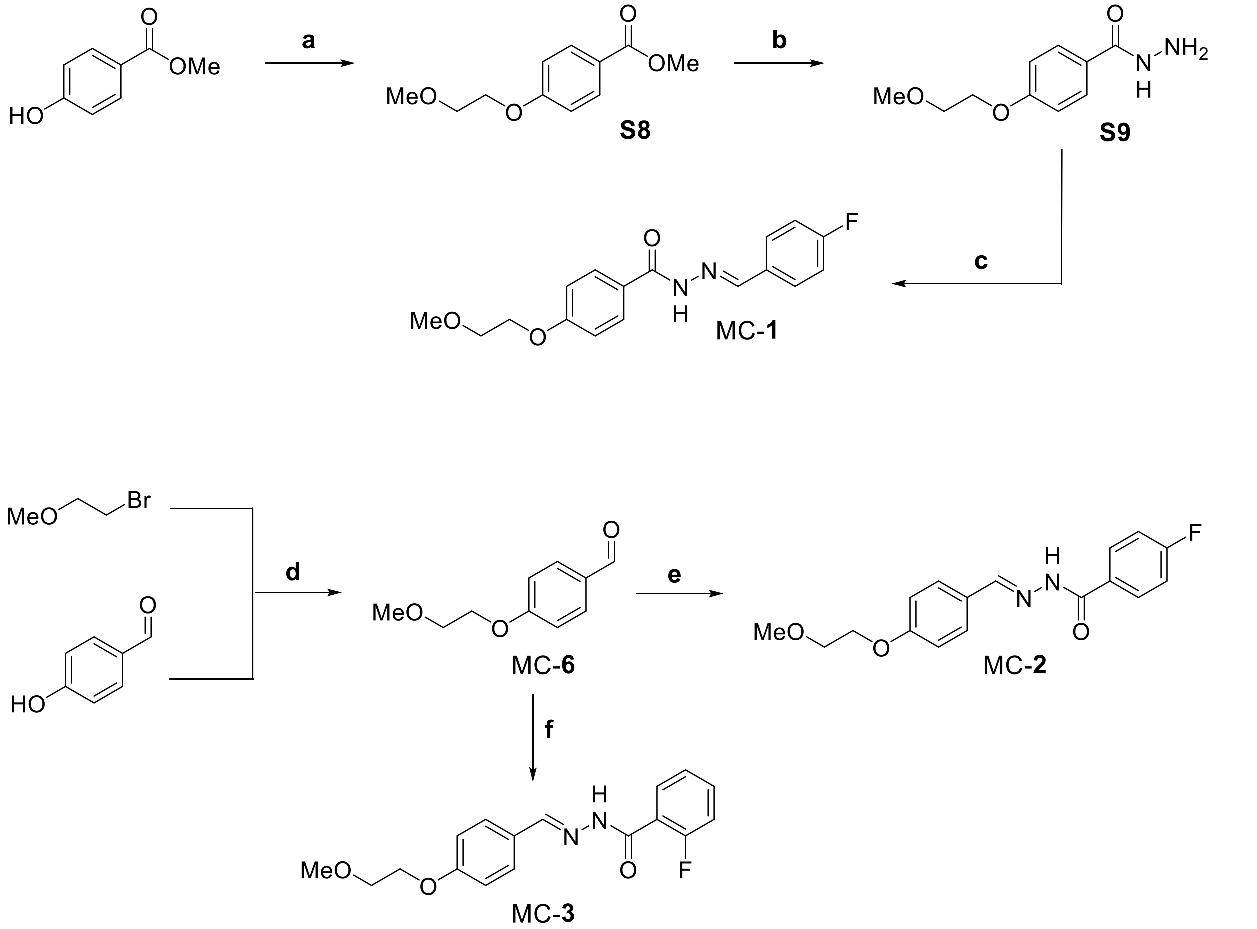

Scheme S2. Synthetic route to hydrazone and aldehyde model compounds MC-**1**, MC-**2** MC-**3** and MC-**6**. Reagents and conditions: a) 2-bromoethyl methyl ether, K_2CO_3, KI, DMF, 80 °C, 6 h, 82%; b) H_2NNH_2•H_2O, MeOH, reflux, 15 h, 76%;c) 4-fluorobenzaldehyde, MeOH, AcOH, rt, 10 h, 62%; d) K_2CO_3, KI, DMF, 80 °C, 18 h, 81%; e) 4-fluorobenzoylhydrazide, 3:1 v/v CH_2Cl_2/MeOH, AcOH, rt, 15 h, 78%; f) 2-fluorobenzoylhydrazide, 3:1 v/v CH_2Cl_2/MeOH, AcOH, rt, 13 h, 72%.

11-(Tritylthio)undecan-1-ol (S1)[3]

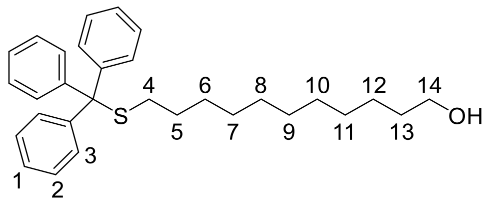

Triphenylmethanethiol (12.0 g, 43.4 mmol) was dissolved in 1:1 *v*/*v* EtOH/toluene (50 mL). Then a solution of NaOH (2.25 g, 56.4 mmol) in H_2O (6 mL) was added and the reaction was stirred at rt for 5 min. Finally, a solution of bromoundecanol (9.91 g, 39.5 mmol) in 1:1 *v*/*v* EtOH/toluene (50 mL) was added and the reaction mixture was stirred at rt for 8 h. The mixture was poured in 100 mL of saturated aqueous $NaHCO_3$ and extracted with Et_2O (3×100 mL). The combined organic layers were washed with saturated brine (2×150 mL), dried over $MgSO_4$ and the solvent was removed under reduced pressure. The crude yellow oil obtained was then purified by flash chromatography (SiO_2, 8:1 to 1:1 *v*/*v* HexH/EtOAc), to afford the alcohol **S1** as a pale-yellow oil. Obtained 16.1 g, yield 91%. Spectral data in agreement with the literature.[3]

^{1}H NMR (500 MHz, $CDCl_3$): δ 1.16–1.46 (m, 16H, 5–12), 1.55–1.61 (m, 2H, 13), 2.18 (t, *J* = 6.9 Hz, 2H, 4), 3.63 (t, *J* = 7.0 Hz, 2H, 14), 7.21–7.24 (m, 3H, 1), 7.28–7.32 (m, 6H, 3), 7.44–7.47 (m, 6H, 2) ppm.

^{13}C NMR (126 MHz, $CDCl_3$): δ 25.8, 28.6, 29.0, 29.2, 29.4, 29.5, 29.5, 29.6, 30.9, 32.0, 32.8, 62.9, 126.5, 127.8, 129.6, 145.1 ppm.

11-(Tritylthio)undecyl methanesulfonate (S2)[3]

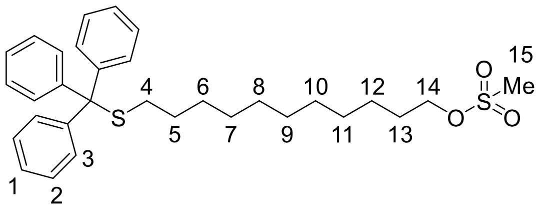

A solution of alcohol **S1** (10.1 g, 22.6 mmol) and Et_3N (7.87 mL, 56.6 mmol) in CH_2Cl_2 (90 mL) was cooled to 0 °C. Methanesulfonyl chloride (3.51 mL, 45.2 mmol) in CH_2Cl_2 (15 mL) was then added dropwise, stirring the mixture at 0 °C. The reaction was warmed to rt and stirred for a further 3 h. The solvent was then removed under reduced pressure and the crude oil was re-dissolved in CH_2Cl_2 (100 mL) and washed with 0.1 M HCl (2 × 100 mL), saturated aqueous $NaHCO_3$ (2 × 100 mL) and saturated brine (100 mL). The organic phase was dried over $MgSO_4$ and evaporated under reduced pressure. The crude yellow oil obtained was then purified by flash chromatography (SiO_2, 3:1 to 3:2 *v*/*v* HexH/EtOAc), to afford compound **S2** as a colourless oil. Obtained 11.1 g, yield 94%. Spectral data in agreement with the literature.[3]

^{1}H NMR (500 MHz, $CDCl_3$): δ 1.18–1.44 (m, 16H, 5–12), 1.73–1.79 (m, 2H, 13), 2.17 (t, *J* = 7.3 Hz, 2H, 4), 2.98 (s, 3H, 15), 4.23 (t, *J* = 7.0 Hz, 2H, 14), 7.20–7.24 (m, 3H, 1), 7.28–7.32 (m, 6H, 3), 7.43–7.47 (m, 6H, 2) ppm.

^{13}C NMR (126 MHz, $CDCl_3$): δ 25.5, 28.7, 29.1, 29.2, 29.3, 29.4, 29.4, 32.1, 32.9, 34.2, 37.5, 66.5, 70.3, 126.6, 127.9, 129.7, 145.2 ppm.

1,1,1-Triphenyl-14,17,20,23-tetraoxa-2-thiapentacosan-25-ol (S3)[3]

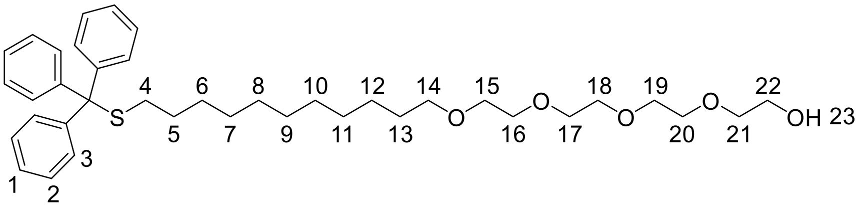

A solution of NaOH (1.83 g, 41.7 mmol) in H_2O (4 mL) was added to tetraethylene glycol (138 g, 709 mmol) and stirred at 90 °C for 1 h. Compound **S2** (21.9 g, 41.7 mmol) was then added to the reaction mixture and stirred at 90 °C for a further 18 h. The solution was then left to cool at rt, poured into H_2O (200 mL) and extracted with Et_2O (5 × 100 mL). The combined organic layers were washed with saturated aqueous $NaHCO_3$ (3 × 150 mL) and saturated brine (3 × 150 mL), dried over $MgSO_4$ and evaporated under reduced pressure to give the desired product **S3** as a pale-yellow oil. Obtained 20.3 g, yield 88%. Spectral data in agreement with the literature.[3]

^{1}H NMR (500 MHz, $CDCl_3$): δ 1.12–1.31 (m, 14H, 6–12), 1.33–1.40 (m, 2H, 5) 1.53–1.61 (m, 2H, 13), 2.12 (t, *J* = 7.4 Hz, 2H, 4), 3.26 (bs, 1H, 23), 3.43 (t, *J* = 6.8 Hz, 2H, 14), 3.55–3.67 (m, 14H, 15–21), 3.70 (t, *J* = 3.8 Hz, 2H, 22), 7.17–7.20 (m, 3H, 1), 7.24–7.28 (m, 6H, 3), 7.40–7.44 (m, 6H, 2) ppm.

^{13}C NMR (126 MHz, $CDCl_3$): δ 26.1, 28.5, 29.0, 29.1, 29.3, 29.4, 29.4, 29.5, 29.5, 32.0, 61.5, 66.3, 70.0, 70.1, 70.4, 70.4, 70.5, 70.6, 71.5, 72.7, 126.4, 127.7, 129.5, 145.0 ppm.

1,1,1-triphenyl-14,17,20,23-tetraoxa-2-thiapentacosan-25-yl methanesulfonate (S4)[3]

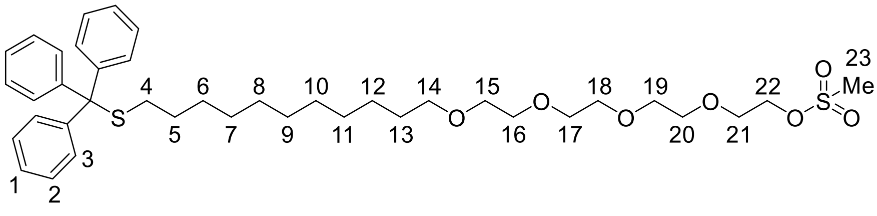

A solution of alcohol **S3** (23.4 g, 37.6 mmol) and Et_3N (11.5 mL, 82.6 mmol) in CH_2Cl_2 (180 mL) was cooled to 0 °C. Methanesulfonyl chloride (3.82 mL, 48.8 mmol) in CH_2Cl_2 (20 mL) was then added

dropwise, stirring the mixture at 0 °C. The reaction was then warmed to rt and stirred for a further 4 h. The solvent was removed under reduced pressure and the crude oil was re-dissolved in CH_2Cl_2 (180 mL) and washed with 0.1 M HCl (2 × 150 mL), saturated aqueous $NaHCO_3$ (2 × 150 mL) and saturated brine (200 mL). The organic phase was dried over $MgSO_4$ and evaporated under reduced pressure. The crude yellow oil obtained was then purified by flash chromatography (SiO_2, 1:3 *v/v* HexH/EtOAc), to afford compound **S4** as a colourless oil. Obtained 20.8 g, yield 79%. Spectral data in agreement with the literature.[3]

^{1}H NMR (500 MHz, $CDCl_3$): δ 1.15–1.35 (m, 14H, 6–12), 1.35–1.42 (m, 2H, 5) 1.53–1.60 (m, 2H, 13), 2.13 (t, *J* = 7.3 Hz, 2H, 4), 3.07 (s, 3H, 23), 3.45 (t, *J* = 6.8 Hz, 2H, 14), 3.55–3.68 (m, 12H, 15–20), 3.73–3.77 (m, 2H, 21), 4.36–4.39 (m, 2H, 22), 7.17–7.21 (m, 3H, 1), 7.24–7.29 (m, 6H, 3), 7.38–7.42 (m, 6H, 2) ppm.

^{13}C NMR (126 MHz, $CDCl_3$): δ 26.2, 27.0, 28.7, 29.0, 29.2, 29.3, 29.5, 29.6, 29.7, 29.8, 32.2, 37.9, 45.3, 69.2, 69.4, 70.2, 70.3, 70.6, 70.72, 70.77, 71.7, 126.6, 127.9, 129.7, 145.2 ppm.

***N'*-(4-Fluorobenzylidene)-4-hydroxybenzohydrazide (S5)**[3]

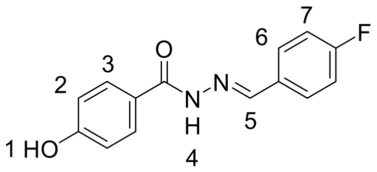

4-Hydroxybenzhydrazide (1.25 g, 8.24 mmol) was dissolved in MeOH (150 mL). Then, 4-fluorobenzaldehyde (0.88 mL, 8.26 mmol) and AcOH 6% v/v (10 mL) were added. The reaction mixture was stirred at rt for 19 h. The solvent was then evaporated under reduced pressure obtaining a white solid which was redissolved in hot EtOH and precipitated with cold water, affording **S5** as a white solid. Obtained 2.02 g, yield 95%. Spectral data in agreement with the literature.[3]

^{1}H NMR (500 MHz, $(CD_3)_2SO$): δ 6.86 (d, *J* = 8.5 Hz, 2H, 2), 7.29 (t, *J* = 8.7 Hz, 2H, 7), 7.74–7.82 (m, 4H, 3 and 6), 8.43 (s, 1H, 5), 10.14 (bs, 1H, 1), 11.67 (bs, 1H, 4) ppm.

^{13}C NMR (75 MHz, $(CD_3)_2SO$): δ 115.0, 115.9 (d, *J* = 22 Hz), 123.8, 129.1 (d, *J* = 8.6 Hz), 129.7, 131.1 (d, *J* = 2.8 Hz), 145.7, 160.7, 162.9 (d, *J* = 248 Hz), 162.8 ppm.

^{19}F NMR (470 MHz, $(CD_3)_2SO$): δ –110.45 (s, 1F) ppm.

***N'*-(4-Fluorobenzylidene)-4-((1,1,1-triphenyl-14,17,20,23-tetraoxa-2-thiapentacosan-25-yl) oxy)benzohydrazide (S6)**[3]

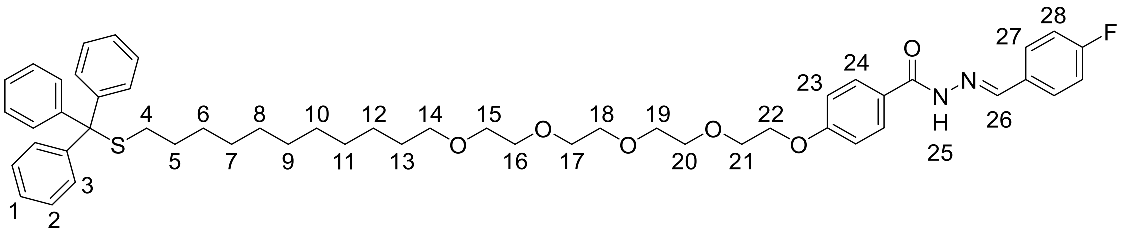

Hydrazone **S5** (0.76 g, 3.04 mmol) K_2CO_3 (1.26 g, 9.12 mmol) and KI (0.29 g, 1.76 mmol) were dissolved in dry DMF (25 mL) and stirred at 80 °C for 20 min. Then, a solution of **S4** (1.77 g, 2.52 mmol) in dry DMF (25 mL) was added dropwise over 30 min and the reaction was heated at 80 °C for 14 h. The mixture was then allowed to cool to rt before saturated brine (50 mL) was added. The aqueous layer was extracted with CH_2Cl_2 (3 x 100 mL). The combined organic layers were dried over $MgSO_4$, filtered and evaporated under reduced pressure. The crude oil was purified by chromatography (SiO_2, 6:1 *v/v* CH_2Cl_2/THF 6:1) to afford **S6** as a colourless oil. Obtained 1.13 g, yield 52%. Spectral data in agreement with the literature.[3]

^{1}H NMR (500 MHz, $CDCl_3$): δ 1.08–1.32 (m, 14H, 6–12), 1.35–1.42 (m, 2H, 5), 1.53–1.59 (m, 2H, 13), 2.12 (t, *J* = 8.5 Hz, 2H, 4), 3.42 (t, *J* = 8.5 Hz, 2H, 14), 3.56–3.74 (m, 12H, 15–20), 3.86–3.91 (m, 2H, 21), 4.16–4.22 (m, 2H, 22), 6.96 (d, *J* = 7.4 Hz, 2H, 23), 7.09 (t, *J* = 8.0 Hz, 2H, 28), 7.18–7.22 (m, 3H, 1), 7.25–7.29 (m, 6H, 3), 7.38–7.42 (m, 6H, 2), 7.66–7.79 (m, 2H, 27), 7.77–7.92 (m, 2H, 24), 8.31 (s, 1H, 26), 9.40 (bs, 1H, 25) ppm.

^{13}C NMR (101 MHz, CD_2Cl_2): δ 26.7, 29.1, 29.5, 29.6, 29.7, 29.9, 30.0, 30.1, 30.3, 32.4, 66.8, 68.2, 70.0, 70.6, 71.0, 71.0, 71.1, 71.2, 71.3, 71.9, 114.9, 116.2 (d, *J* = 22 Hz), 116.4, 127.0, 128.3, 129.7, (d, *J* = 8.1 Hz), 129.9, 130.1, 130.6, 131.0 (d, *J* = 2.8 Hz), 145.7, 160.6, 162.4, 164.4 (d, *J* = 250 Hz) ppm.

^{19}F NMR (470 MHz, (CD_2Cl_2): δ –110.19 (s, 1F) ppm.

4,4'-((3,6,9,12,37,40,43,46-octaoxa-24,25-dithiaoctatetracontane-1,48-diyl)bis(oxy)) bis(*N*'-((*E*)-4-fluorobenzylidene)benzohydrazide) (1_2)[3]

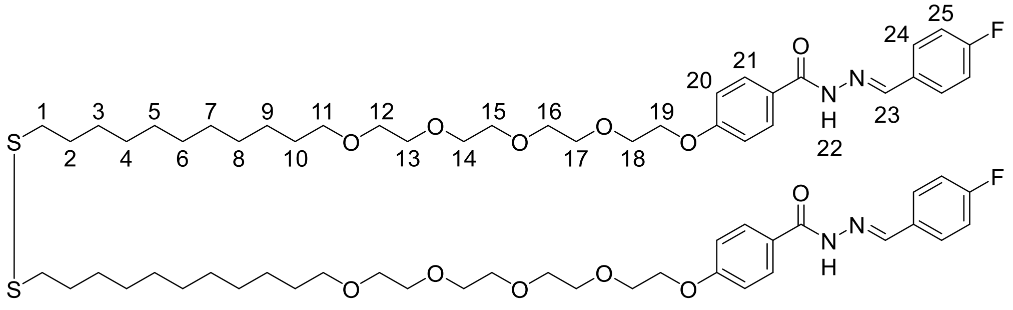

Iodine (0.16 g, 0.64 mmol) was added to a solution of compound **S5** (0.28 g, 0.32 mmol) in MeOH (20 mL). The mixture was stirred at rt for 1.5 h, then, the excess of iodine was removed by addition of saturated aqueous $NaHSO_3$. The resulting solution was extracted with CH_2Cl_2 (3 × 20 mL). Organics were combined, dried over $MgSO_4$ and evaporated under reduced pressure. The crude oil was purified by chromatography (SiO_2, 10:0.7:0.2 *v*/*v* CH_2Cl_2/THF/MeOH) to afford $\mathbf{1}_2$ as a colourless oil. Obtained 0.21g, yield 95%. Spectral data in agreement with the literature.[3]

^{1}H NMR (500 MHz, $CDCl_3$): δ 1.23–1.34 (m, 28H, 3–9), 1.51–1.55 (m, 4H, 2), 1.61–1.68 (m, 4H, 10), 2.66 (t, *J* = 7.5 Hz, 4H, 1), 3.40 (t, *J* = 6.5 Hz, 4H, 11), 3.51–3.72 (m, 24H, 12–17), 3.72–3.86 (m, 4H, 18), 3.98–4.15 (m, 4H, 19), 6.80–6.95 (m, 4H, 20), 6.97–7.08 (m, 4H, 25), 7.50–7.58 (dd, *J* = 7.6, 5.5 Hz, 24), 7.86–8.03 (d, *J* = 8.0 Hz, 4H, 21), 8.35 (s, 2H, 23), 10.32 (bs, 2H, 22) ppm.

^{13}C NMR (126 MHz, $CDCl_3$): δ 26.2, 28.6, 29.3, 29.3, 29.5, 29.6, 29.7, 29.7, 29.8, 39.3, 67.6, 69.7, 70.1, 70.6, 70.7, 70.7, 70.8, 71.0, 71.7, 114.5, 115.9 (d, *J* = 22 Hz), 125.6, 129.6 (d, *J* = 8.1 Hz), 130.3, 130.9 (d, *J* = 1.9 Hz), 147.1, 161.9, 164.1 (d, *J* = 250 Hz), 165.2 ppm.

^{19}F NMR (376 MHz, $CDCl_3$): δ –110.18 (s, 2F) ppm.

4-((1,1,1-triphenyl-14,17,20,23-tetraoxa-2-thiapentacosan-25-yl)oxy)benzaldehyde (S7)

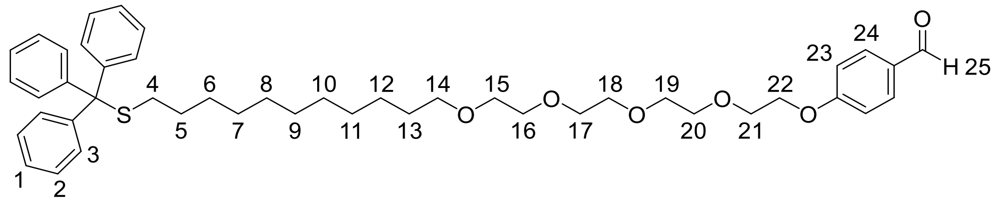

4–Hydroxybenzaldehyde (1.10 g, 8.82 mmol), K_2CO_3 (3.51 g, 25.2 mmol) and KI (0.92 g, 5.45 mmol) were dissolved in dry DMF (120 mL) and the reaction mixture was stirred at 80 °C for 40 min. Then, a solution of compound **S4** (5.9 g, 8.4 mmol) in dry DMF (20 mL) was added dropwise over 30 min and the reaction was left to stir at 80 °C for a further 14 h. Then, the mixture was cooled to rt and the solvent removed under reduced pressure. The crude oil obtained was re-dissolved in EtOAc (180 mL). The organic phase was washed with H_2O (1 x 150 mL) and saturated brine (2 x 150 mL), dried over $MgSO_4$, filtered and solvent removed under reduced pressure. The crude yellow oil obtained was then purified by chromatography (SiO_2, 1:3 *v*/*v* HexH/EtOAc) to afford compound **S7** as a rose oil. Obtained 4.92 g, yield 80%.

^{1}H NMR (400 MHz, $CDCl_3$): δ 1.20–1.31 (m, 14H, 6–12), 1.34–1.41 (m, 2H, 5), 1.53–1.59 (m, 2H, 13), 2.14 (t, *J* = 7.3 Hz, 2H, 4), 3.45 (t, *J* = 6.8 Hz, 2H, 14), 3.55–3.75 (m, 12H, 15–20), 3.87–3.91 (m, 2H, 21), 4.19–4.22 (m, 2H, 22),

7.04 (d, *J* = 8.4 Hz, 2H, 23), 7.22–7.26 (m, 3H, 1), 7.28–7.34 (m, 6H, 3), 7.42–7.45 (m, 6H, 2), 7.84 (d, *J* = 8.5 Hz, 2H, 24), 9.92 (s, 1H, 25) ppm.

^{13}C NMR (101 MHz, $CDCl_3$): δ 26.1, 28.6, 29.0, 29.2, 29.4, 29.5, 29.5, 29.6, 29.7, 32.0, 60.4, 66.4, 67.8, 69.5, 70.1, 70.6, 70.7, 70.9, 71.2, 71.6, 114.9, 126.5, 127.8, 129.6, 130.0, 132.0, 145.1, 163.9,190.8 ppm.

MALDI-MS calculated *m/z* for $C_{45}H_{58}NaO_6S$ $[M+Na]^+$ = 749,3846, found 749.3868.

4,4'-((3,6,9,12,37,40,43,46-octaoxa-24,25-dithiaoctatetracontane-1,48-diyl)bis(oxy)) dibenzaldehyde (6$_2$)

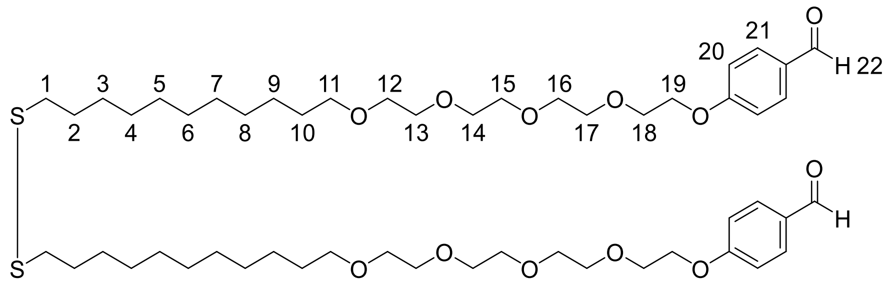

Compound **S7** (5.37 g, 7.29 mmol) was dissolved in 3:1 *v/v* CH_2Cl_2/MeOH (220 mL). Iodine (2.81 g, 10.9 mmol) was added and the mixture was stirred at rt for 1 h. The excess of iodine was quenched by addition of saturated aqueous $NaHSO_3$ (100 ml). The solvent was partially removed under reduced pressure and the resulting residue was poured in to saturated brine (120 mL). The aqueous phase was extracted with CH_2Cl_2 (3 x 150 mL). Organics were combined, dried over $MgSO_4$, filtered and evaporated under reduced pressure. The crude yellow oil obtained was then purified by chromatography (SiO_2, 10:1 *v/v* CH_2Cl_2/THF) to afford compound **6**$_2$ as a colourless oil. Obtained 3.13 g, yield 85%.

^{1}H NMR (400 MHz, $CDCl_3$): δ 1.26–1.44 (m, 28H, 3–9), 1.54–1.60 (m, 4H, 2), 1.63–1.71 (m, 4H, 10), 2.69 (t, *J* = 7.3 Hz, 4H, 1), 3.44 (t, *J* = 6.7 Hz, 4H, 11), 3.56–3.74 (m, 24H, 12–17), 3.88–3.92 (m, 4H, 18), 4.21–4.24 (m, 4H, 19), 7.01–7.05 (m, 4H, 20), 7.82–7.86 (m, 4H, 21), 9.90 (s, 2H, 22) ppm.

^{13}C NMR (101 MHz, $CDCl_3$): δ 26.1, 28.5, 29.2, 29.3, 29.5, 29.5, 29.6, 29.6, 30.3, 39.2, 67.8, 69.5, 70.1, 70.6, 70.6, 70.8, 70.9, 71.5, 71.7, 114.9, 130.0, 131.9, 163.8,190.8 ppm.

MALDI-MS calculated *m/z* for $C_{52}H_{86}NaO_{12}S_2$ $[M+Na]^+$ = 989.5453, found 989.5473.

***N'*,*N'''*-((1*E*,1'*E*)-(((3,6,9,12,37,40,43,46-Octaoxa-24,25-dithiaoctatetracontane-1,48-diyl)bis (oxy))bis(4,1-phenylene))bis(methanylylidene))bis (4-fluorobenzohydrazide) (2$_2$)**

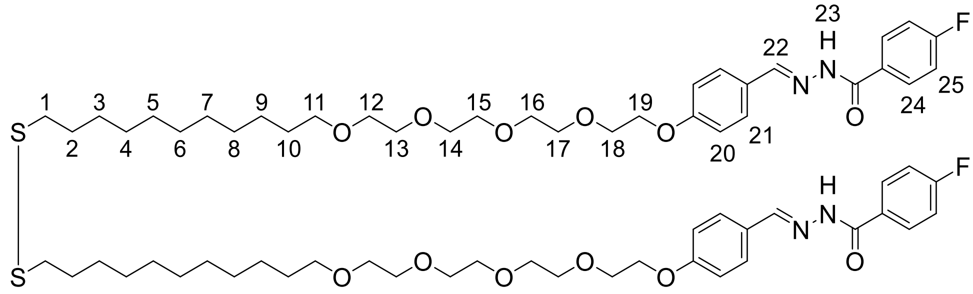

Compound **6**$_2$ (1.10 g, 1.11 mmol) was dissolved in 3:1 *v/v* CH_2Cl_2/MeOH (20 mL). Then, 4-fluorobenzohydrazide (0.35 g, 2.33 mmol) and AcOH 6% *v/v* (1.20 mL) were added and the mixture was stirred at rt for 15 h. Once the reaction was completed, the mixture was poured in fresh CH_2Cl_2 (100 mL) and washed with saturated aqueous $NaHCO_3$ (3 x 50 mL) and saturated brine (50 mL). The organic layer was dried over $MgSO_4$, filtered and the solvent was removed under reduced pressure. The crude yellow oil obtained was then purified by chromatography (SiO_2, 10:0.7:0.2 to 10:0.7:0.3 *v/v* CH_2Cl_2/THF/MeOH) to afford compound **2**$_2$ as a pale-yellow oil. Obtained 1.22 g, yield 88%.

^{1}H NMR (500 MHz, $CDCl_3$): δ 1.22–1.38 (m, 28H, 3–9), 1.48–1.54 (m, 4H, 2), 1.60–1.66 (m, 4H, 10), 2.65 (t, J = 7.3 Hz, 4H, 1), 3.40 (t, J = 6.7 Hz, 4H, 11), 3.51–3.68 (m, 24H, 12–17), 3.76–3.85 (m, 4H, 18), 3.98–4.13 (m, 4H, 19), 6.71–6.80 (m, 4H, 20 (major *E-anti*-conformer)), 6.84–6.91 (m, 4H, 20 (minor *E-syn*-conformer)), 7.01–7.07 (m, 4H, 25 (major *E-anti*-conformer)), 7.08–7.16 (m, 4H, 25 (minor *E-syn*-conformer)), 7.43–7.50 (m, 4H, 21 (minor *E-syn*-conformer)), 7.51–7.60 (m, 4H, 21 (major *E-anti*-conformer)), 7.80–7.86 (m, 4H, 24 (minor *E-syn*-conformer)), 7.89–7.99 (m, 4H, 24 (major *E-anti*-conformer)), 8.03 (s, 2H, 22 (minor *E-syn*-conformer)), 8.29 (s, 2H, 22 (major *E-anti*-conformer)), 9.85 (bs, 2H, 23 (minor *E-syn*-conformer)),10.08 (bs, 2H, 23 (major *E-anti*-conformer)) ppm.

^{13}C NMR (126 MHz, $CDCl_3$): δ 26.2, 28.6, 29.3, 29.4, 29.5, 29.6, 29.6, 29.6, 29.7, 39.3, 67.5, 69.7, 70.1, 70.2, 70.6, 70.7, 70.7, 71.5, 71.6, 114.7, 115.7 (d, J = 21.8 Hz), 126.6, 129.3, 129.6, 130.1 (d, J = 9.0 Hz), 149.1, 160.7, 163.5, 164.9 (d, J = 249 Hz), ppm.

^{19}F NMR (470 MHz, $CDCl_3$): δ –107.76 (s, 2F, major *E-anti*-conformer), –108.27 (s, 2F, (minor *E-syn*-conformer) ppm.

MALDI-MS calculated m/z for $C_{66}H_{96}F_2N_4NaO_{12}S_2$ $[M+Na]^+$ = 1261.6326, found 1261.5799.

_N'_,_N'''_-((1_E_,1'_E_)-(((3,6,9,12,37,40,43,46-Octaoxa-24,25-dithiaoctatetracontane-1,48-diyl) bis(oxy))bis(4,1-phenylene))bis(methanylylidene))bis (2-fluorobenzohydrazide) (3$_2$)

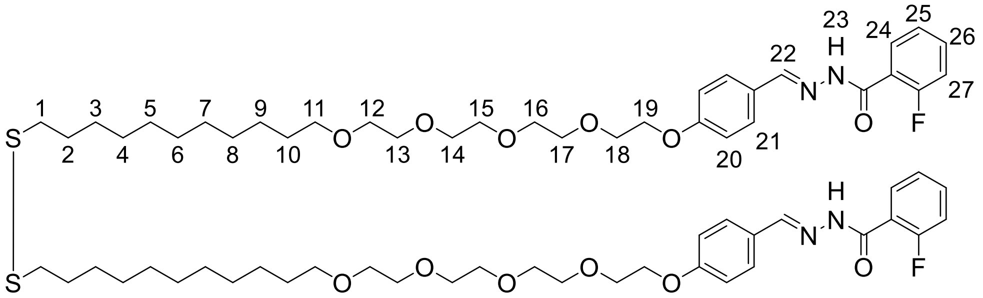

Compound **6**$_2$ (0.12 g, 0.13 mmol) was dissolved in 3:1 *v*/*v* CH_2Cl_2/MeOH (9 mL). Then, 2-fluorobenzohydrazide (420 mg, 0.24 mmol) and AcOH 6% v/v (0.42 mL) were added. The mixture was left stirring at rt for 18 h. Once the reaction was complete, the mixture was poured in to fresh CH_2Cl_2 (30 mL), washed with saturated aqueous $NaHCO_3$ (3 x 20 mL) and saturated brine (20 mL). Then the organic layer was dried over $MgSO_4$, filtered and the solvent removed under reduced pressure. The crude pale-yellow oil obtained was purified by chromatography (SiO_2, 10:0.7:0.2 to 10:0.7:0.3 *v*/*v* CH_2Cl_2/THF/MeOH), to afford **3**$_2$ as a colourless oil. Obtained 0.14 g, yield 87%.

^{1}H NMR (500. MHz, $CDCl_3$): δ 1.27–1.41 (m, 28H, 3–9), 1.55–1.61 (m, 4H, 2), 1.64–1.70 (m, 4H, 10), 2.68 (t, J = 7.2 Hz, 4H, 1), 3.45 (t, J = 6.8 Hz, 4H, 11), 3.54–3.77 (m, 24H, 12–17), 3.87–3.92 (m, 4H, 18), 4.20–4.24 (m, 4H, 19), 6.86 (d, J = 9.3 Hz, 4H, 20, (minor *E-syn*-conformer)), 6.95 (d, J = 8.7 Hz, 4H, 20, (major *E-anti*-conformer)), 7.15–7.20 (m, 2H, 27), 7.30–7.35 (m, 2H, 25), 7.38–7.43 (m, 4H, 21, (minor *E-syn*-conformer)), 7.51–7.55 (m, 2H, 26, (major *E-anti*-conformer)), 7.56–7.60 (m, 2H, 26, (minor *E-syni*-conformer)) 7.74 (d, J = 8.7 Hz, 4H, 21, (major *E-syn*-conformer)), 8.16–8.22 (m, 4H, 22 and 24), 9.38 (bs, 2H, 23 (minor *E-syn*-conformer)), 9.67 (d, J = 13.7 Hz, 2H, 23 (major *E-anti*-conformer)) ppm.

^{13}C NMR (126 MHz, $CDCl_3$): δ 26.1, 28.5, 29.2, 29.3, 29.5, 29.5, 29.6, 29.9, 30.1, 39.2, 67.5, 69.6, 70.0, 70.5, 70.6, 70.8, 70.9, 71.5, 71.8, 114.8, 116.0 (d, J = 24.7 Hz), 120.1 (d, J = 13.2 Hz), 125.2 (d, J = 3.1 Hz), 126.2, 129.5, 132.4, 133.8 (d, J = 9.3 Hz), 148.6, 159.8 (d, J = 3.0 Hz), 160.1 (d, J = 204.5 Hz) ppm.

^{19}F NMR (470 MHz, $CDCl_3$): δ –112.54 (s, 2F, (minor *E-syn*-conformer)), –113.79 (s, 2F, (major *E-anti*-conformer)) ppm.

Methyl 4-(2-methoxyethoxy)benzoate (S8)

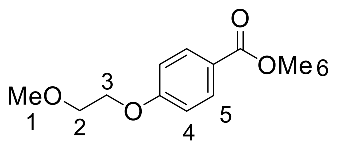

Methyl 4-hydroxybenzoate (0.56 g, 3.61 mmol), K_2CO_3 (1.50 g, 10.9 mmol) and KI (0.41 g, 2.52 mmol) were dissolved in dry DMF (20 mL) and stirred at 80 °C for 20 min. Then a solution of 2-bromoethyl methyl ether (0.36 mL, 3.80 mmol) in dry DMF (4 mL) was added dropwise over 10 min and the reaction was heated at 80 °C for 6 h. The mixture was then allowed to cool to rt, before EtOAc (50 mL) was added. The organic phase was washed with H_2O (50 mL) and saturated brine (2 x 50 mL), dried over $MgSO_4$, filtered and evaporated under reduced pressure to afford **S8** as a yellow oil. Obtained 0.63 g, yield 82%. Spectral data in agreement with the literature.[3]

^{1}H NMR (500 MHz, $CDCl_3$): δ 3.47 (s, 3H, 1), 3.78–3.40 (m, 2H, 3), 3.90 (s, 3H, 6), 4.18–4.20 (m, 2H, 2), 6.96 (d, *J* = 9.0 Hz, 2H, 4), 8.00 (d, *J* = 8.5 Hz, 2H, 5) ppm.

^{13}C NMR (126 MHz, $CDCl_3$): δ 52.0, 59.4, 67.5, 70.9, 114.3, 122.9, 131.7, 162.6, 167.0 ppm.

4-(2-Methoxyethoxy)benzohydrazide (S9)

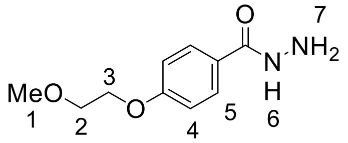

Compound **S7** (1.25 g, 5.94 mmol) was dissolved in MeOH (30 mL). Hydrazine monohydrate (2.88 mL, 59.4 mmol) was added and the reaction mixture was refluxed for 15 h. The mixture was then cooled to rt, diluted with H_2O (50 mL) and extracted with CH_2Cl_2 (3 x 50 mL). The organic phase was dried over MgSO4, filtered and evaporated under reduced pressure, obtaining a white solid. The crude product was redissolved in hot EtOH and precipitated with HexH, affording **S9** as a white solid. Obtained 0.95 g, yield 76%. Spectral data in agreement with the literature.[3]

^{1}H NMR (500 MHz, $CDCl_3$): δ 3.45 (s, 3H, 1), 3.74–3.77 (m, 2H, 3), 4.09 (bs, 2H, 7), 4.14–4.17 (m, 2H, 2), 6.94 (d, *J* = 9.0 Hz, 2H, 4), 7.54 (bs, 1H, 6), 7.71 (d, *J* = 9.0 Hz, 2H, 5) ppm.

^{13}C NMR (126 MHz, $CDCl_3$): δ 59.4, 67.5, 70.9, 114.6, 125.2, 128.8, 161.8, 168.4 ppm.

***N'*-(4-Fluorobenzylidene)-4-(2-methoxyethoxy)benzohydrazide (MC-1)**

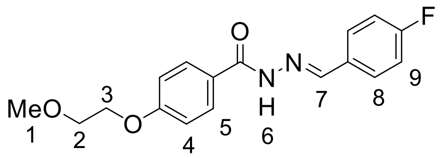

Compound **S9** (1.00 g, 4.76 mmol) was dissolved in 1:1 *v/v* CH_2Cl_2/MeOH (50 mL). Then, 4-fluorobenzaldehyde (0.54 mL, 5.03 mmol) and AcOH 6% v/v (3 mL) were added. The reaction mixture was stirred for 22 h at rt, then the solvent was evaporated under reduced pressure. The crude white solid obtained was then purified by chromatography (SiO_2, 8:1 v/v CH_2Cl_2/THF) to afford compound MC-**1** as a white solid. Obtained 1.31 g, yield 87% Spectral data in agreement with the literature.[3]

^{1}H NMR (400 MHz, $(CD_3)_2SO$): δ 3.32 (s, 3H, 1), 3.67–3.69 (m, 2H, 3), 4.17–4.19 (m, 2H, 2), 7.07 (d, *J* = 8.4 Hz, 2H, 4), 7.30 (t, *J* = 7.3 Hz, 2H, 9), 7.76–7.81 (m, 2H, 8), 7.90 (d, *J* = 8.4 Hz, 2H, 5), 8.44 (s, 1H, 7), 11.75 (bs, 1H, 6) ppm.

^{13}C NMR (101 MHz, $(CD_3)_2SO$): δ 58.2, 67.2, 70.3, 114.2, 115.9 (d, *J* = 22 Hz), 125.4, 129.2 (d, *J* = 8.1 Hz), 129.5, 131.1 (d, *J* = 2.2 Hz), 146.0, 161.3, 162.5, 163.2 (d, *J* = 247 Hz) ppm.

^{19}F NMR (376 MHz, $(CD_3)_2SO$): δ –110.85 (s, 1F) ppm.

4-(2-Methoxyethoxy)benzaldehyde (MC-6)

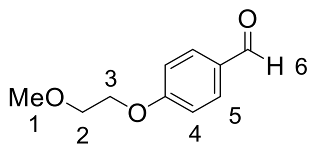

4-Hydroxybenzaldehyde (1.04 g, 8.18 mmol), K_2CO_3 (3.37 g, 24.5 mmol) and KI (0.98 g, 5.7 mmol) were dissolved in dry DMF (40 mL) and the reaction mixture was stirred for 10 min at 80 °C. Then, a solution of 2-bromoethyl methyl ether (0.79 mL, 8.5 mmol) in dry DMF (7 ml) was added dropwise over 20 min and the mixture stirred at 80 °C for a further 12 h. After cooling the reaction to rt, EtOAc (80 mL) was added. The organic phase was washed with saturated aqueous LiCl (2 x 60 mL), saturated brine (3 x 60 mL), dried over $MgSO_4$, filtered and the solvent removed under reduced pressure. The crude yellow obtained was purified by chromatography (SiO_2, 10:1 *v*/*v* CH_2Cl_2/Et_2O) to afford MC-**6** as a colourless oil. Obtained 1.19 g, yield 81%.

^{1}H NMR (400 MHz, $CDCl_3$): δ 3.47 (s, 3H, 1), 3.78–3.80 (m, 2H, 3), 4.20–4.22 (m, 2H, 2), 7.04 (d, *J* = 8.1Hz, 2H, 4), 7.84 (d, *J* = 8.2Hz, 2H, 5), 9.89 (s, 1H, 6) ppm.

^{13}C NMR (101 MHz, $CDCl_3$): δ 59.4, 67.7, 70.8, 115.0, 130.2, 132.0, 163.9, 190.9 ppm.

HRMS (ES$^+$) calculated m/z for $C_{10}H_{13}O_3$ $[M+H]^+$ 181.0865, found 181.0857.

4-Fluoro-*N'*-(4-(2-methoxyethoxy)benzylidene)benzohydrazide (MC-2)

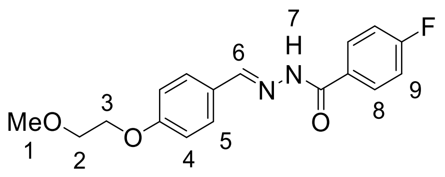

Compound MC-**6** (0.24 g, 1.29 mmol) and 4-fluorobenzohydrazide (0.25 g, 1.32 mmol) were dissolved in 2:1 *v*/*v* CH_2Cl_2/MeOH (20 mL) in presence of AcOH 6% *v*/*v* (1.20 mL). The reaction mixture was left stirring at rt for 7 h. Then, fresh CH_2Cl_2 (40 mL) was added and the organic phase was washed with saturated aqueous $NaHCO_3$ (3 x 25 mL), saturated brine (25 mL), dried over $MgSO_4$, filtered and the solvent removed under reduced pressure. The crude solid obtained was then purified by chromatography (SiO_2, 8:1 v/v CH_2Cl_2/THF) to afford MC-**2** as a white solid. Obtained 0.32 g, yield 78%. Mp: 158–160 °C.

^{1}H NMR (500 MHz, $(CD_3)_2SO$): δ 3.31 (s, 3H, 1), 3.67–3.69 (m, 2H, 3), 4.14–4.17 (m, 2H, 2), 6.97 (d, *J* = 8.7 Hz, 2H, 4, (minor *E-syn*-conformer)), 7.03 (d, *J* = 8.7 Hz, 2H, 4, (major *E-anti*-conformer)), 7.37 (t, *J* = 8.8 Hz, 2H, 9), 7.50 (d, *J* = 8.8 Hz, 2H, 5, (minor *E-syn*-conformer)), 7.66 (d, *J* = 8.8 Hz, 2H, 5, (major *E-anti*-conformer)), 7.88–7.92 (m, 2H, 8, (minor *E-syn*-conformer)), 7.97–8.00 (m, 2H, 8, (major *E-anti*-conformer)), 8.03 (bs, 1H, 6, (minor *E-syn*-conformer)), 8.38 (bs, 2H, 6, (major *E-anti*-conformer)), 11.64 (bs, 1H, 7, (minor *E-syn*-conformer)), 11.75 (bs, 2H, 8, (major *E-anti*-conformer)) ppm.

^{13}C NMR (126 MHz, $(CD_3)_2SO$): δ 58.2, 67.1, 70.3, 114.8, 115.3 (d, *J* = 21.9 Hz), 126.9, 128.7, 130.0, 130.2 (d, *J* = 9.1 Hz), 147.7, 160.1, 163.1, 163.5 (d, *J* = 245 Hz) ppm.

^{19}F NMR (470 MHz, $(CD_3)_2SO$): δ –108.10 (s, 1F, (major *E-anti*-conformer)), –108.90 (s, 1F, (minor *E-syn*-conformer)) ppm.

HRMS (ES$^+$) calculated m/z for $C_{17}H_{18}FN_2O_3$ $[M+H]^+$ 317.1301, found 317.1294.

2-Fluoro-*N*'-(4-(2-methoxyethoxy)benzylidene)benzohydrazide (MC-3)

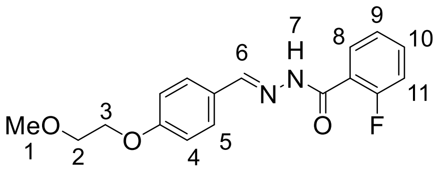

Compound MC-**6** (0.18 g, 1.13 mmol) and 2-fluorobenzohydrazide (0.17 g, 1.19 mmol) were dissolved in 2:1 *v*/*v* CH_2Cl_2/MeOH (15 mL) in presence AcOH 6% *v*/*v* (0.90 mL). The reaction mixture was left stirring at rt for 8 h. Then, fresh CH_2Cl_2 (30 mL) was added and the organic phase was washed with saturated aqueous $NaHCO_3$ (3 x 20 mL), saturated brine (20 mL), dried over $MgSO_4$, filtered and the solvent removed under reduced pressure. The crude solid obtained was then purified by chromatography (SiO_2, 8:1 *v*/*v* CH_2Cl_2/THF) to afford MC-**3** as a white solid. Obtained 0.24 g, yield 69%. Mp: 135–137 °C.

^{1}H NMR (400 MHz, $(CD_3)_2SO$): δ 3.28 (s, 3H, 1, (major *E*-*anti*-conformer)), 3.32 (s, 3H, 1, (major *E*-*anti*-conformer)), 3.61–3.64 (m, 2H, 3, (minor *E*-*anti*-conformer)), 3.65–3.69 (m, 2H, 3, (major *E*-*anti*-conformer)), 4.07–4.10 (m, 2H, 2, (minor *E*-*anti*-conformer)), 4.13–4.16 (m, 2H, 2, (major *E*-*anti*-conformer)), 6.92 (d, *J* = 8.7 Hz, 2H, 4, (minor *E*-*anti*-conformer)), 7.03 (d, *J* = 8.7 Hz, 2H, 4, (major *E*-*anti*-conformer)), 7.27–7.37 (m, 2H, 9 and 11), 7.47–7.61 (m, 2H, 8 and 10), 7.66 (d, *J* = 8.8 Hz, 2H, 5), 8.01 (s, 1H, 6, (minor *E*-*anti*-conformer)), 8.26 (bs, 2H, 6, (major *E*-*anti*-conformer)), 11.72 (bs, 1H, 7) ppm.

^{13}C NMR (126 MHz, $(CD_3)_2SO$): δ 58.2, 67.1, 70.3, 114.7 (minor *E*-*anti*-conformer), 114.8 (major *E*-*anti*-conformer), 115.3 (d, *J* = 21.6 Hz, (minor *E*-*anti*-conformer)), 116.2 (d, *J* = 21.6 Hz, (major *E*-*anti*-conformer)), 123.4 (d, *J* = 15.6 Hz), 124.1 (d, *J* = 3.2 Hz, (minor *E*-*anti*-conformer)), 124.6 (d, *J* = 3.2 Hz, (major *E*-*anti*-conformer)), 126.7, 128.2 (minor *E*-*anti*-conformer), 128.8 (major *E*-*anti*-conformer), 129.5 (d, *J* = 2.6 Hz, (minor *E*-*anti*-conformer)), 130.1 (d, *J* = 2.6 Hz, (major *E*-*anti*-conformer)), 131.8 (d, *J* = 8.3 Hz, (minor *E*-*anti*-conformer)), 132.6 (d, *J* = 8.3 Hz, (major *E*-*anti*-conformer)), 143.8 (minor *E*-*anti*-conformer), 147.8 (major *E*-*anti*-conformer), 158.8 (d, *J* = 4.0 Hz), 160.2, 163.5 (d, *J* = 201 Hz) ppm.

^{19}F NMR (470 MHz, $(CD_3)_2SO$): δ –112.40 (s, 1F, (minor *E*-*anti*-conformer)), –113.64 (s, 1F, (major *E*-*anti*-conformer)) ppm.

HRMS (ES$^+$) calculated *m/z* for $C_{17}H_{18}FN_2O_3$ $[M+Na]^+$ 339.1115, found 339.1104

3. Synthesis and characterisation of 'nucleophilic' 4-fluorobenzylidene terminated nanoparticles (AuNP-1)

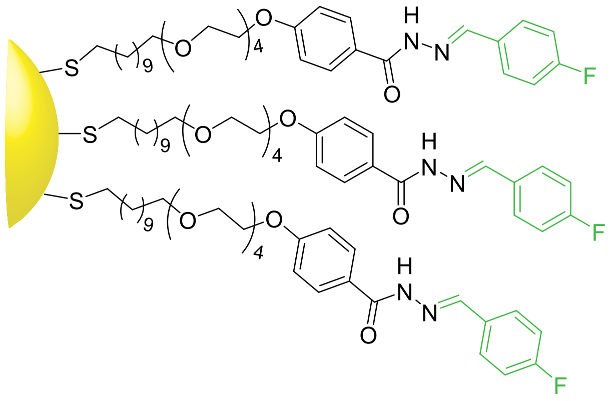

Synthesis

AuNP-**1** were synthesised following an adapted protocol to that previously described.[3] Disulfide $\mathbf{1}_2$ (0.30 g, 0.24 mmol) was dissolved in 8:1 *v*/*v* THF/DMF (30 mL) and the mixture was heated at 50 °C. After 5 min, PPh_3AuCl (0.24 g, 0.48 mmol) was added to the reaction mixture. Finally, *tert*-butylamine borane complex (0.42 g, 4.8 mmol) was added and the solution stirred at 50 °C for a further 5 min. The mixture was then left to cool at room temperature and stirred for 6 h. Nanoparticle precipitation was achieved by addition of 15:2:1 *v*/*v*/*v* Et_2O/EtOH/H_2O (54 mL), followed by sonication (10 min, 20 °C), and centrifugation (1935×g rcf, 10 min, 4 °C). The colourless supernatant was carefully discharged, then the black solid obtained was washed using the following procedure: nanoparticles were dispersed in 3:1:0.1 *v*/*v*/*v* Et_2O/EtOH/CH_2Cl_2 (10 mL), sonicated (15 min, 20 °C), and recollected by centrifugation (1935×g rcf, 10 min, 4 °C). The same operation was repeated for 3 times. At this stage, no unbound molecular species were detected in the supernatant by TLC or NMR analysis. Traces of volatile solvents were removed from the purified residue under a stream of compressed air, 1 mL water added, and the sample freeze dried to provide AuNP-**1**. Obtained 29 mg. Mean nanoparticle diameters produced by this procedure were found to be in the range of 2.8 – 3.4 nm (see below).

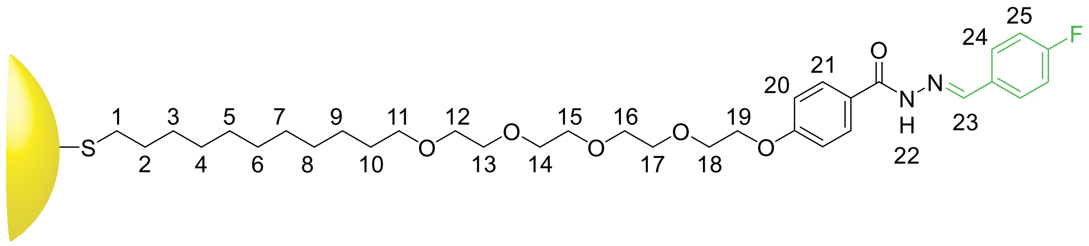

^{1}H NMR (500 MHz, [D$_7$]DMF): δ 1.41 (bs, 18H, 2–10), 3.48 (bs, 16H, 11–18), 4.15 (bs, 2H, 19), 7.12 (m, 4H, 20 and 25), 7.78 (bs, 2H, 24), 8.52 (bs, 1H, 22), 11.84 (bs, 1H, 23) ppm.

^{19}F NMR (470 MHz, [D$_7$]DMF): δ –111.49 (s, 1F) ppm.

Electron microscopy analysis

The inherently heterogeneous nature of all nanoparticle samples means that each batch exhibits small differences in nanoparticulate characteristics. TEM analysis performed on four independent batches of AuNP-**1** demonstrated excellent batch-to-batch consistency for nanoparticle mean diameter, size distribution and dispersity (**Figure S1**). The optimised synthetic procedure developed for producing ‘nucleophilic’ AuNP-**1**, provides nanoparticles with reproducible size (mean diameter: 2.8 – 3.4 nm) and low dispersity (< 20%). Furthermore, each repetition of the synthetic procedure yielded identical monolayer characterization data (see below).

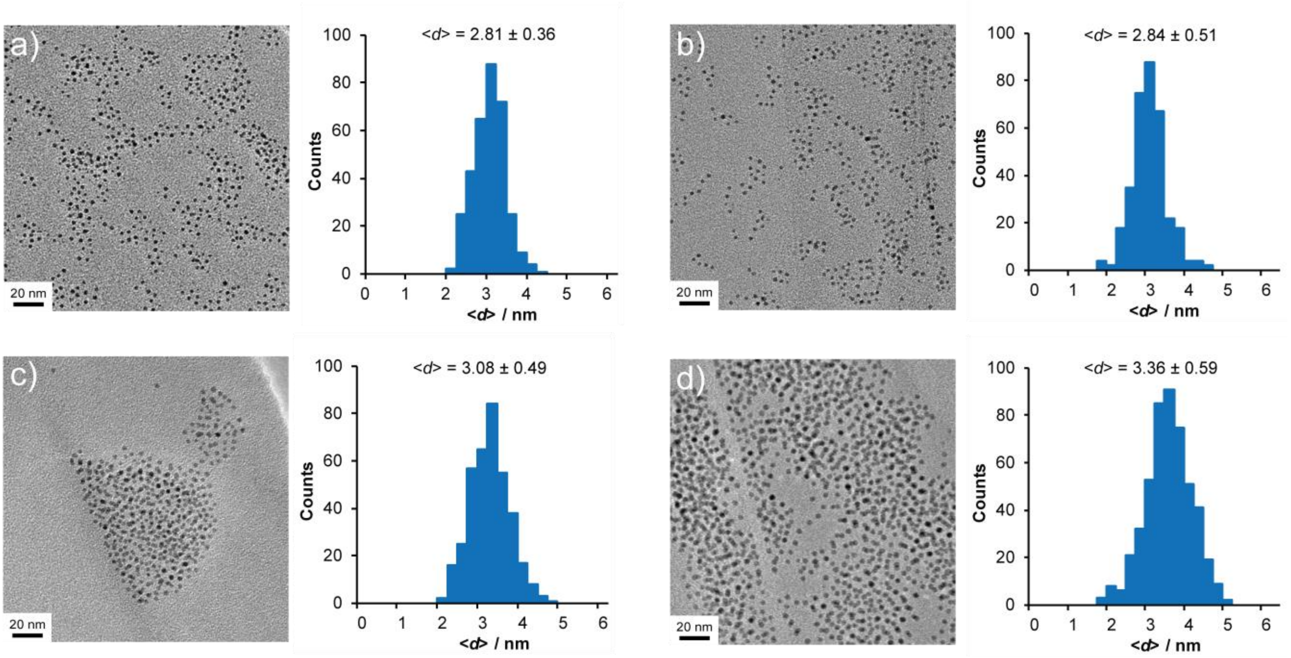

Figure S1. Representative TEM micrographs (scale bar 20 nm) for four independent batches of AuNP-**1** synthesised following the protocol described above. Size distribution (determined for a minimum of 200 particle measurements): a) <*d*> = 2.81 ± 0.36 nm (16% dispersity); b) <*d*> = 2.84 ± 0.51 nm (18% dispersity); c) <*d*> = 3.08 ± 0.49 nm (18% dispersity); d) <*d*> = 3.36 ± 0.59 nm (18% dispersity).

Thermal gravimetric analysis

Thermal gravimetric analysis (TGA) and differential thermal analysis (DTA) were performed by heating AuNP-**1** powder, under a stream of air, from 20 – 900 °C. Thermal decomposition of AuNP-**1** surface-bound monolayer resulted in progressive mass loss as temperature increased above the onset melting temperature (T_m) at 291 °C. Assuming an isotropic gold core (d = 3.08 nm) the organic mass lost between 291 – 900 °C was used to estimate molar weight and number of ligands per nanoparticle (**Figure S2** and **Table S1**). The small increase in mass observed at low temperatures in all TGA measurements can be ascribed to buoyancy effects that result from the changing air density on heating.[4] This can be caused by the relatively high air-flow rate (22 mL min^{-1}), which was necessary to ensure a sharp mass loss transition. In all experiments here, the mass increase observed is never more than 2.5 mass% and so within the experimental error of the measurement.

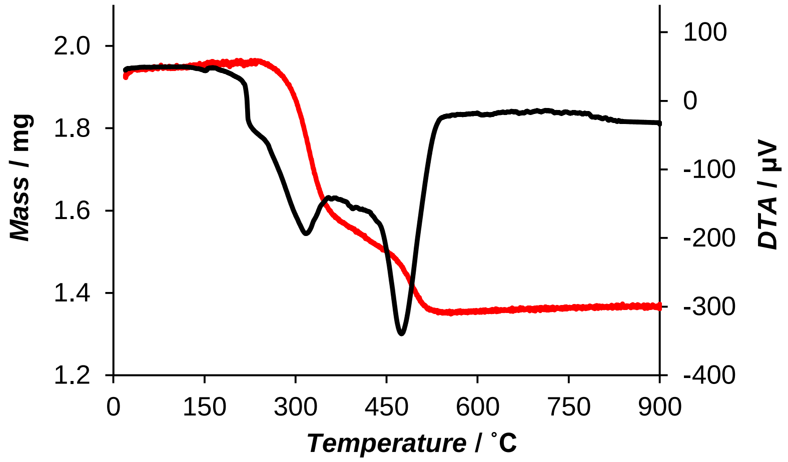

Figure S2. TGA (red curve) and DTA (black curve) plots for AuNP-**1** (1.92 mg). The sample was heated under air at a ramp rate of 10 °C min^{-1} over the range 20–900 °C.

Table S1. AuNP-**1** structural characterisation data calculated from TGA, DTA and TEM.

<*d*> / nm	AuNP-**1** surface area / nm^2	Au %	Surface-bound **1** %	Ligand **1** surface area / nm^2	AuNP-**1** M.W. / g mol^{-1}	Molecules **1** per nanoparticle
3.08	29.8	70.9	29.1	0.253	2.51 x 10^5	118

In situ NMR Characterization of AuNP-1

Solution-state NMR spectroscopy was employed to investigate the molecular composition of AuNP-**1** surface-bound monolayer (**Figure S3**).

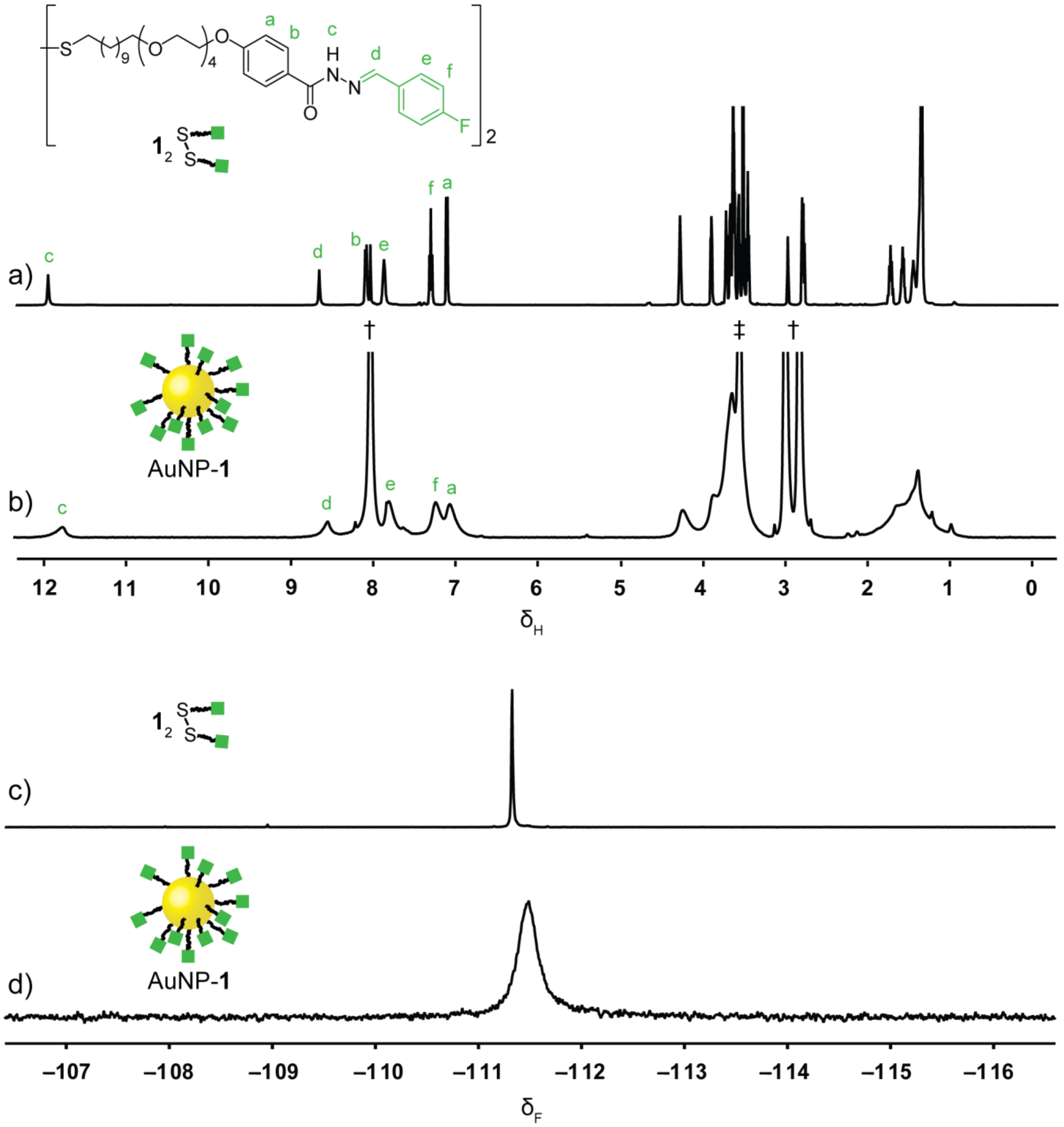

Figure S3. Full sweep-width NMR characterization of AuNP-**1**: a) ^{1}H NMR (500 MHz, [D$_7$]DMF, rt) spectrum of disulfide **1**$_2$; b) ^{1}H NMR (500 MHz, [D$_7$]DMF, rt) spectrum of AuNP-**1**. All sharp signals can be assigned to residual non-deuterated solvents as indicated († = DMF, ‡ = H_2O); c) ^{19}F NMR (470 MHz, [D$_7$]DMF, rt) spectrum of disulfide **1**$_2$; d) ^{19}F NMR (470 MHz, [D$_7$]DMF, rt) spectrum of AuNP-**1**.

Purity from non-solvent unbound contaminants was confirmed by T_2-filtered NMR analysis. The CPMG-z pulse sequence abolishes all signals produced by molecules with relaxation times shorter than a set value, such as those corresponding to nanoparticle-bound molecules, while resonances produced by molecular species in bulk solution remain visible.[5] An appropriate delay time was determined by empirical optimisation with an upper limit of the longest delay for which signals of all potential molecular contaminants are still visible. Setting D21 at 0.4 s, produces a T_2-filtered spectrum of molecule **1**$_2$ that is essentially identical to that obtained from a regular ^{1}H pulse sequence (**Figure S4 a** and **b**). In contrast, the T_2-filtered spectrum of AuNP-**1** only exhibited signals of residual non-deuterated solvent and water, confirming complete removal of unbound species from the nanoparticle sample (**Figure S4 d**).

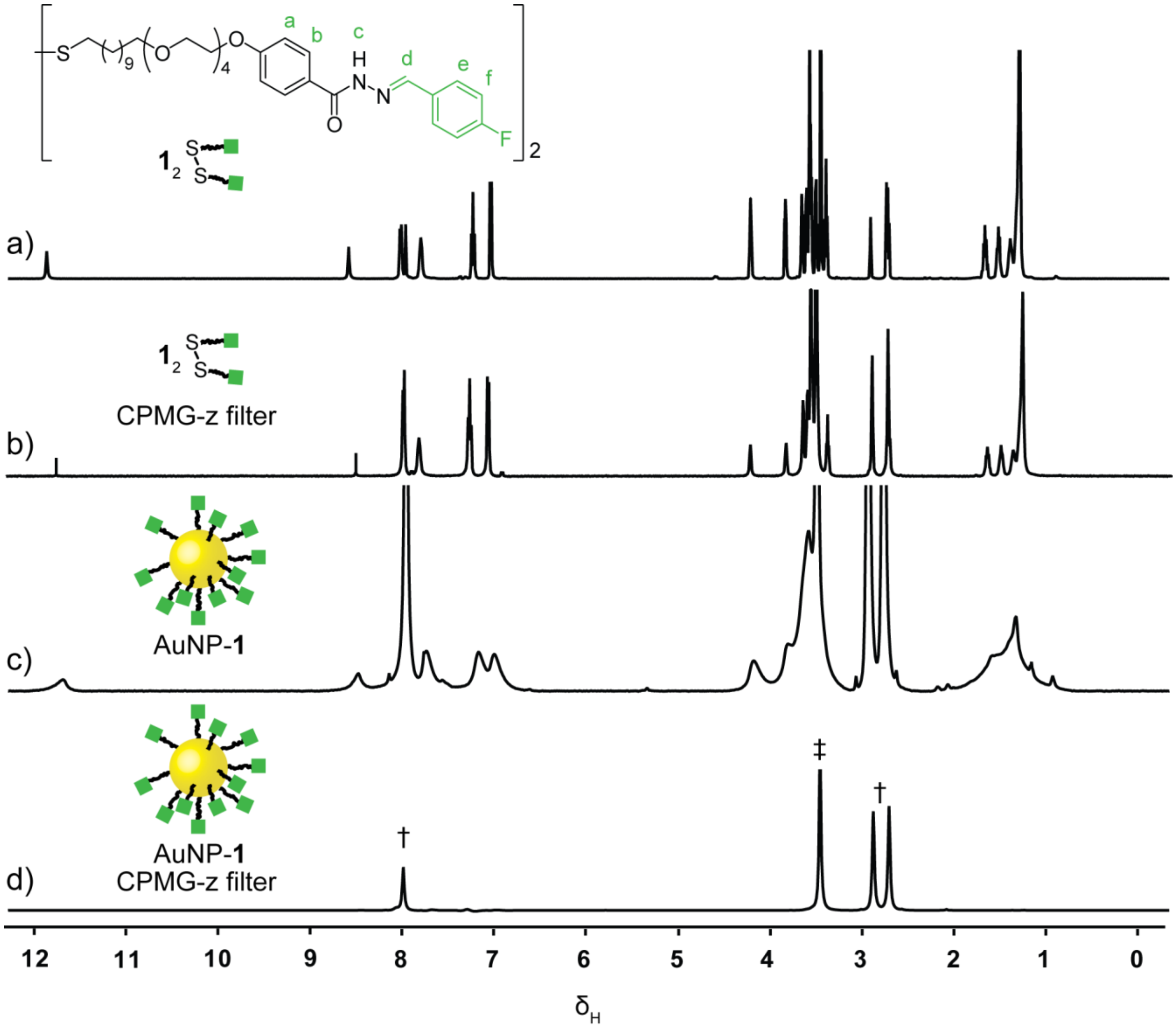

Figure S4. ^{1}H NMR Characterization of AuNP-**1** purity by relaxation time filtered spectra (500 MHz, [D$_7$]DMF, rt): a) ^{1}H NMR spectrum of disulfide **1**$_2$; b)T_2-filtered ^{1}H NMR spectrum of disulfide **1**$_2$ acquired using the CPMG-z pulse sequence,[5] D21 = 0.4 s; c) ^{1}H NMR spectrum of AuNP-**1**; d) T_2-Filtered ^{1}H NMR spectrum of AuNP-**1** acquired using the CPMG-z pulse sequence,[5] D21 = 0.4 s. All sharp signals can be assigned to residual non-deuterated solvents as indicated († = DMF, ‡ = H_2O).

Ex situ NMR Characterization of AuNP-1: oxidative ligand desorption

Ligand desorption using a mild oxidising agent such as iodine allows analysis of the released molecular species in bulk solution. Recently we have demonstrated how this approach can be successfully employed for quantitative investigation of integrity and composition for hydrazone-functionalised nanoparticle-bound monolayers.[3, 6] Oxidative ligand desorption performed on AuNP-**1** was followed by ^{19}F NMR. In the ^{19}F NMR spectra, all fluorinated species could be assigned, and concentrations quantified relative to an internal standard.

A colloidally stable solution of AuNP-**1** (5.6 mg) containing 4-fluorotoluene as internal standard (5.00 mM) was prepared in 9:1 *v*/*v* DMF/D_2O (600 µL) giving 4.93 mM in terms of surface-bound hydrazone **1** (**Figure S5d**). The solution was then treated with iodine (2 mg) and incubated at rt for 4.2 h. The first spectrum recorded 54 minutes after addition of the oxidising agent revealed three sharp peaks (**Figure S5e**). The characteristic broad nanoparticle signal at –110.50 ppm had disappeared, indicating that there were no longer molecules bound to the nanoparticle surface. The signal at –110.77 ppm could be assigned to disulfide $\mathbf{1}_2$, while the resonance appearing at –103.51 ppm was assigned as aldehyde **10**. This species, which results from hydrazone hydrolysis in presence of iodine, undergoes further decomposition generating the corresponding 4-fluorobenzoic acid (–107.49 ppm). The series of spectra recorded at later times showed a progressive decrease in concentration of disufide $\mathbf{1}_2$ and corresponding increase in concentration of aldehyde **10**, while the amount of 4-fluorobenzoic acid produced remained constant (**Figure S5e–h**). Pleasingly, during the entire course of the experiment, the total concentration of fluorinated species measured in bulk solution remained constant and always equal to the total amount of surface-bound ligands estimated before iodine addition (within experimental error, **Table S2**). The absence of any further peaks confirmed the integrity of the hydrazone moiety was maintained during nanoparticle direct synthesis. To verify that both aldehyde **10** and 4-fluorobenzoic acid were generated in situ upon addition of iodine, the behaviour of disulfide $\mathbf{1}_2$ was investigated under the same conditions, revealing the same pattern of peaks as observed during oxidative desorption from AuNP-**1** (**Figure S6, Table S3**).

Table S2. Concentrations of fluorine-containing species during oxidative ligand desorption from AuNP-**1**.

Spectrum **Figure S5**	*t* / h	[AuNP-**1**] / mM	[$\mathbf{1}_2$] / mM	[**10**] / mM[a]	Overall conc./ mM
d)	0	4.15			4.15
e)	0.9		0.14	4.07	4.21
f)	2.15			4.18	4.18
g)	3.17			4.17	4.17
h)	4.18			4.19	4.19

[a] Including the concentration of 4-fluorobenzoic acid.

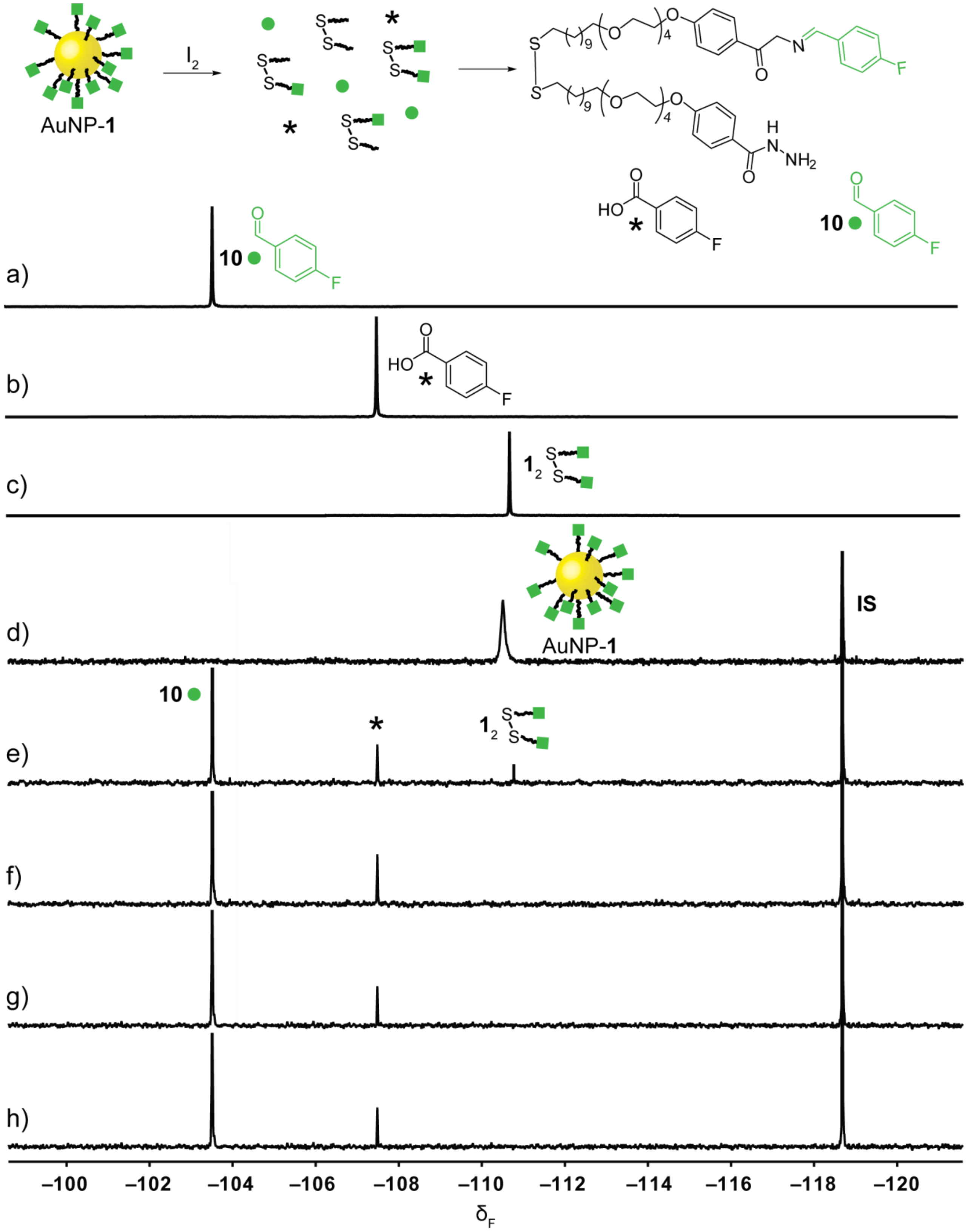

Figure S5. Oxidative ligand desorption performed on a solution of purified AuNP-**1**: a) ^{19}F NMR (470 MHz, 9:1 *v*/*v* DMF/D_2O) spectrum of aldehyde **10**; b) ^{19}F NMR (470 MHz, 9:1 *v*/*v* DMF/D_2O) spectrum of 4-fluorobenzoic acid; c) ^{19}F NMR (470 MHz, 9:1 *v*/*v* DMF/D_2O) spectrum of disulfide $\mathbf{1}_2$; d) ^{19}F NMR (470 MHz, 9:1 *v*/*v* DMF/D_2O, D1: 25 s) spectrum of AuNP-**1**. Nanoparticle-bound [**1**] = 4.15 mM (internal standard 5 mM 4-fluorotoluene, δ_F –118.68 ppm); e–h) ^{19}F NMR (470 MHz, 9:1 *v*/*v* DMF/D_2O, D1: 25 s) spectra recorded 54, 129, 190 and 251 min after addition of iodine to a solution of AuNP-**1**. Total concentration of all fluorinated species remained constant during the experiment time-course and in close agreement with on-nanoparticle concentration measured at t = 0 min (**Table S2**).

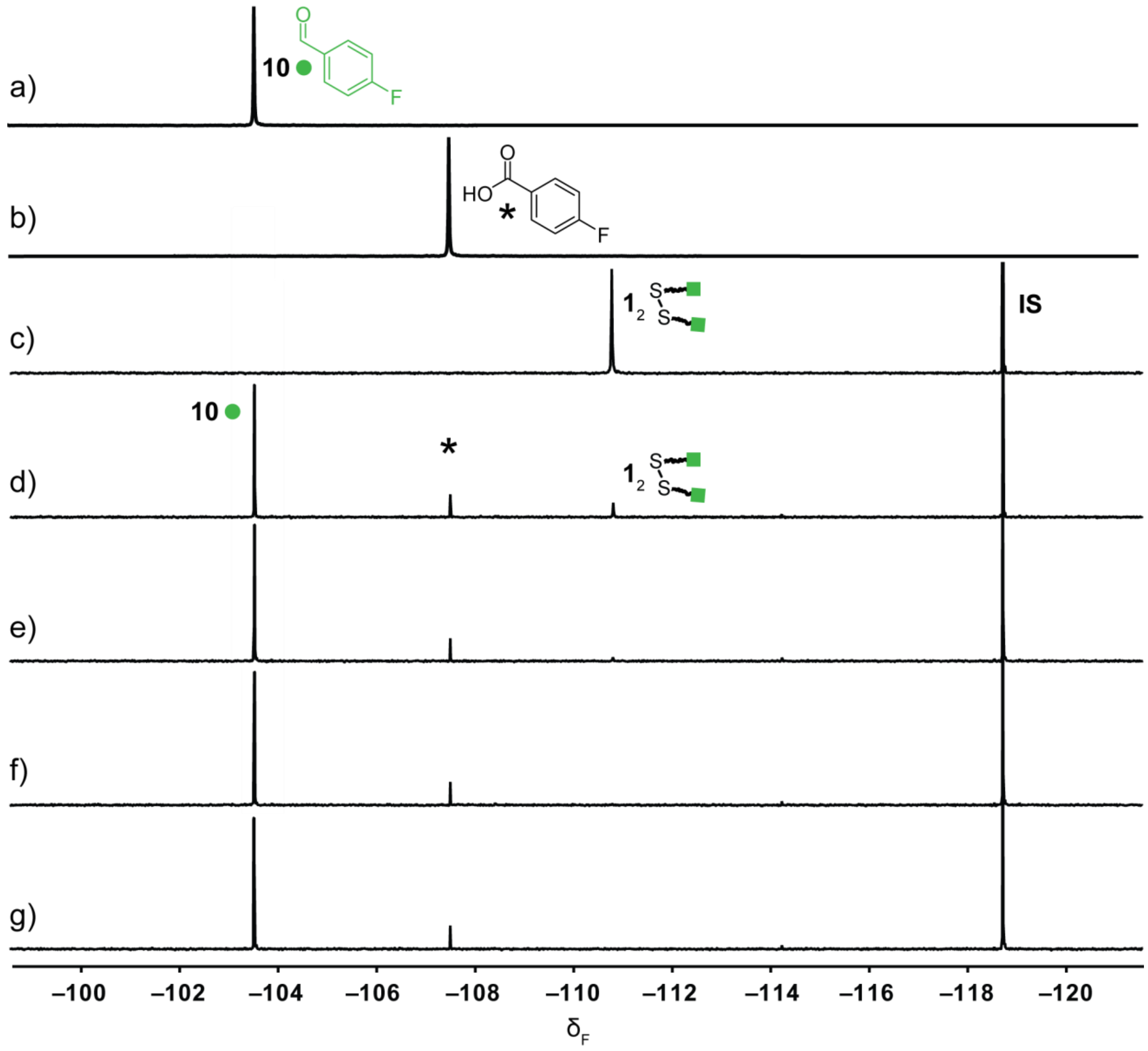

Figure S6. Solution of disulfide **1**$_2$ incubated with iodine. a) ^{19}F NMR (470 MHz, 9:1 *v*/*v* DMF/D$_2$O) spectrum of aldehyde **10**; b) ^{19}F NMR (470 MHz, 9:1 *v*/*v* DMF/D$_2$O) spectrum of 4-fluorobenzoic acid; c) ^{19}F NMR (470 MHz, 9:1 *v*/*v* DMF/D$_2$O, D1: 25 s) spectrum of disulfide **1**$_2$. [**1**$_2$] =4.18 mM (internal standard 5 mM 4-fluorotoluene, δ_F –118.68 ppm); d–g) ^{19}F NMR (470 MHz, 9:1 *v*/*v* DMF/D$_2$O, D1: 25 s) spectra recorded 55, 103, 185 and 343 min after addition of iodine to a solution of disulfide **1**$_2$. Total concentration of all fluorinated species remained constant during the experiment time-course and in close agreement with the concentration of disulfide **1**$_2$ measured at t = 0 min (**Table S3**).

Table S3. Concentrations of fluorine-containing species during incubation of disulfide **1**$_2$ with iodine

Spectrum **Figure S6**	*t* / h	[**1**$_2$] / mM	[**10**] / mM[a]	Overall conc./ mM
c)	0	4.18		4.18
d)	0.92	0.60	3.61	4.22
e)	1.72	0.11	4.05	4.16
f)	3.08		4.20	4.20
g)	5.72		4.22	4.22

[a] Including the concentration of 4-fluorobenzoic acid.

4. Synthesis and characterisation of 'electrophilic' 4-fluorobenzoylhydrazide terminated nanoparticles (AuNP-2)

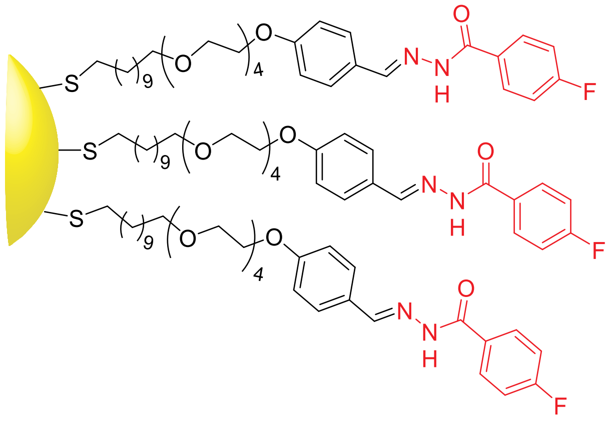

Synthesis

Disulfide **2**$_2$ (0.30 g, 0.24 mmol) was dissolved in 8:1 *v*/*v* THF/DMF (30 mL) and the mixture was heated at 50 °C. After 5 min, PPh_3AuCl (0.24 g, 0.48 mmol) was added to the reaction mixture. Finally, *tert*-butylamine borane complex (0.42 g, 4.84 mmol) was added and the solution stirred at 50 °C for a further 5 min. The mixture was then left to cool at room temperature and stirred for 6 h. After this time, the reaction was cooled to 0 °C. Nanoparticle precipitation was achieved by addition of 15:1:1 *v*/*v* Et_2O/EtOH/H_2O (54 mL), followed by sonication (10 min, 20 °C), and centrifugation (1935×g rcf, 10 min, 4 °C). The colourless supernatant was carefully discharged, then the black solid obtained was washed using the following procedure: nanoparticles were dispersed in 1:1 *v*/*v* Et_2O/EtOH (10 mL), sonicated (15 min, 20 °C), and recollected by centrifugation (1935×g rcf, 10 min, 4 °C). The same operation was repeated 3 times, progressively increasing the amount of EtOH used in the mixture (from 1:1 to 3:1). At this stage, no unbound molecular species were detected in the supernatant by TLC or NMR analysis. Traces of volatile solvents were removed from the purified residue under a stream of compressed air, 1 mL water added, and the sample freeze dried to provide AuNP-**2**. Obtained 30 mg. Mean nanoparticle diameters produced by this procedure were found to be in the range of 2.7 – 3.0 nm (see below).

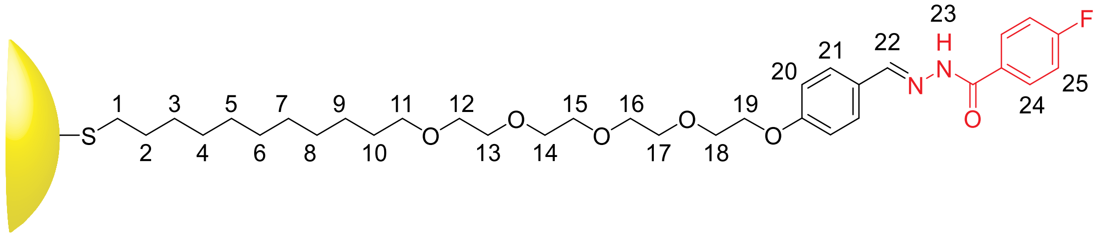

^{1}H NMR (500 MHz, [D$_7$]DMF): δ 1.52 (bs, 18H, 2–10), 3.55 (bs, 16H, 11–18), 4.20 (bs, 2H, 19), 6.99 (bs, 2H, 20), 7.47 (bs, 2H, 25), 7.75 (bs, 2H, 21), 8.58 (bs, 1H, 22), 11.84 (bs, 1H, 23) ppm.

^{19}F NMR (470 MHz, [D$_7$]DMF): δ –108.22 (s, 1F (major *E*-anti-conformer)), –108.77 (s, 1F (minor *E*-syn-conformer)) ppm.

Electron microscopy analysis

TEM analysis performed on four independent batches of AuNP-**2** demonstrated excellent batch-to-batch consistency for nanoparticle mean diameter, size distribution and dispersity (**Figure S7**). The optimised synthetic procedure developed for producing ‘electrophilic’ AuNP-**2**, provides nanoparticles with reproducible size (mean diameter: 2.7 – 3.0 nm) and low dispersity (< 20%). Each repetition of the synthetic procedure yielded identical monolayer characterization data (see below).

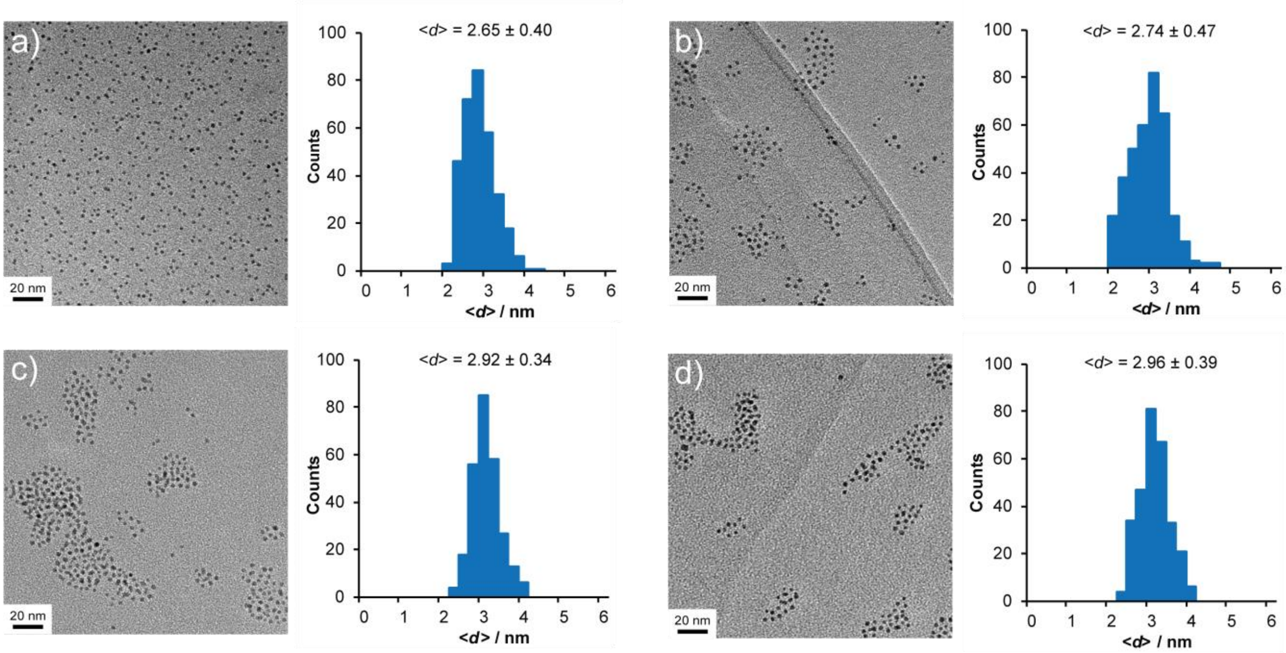

Figure S7. Representative TEM micrographs (scale bar 20 nm) for four independent batches of AuNP-**2** synthesised following the protocol described above. Size distribution (determined for a minimum of 200 particle measurements): a) <*d*> = 2.65 ± 0.40 nm (15% dispersity); b) <*d*> = 2.74 ± 0.47 nm (17% dispersity); c) <*d*> = 2.92 ± 0.34 nm (13% dispersity); d) <*d*> = 2.96 ± 0.39 nm (14% dispersity).

Thermal gravimetric analysis

Thermal gravimetric analysis (TGA) and differential thermal analysis (DTA) were performed on AuNP-**2** following the same procedure described above for AuNP-**1**. Thermal decomposition of AuNP-**2** surface-bound monolayer resulted in progressive mass loss as temperature increased above the onset melting temperature (T_m) at 290 °C. Assuming an isotropic gold core (d = 2.96 nm), the organic mass lost between 290–900 °C was used to estimate molar weight and number of ligands per nanoparticle (**Figure S8** and **Table S4**). The small increase in mass (< 2%) at low temperatures can be ascribed to the same effects as discussed above for AuNP-**1**.

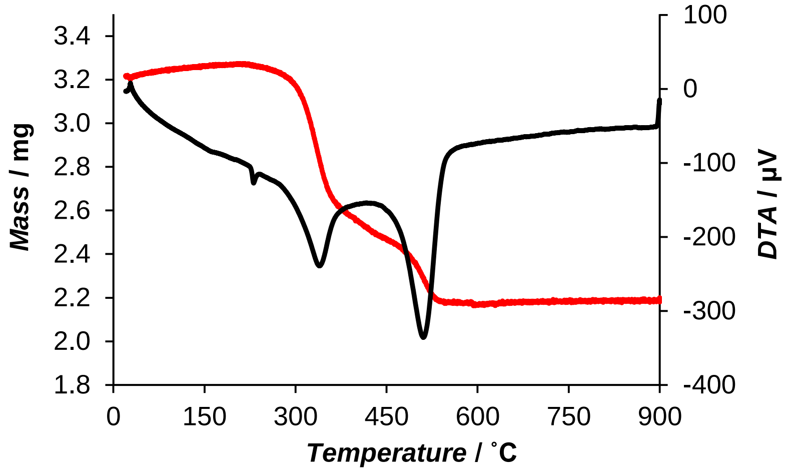

Figure S8. TGA (red curve) and DTA (black curve) plots for AuNP-**2** (3.20 mg). The sample was heated under air at a ramp rate of 10 °C min^{-1} over the range 20–900 °C.

Table S4. AuNP-**2** structural characterisation data calculated from TGA, DTA and TEM.

<*d*> / nm	AuNP-**2** surface area / nm^2	Au %	Surface-bound **2** %	Ligand **2** surface area / nm^2	AuNP-**2** M.W. / g mol^{-1}	Molecules **2** per nanoparticle
2.96	27.5	68.4	31.6	0.232	2.31 x 10^5	119

In situ NMR Characterization of AuNP-2

The molecular composition of AuNP-**2** surface-bound monolayer was investigated using solution-state ^{1}H and ^{19}F NMR spectroscopy (**Figure S9** and **Figure 2a, b**).

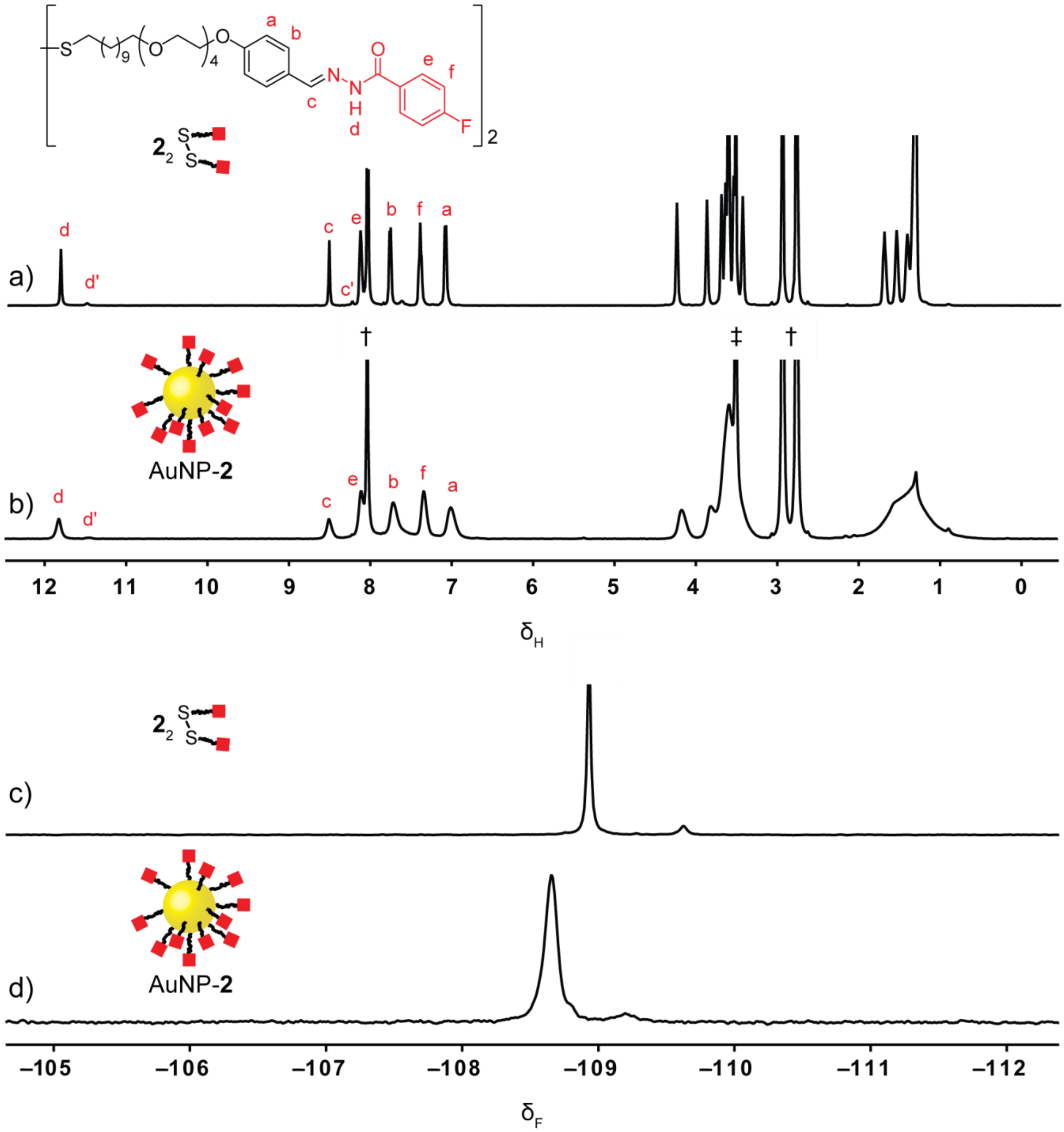

Figure S9. Full sweep-width NMR characterization of AuNP-**2**: a) ^{1}H NMR (500 MHz, [D$_7$]DMF) spectrum of disulfide **2**$_2$; b) ^{1}H NMR (500 MHz, [D$_7$]DMF) spectrum of AuNP-**2**. All sharp signals can be assigned to residual non-deuterated solvents as indicated († = DMF, ‡ = H_2O); c) ^{19}F NMR (470 MHz, [D$_7$]DMF) spectrum of disulfide **2**$_2$; d) ^{19}F NMR (470 MHz, [D$_7$]DMF) spectrum of AuNP-**2**.

Purity from non-solvent unbound contaminants was confirmed by T_2-filtered NMR analysis with a delay time set at 0.4 s (**Figure S10**), as described for AuNP-**1** above.

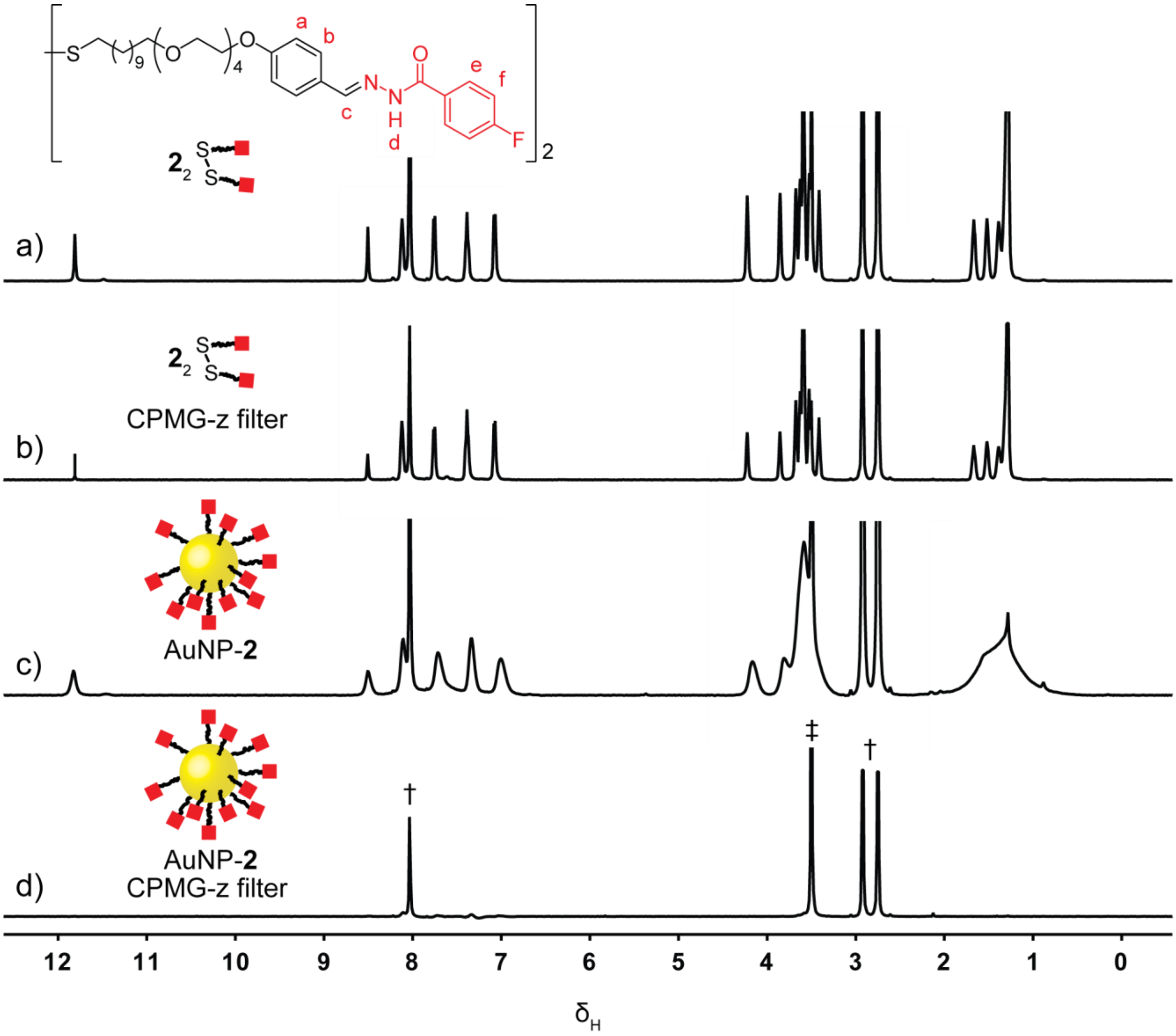

Figure S10. ^{1}H NMR Characterization of AuNP-**2** purity by relaxation time filtered spectra (500 MHz, [D7]DMF, rt): a) ^{1}H NMR spectrum of disulfide **2**$_2$; b) T_2-filtered ^{1}H NMR spectrum of disulfide **2**$_2$ acquired using the CPMG-z pulse sequence,[5] D21 = 0.4 s; c) ^{1}H NMR spectrum of AuNP-**2**; d) T_2-Filtered ^{1}H NMR spectrum of AuNP-**2** acquired using the CPMG-z pulse sequence,[5] D21 = 0.4 s. All sharp signals can be assigned to residual non-deuterated solvents as indicated († = DMF, ‡ = H_2O).

Ex situ NMR Characterization of AuNP-2: oxidative ligand desorption

AuNP-**2** was subjected to oxidative ligand desorption as described above for AuNP-**1**. The desorption process was followed by ^{19}F NMR (**Figure S11**, **Table S5**), and likewise the behaviour of disulfide $\mathbf{2}_2$ in the presence of iodine (**Figure S12**, **Table S6**), confirming the identity and compositional purity of the AuNP-**2** surface-bound ligand shell.

Table S5. Concentrations of fluorine-containing species during oxidative ligand desorption from AuNP-**2**.

Spectrum **Figure S11**	*t* / h	[AuNP-**2**] / mM	[$\mathbf{2}_2$] / mM	[**5**] / mM[a]	Overall conc./ mM
d)	0	3.27			3.27
e)	0.82		1.13	2.11	3.24
f)	1.58		0.40	2.89	3.29
g)	2.42		0.16	3.11	3.27
h)	4.53			3.28	3.28

[a] Including the concentration of 4-fluorobenzoic acid.

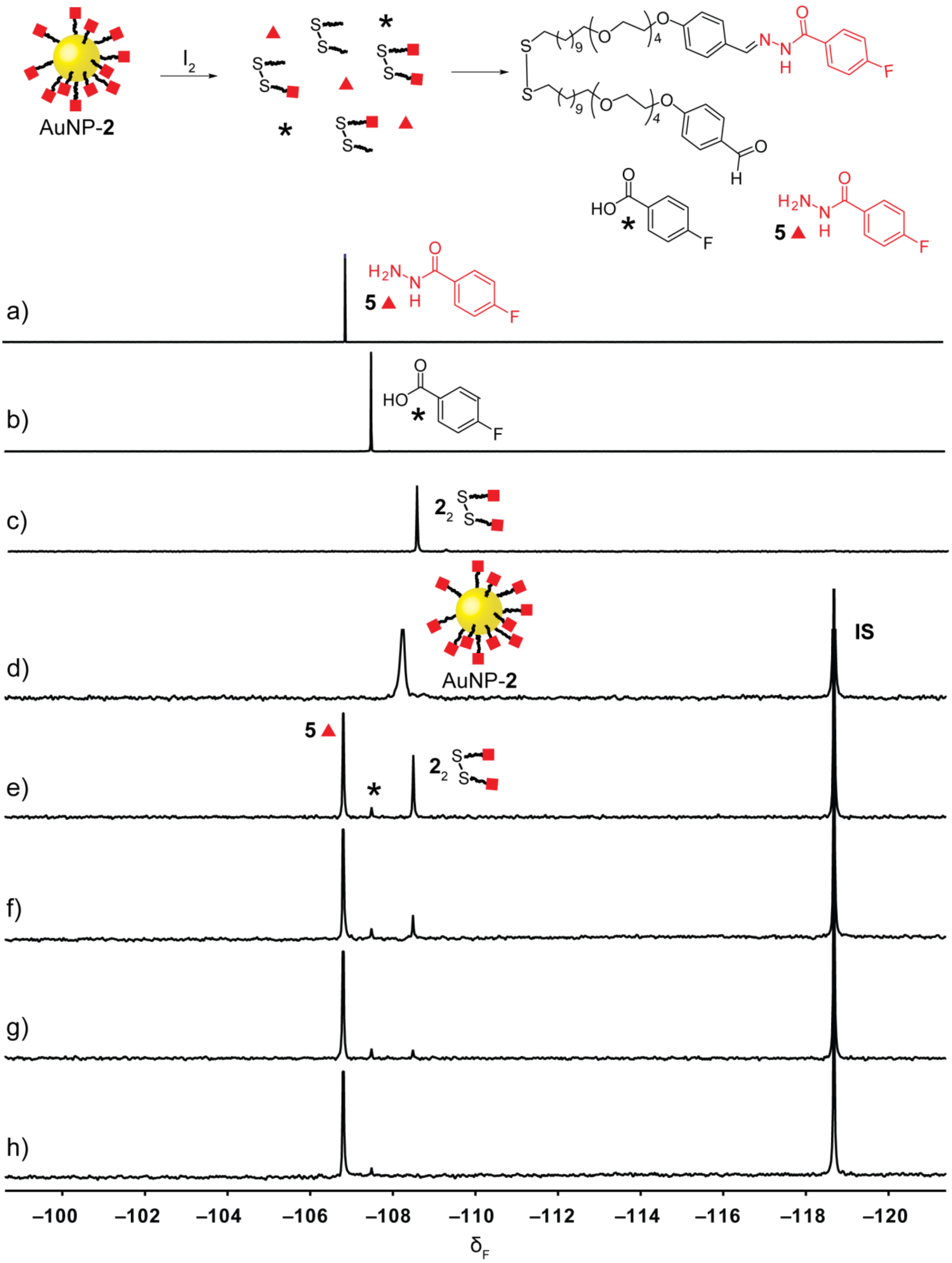

Figure S11. Oxidative ligand desorption performed on a solution of purified AuNP-**2**: a) ^{19}F NMR (470 MHz, 9:1 *v*/*v* DMF/D_2O) spectrum of hydrazide **5**; b) ^{19}F NMR (470 MHz, 9:1 *v*/*v* DMF/D_2O) spectrum of 4-fluorobenzoic acid; c) ^{19}F NMR (470 MHz, 9:1 *v*/*v* DMF/D_2O) spectrum of disulfide $\mathbf{2}_2$; d) ^{19}F NMR (470 MHz, 9:1 *v*/*v* DMF/D_2O, D1: 25 s) spectrum of AuNP-**2**. Nanoparticle-bound [**2**] = 3.27 mM (internal standard 5 mM 4-fluorotoluene, δ_F –118.68 ppm); e–h) ^{19}F NMR (470 MHz, 9:1 *v*/*v* DMF/D_2O, D1: 25 s) spectra recorded 49, 95, 145 and 272 min after addition of iodine to a solution of AuNP-**2.** Total concentration of all fluorinated species remained constant during the experiment time-course and in close agreement with on-nanoparticle concentration measured at t = 0 min (**Table S5**).

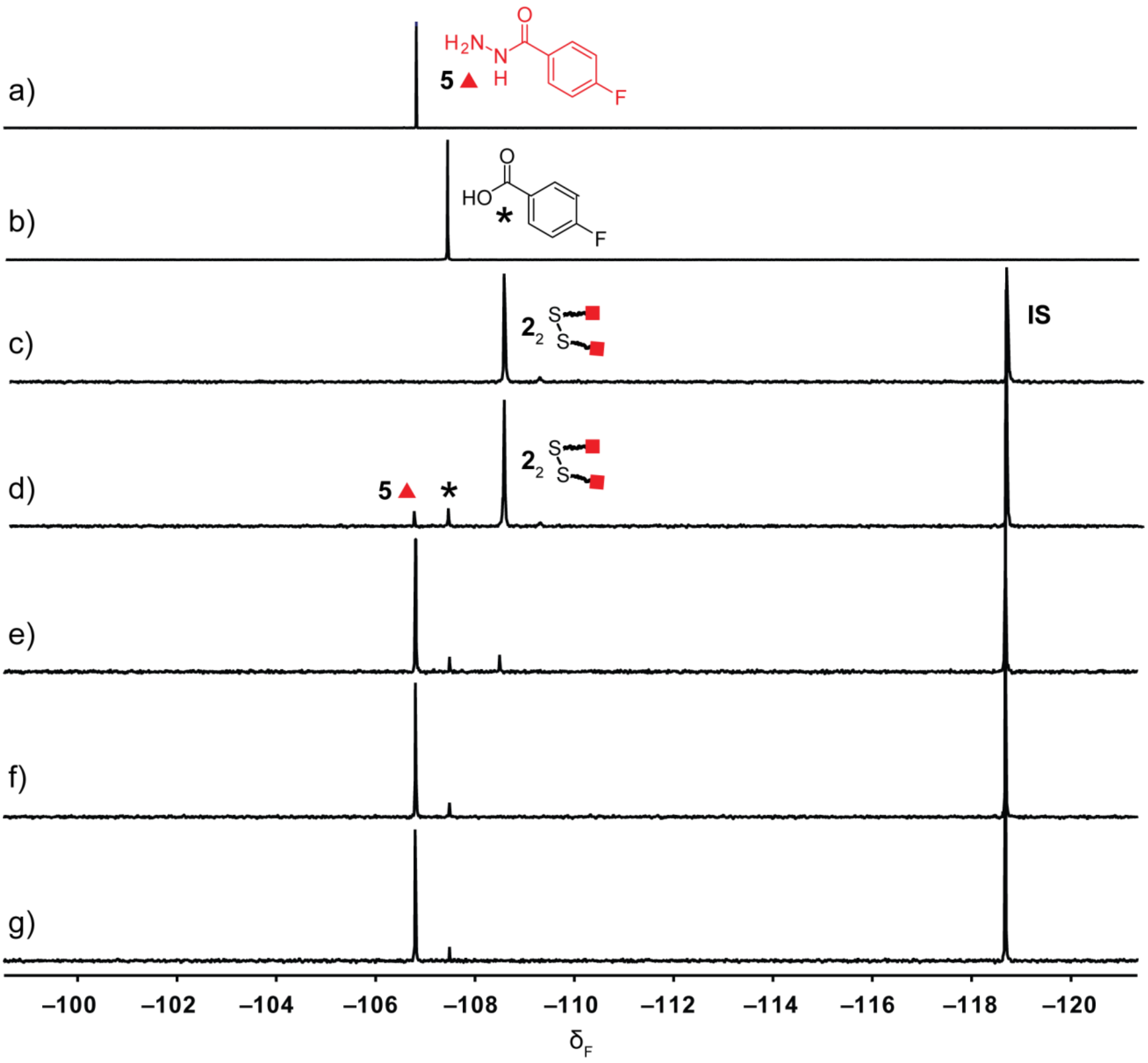

Figure S12. Solution of disulfide **2**$_2$ incubated with iodine. a) ^{19}F NMR (470 MHz, 9:1 *v*/*v* DMF/D_2O) spectrum of hydrazide **5**; b) ^{19}F NMR (470 MHz, 9:1 *v*/*v* DMF/D_2O) spectrum of 4-fluorobenzoic acid; c) ^{19}F NMR (470 MHz, 9:1 *v*/*v* DMF/D_2O, D1: 25 s) spectrum of disulfide **2**$_2$. [**2**$_2$] = 3.42 mM (internal standard 5 mM 4-fluorotoluene, δ_F –118.68 ppm); d–g) ^{19}F NMR (470 MHz, 9:1 *v*/*v* DMF/D_2O, D1: 25 s) spectra recorded 64, 132, 235 and 404 min after addition of iodine to a solution of disulfide **2**$_2$. Total concentration of all fluorinated species remained constant during the experiment time-course and in close agreement with the concentration of disulfide **2**$_2$ measured at t = 0 min (**Table S6**).

Table S6. Concentrations of fluorine-containing species during incubation of disulfide **2**$_2$ with iodine.

Spectrum **Figure S12**	*t* / h	[**2**$_2$] / mM	[**5**] / mM[a]	Overall conc./ mM
c)	0.00	3.42		3.42
d)	1.07	1.77	1.68	3.45
e)	2.20	0.82	2.60	3.42
f)	3.92		3.43	3.43
g)	6.73		3.45	3.45

[a] Including the concentration of 4-fluorobenzoic acid.

LDI-MS of AuNP-2

The laser desorption ionization of AuNP-**2** (performed in absence of an energy absorbing matrix) produced a mass spectrum revealing molecular ions corresponding to disulfide **2**$_2$ and thiol **2**H, along with characteristic fragmentation patterns identical to those previously observed for structurally similar nanoparticle-bound monolayers.[3, 7]

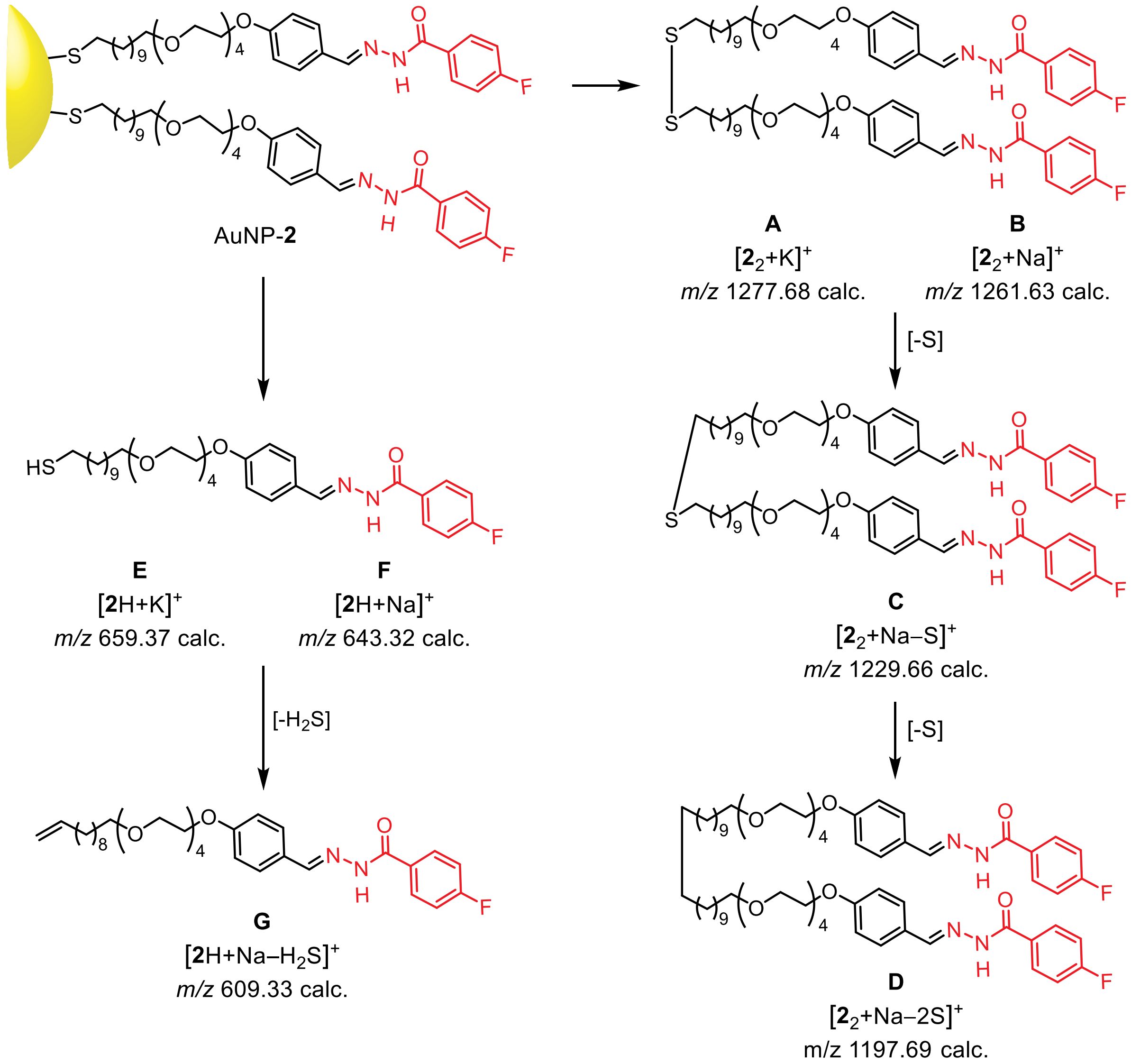

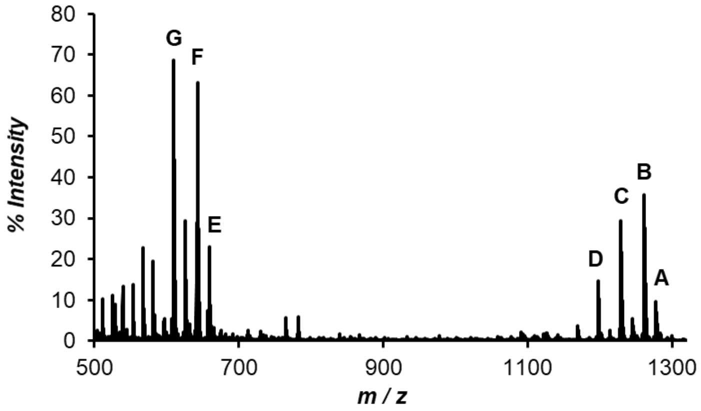

Figure S13. LDI mass spectrum and fragmentation pattern for AuNP-**2**. Molecular ion of disulfide **2**$_2$ (peak **A**, [**2**$_2$+K]$^+$: m/z 1277.68; peak **B**, [2$_2$+Na]$^+$: m/z 1261.63) undergoes a progressive loss of sulfur atoms (peak **C**, [**2**$_2$–S+Na]$^+$: m/z 1229.64; peak **D**, [2$_2$–2S+Na]$^+$: m/z 1197.67). In a similar manner, molecular ion corresponding to thiol **2**H (peak **E**, [**2**H+K]$^+$: m/z 659.37; peak **F**, [**2**H+Na]+: m/z 643.32) loses a H_2S fragment (peak **G**, [**2**H–H_2S+Na]$^+$: m/z 609.33).

5. Synthesis and characterisation of 'electrophilic' 2-fluorobenzoylhydrazide terminated nanoparticles (AuNP-3)

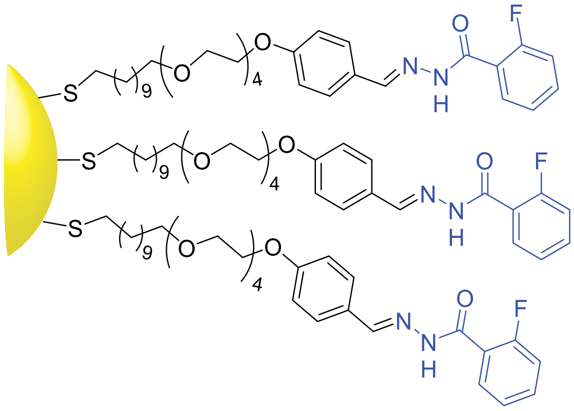

Synthesis

Disulfide **3**$_2$ (60.0 mg, 48.4 µmol) and PPh_3AuCl (47.9 mg, 96.8 µmol) were dissolved in 8:1 *v*/*v* THF/DMF (6 mL). Then, *tert*-butylamine borane complex (84.2 mg, 968 µmol) was added and the solution stirred for 6 h at rt. After this time, the reaction was cooled to 0 °C. Nanoparticle precipitation was achieved by addition of 8:2:1 *v*/*v* Et_2O/EtOH/MeCN (15 mL), followed by sonication (10 min, 20 °C), and centrifugation (1312 ×g rcf, 10 min, 4 °C). The colourless supernatant was carefully discharged, then the black solid obtained was washed using the following procedure: nanoparticles were dispersed in 1:1 *v*/*v* EtOH/Et_2O (10 mL), sonicated for 15 min, and centrifuged (1312 ×g rcf, 10 min, 4 °C). The operation was repeated a further 3 times, progressively increasing the amount of EtOH in the mixture (from 1:1 to 3:1 *v*/*v*). Traces of volatile solvents were removed from the purified residue under a stream of compressed air, 1 mL water added, and the sample freeze dried to provide AuNP-**3**. Obtained 3.02 mg. Mean diameter: 3.66 ± 0.62 nm (17% dispersity).

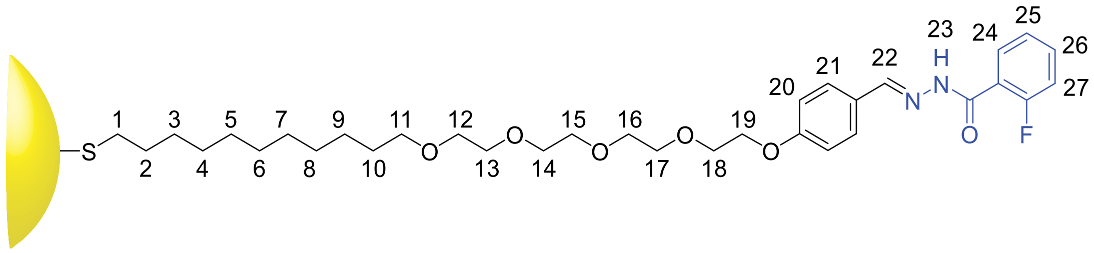

^{1}H NMR (500 MHz, [D$_7$]DMF): δ 1.48 (bs, 18H, 2–10), 3.57 (bs, 16H, 11–18), 4.19 (bs, 2H, 19), 6.85 (bs, 2H, 20), 7.49 (m, 6H, 21 and 24–27), 8.47 (bs, 1H, 22), 11.45 (bs, 1H, 23) ppm.

^{19}F NMR (470 MHz, [D$_7$]DMF): δ –111.93 (s, 1F (minor *E*-anti-conformer)), –113.25 (s, 1F (major *E*-anti-conformer)) ppm.

For full ^{1}H and ^{19}F spectral comparison of AuNP-**3** to AuNP-**3**(e) and disulfide precursor **3**$_2$, see **Figure S17**.

Electron microscopy analysis

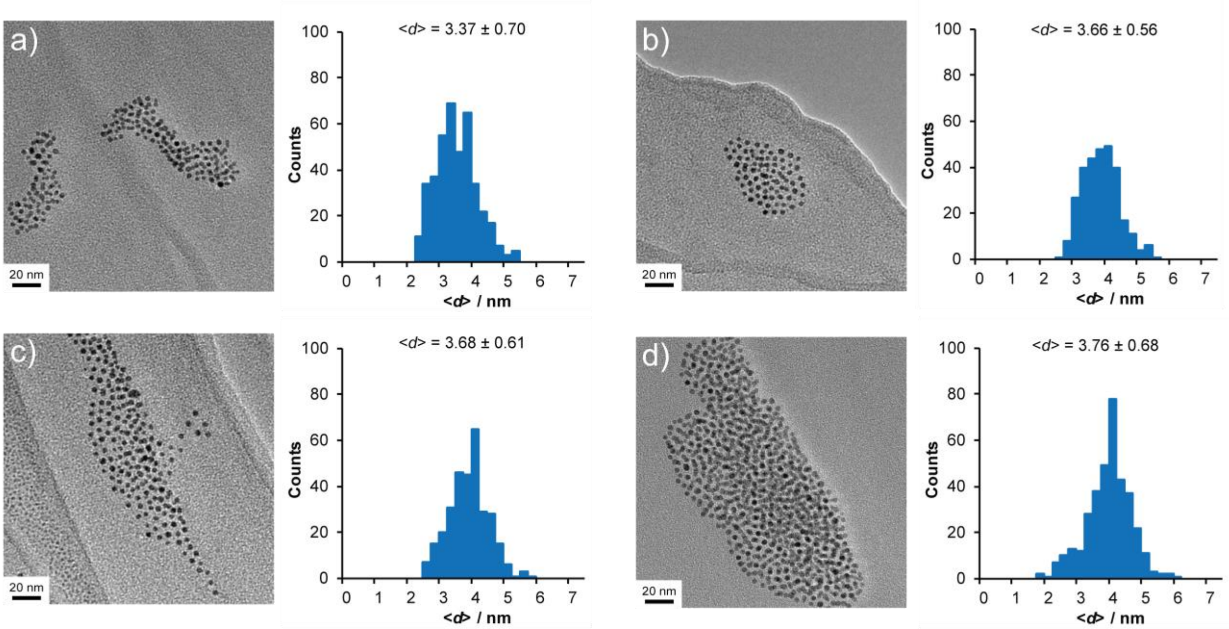

Figure S14. Representative TEM micrographs (scale bar 20 nm) for four independent batches of AuNP-**3** synthesised following the protocol described above. Size distribution (determined for a minimum of 200 particle measurements): a) <*d*> = 3.37 ± 0.70 nm (21% dispersity); b) <*d*> = 3.66 ± 0.56 nm (15% dispersity); c) <*d*> = 3.68 ± 0.61 nm (17% dispersity); d) <*d*> = 3.76 ± 0.68 nm (18% dispersity).

6. Dynamic covalent modification of 'electrophilic' nanoparticles with nucleophilic modifiers

6.1 Generation and characterization of AuNP-3(e) and mixed monolayer compositions AuNP-$\mathbf{2_x3_y}$

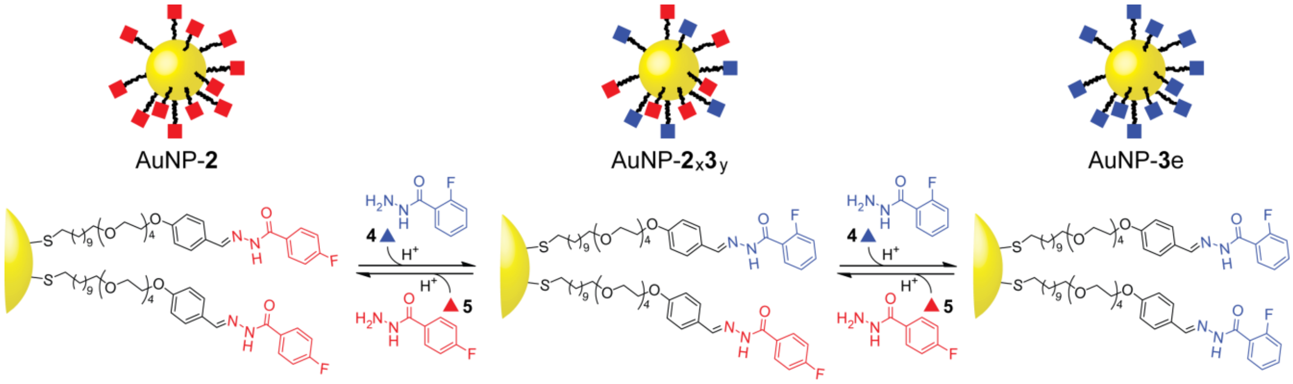

Concentrations of all fluorine-containing species were determined by quantitative ^{19}F NMR in the presence of 4-fluorotoluene as an internal standard of known concentration.

A stock solution of CF_3CO_2H was prepared in 9:1 *v*/*v* DMF/D_2O with 4-fluorotoluene as internal standard (5.00 mM) and concentration measured by ^{19}F NMR.

Generation of AuNP-$\mathbf{2_{0.4}3_{0.6}}$

A colloidal solution of AuNP-**2** (6.81 mg) containing 4-fluorotoluene (5.00 mM) was prepared in 9:1 *v*/*v* DMF/D_2O (700 μL) giving 4.93 mM in terms of surface-bound hydrazone **2**. Then, an equimolar quantity of hydrazide **4** (0.53 mg, 3.46 μmol) was added, followed by an aliquot of the CF_3CO_2H stock solution (16.3 μL, 13.4 μmol), giving final concentrations of AuNP-**2** (4.82 mM), **4** (4.83 mM, 1.0 Eq.), and CF_3CO_2H (20.5 mM). This mixture was incubated at rt and the reaction was followed by ^{19}F NMR. A new sharp signal appeared at –106.86 ppm, corresponding to hydrazide **5** released from the nanoparticle-bound monolayer, together with a new set of broad nanoparticle-bound signals (**Figure S15b**). No further changes were observed after 1 h. Nanoparticles were precipitated by adding 8:1 v/v Et_2O/EtOH (10 mL). The black solid recovered was resuspended in 7:1 *v*/*v* Et_2O/EtOH (8 mL), sonicated for 10 min and recollected by centrifugation (1312 ×g rcf, 4 °C, 20 min). This operation was repeated a further 2 times. Traces of volatile solvents were removed from the purified residue under a stream of compressed air, 1 mL water added, and the sample freeze dried to provide AuNP-$\mathbf{2}_{0.4}\mathbf{3}_{0.6}$ free from unbound species. Pleasingly, the ratio for the two surface-bound hydrazones calculated by area deconvolution of either the sharp signals for hydrazides in bulk solution and broad nanoparticle signals before nanoparticle purification (**Figure S15b**), agree with the monolayer composition assessed after nanoparticle purification (**Figure S16b** and **Table S7**).

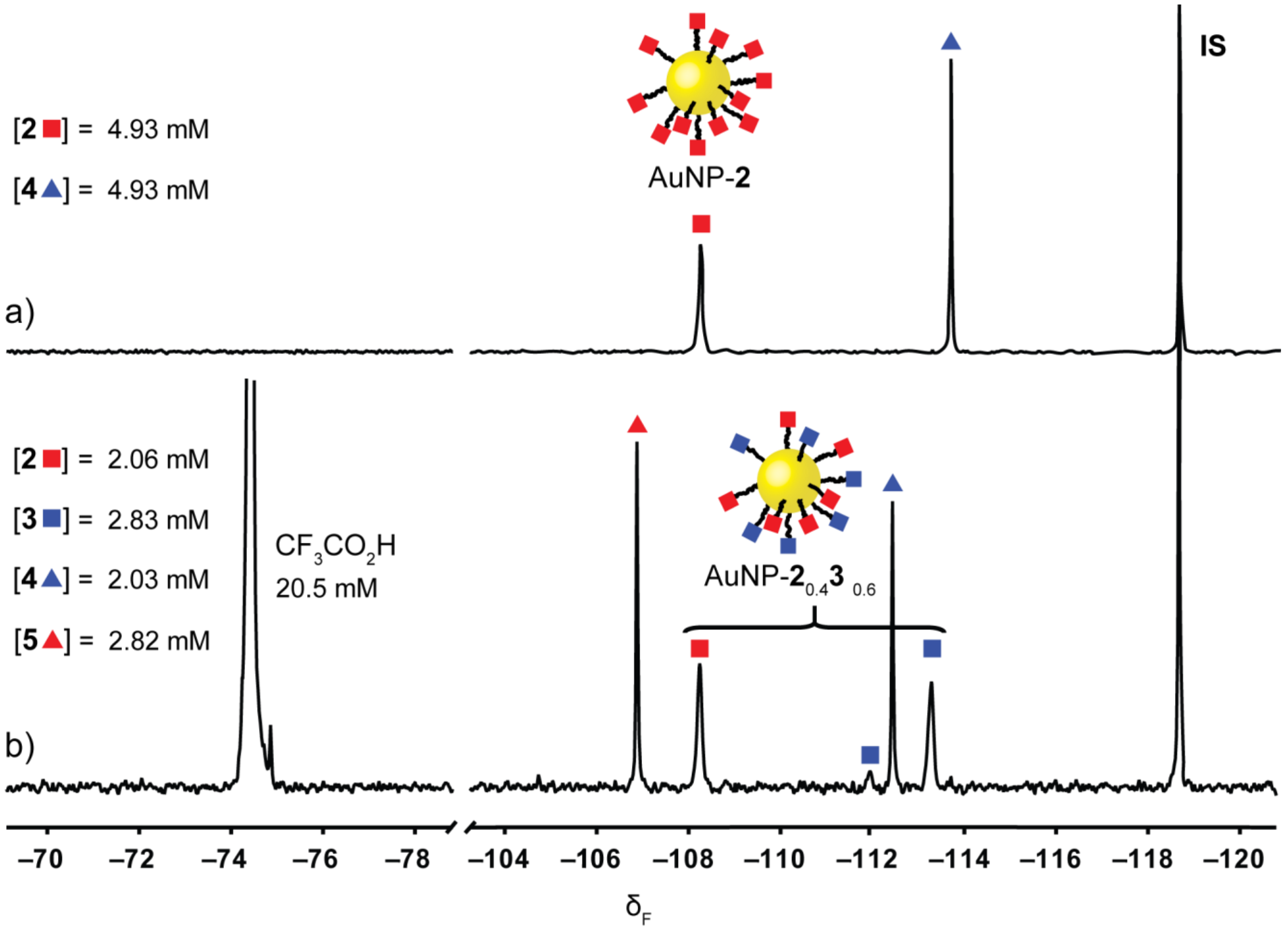

Figure S15. In situ monitoring of dynamic covalent exchange from AuNP-**2** to AuNP-$\mathbf{2}_{0.4}\mathbf{3}_{0.6}$ by ^{19}F NMR. (470 MHz, 9:1 *v*/*v* DMF/D_2O, D1: 25 s): a) AuNP-**2** (4.93 mM) and hydrazide **3** (4.93 mM); b) Reaction mixture incubated at rt for 56 min after addition of CF_3CO_2H (20.5 mM), confirming a 2:3 ratio of **2** and **3** surface-bound hydrazones. IS: internal standard (4-fluorotoluene, 5.00 mM). The change in chemical shift of hydrazide **4** is brought about by protonation in the presence of CF_3CO_2H. Two signals are assigned to surface-bound hydrazone **3** corresponding to two hydrazone rotamers.

Generation of AuNP-$\mathbf{2}_{0.1}\mathbf{3}_{0.9}$

To drive the exchange towards complete replacement of the starting hydrazone, a solution containing AuNP-**2** (6.21 mg) and 4-fluorotoluene (5.00 mM) was prepared in 9:1 *v*/*v* DMF/D_2O (620 μL) giving 5.04 mM in terms of surface-bound hydrazone **2**. To this hydrazide **4** was added (2.51 mg, 16.3 μmol), followed by an aliquot of the CF_3CO_2H stock solution (10 μL, 12.4 μmol), giving final concentrations of AuNP-**2** (4.96 mM), **4** (25.9 mM, 5.2 Eq.), and CF_3CO_2H (19.7 mM). This mixture was incubated at rt, until the exchange reached equilibrium (2.5 h). At this stage, ^{19}F NMR analysis revealed a 1:9 ratio of surface-bound **2** and **3** (AuNP-$\mathbf{2}_{0.1}\mathbf{3}_{0.9}$, **Figure S16c**). Nanoparticles were precipitated by adding 8:1 v/v Et_2O/EtOH (10 mL). The black solid recovered was resuspended in 7:1 *v*/*v* Et_2O/EtOH (8 mL), sonicated for 10 min and recollected by centrifugation (1312 ×g rcf, 4 °C, 20 min). This operation was repeated a further 2 times. After removing traces of volatile solvents under a stream of compressed air, AuNP-$\mathbf{2}_{0.1}\mathbf{3}_{0.9}$ was re-dissolved in 9:1 *v*/*v* DMF/D_2O (550 μL) and subjected to ^{19}F NMR analysis. Pleasingly, the ratio for the two surface-bound hydrazones calculated by area deconvolution of either the sharp signals for hydrazides in bulk solution and broad nanoparticle signals before nanoparticle purification (**Figure S16c**), agree with the monolayer composition assessed after nanoparticle purification (**Figure S16d** and **Table S7**).

Generation of AuNP-3(e)

To produce exhaustively exchanged AuNP-**3**(e), a solution containing AuNP-**2** (5.37 mg) and 4-fluorotoluene (5.00 mM) was prepared in 9:1 *v*/*v* DMF/D_2O (550 μL) giving 4.87 mM in terms of surface-bound hydrazone **2**. To this, hydrazide **4** was added (10.4 mg, 67.3 μmol), followed by an aliquot of the CF_3CO_2H stock solution (8.90 μL, 1.11 μmol), giving final concentrations of AuNP-**2**

(4.79 mM), **4** (120 mM, 25 Eq.), and CF_3CO_2H (20.1 mM). The mixture was incubated at rt until the exchange reached equilibrium (1.2 h). At this stage, ^{19}F NMR revealed that the broad signal corresponding to AuNP-**2** had completely disappeared indicating full conversion of surface-bound **2** into surface-bound **3** (**Figure S16e**). Nanoparticles were precipitated by adding 8:1 *v*/*v* Et_2O/EtOH (10 mL). The black solid recovered was then washed with Et_2O/EtOH, using the same procedure described above. Traces of volatile solvents were removed from the purified residue under a stream of compressed air, 1 mL water added, and the sample freeze dried to provide AuNP-**3**(e) (4.92 mg) (**Figures S16f and S17**).

Table S7. Summary of nanoparticle samples produced by hydrazone exchange AuNP-**2** → AuNP-**3**(e) → AuNP-**2**(e), showing close agreement between monolayer compositions determined in situ pre- and post-purification, and ex situ following oxidative ligand desorption.

Sample	% **4** released [a]	% **5** released [a]	During exchange[b]		Post purification[c]		Oxidative ligand desorption[d]	
			χ_2	χ_3	χ_2	χ_3	χ_2	χ_3
AuNP-**2**	N/A	N/A	N/A	N/A	0	100	0	100
AuNP-$\mathbf{2}_{0.4}\mathbf{3}_{0.6}$	N/A	57	42	58	43	57	42	58
AuNP-$\mathbf{2}_{0.1}\mathbf{3}_{0.9}$	N/A	87	14	86	12	88	13	87
AuNP-**3(e)**	N/A	100	< 0.03[e]	> 97	< 0.03[e]	> 97	< 0.03[e]	> 97
AuNP-**2(e)**	100	N/A	> 97	< 0.03[e]	> 97	< 0.03[e]	> 97	< 0.03[e]

[a] Determined by in situ ^{19}F NMR (N/A = not applicable). Calculated by area deconvolution of the sharp signals for hydrazides **4** and **5** in bulk solution (**Figures S15** and **S16**).
[b] Determined by in situ ^{19}F NMR. Calculated by area deconvolution of broad nanoparticle signals corresponding to surface-bound **2** and **3** (**Figures S15** and **S16**).
[c] Determined by in situ ^{19}F NMR on purified samples. Calculated by area deconvolution of broad nanoparticle signals corresponding to surface-bound **2** and **3** (**Figures S16b**, **d**, **f**, **h**).
[d] Determined ex situ following oxidative ligand desorption using I_2 (**Figures S19, S20**).
[e] Undetectable by ^{19}F NMR (470 MHz, 9:1 *v*/*v* DMF/D_2O, 16 scans, D1 = 25 s).

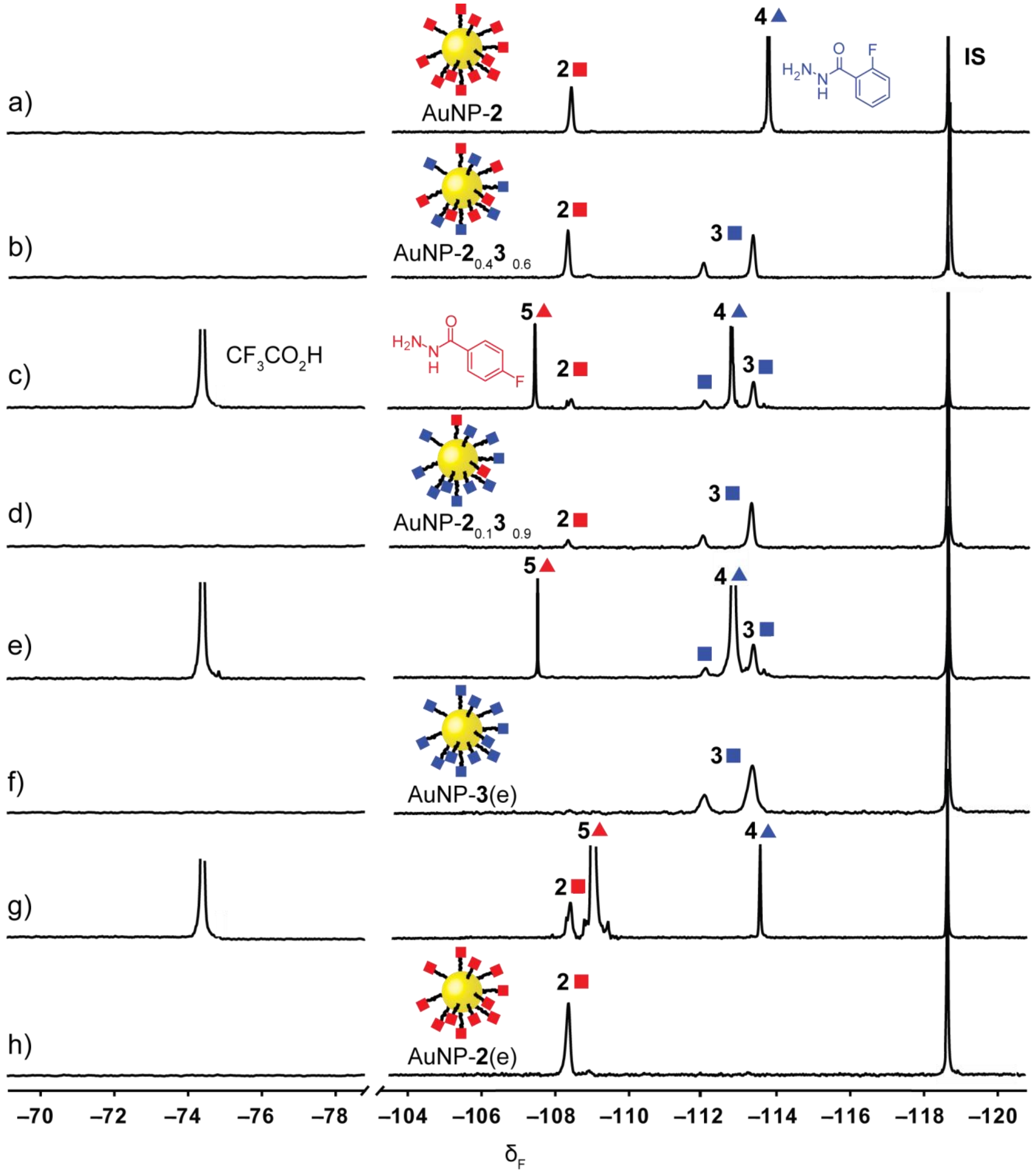

Figure S16 ^{19}F NMR (470 MHz, 9:1 *v*/*v* DMF/D_2O, D1: 25 s) spectra of pre- and post-purification samples prepared by hydrazone exchange AuNP-**2** → AuNP-**2**(e) and mixed-monolayer compositions: a) AuNP-**2** (4.92 mM) and hydrazide **4** (4.93 mM); b) Purified AuNP-$\mathbf{2}_{0.4}\mathbf{3}_{0.6}$ produced by exchange from AuNP-**2** with 1.0 Eq. **4**; c) Crude AuNP-$\mathbf{2}_{0.1}\mathbf{3}_{0.9}$ produced by exchange from AuNP-**2** with 5.2 Eq. **4**; d) Purified AuNP-$\mathbf{2}_{0.1}\mathbf{3}_{0.9}$; e) Crude of AuNP**3**(e) produced by exchange from AuNP-**2** with 25 Eq. **4**; f) Purified AuNP-**3**(e); g) Crude sample of AuNP-**2**(e) produced during exchange with hydrazide **5** and CF_3CO_2H; h) Purified AuNP-**2**(e) prepared by hydrazone exchange. Conditions: AuNP-**3**(e) (4.88 mM), hydrazide **5** (121 mM, 25 Eq.), CF_3CO_2H (19.6 mM), 2.2 h, rt.

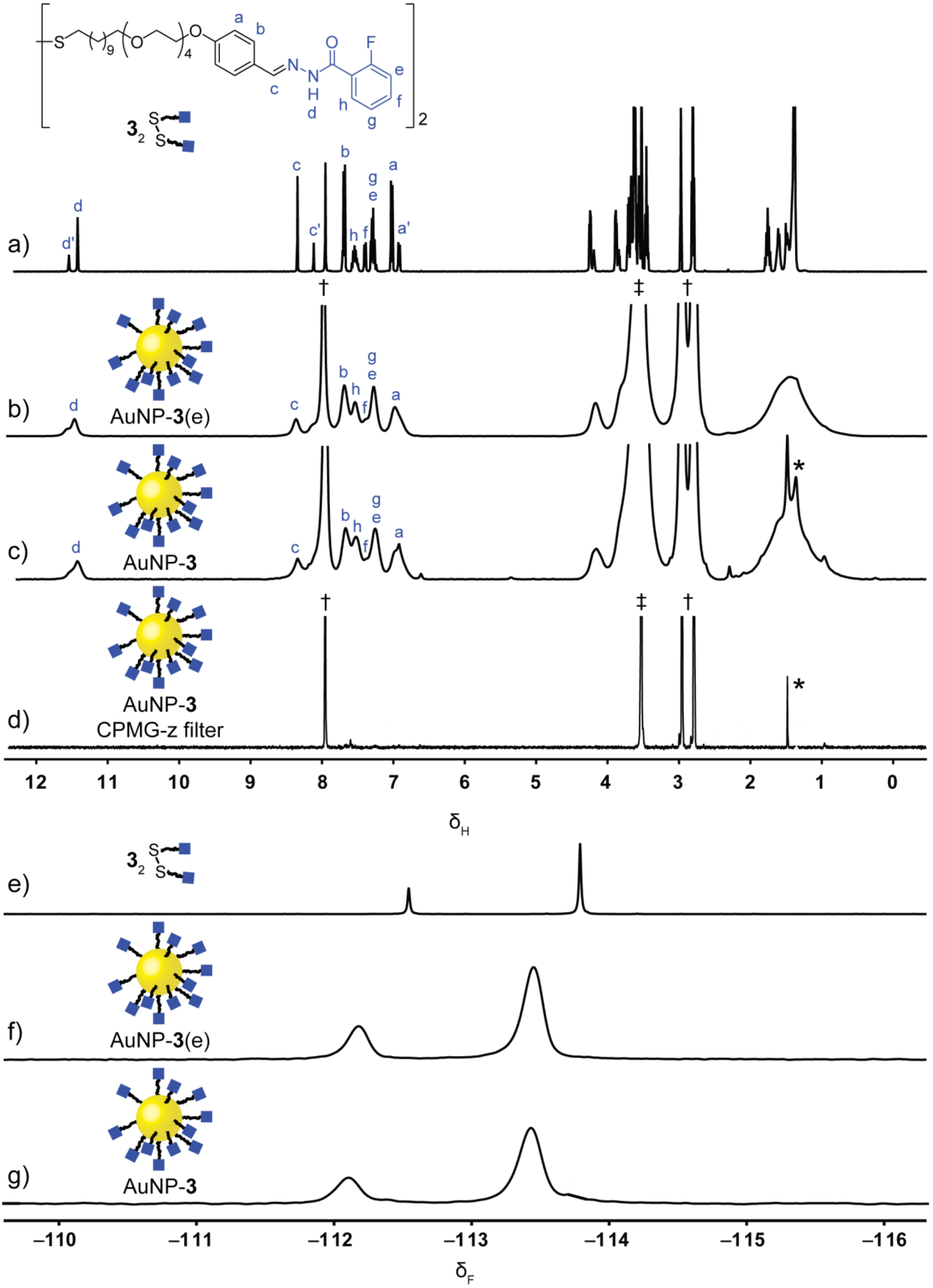

Figure S17. Full sweep-width NMR characterization of AuNP-**3** and AuNP-**3**(e). ^{1}H NMR (500 MHz, [D$_7$]DMF) spectra of: a) disulfide **3**$_2$; b) AuNP-**3**(e) (all sharp signals can be assigned to residual non-deuterated solvents as indicated † = DMF, ‡ = H_2O); c) AuNP-**3**; d) T_2-Filtered ^{1}H NMR spectrum of AuNP-**3** acquired using the CPMG-z pulse sequence,[5] D21 = 0.4 s (all sharp signals can be assigned to residual non-deuterated solvents and impurities as indicated † = DMF, ‡ = H_2O, * = *tert*-butylamine). ^{19}F NMR (470 MHz, [D$_7$]DMF) spectra of: e) disulfide **3**$_2$; f) AuNP-**3**(e); g) AuNP-**3**.

Ex situ NMR Characterization of AuNP-3(e) and mixed monolayer compositions AuNP-2_x3_y: oxidative ligand desorption

Oxidative ligand desorption followed by ex situ ^{19}F NMR analysis was performed to verify the monolayer compositions of all AuNP-$\mathbf{2}_x\mathbf{3}_y$ samples produced via hydrazone exchange.

The characteristic fragmentation patterns of disulfide $\mathbf{2}_2$ (**Figure S12**, **Table S6**) and $\mathbf{3}_2$ (**Figure S18** and **Table S8**) in the presence of iodine were first established. Following the same protocol described above for AuNP-**1** and AuNP-**2**, a colloidally stable solution of AuNP-$\mathbf{2}_x\mathbf{3}_y$ containing 4-fluorotoluene as internal standard (5.00 mM) was prepared in 9:1 *v*/*v* DMF/D_2O (600 μL) and the concentration(s) of surface-bound hydrazone(s) **2** and/or **3** assessed. Iodine (2 mg) was then added and the solution incubated at rt, recording ^{19}F NMR spectra at various timepoints. In each case, the characteristic broad nanoparticle signals were observed to disappear, and sharp signals appeared corresponding to disulfides $\mathbf{2}_2$ and/or $\mathbf{3}_2$, as well as signals corresponding to 4-fluorobenzoic acid and/or 2-fluorobenzoic acid. In all cases the total concentration of fluorinated species measured in bulk solution remained constant and always equal to the total amount of surface-bound ligands estimated before iodine addition (within experimental error).

Pleasingly, the ratios of surface-bound **2** and **3** species determined from oxidative ligand desorption were found to be in excellent agreement with the results obtained from both pre- and post-purification analysis by in situ NMR spectroscopy (**Table S7**).

Table S8. Concentrations of fluorine-containing species during incubation of disulfide $\mathbf{3}_2$ with iodine.

Spectrum **Figure S18**	*t* / h	[$\mathbf{3}_2$] / mM	[**4**] / mM[a]	Overall conc./ mM
b)	0	3.22		3.22
c)	1.37	0.89	2.30	3.19
d)	2.25	0.38	2.82	3.20
e)	4.43		3.22	3.22
f)	7.93		3.25	3.25

[a] Including the concentration of 4-fluorobenzoic acid.

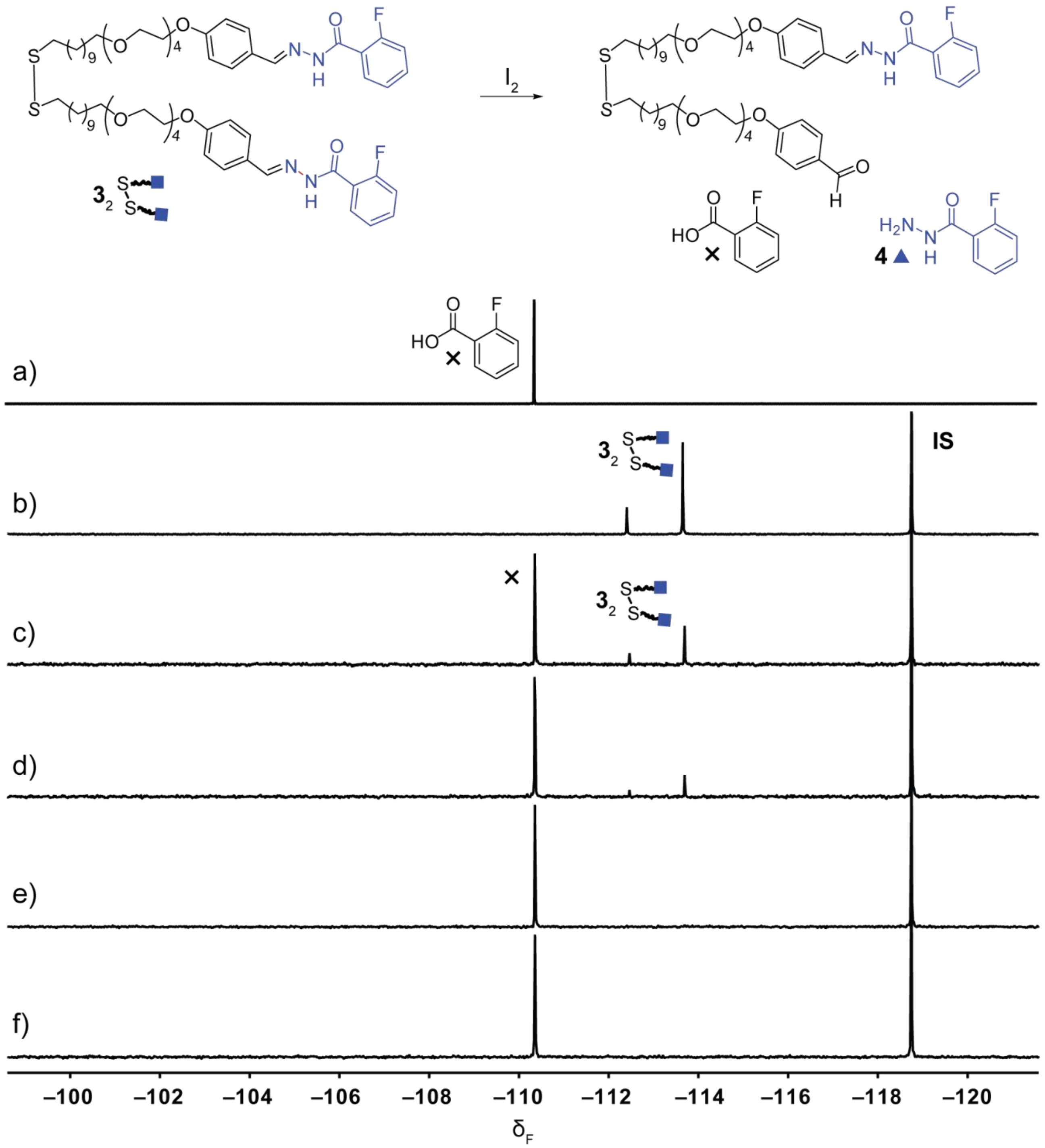

Figure S18. Solution of disulfide **3**$_2$ incubated with iodine. a) ^{19}F NMR (470 MHz, 9:1 *v*/*v* DMF/D$_2$O,) spectrum of 2-fluorobenzoic acid; b) ^{19}F NMR (470 MHz, 9:1 *v*/*v* DMF/D$_2$O, D1: 25 s) spectrum of disulfide **3**$_2$. [**3**$_2$] = 3.22 mM (internal standard 5 mM 4-fluorotoluene, δ_F –118.68 ppm); c–f) ^{19}F NMR (470 MHz, 9:1 *v*/*v* DMF/D$_2$O, D1: 25 s) spectra recorded 82, 135, 266 and 476 min after addition of iodine to a solution of disulfide 3$_2$. Total concentration of all fluorinated species remained constant during the experiment time-course and in close agreement with the concentration of disulfide **3**$_2$ measured at t = 0 min (**Table S8**).

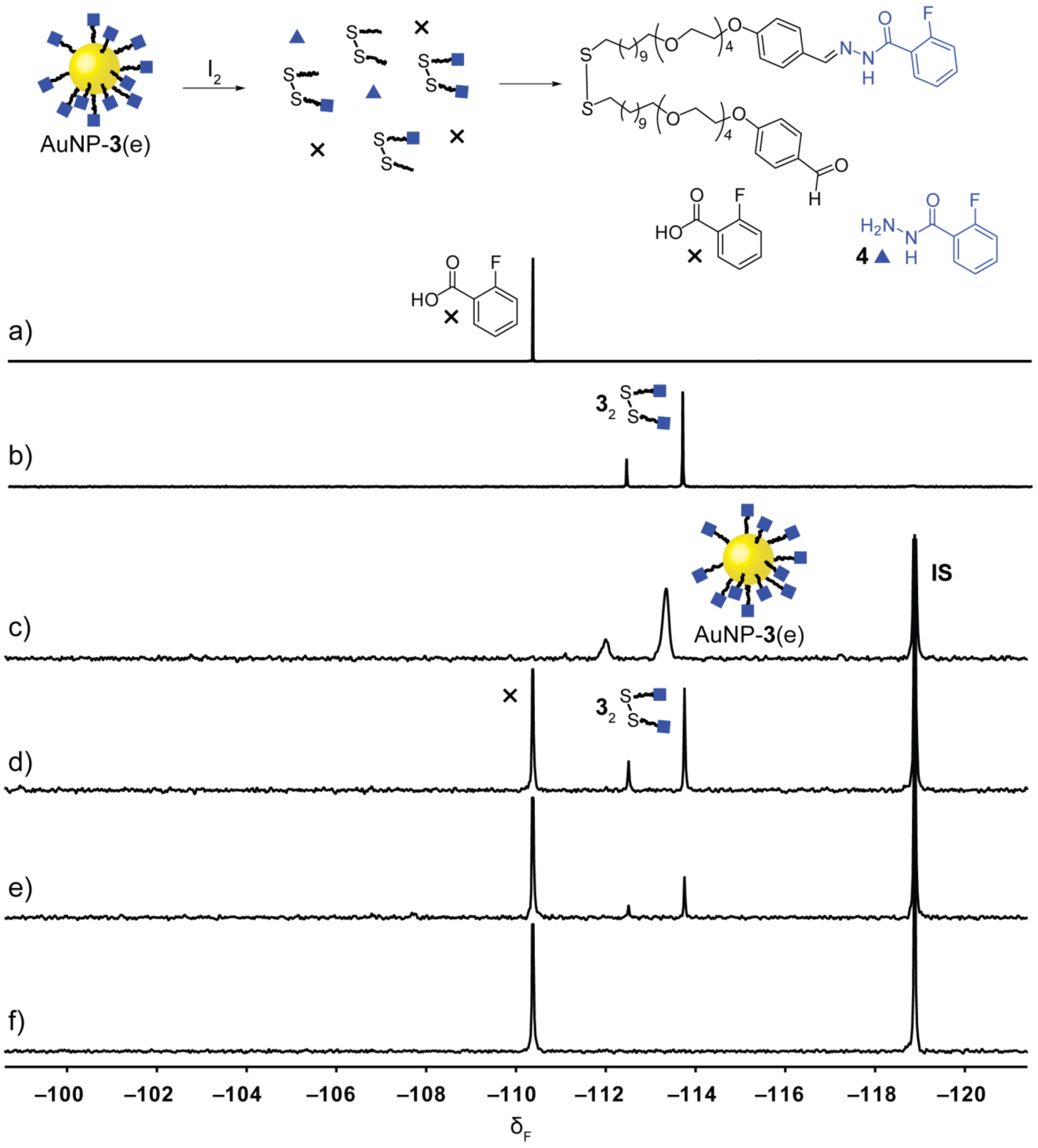

Figure S19. Oxidative ligand desorption performed on a solution of purified AuNP-**3**(e): a) ^{19}F NMR (9:1 *v*/*v* DMF/D_2O, 470 MHz) spectrum of 2-fluorobenzoic acid; b) ^{19}F NMR (470 MHz, 9:1 *v*/*v* DMF/D_2O) spectrum of disulfide **3**$_2$; c) ^{19}F NMR (470 MHz, 9:1 *v*/*v* DMF/D_2O, D1: 25 s) spectrum of AuNP-**3**(e). Nanoparticle-bound [**3**] = 3.17 mM (internal standard 5 mM 4-fluorotoluene, δ_F –118.68 ppm); d–g) ^{19}F NMR (470 MHz, 9:1 *v*/*v* DMF/D_2O, D1: 25 s) spectra recorded 48, 143 and 270 min after addition of iodine to a solution of AuNP-**3**(e). Total concentration of all fluorinated species remained constant during the experiment time-course and in close agreement with on-nanoparticle concentration measured at t = 0 min (**Table S9**).

Table S9. Concentrations of fluorine-containing species during oxidative ligand desorption from AuNP-**3**(e).

Spectrum **Figure S19**	*t* / h	[AuNP-**3**(e)] / mM	[$\mathbf{3}_2$] / mM	[**4**] / mM[a]	Overall conc./ mM
c)	0	3.17			3.17
d)	0.80		1.46	1.74	3.20
e)	2.38		0.34	2.82	3.16
f)	4.50			3.18	3.18

[a] Including the concentration of 4-fluorobenzoic acid.

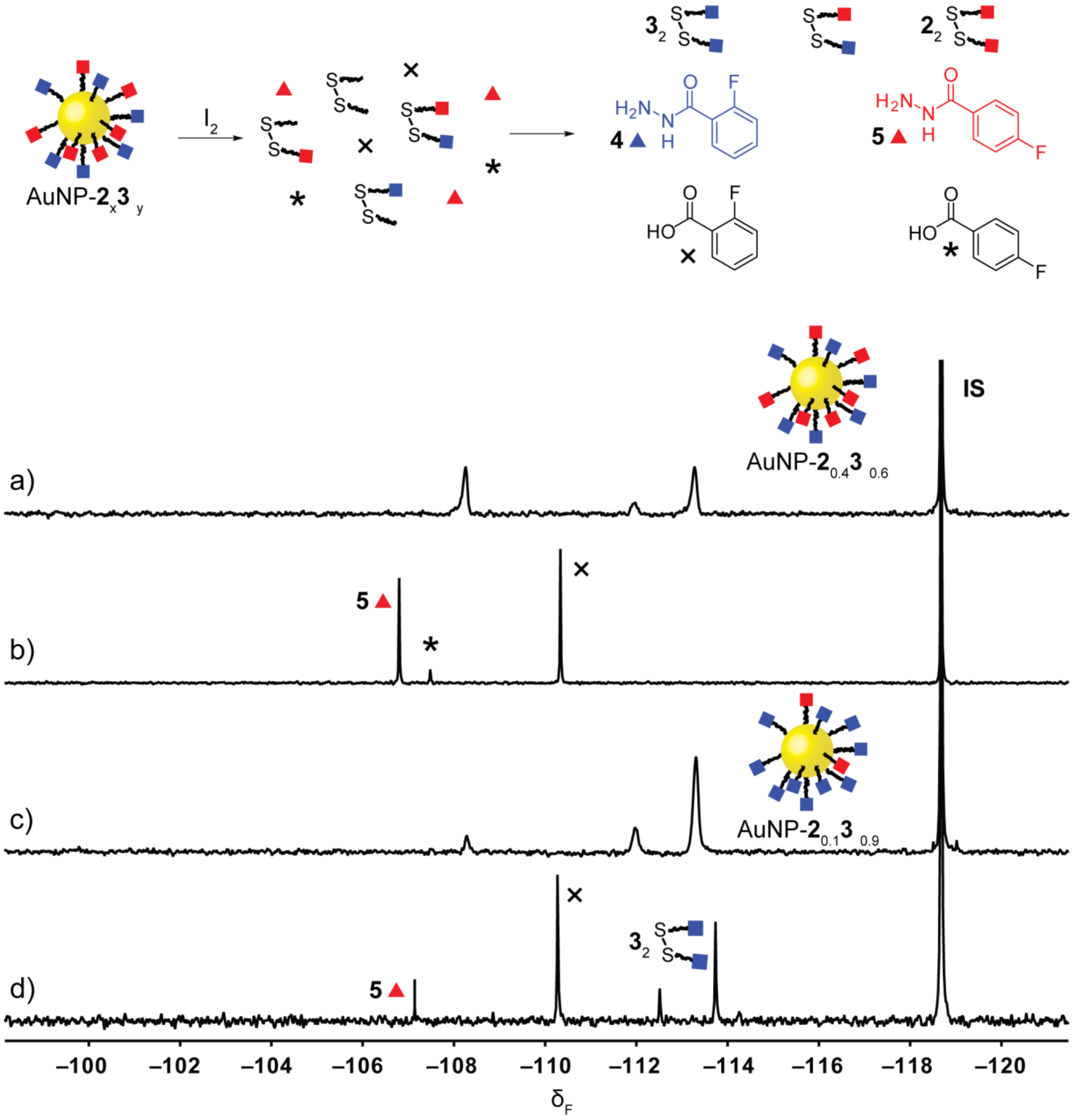

Figure S20. Oxidative ligand desorption performed on solutions of purified AuNP-$\mathbf{2}_x\mathbf{3}_y$ with mixed monolayer compositions: a) ^{19}F NMR (470 MHz, 9:1 *v*/*v* DMF/D_2O, D1: 25 s) spectrum of AuNP-$\mathbf{2}_{0.4}\mathbf{3}_{0.6}$. Nanoparticle-bound [**2**+**3**] = 2.18 mM (internal standard 5 mM 4-fluorotoluene, δ_F –118.68 ppm); b) ^{19}F NMR (470 MHz, 9:1 *v*/*v* DMF/D_2O, D1: 25 s) spectrum recorded 325 min after addition of iodine to a solution of AuNP-$\mathbf{2}_{0.4}\mathbf{3}_{0.6}$. c) ^{19}F NMR (470 MHz, 9:1 *v*/*v* DMF/D_2O, D1: 25 s) spectrum of AuNP-$\mathbf{2}_{0.1}\mathbf{3}_{0.9}$. Nanoparticle-bound [**2**+**3**] = 1.17 mM (internal standard 5 mM 4-fluorotoluene, δ_F –118.68 ppm); d) ^{19}F NMR (470 MHz, 9:1 *v*/*v* DMF/D_2O, D1: 25 s) spectrum recorded 231 min after addition of iodine to a solution of AuNP-$\mathbf{2}_{0.1}\mathbf{3}_{0.9}$.Total concentration of all fluorinated species remained constant during the experiment time-course and in close agreement with on-nanoparticle concentration measured at t = 0 min (**Table S7**).

6.2 Generation and characterization of AuNP-2(e)

AuNP-**2**(e) were produced by dynamic covalent exchange from AuNP-**3**(e) with an excess of hydrazide **5**. A solution containing AuNP-**3**(e) (6.11 mg) and 4-fluorotoluene (5.00 mM) was prepared in 9:1 *v*/*v* DMF/D_2O (620 μL) giving 4.97 mM in terms of surface-bound hydrazone **3**. To this, hydrazide **5** was added (11.8 mg, 76.5 μmol), followed by an aliquot of the CF_3CO_2H stock solution (11.7 μL, 12.4 μmol), giving final concentrations of AuNP-**3**(e) (4.88 mM), **5** (121 mM, 25 Eq.), and CF_3CO_2H (19.6 mM). The mixture was incubated at rt until the exchange reached equilibrium (2.2 h). At this stage, ^{19}F NMR revealed that the broad signal corresponding to AuNP-**2** had completely disappeared indicating full conversion of surface-bound **3** into surface-bound **2** (**Figure S16g**). Nanoparticles were precipitated by adding 8:1 v/v Et_2O/EtOH (10 mL). The black solid recovered was then washed with Et_2O/EtOH, using the same procedure described above. Traces of volatile solvents were removed from the purified residue under a stream of compressed air, 1 mL water added, and the sample freeze dried to provide AuNP-**2**(e) (5.84 mg) (**Figures S16h** and **S21**).

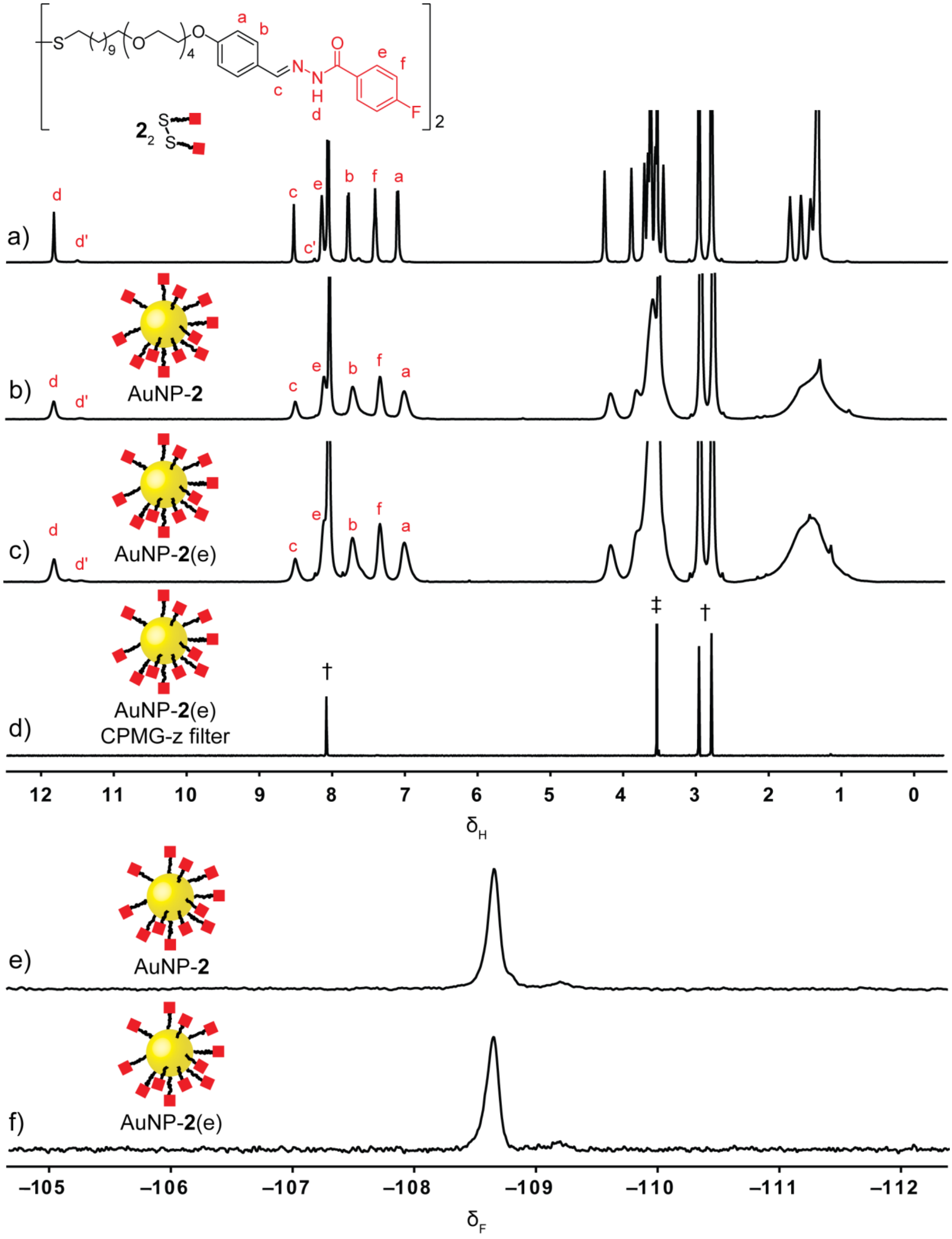

Figure S21. Full sweep-width NMR characterization of AuNP-**2** and AuNP-**2**(e). ^{1}H NMR (500 MHz, [D$_7$]DMF) spectra of: a) disulfide **2**$_2$; b) AuNP-**2**; c) AuNP-**2**(e); d) T_2-Filtered ^{1}H NMR spectrum of AuNP-**2**(e) acquired using the CPMG-z pulse sequence,[5] D21 = 0.4 s (all sharp signals can be assigned to residual non-deuterated solvents and impurities as indicated † = DMF, ‡ = H_2O). ^{19}F NMR (470 MHz, [D$_7$]DMF) spectra of: e) AuNP-**2**; g) AuNP-**2**(e).

Ex situ NMR Characterization of AuNP-2(e): oxidative ligand desorption

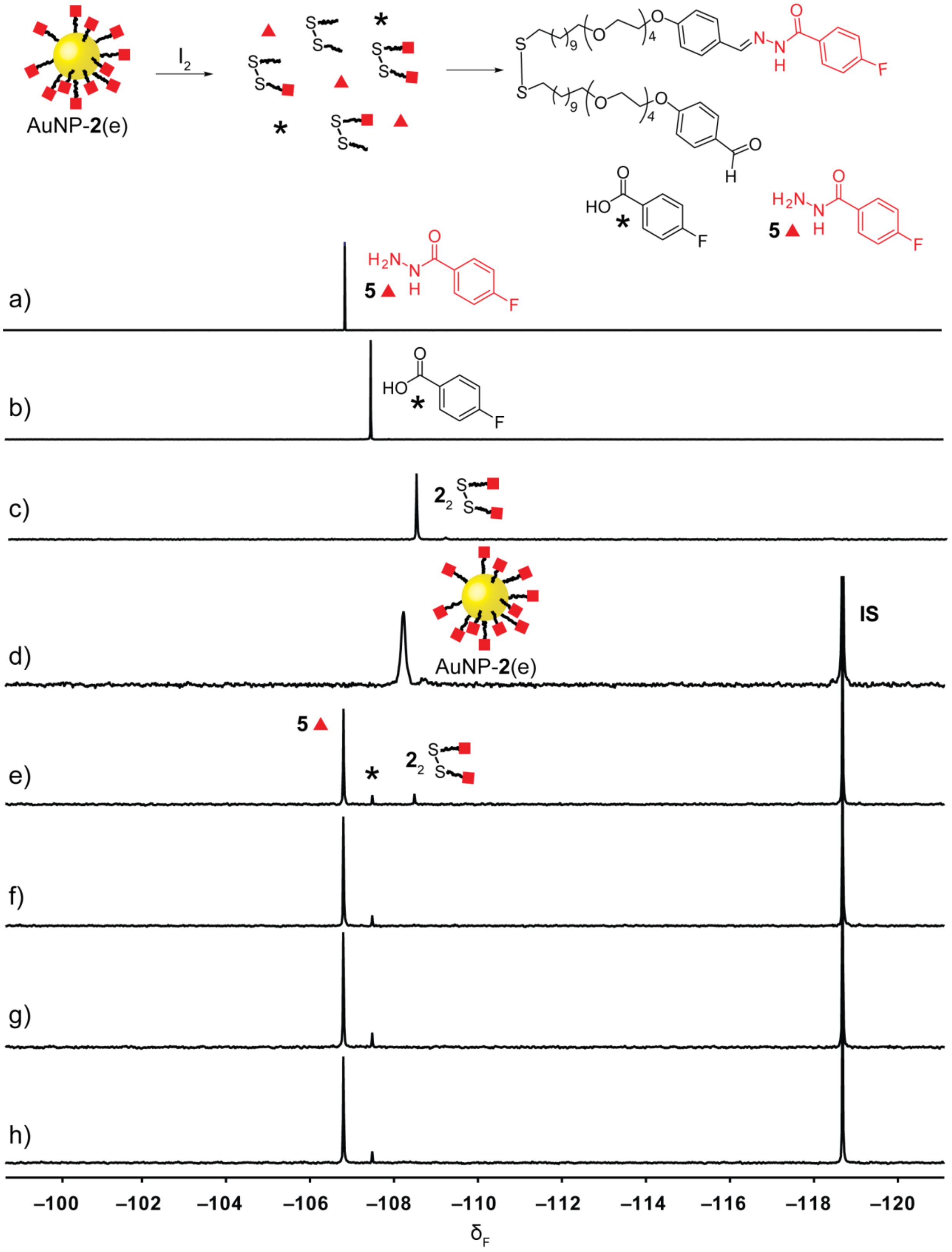

Figure S22. Oxidative ligand desorption performed on a solution of purified AuNP-**2**(e): a) ^{19}F NMR (470 MHz, 9:1 *v*/*v* DMF/D_2O) spectrum of hydrazide **5**; b) ^{19}F NMR (470 MHz, 9:1 *v*/*v* DMF/D_2O) spectrum of 4-fluorobenzoic acid; c) ^{19}F NMR (470 MHz, 9:1 *v*/*v* DMF/D_2O) spectrum of disulfide **2**$_2$; d) ^{19}F NMR (470 MHz, 9:1 *v*/*v* DMF/D_2O, D1: 25 s) spectrum of AuNP-**2**(e). Nanoparticle-bound [**2**] = 3.05 mM (internal standard 5 mM 4-fluorotoluene, δ_F –118.68 ppm); e–h) ^{19}F NMR (470 MHz, 9:1 *v*/*v* DMF/D_2O, D1: 25 s) spectra recorded 82, 157, 228 and 318 min after addition of iodine to a solution of AuNP-**2**(e). Total concentration of all fluorinated species remained constant during the experiment time-course and in close agreement with on-nanoparticle concentration measured at *t* = 0 min (**Table S10**).

Table S10. Concentrations of fluorine-containing species during oxidative ligand desorption from AuNP-**2**(e).

Spectrum **Figure S22**	*t* / h	[AuNP-**2**(e)] / mM	[**2**$_2$] / mM	[**5**] / mM[a]	Overall conc./ mM
d)	0.00	3.05			3.05
e)	1.37		0.30	2.78	3.08
f)	2.62			3.04	3.04
g)	3.80			3.06	3.06
h)	5.30			3.07	3.07

[a] Including the concentration of 4-fluorobenzoic acid.

6.3 Verification of nanoparticle integrity following dynamic covalent modification

In order to confirm that reactions happening at the periphery of the surface-bound monolayers do not alter features of the nanoparticle core (e.g. size, shape and size distribution), TEM analysis was performed on a purified sample of AuNP-**2**(e) obtained after back-and-forth hydrazone exchange AuNP-**2** → AuNP-**3**(e) → AuNP-**2**(e). Analysis of the micrographs obtained for AuNP-**2** and AuNP-**2**(e) revealed only a slight increase of the mean diameter (within less than one standard deviation) while the nanoparticle size distribution appeared almost unchanged (**Figure S23**). Further comparison of the mean and median values indicated that the symmetrical distribution of sizes was preserved, suggesting that the observed size increase is not the result of outlier values nor affected by selective removal of smaller nanoparticles during the various synthetic manipulations.

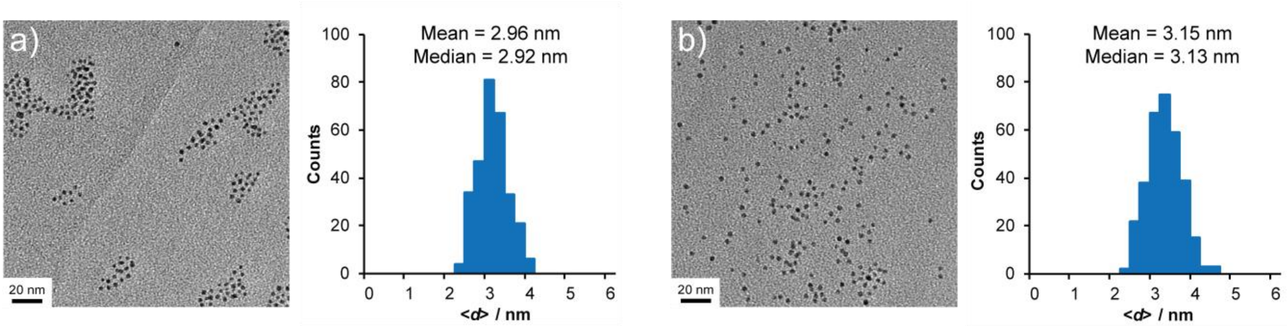

Figure S23. Representative TEM micrographs recorded before and after back-and-forth hydrazone exchange AuNP-**2** → AuNP-**3**(e) → AuNP-**2**(e). a) TEM micrograph (scale bar 20 nm) and corresponding size distribution of AuNP-**2**: <*d*> = 2.96 ± 0.40 nm (13% dispersity). b) TEM micrograph (scale bare 20 nm) and respective size distribution of AuNP-**2**(e): <*d*> = 3.15 ± 0.42 nm (14% dispersity). Size distributions were determined from a minimum of 200 particle measurements.

Thermal decomposition analysis of AuNP-**2**(e) surface-bound monolayer resulted in progressive mass loss as temperature increased above the onset melting temperature (T_m) at 292 °C. Assuming an isotropic gold core (d = 3.15 nm), the organic mass lost between 292–900 °C was used to estimate molar weight and number of ligands per nanoparticle (**Figure S24** and **Table S11**). The small increase in mass (< 2.5%) at low temperatures can be ascribed to the same effects as discussed above for AuNP-**1**. As a result, it was possible to calculate the expected concentration of surface-bound **2** in a colloidal solution of AuNP-**2**(e) of known mass, which could be compared to the concentration of this sample as determined by solution-state ^{19}F NMR spectroscopy. The excellent agreement between TGA and NMR results indicates the absence of free aldehyde within the AuNP-**2**(e) surface-bound monolayer, as well as confirming no oxidation or other decomposition of the electrophilic moiety during hydrazone exchange AuNP-**2** → AuNP-**2**(e) (**Table S11**).

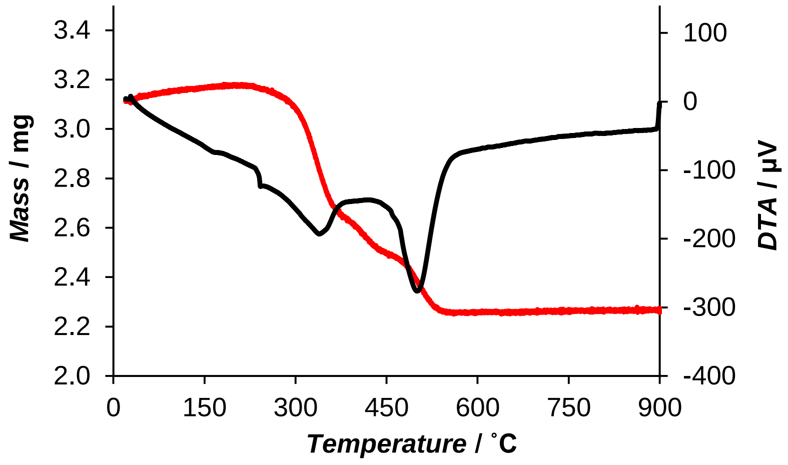

Figure S24. TGA (red curve) and DTA (black curve) plots for AuNP-**2**(e) (3.10 mg). The sample was heated under air at a ramp rate of 10 °C min^{-1} over the range 20–900 °C.

Table S11. AuNP-**2**(e) structural characterisation data calculated from TGA, DTA and TEM, compared with data obtained from solution-state ^{19}F NMR spectroscopy.

<*d*> / nm	AuNP-**2** surface area / nm^2	Au %	Surface-bound **2** %	Ligand **2** surface area / nm^2	AuNP-**2** M.W. / g mol^{-1}	Molecules **2** per nanoparticle	[**2**] / mM	
							TGA	NMR
3.15	31.2	73.1	26.9	0.275	2.60 x 10^5	113	2.90[a]	3.05[b]

[a] Calculated for a colloidal solution of AuNP-**2**(e) (4.00 mg) in 9:1 *v*/*v* DMF/D_2O (600 µL), using molar weight nanoparticle estimated by TGA analysis.
[b] Determined ex situ following oxidative ligand desorption of purified AuNP-**2**(e) (4.00 mg) in 9:1 *v*/*v* DMF/D_2O (600 µL) in presence of I_2. (**Figure S22** and **Table S10**).

7. Dynamic covalent modification of ‘electrophilic’ nanoparticles with electrophilic modifiers

7.1 Generation of single-component aldehyde-functionalized monolayers AuNP-6

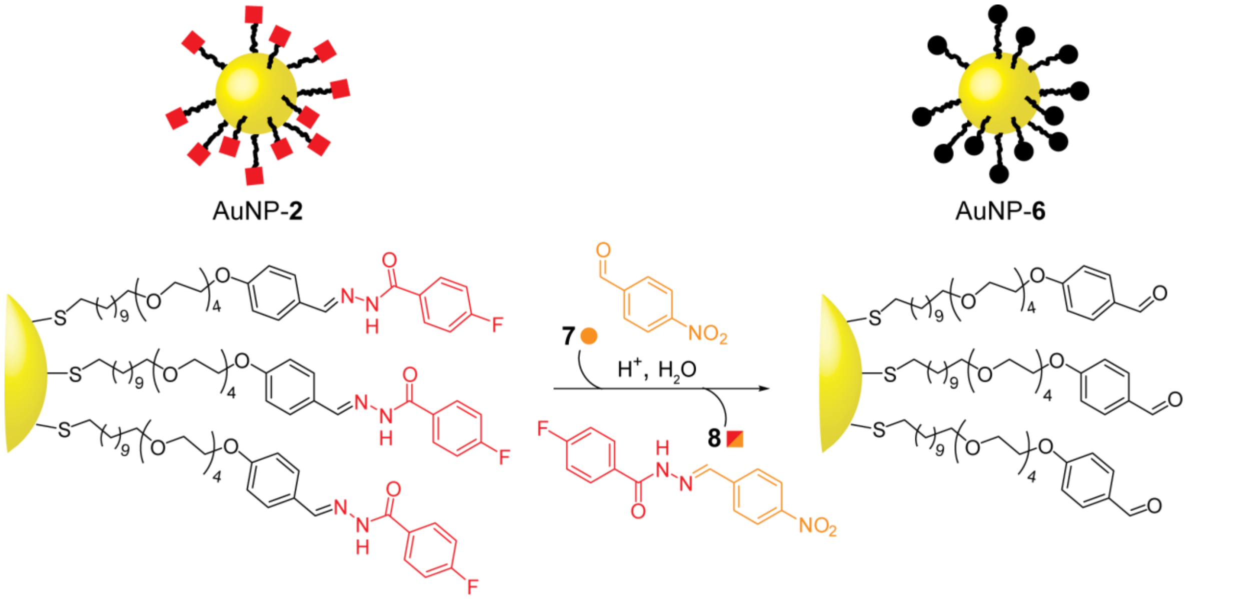

Concentrations of all fluorine-containing species were determined by quantitative ^{19}F NMR in the presence of 4-fluorotoluene as an internal standard of known concentration.

A stock solution of CF_3CO_2H was prepared in 9:1 *v*/*v* DMF/D_2O with 4-fluorotoluene as internal standard (5.00 mM) and concentration measured as 1.07 M.

A colloidal solution of AuNP-**2** (6.2 mg) containing 4-fluorotoluene (5.00 mM) was prepared in 9:1 *v*/*v* DMF/D_2O (600 μL) giving 5.10 mM in terms of surface-bound **2** (3.1 μmol). Then, aldehyde **7** (23.5 mg, 155 μmol) and an aliquot of the CF_3CO_2H stock solution (11.4 μL,12.2 μmol) were added, giving final concentrations of AuNP-**2** (5.00 mM), aldehyde **7** (250 mM, 50.0 Eq.), and CF_3CO_2H (20 mM). This mixture was heated to 45 °C. After 2.7 h, ^{19}F NMR analysis (**Figure S25b**) revealed a decrease of the broad signal at –108.86 ppm corresponding to nanoparticle-bound **2** and quantitative conversion to a sharp signal at –107.72 ppm for hydrazone **8** in bulk solution. At this stage, the concentration measured for hydrazone **8** was 2.7 mM indicating that 54% of surface-bound **2** was hydrolysed revealing surface-bound **6**. After 21.7 h, the signal for nanoparticle-bound **2** was no-longer visible and the concentration of hydrazone **8** reached 4.8 mM (96% conversion). After 26 h,^{19}F NMR analysis indicated 100% hydrolysis of nanoparticle-bound **2** according to the concentration of hydrazone **8** observed in bulk solution (**Figure S25** and **Table S12**). The mixture was cooled to room temperature. Nanoparticle precipitation was achieved by addition of 10:2:1 *v*/*v* Et_2O/EtOH/H_2O (10 mL), followed by sonication (10 min, 20 °C), and centrifugation 1935 ×g rcf, 10 min, 4 °C). The colourless supernatant was carefully discharged, then the black solid obtained was washed using the following procedure: nanoparticles were dispersed in 1:1 *v*/*v* EtOH/Et_2O (10 mL), sonicated (10 min, 20 °C), and recollected by centrifugation (1935 ×g rcf, 4 °C, 10 min). This operation was repeated a further 3 times, progressively increasing the amount of EtOH used in the mixture (from 1:1 to 3:1). At this stage, no unbound molecular species were detected in the supernatant by TLC or NMR analysis. Traces of volatile solvents were removed from the purified residue under a stream of compressed air, 1 mL water added, and the sample freeze dried to provide AuNP-**6** (5.8 mg).

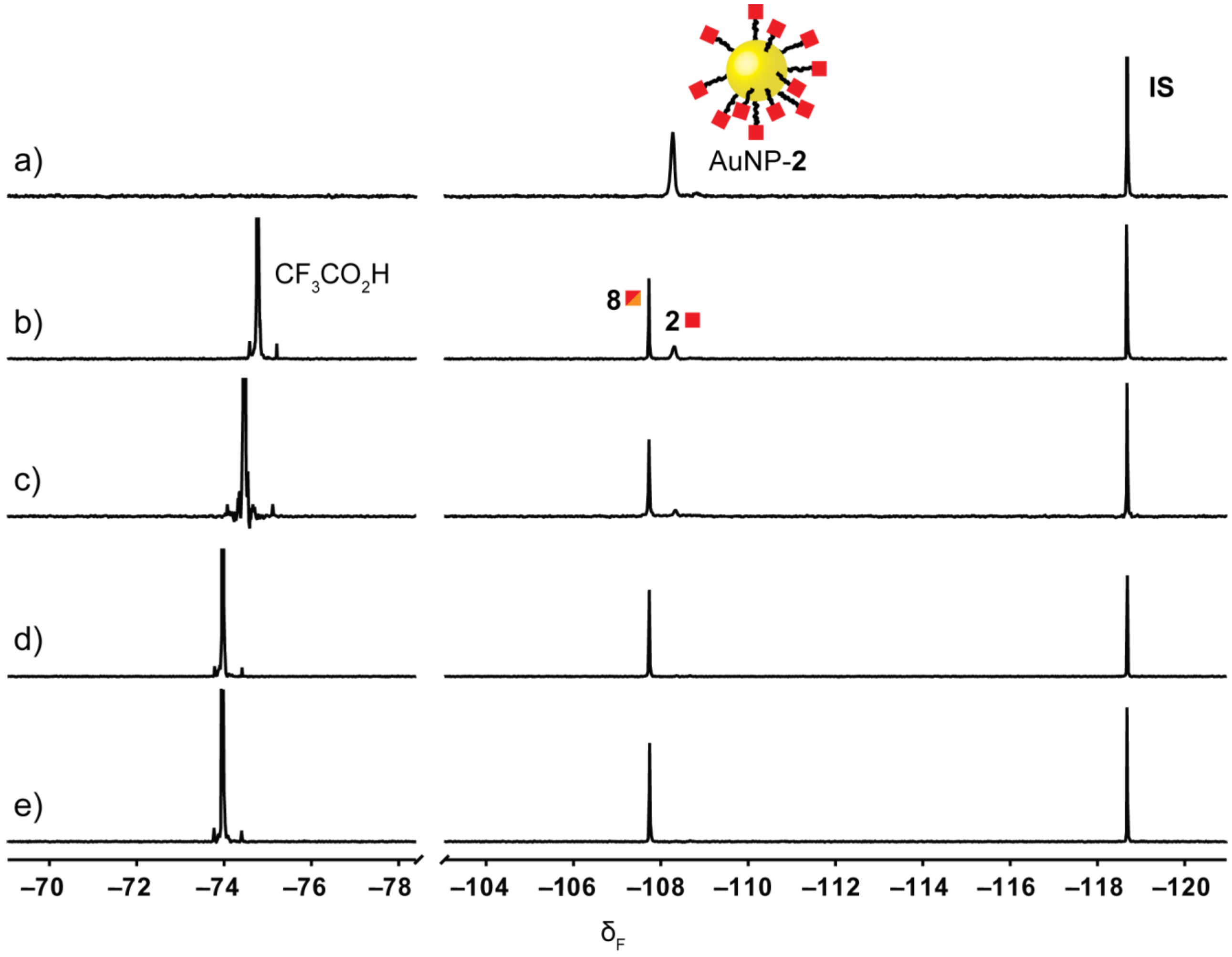

Figure S25. In situ monitoring of dynamic covalent exchange from AuNP-**2** to AuNP-**6** by ^{19}F NMR (470 MHz, 9:1 *v*/*v* DMF/D_2O, D1: 25 s): a) AuNP-**2** (5.1 mM); b–e) Reaction mixture incubated for 2.7, 7.3, 21.7 and 26 h after addition of CF_3CO_2H. Conditions: AuNP-**2** (5.00 mM), aldehyde **7** (250 mM, 50 Eq.), CF_3CO_2H (20.5 mM), 45 °C, 26 h. Exhaustive hydrolysis of nanoparticle-bound hydrazone **2** was confirmed according to the concentration of hydrazone **8** measured in bulk solution. IS: internal standard (4-fluorotoluene, 5.00 mM). For concentrations of all species, see **Table S12**.

Table S12. Concentrations of fluorine-containing species during dynamic covalent exchange from AuNP-**2** to AuNP-**6.**

Spectrum **Figure S25**	*t* / h	[**2**] / mM	[**8**] / mM	% hydrolysis surface-bound **2**[a]	Overall conc. / mM[c]
a)	0.00	5.13		N/A	5.13
b)	2.70	2.35	2.72	54	5.07
c)	7.28	1.05	4.02	80	5.07
d)	21.7	n.d.[b]	4.84	96	4.84
e)	25.9	n.d.[b]	5.14	100	5.14

[a] Determined by *in situ* ^{19}F NMR (N/A = not applicable). Calculated by deconvolution of sharp signal corresponding to hydrazone **8** in bulk solution, before nanoparticle purification (**Figure S25**).
[b] Signal corresponding to nanoparticle-bound 2 undetectable by ^{19}F NMR (9:1 *v*/*v* DMF/D_2O, 470 MHz, 16 scans, D1 = 25 s).
[c] Concentration of CF_3CO_2H not inlcuded.

In situ NMR Characterization of AuNP-6

The molecular composition of AuNP-**6** surface-bound monolayer was investigated using solution-state ^{1}H and ^{19}F NMR spectroscopy (**Figure S26**).

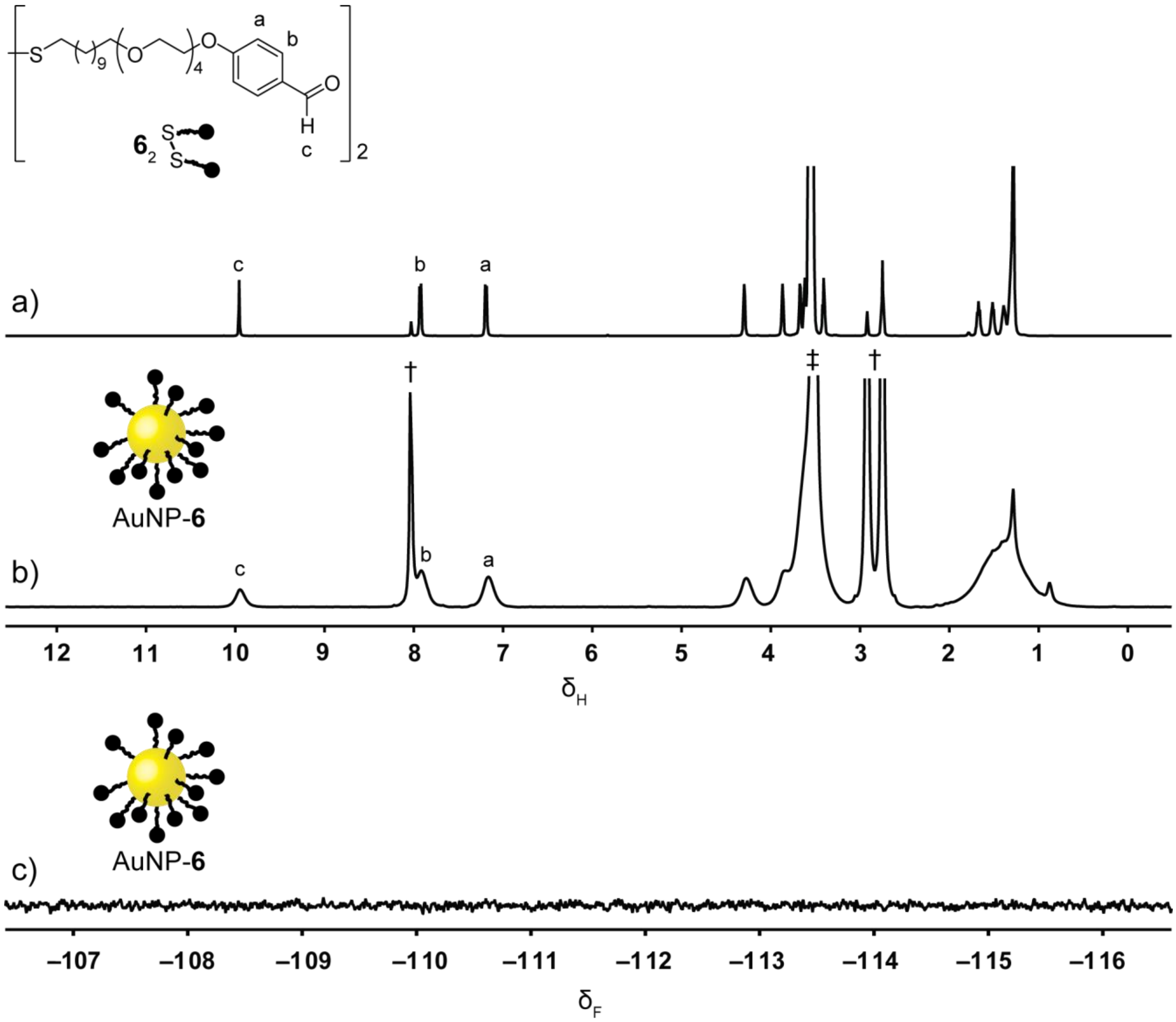

Figure S26. Full sweep-width NMR characterization of AuNP-**6**. a) ^{1}H NMR (500 MHz, [D7]DMF, rt) spectrum of disulfide **6**2; b) ^{1}H NMR (500 MHz, [D7]DMF, rt) spectrum of AuNP-**6**. All sharp signals can be assigned to residual non-deuterated solvents as indicated († = DMF, ‡ = H_2O). c) ^{19}F NMR (470 MHz, [D7]DMF, rt) spectrum of AuNP-**6**.

Purity from non-solvent unbound contaminants was confirmed by T_2-filtered NMR analysis with a delay time set at 0.4 s (**Figure S27**), as described for AuNP-**1** above.

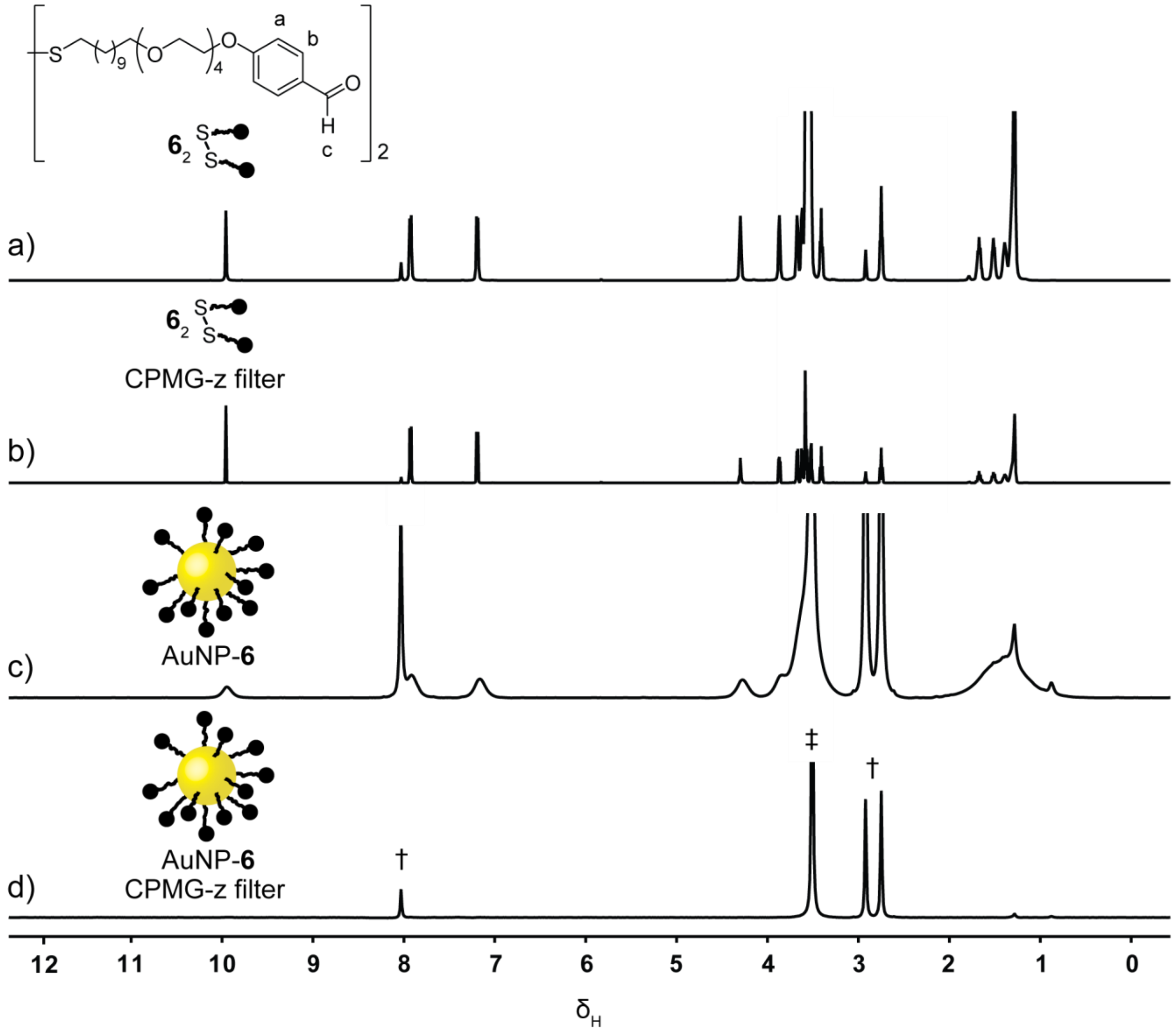

Figure S27. NMR Characterization of AuNP-**6** purity by relaxation time filtered spectra: a) ^{1}H NMR (500 MHz, [D$_7$]DMF) spectrum of disulfide **6**$_2$; b) T_2-filtered 1H NMR (500 MHz, [D$_7$]DMF) spectrum of disulfide **6**$_2$ acquired using the CPMG-z pulse sequence,[5] D21 = 0.4 s; c) ^{1}H NMR (500 MHz, [D$_7$]DMF) spectrum of AuNP-**6**; d) T_2-Filtered ^{1}H NMR (500 MHz, [D$_7$]DMF) spectrum of AuNP-**6** acquired using the CPMG-z pulse sequence,[5] D21 = 0.4 s. All sharp signals can be assigned to residual non-deuterated solvents as indicated († = DMF, ‡ = H_2O).

Ex situ NMR Characterization of AuNP-6: oxidative ligand desorption

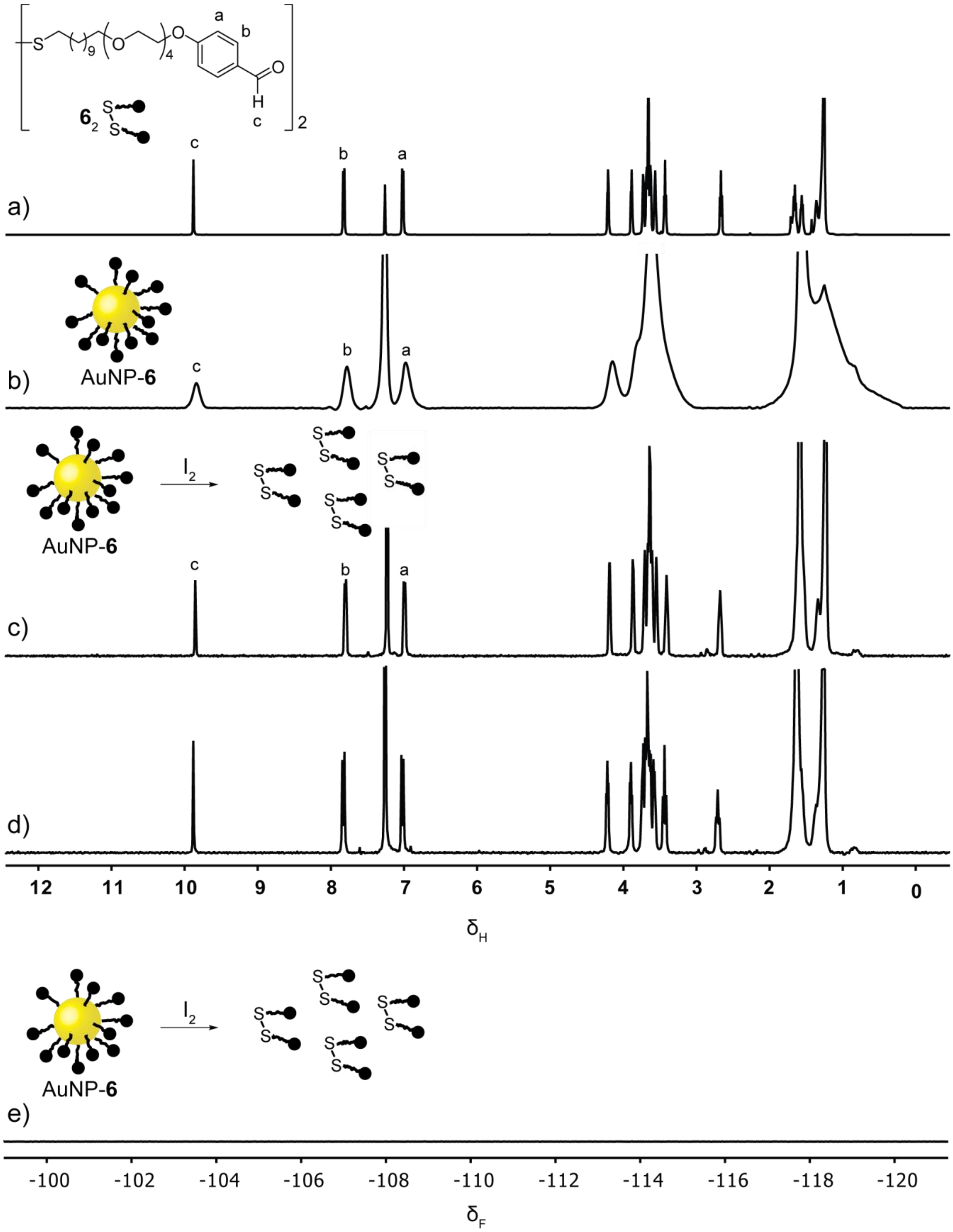

Figure S28. Oxidative ligand desorption performed on freshly prepared AuNP-**6**: a) Quantitative ^1H NMR (500 MHz, $CDCl_3$, D1 = 30 s) spectrum of disulfide **6**$_2$; b) Quantitative ^1H NMR (400 MHz, $CDCl_3$, D1 = 30 s) spectrum of AuNP-**6** before addition of iodine; c–d) Quantitative ^1H NMR (400 MHz, $CDCl_3$, D1 = 30 s) spectra recorded 180 and 510 min after addition of iodine to a solution of AuNP-**6**; e) ^{19}F NMR (376 MHz, $CDCl_3$) spectrum recorded 510 min after addition of iodine to a solution of AuNP-**6.**

7.2 Generation of two-component hydrazone/aldehyde monolayers AuNP-2_x6_y

Generation of AuNP-$2_{0.2}6_{0.8}$ by kinetic trapping

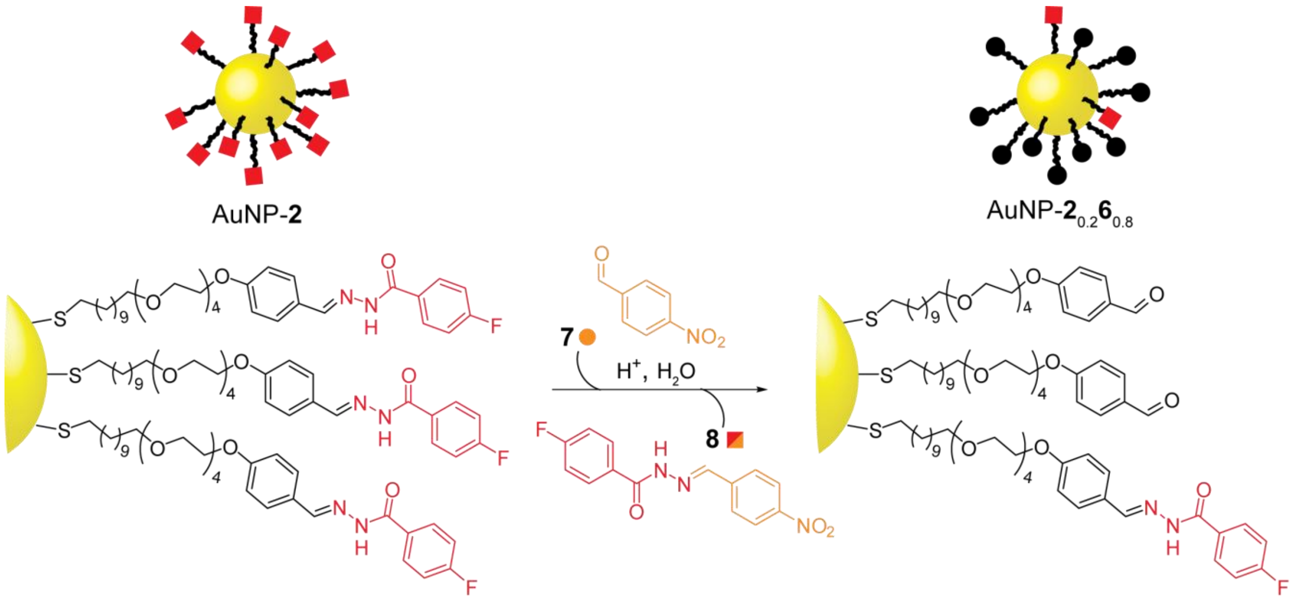

Concentrations of all fluorine-containing species were determined by quantitative ^{19}F NMR in the presence of 4-fluorotoluene as an internal standard of known concentration.

A stock solution of CF_3CO_2H was prepared in 9:1 *v*/*v* DMF/D_2O with 4-fluorotoluene as internal standard (5.00 mM) and concentration determined by ^{19}F NMR.

A colloidal solution of AuNP-**2** (10.7 mg) containing 4-fluorotoluene (5.00 mM) was prepared in 9:1 *v*/*v* DMF/D_2O (1 mL) giving 5.01 mM in terms of surface-bound hydrazone **2**. Then, aldehyde **7** (37.9 mg, 250 μmol) was added, followed by an aliquot of the CF_3CO_2H stock solution (22.7 μL, 19.9 μmol), giving final concentrations of AuNP-**2** (4.85 mM), **4** (244 mM, 50 Eq.), and CF_3CO_2H (20.5 mM). This mixture was incubated at 45 °C and the reaction was followed by ^{19}F NMR (**Figure S29** and **Table S13**). The first spectrum recorded 2.5 h after addition of CF_3CO_2H revealed a decrease of the broad signal at –108.86 ppm corresponding to nanoparticle-bound **2** and quantitative conversion to a sharp signal at –107.72 ppm corresponding to hydrazone **8** in bulk solution. At this stage, the concentration measured for hydrazone **8** was 2.49 mM indicating that 50% of surface-bound **2** was hydrolysed to reveal surface-bound **6**. After 4.1 h, the concentration of nanoparticle-bound **2** was found to be 1.63 mM while hydrazone **8** reached 3.25 mM (67% conversion). After 5.8 h, ^{19}F NMR analysis indicated hydrolysis of 79% nanoparticle-bound **2** (1.05 mM remaining) according to the concentration of hydrazone **8** observed in bulk solution (3.82 mM). At this stage, to arrest the hydrolysis of surface-bound **2** and prevent any further changes in the monolayer composition, nanoparticles were precipitated by adding 8:1 *v*/*v* Et_2O/EtOH (20 mL). The black solid recovered was resuspended in 7:1 *v*/*v* Et_2O/EtOH (16 mL), sonicated for 10 min and recollected by centrifugation (1312 ×g rcf, 4 °C, 20 min). This operation was repeated a further 2 times. Traces of volatile solvents were removed from the purified residue under a stream of compressed air, 1 mL water added, and the sample freeze dried to provide AuNP-$2_{0.2}6_{0.8}$ (9.72 mg) free from unbound species. Pleasingly, the ratio for surface-bound **2** and **6** calculated by area deconvolution of either the sharp signal for hydrazone **8** in bulk solution or the broad signal for surface-bound **2**, measured before nanoparticle purification (**Figure S29d** and **Table S13**), agree with the monolayer composition assessed after nanoparticle purification (**Figure S31c** and **TableS15**).

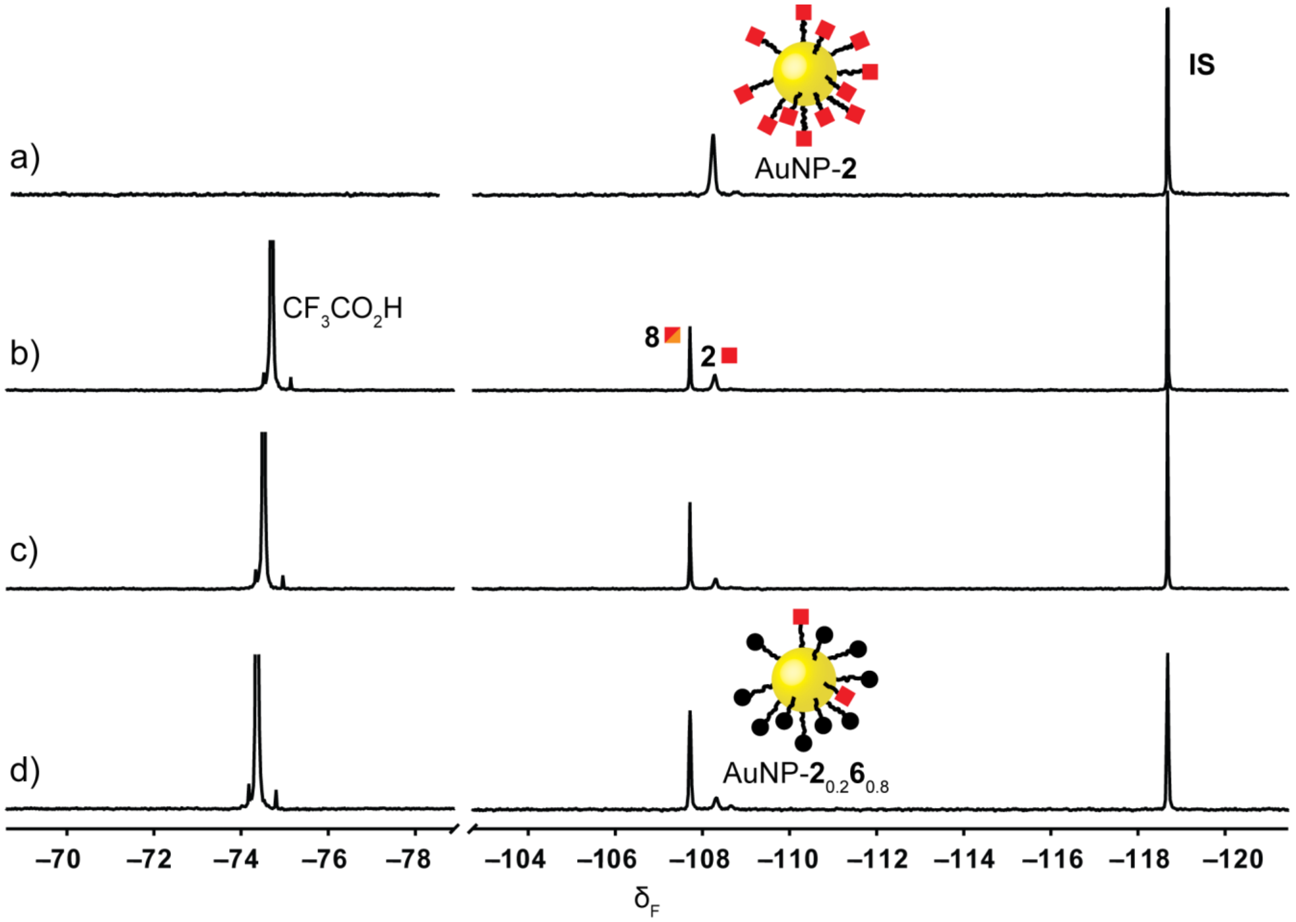

Figure S29. In situ monitoring of dynamic covalent exchange from AuNP-**2** to AuNP-$\mathbf{2}_{0.2}\mathbf{6}_{0.8}$ by ^{19}F NMR (470 MHz, 9:1 *v*/*v* DMF/D_2O, D1: 25 s): a) AuNP-**2** (5.01 mM); b–d) Reaction mixture incubated for 2.5, 4.1 and 5.8 h after addition of CF_3CO_2H. Conditions: AuNP-**2** (4.85 mM), aldehyde **7** (244 mM, 50 Eq.), CF_3CO_2H (20.5 mM), 45 °C, 5.8 h. After this time, hydrolysis of surface-bound **2** was arrested by promoting nanoparticle precipitation. Generation of AuNP-$\mathbf{2}_{0.2}\mathbf{6}_{0.8}$ is confirmed by formation of hydrazone **8** in bulk solution and corresponding reduction in concentration of nanoparticle-bound **2**. IS: internal standard (4-fluorotoluene, 5.00 mM). For concentrations of all species, see **Table S13**.

Table S13. Concentrations of fluorine-containing species during dynamic covalent exchange from AuNP-**2** to AuNP-$\mathbf{2}_{0.2}\mathbf{6}_{0.8}$

Spectrum **Figure S29**	*t* / h	[**2**] / mM	[**8**] / mM	% hydrolysis surface-bound **2**[a]	Overall conc. / mM[b]
a)	0.00	5.01		N/A	5.01
b)	2.50	2.40	2.43	50	4.83
c)	4.07	1.63	3.25	67	4.88
d)	5.78	1.05	3.82	79	4.87

[a] Determined by in situ ^{19}F NMR (N/A = not applicable). Calculated by area deconvolution of the sharp signal for hydrazone **8** in bulk solution, before nanoparticle purification (**Figure S29**). Area deconvolution of the broad nanoparticle signal for surface-bound **2** before nanoparticle purification provided identical results.
[b] Concentration of CF_3CO_2H not inlcuded.

Generation of AuNP-$2_{0.25}6_{0.75}$ by equilibrium control

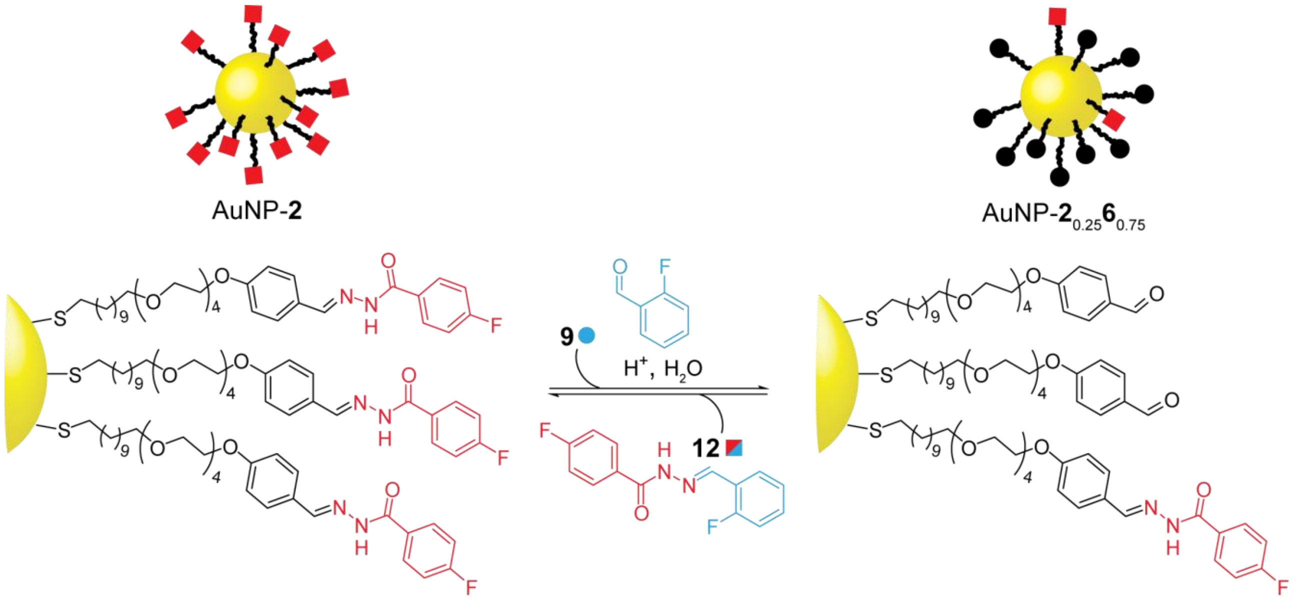

Concentrations of all fluorine-containing species were determined by quantitative ^{19}F NMR in the presence of 4-fluorotoluene as an internal standard of known concentration.

A stock solution of CF_3CO_2H was prepared in 9:1 *v*/*v* DMF/D_2O with 4-fluorotoluene as internal standard (5.00 mM) and concentration determined by ^{19}F NMR.

A colloidal solution of AuNP-**2** (6.60 mg) containing 4-fluorotoluene (5.00 mM) was prepared in 9:1 *v*/*v* DMF/D_2O (650 μL) giving 5.04 mM in terms of surface-bound hydrazone **2**. Then, aldehyde **9** (0.35 μL, 0.41 mg, 3.28 μmol) was added, followed by an aliquot of the CF_3CO_2H stock solution (12.7 μL, 12.9 μmol), giving final concentrations of AuNP-**2** (4.94 mM), **4** (4.95 mM, 1 Eq.), and CF_3CO_2H (20.0 mM). This mixture was incubated at rt and the reaction was followed by ^{19}F NMR (**Figure S30** and **Table S14**). The first spectrum recorded 3.85 h after addition of CF_3CO_2H revealed a decrease of the broad signal at –108.86 ppm corresponding to nanoparticle-bound **2** and quantitative conversion to two sharp signals at –108.00 and –121.14 ppm for hydrazone **12** in bulk solution. At this stage, the concentration measured for hydrazone **12** was 1.15 mM while free aldehyde **9** in bulk solution and nanoparticle-bound-**2** were found to be 3.80 mM and 3.78 mM respectively, indicating that 23% of surface-bound **2** had been hydrolysed (**Figure S30b** and **Table S14**). After 20.6 h, the concentration of nanoparticle-bound **2** and aldehyde **9** in bulk solution were both found to be 2.14 mM while hydrazone **12** reached 2.79 mM (56% conversion). After 59.0 h, ^{19}F NMR analysis indicated 73% hydrolysis of nanoparticle-bound **2** (1.30 mM) according to the concentration of aldehyde **9** (1.29 mM) and hydrazone **12** observed in bulk solution (3.64 mM). The reaction reached equilibrium after 110 h. At this stage, ^{19}F NMR analysis revealed that 75% of nanoparticle-bound **2** was hydrolysed, producing mixed-monolayer nanoparticles bearing a 1:3 ratio of surface-bound **2** and **6** (AuNP-$2_{0.25}6_{0.75}$, **Figure S30e** and **Table S14**). Nanoparticle precipitation was achieved by addition of 10:2:1 *v*/*v* Et_2O/EtOH/H_2O (10 mL), followed by sonication (10 min, 20 °C), and centrifugation 1935 ×g rcf, 10 min, 4 °C). The black solid recovered was then washed with Et_2O/EtOH, using the same procedure described above. Traces of volatile solvents were removed from the purified residue under a stream of compressed air, 1 mL water added, and the sample freeze dried to provide AuNP-$2_{0.25}6_{0.75}$ (5.92 mg). Pleasingly, the ratio for surface-bound **2** and **6** calculated by area deconvolution of either the sharp signal for hydrazone **12** in bulk solution or broad signal for surface-bound **2**, measured before nanoparticle purification (**Figure S30e** and **Table S14**), agree with the monolayer composition assessed after nanoparticle purification (**Figure S31b** and **Table S15**).

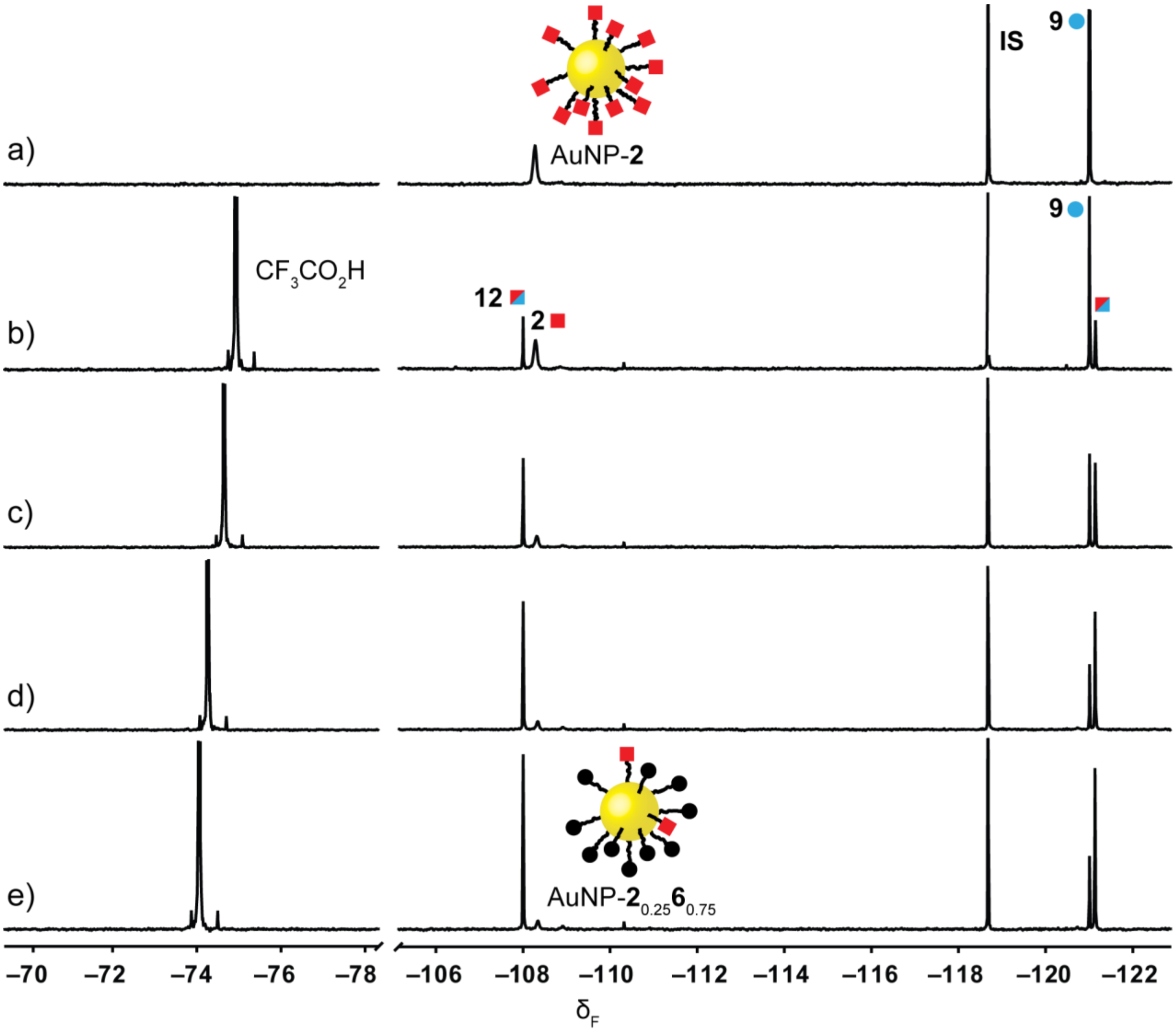

Figure S30. In situ monitoring of dynamic covalent exchange from AuNP-**2** to AuNP-$\mathbf{2}_{0.25}\mathbf{6}_{0.75}$ by ^{19}F NMR (470 MHz, 9:1 *v*/*v* DMF/D_2O, D1: 25 s): a) AuNP-**2** (5.04 mM), aldehyde **9** (5.04 mM); b–d) Reaction mixture incubated for 3.85, 20.6, 59.0 and 101 h after addition of CF_3CO_2H. Conditions: AuNP-**2** (4.94 mM), aldehyde **9** (4.95 mM, 1 Eq.), CF_3CO_2H (20.0 mM), rt, 101 h. Generation of AuNP-$\mathbf{2}_{0.25}\mathbf{6}_{0.75}$ monolayer is confirmed by formation of hydrazone **12** in bulk solution and corresponding reduction in concentration of nanoparticle-bound **2** and aldehyde **9**. IS: internal standard (4-fluorotoluene, 5.00 mM). For concentrations of all species, see **Table S14**.

Table S14. Concentrations of fluorine-containing species during dynamic covalent exchange from AuNP-**2** to AuNP-$\mathbf{2}_{0.25}\mathbf{6}_{0.75}$

Spectrum **Figure S30**	*t* / h	[**2**] / mM	[**8**] / mM	% hydrolysis surface-bound **2**[a]	Overall conc. / mM[b]
a)	0.00	5.04	5.04	0	N/A
b)	3.85	3.78	3.80	1.15	23
c)	20.6	2.14	2.14	2.79	56
d)	59.0	1.30	1.29	3.64	73
e)	101	1.20	1.19	3.72	75

[a] Determined by in situ ^{19}F NMR (N/A = not applicable). Calculated by area deconvolution of the sharp signal for hydrazone **12** in bulk solution, before nanoparticle purification.
[b] Concentration of CF_3CO_2H not inlcuded.

Summary of two-component hydrazone/aldehyde monolayers AuNP-2_x6_y

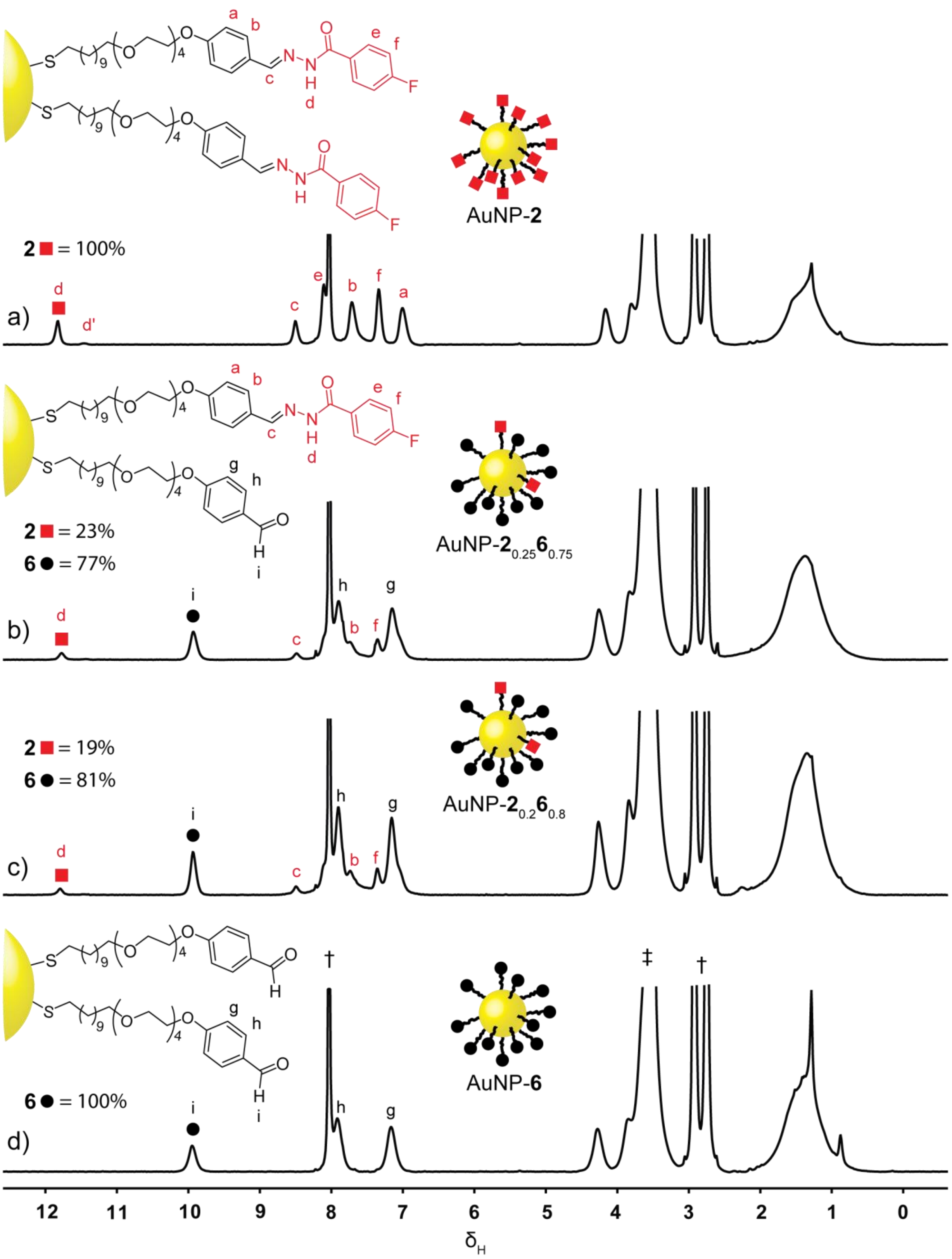

Figure S31. a) ^{1}H NMR (500 MHz, [D$_7$]DMF) spectrum recorded on purified AuNP-**2** obtained by direct synthesis; b) Quantitative ^{1}H NMR (500 MHz, [D$_7$]DMF, D1 = 30) spectrum recorded on purified AuNP-$\mathbf{2}_{0.25}\mathbf{6}_{0.75}$ obtained by equilibrium controlled hydrazone exchange. c) Quantitative ^{1}H NMR (500 MHz, [D$_7$]DMF, D1 = 30) spectrum recorded on purified AuNP-$\mathbf{2}_{0.2}\mathbf{6}_{0.8}$ obtained by kinetically arrested hydrazone exchange. d) ^{1}H NMR (500 MHz, [D$_7$]DMF) spectrum recorded on purified AuNP-**6** obtained by exhaustive hydrazone exchange. All sharp signals can be assigned to residual non-deuterated solvents as indicated († = DMF, ‡ = H_2O). For concentrations of all species, see **Table S15**.

Table S15. Summary of nanoparticle samples produced by hydrazone exchange AuNP-**2** → AuNP-$\mathbf{2}_x\mathbf{6}_y$, showing close agreement between monolayer compositions determined pre- and post-purification.

Sample	% hydrolysis surface-bound **2**[a]	During exchange[b]		Post purification[c]	
		χ_2	χ_6	χ_2	χ_6
AuNP-**2**	N/A	N/A	N/A	100	0
AuNP-$\mathbf{2}_{0.25}\mathbf{3}_{0.75}$	75	24	76	23	77
AuNP-$\mathbf{2}_{0.2}\mathbf{3}_{0.8}$	79	21	79	20	80
AuNP-**6**	100	< 0.03[d]	> 97	< 0.03[d]	> 97

[a] Determined by in situ ^{19}F NMR (N/A = not applicable). Calculated by area deconvolution of the sharp signal for hydrazone **8** or **12** in bulk solution, before nanoparticle purification (**Figures S25**, **S29** and **S30**).
[b] Determined by in situ ^{19}F NMR. Calculated by area deconvolution of broad nanoparticle signals corresponding to surface-bound **2**, before nanoparticle purification (**Figures S25**, **S29** and **S30**).
[c] Determined by in situ ^{1}H NMR on purified samples (area deconvolution of broad nanoparticle signals corresponding to surface-bound **2** and **6**) (**Figure S31**).
[d] Undetectable by either ^{19}F NMR (470 MHz, 9:1 *v*/*v* DMF/D_2O, 16 scans, D1 = 25 s) or ^{1}H NMR (500 MHz, [D_7]DMF, 8 scans, D1 = 30 s).

Conversion AuNP-$\mathbf{2}_{0.2}\mathbf{6}_{0.8}$ to AuNP-$\mathbf{2}_{0.2}\mathbf{3}_{0.8}$

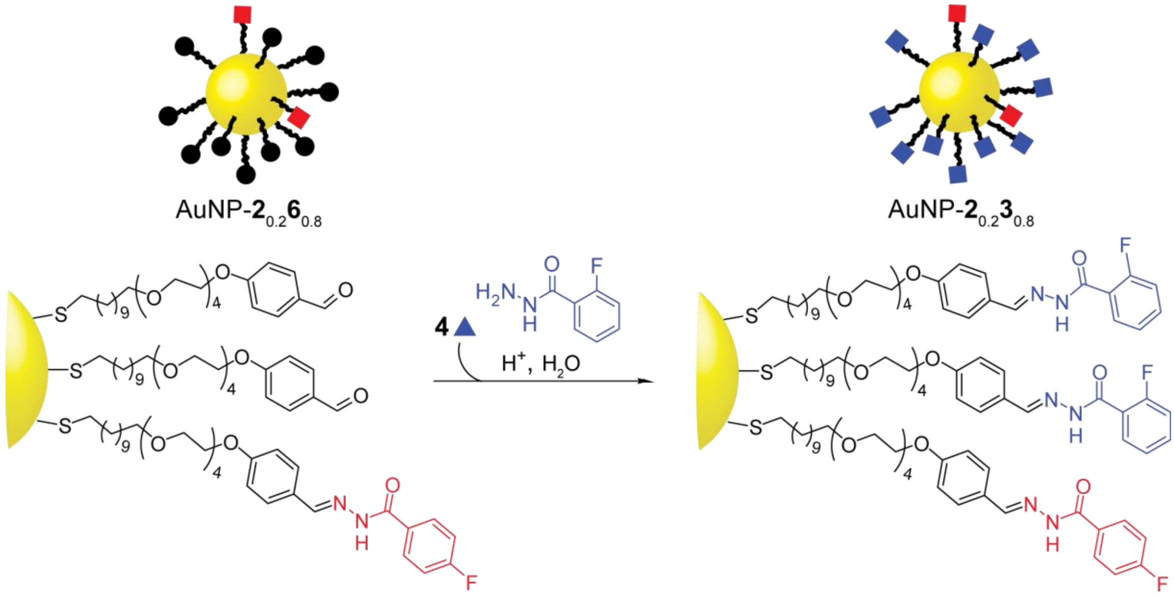

Concentrations of all fluorine-containing species were determined by quantitative ^{19}F NMR in the presence of 4-fluorotoluene as an internal standard of known concentration.

A stock solution of CF_3CO_2H was prepared in 9:1 *v*/*v* DMF/D_2O with 4-fluorotoluene as internal standard (5.00 mM) and concentration determined by ^{19}F NMR.

A colloidal solution of AuNP-$\mathbf{2}_{0.2}\mathbf{6}_{0.8}$ (6.05 mg) containing 4-fluorotoluene (5.00 mM) was prepared in 9:1 *v*/*v* DMF/D_2O (620 μL) giving 0.96 mM in terms of surface-bound hydrazone **2** and 3.86 mM in terms of surface-bound aldehyde **6** (surface-bound [**2**+**6**] = 4.82 mM). Then, an equimolar quantity of hydrazide **4** against surface-bound **6** (0.38 mg, 2.53 μmol) was added, followed by an aliquot of the CF_3CO_2H stock solution (10 μL, 12.7 μmol), giving final concentrations of AuNP-$\mathbf{2}_{0.2}\mathbf{6}_{0.8}$ (surface-bound [**2**+**6**] = 4.76 mM, [**2**] = 0.95 mM, [**6**] = 3.81 mM), **4** (3.98 mM, 1.1 Eq. against surface-bound **6**), and CF_3CO_2H (20.1 mM). This mixture was incubated at rt and the reaction was followed by ^{19}F NMR. (**Figure S32** and **Table S16**). The first spectrum recorded 0.33 h after addition of CF_3CO_2H revealed a new set of broad signals at –112.40 and –113.75 ppm corresponding to nanoparticle-bound **3**, in concomitance with a quantitative decrease of the signal at –112.54 ppm for hydrazide **4** in bulk solution. At this stage, a new sharp signal at –106.89 ppm corresponding to hydrazide **5**

released in bulk solution from the nanoparticle-bound monolayer was also detected (0.26 mM, 27% of initial surface-bound **2**) (**Figure S32b**). This observation indicates that two pathways are involved in the conversion of AuNP-$\mathbf{2}_{0.2}\mathbf{6}_{0.8}$ to AuNP-$\mathbf{2}_{0.2}\mathbf{3}_{0.8}$. Hydrazide **4** can condense with surface-bound aldehyde **6** to produce nanoparticle-bound **3**, but can also react with surface-bound hydrazone **2** to give surface-bound **3** via transimination, releasing hydrazide **5** into solution. Spectra recorded 0.90 h and 1.97 h after addition of CF_3CO_2H indicated a progressive increase in the concentration of nanoparticle-bound **3** (3.29 and 3.68 mM, respectively) (**Figure S32c** and **d**). As there was only an equimolar quantity of hydrazide present, as the system evolved towards equilibrium the concentration of released hydrazide **5** in bulk solution was observed to fall again, restoring the initial concentration of nanoparticle-bound **2**. ^{19}F NMR analysis performed 4.35 h after addition of acid catalyst indicated 100% conversion of NP-bound aldehyde **6** to hydrazone **3**, almost complete consumption of hydrazides **4** and **5**, and negligible change in the concentration of surface-bound **2** (**Figure S32e**). No further changes were observed after a further 4 h at rt (**Figure S32f** and **Table S16**). Nanoparticle precipitation was achieved by addition of 10:2:1 *v*/*v* Et_2O/EtOH/H_2O (10 mL), followed by sonication (10 min, 20 °C), and centrifugation 1935 ×g rcf, 10 min, 4 °C). The black solid recovered was then washed with Et_2O/EtOH, using the same procedure described above. Traces of volatile solvents were removed from the purified residue under a stream of compressed air, 1 mL water added, and the sample freeze dried to provide AuNP-$\mathbf{2}_{0.2}\mathbf{3}_{0.8}$ (5.77 mg). Pleasingly, the ratio for surface-bound **2** and **3** calculated by area deconvolution of either the broad signals for surface-bound **2** and **3**, before nanoparticle purification (**Figure S32f Table S16**), agree with the monolayer composition assessed after nanoparticle purification (**Figures S33, S34** and **Table S17**).

Table S16. Concentrations of fluorine-containing species during dynamic covalent exchange from AuNP-$\mathbf{2}_{0.2}\mathbf{6}_{0.8}$ to AuNP-$\mathbf{2}_{0.2}\mathbf{3}_{0.8}$

Spectrum **Figure S32**	*t* / h	[**2**] / mM	[**3**] / mM	[**4**] / mM	[**5**] / mM	% surface-bound **2**[a]	% surface-bound **3**[a]	% surface-bound **6**	Overall conc. / mM[b]
a)	0.00	0.96		4.05		20	N/A	80	5.01
b)	0.33	0.71	2.07	1.84	0.26	15	44	41	4.88
c)	0.90	0.78	3.29	0.63	0.23	16	69	14	4.94
d)	1.97	0.88	3.68	0.25	0.12	18	78	4	4.92
e)	4.35	0.92	3.83	0.14	0.07	19	81		4.95
f)	8.25	0.92	3.85	0.13	0.06	19	81		4.96

[a] Determined by in situ ^{19}F NMR (N/A = not applicable). Calculated by area deconvolution of either of the broad signals for surface-bound **2** and **3**, before nanoparticle purification (**Figure S32**).
[b] Concentration of CF_3CO_2H not inlcuded.

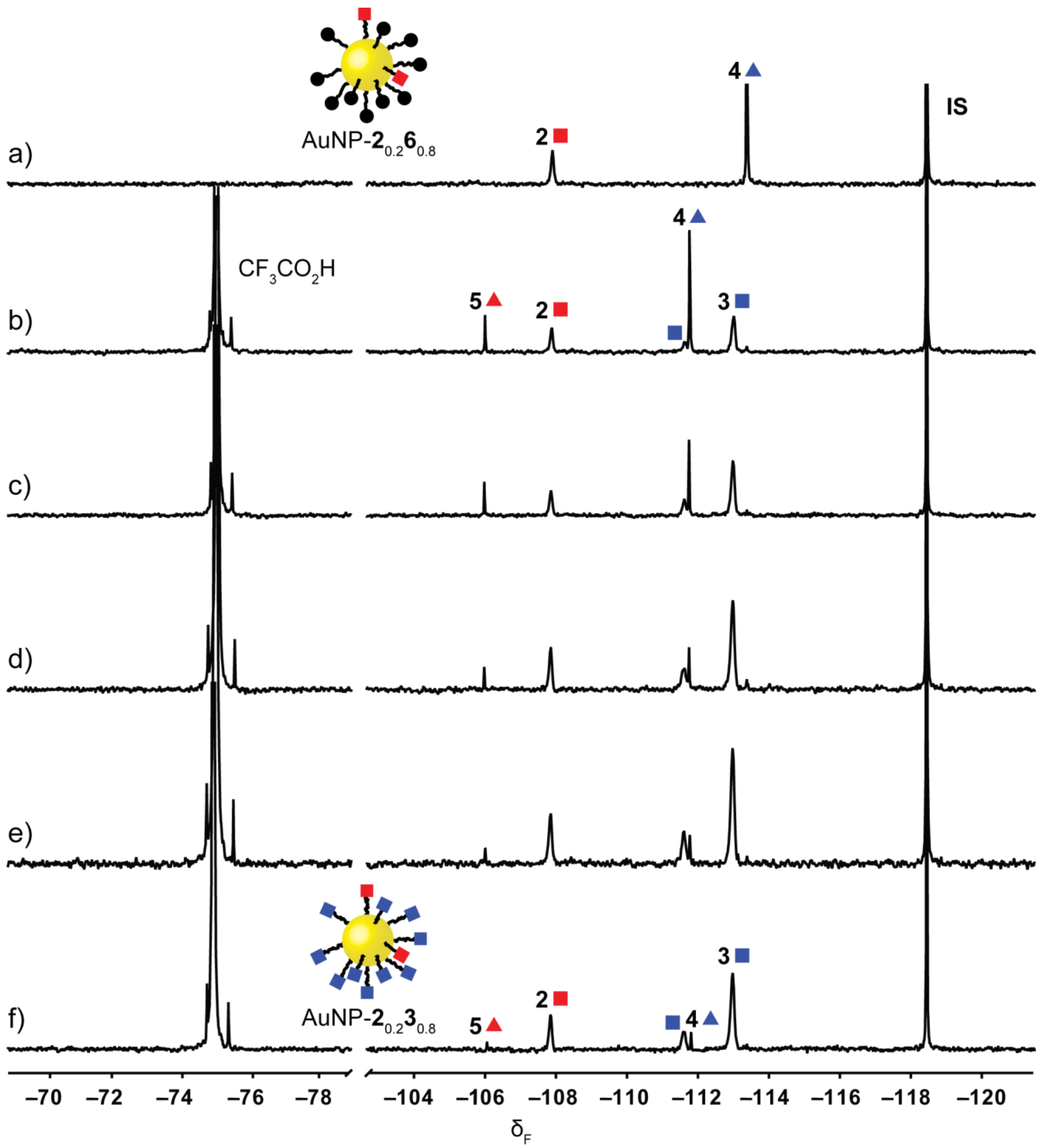

Figure S32. In situ monitoring of dynamic covalent exchange from AuNP-$\mathbf{2}_{0.2}\mathbf{6}_{0.8}$ to AuNP-$\mathbf{2}_{0.2}\mathbf{3}_{0.8}$ by ^{19}F NMR (470 MHz, 9:1 *v*/*v* DMF/D_2O, D1: 25 s): a) AuNP-$\mathbf{2}_{0.2}\mathbf{6}_{0.8}$ (surface-bound [**2**+**6**] = 4.82 mM, [**2**] = 0.96 mM, [**6**] = 3.86mM), hydrazide **4** (4.05 mM); b–f) Reaction mixture incubated for 0.33, 0.90, 1.97, 4.35 and 8.25 h after addition of CF_3CO_2H. Conditions: AuNP-$\mathbf{2}_{0.2}\mathbf{6}_{0.8}$ (surface-bound [**2**+**6**] = 4.76 mM, [**2**] = 0.95 mM, [**6**] = 3.81 mM), hydrazide **4** (3.98 mM, 1.1 Eq. against surface-bound **6**), CF_3CO_2H (20.1 mM), rt, 8.25 h. Generation of AuNP-$\mathbf{2}_{0.2}\mathbf{3}_{0.8}$ monolayer is confirmed by formation of nanoparticle-bound **3** and corresponding reduction in concentration of hydrazide **4** in bulk solution. IS: internal standard (4-fluorotoluene, 5.00 mM). For concentrations of all species, see **Table S16**.

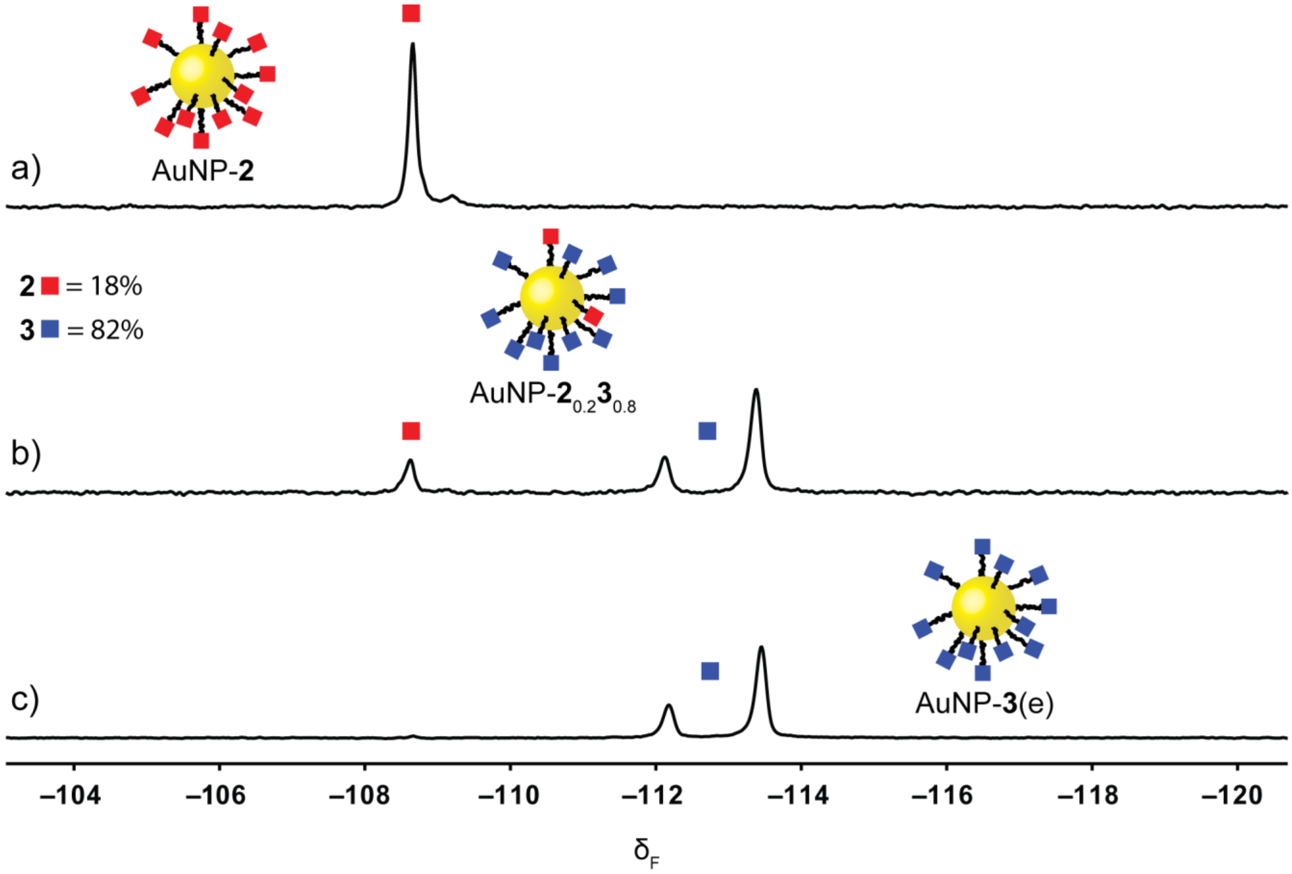

Figure S33. ^{19}F NMR (470 MHz, [D7]DMF, D1: 25 s) characterization of purified AuNP-**2**$_{0.2}$**3**$_{0.8}$ prepared by dynamic covalent exchange from AuNP-**2**$_{0.2}$**6**$_{0.8}$: a) AuNP-**2**; b) AuNP-**2**$_{0.2}$**3**$_{0.8}$; c) AuNP-**3**(e)**.**

Table S17. Summary of characterization data obtained for AuNP-**2**$_{0.2}$**3**$_{0.8}$ produced by dynamic covalent exchange from AuNP-**2**$_{0.2}$**6**$_{0.8}$, showing close agreement between monolayer compositions determined in situ pre- and post-purification, and ex situ following oxidative ligand desorption.

Sample	During exchange[a]		Post purification[b]		Oxidative ligand desorption[c]	
	χ_2	χ_3	χ_2	χ_3	χ_2	χ_3
AuNP-**2**$_{0.2}$**3**$_{0.8}$	19	81	18	82	19	81

[a] Determined by in situ ^{19}F NMR. Calculated by area deconvolution of broad nanoparticle signals corresponding to surface-bound **2** and **3** (**Figure S32** and **Table S16**).
[b] Determined by in situ ^{19}F NMR on purified sample. Calculated by area deconvolution of broad nanoparticle signals corresponding to surface-bound **2** and **3** (**Figure S33b**).
[c] Determined ex situ following oxidative ligand desorption using I_2 (**Figure S34** and **Table S18**).

Ex situ NMR Characterization of AuNP-$2_{0.2}3_{0.8}$: oxidative ligand desorption

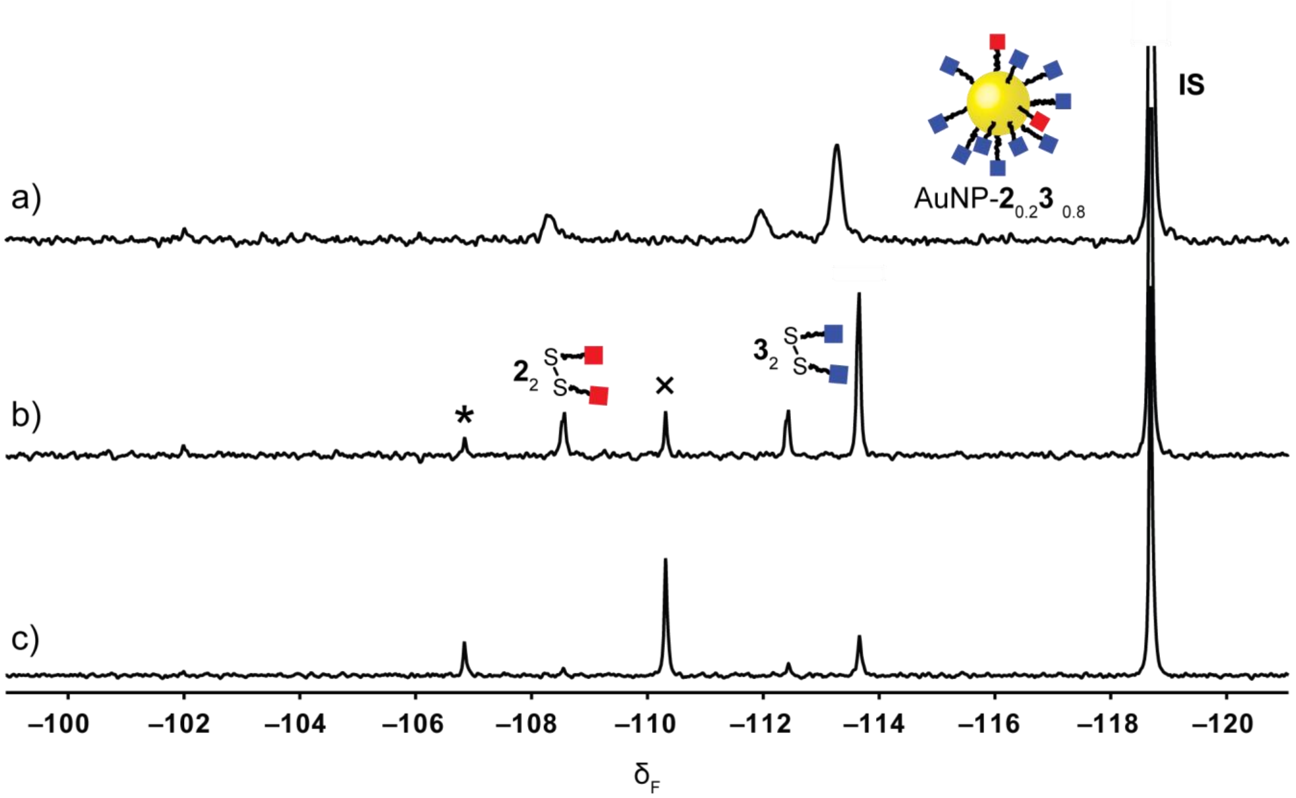

Figure S34. Oxidative ligand desorption performed on a solution of purified AuNP-$\mathbf{2}_{0.2}\mathbf{3}_{0.8}$: a) ^{19}F NMR (470 MHz, 9:1 *v*/*v* DMF/D_2O, D1: 25 s) AuNP-$\mathbf{2}_{0.2}\mathbf{3}_{0.8}$. Nanoparticle-bound [**2**+**3**] = 2.94 mM (internal standard 5 mM 4-fluorotoluene, δ_F –118.68 ppm); b–c) ^{19}F NMR (470 MHz, 9:1 *v*/*v* DMF/D_2O, D1: 25 s) spectra recorded 53 and 249 min after addition of iodine to a solution of AuNP-$\mathbf{2}_{0.2}\mathbf{3}_{0.8}$. Total concentration of all fluorinated species remained constant during the experiment time-course and in close agreement with on-nanoparticle concentration measured at t = 0 min (**Table S18**).

Table S18. Concentrations of fluorine-containing species during oxidative ligand desorption from AuNP-$\mathbf{2}_{0.2}\mathbf{3}_{0.8}$.

Spectrum **Figure S34**	*t* / h	[AuNP-$\mathbf{2}_{0.2}\mathbf{3}_{0.8}$] / mM	[$\mathbf{2}_2$] / mM	[**5**] / mM[a]	[$\mathbf{3}_2$] / mM	[**4**] / mM[b]	Overall conc./ mM
a)	0	2.94					2.94
b)	0.88		0.46	0.13	2.02	0.31	2.92
c)	4.15		0.10	0.50	0.74	1.56	2.90

[a] Including the concentration of 4-fluorobenzoic acid.
[b] Including the concentration of 2-fluorobenzoic acid.

7.3 Stability tests on nanoparticle-bound aldehydes

A colloidal solution of purified AuNP-**6** (5.19 mg) was prepared in $CDCl_3$ (550 µL). The stability of nanoparticle-bound aldehydes was investigated by ageing at 45 °C for 89 h. No formation of surface-bound carboxylic acids, nor other degradation products or free ligands were detected by in situ ^1H NMR (**Figure S35**).

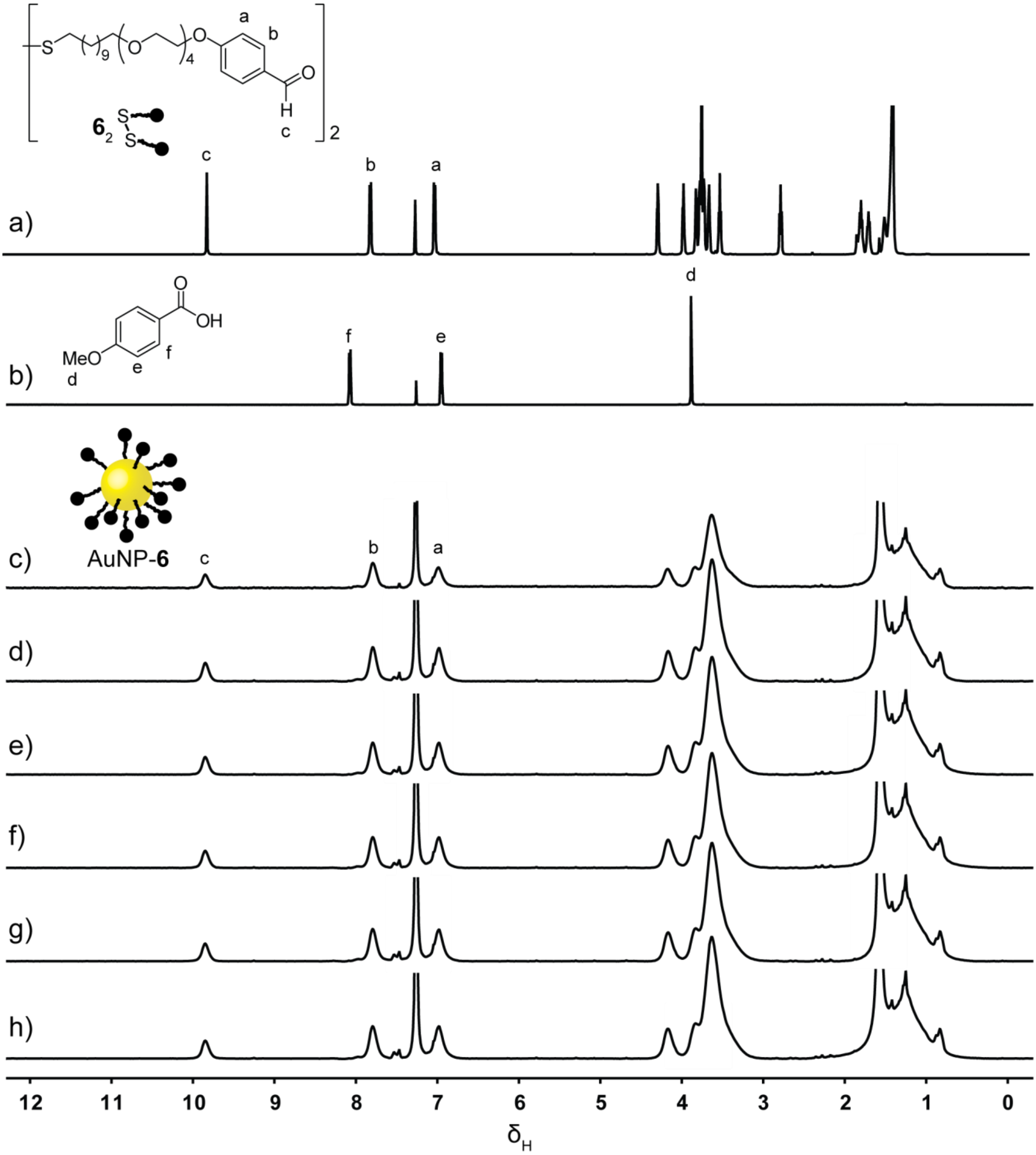

Figure S35. Stability monitoring of AuNP-**6** surface-bound aldehydes by in situ ^1H NMR spectroscopy: a) ^1H NMR (500 MHz, $CDCl_3$) spectrum of disulfide $\mathbf{6}_2$; b) ^1H NMR (500 MHz, $CDCl_3$) spectrum of 4-methoxybenzoic acid; c) Quantitative ^1H NMR (500 MHz, $CDCl_3$, D1: 30 s) spectrum of AuNP-**6** recorded before incubating the sample at 45 °C; d–h) Quantitative ^1H NMR (500 MHz, $CDCl_3$, D1: 30 s) spectra recorded 8.0, 21, 44, 65 and 89 h after incubating AuNP-**6** at 45 °C. No evidence of surface-bound carboxylic acids, nor degradation products or free ligands were detected.

To test the stability of nanoparticle-bound aldehydes under conditions of hydrazone exchange, a sample of nanoparticles bearing a mixed monolayer of aldehydes and hydrazones (AuNP-$\mathbf{2}_{0.2}\mathbf{6}_{0.8}$, 5.10 mg) was incubated with 20 mM CF_3CO_2H in [D_7]DMF (520 uL) at 45 °C for 116 h. Pleasingly, no change to the monolayer constitution, nor appearance of degradation products or free ligands was observed by in situ ^{1}H NMR (**Figure S36**). Furthermore, close agreement was always observed for monolayer compositions determined over time by area deconvolution of broad nanoparticle signals corresponding to surface-bound **2** and **6** (**Table S19**).

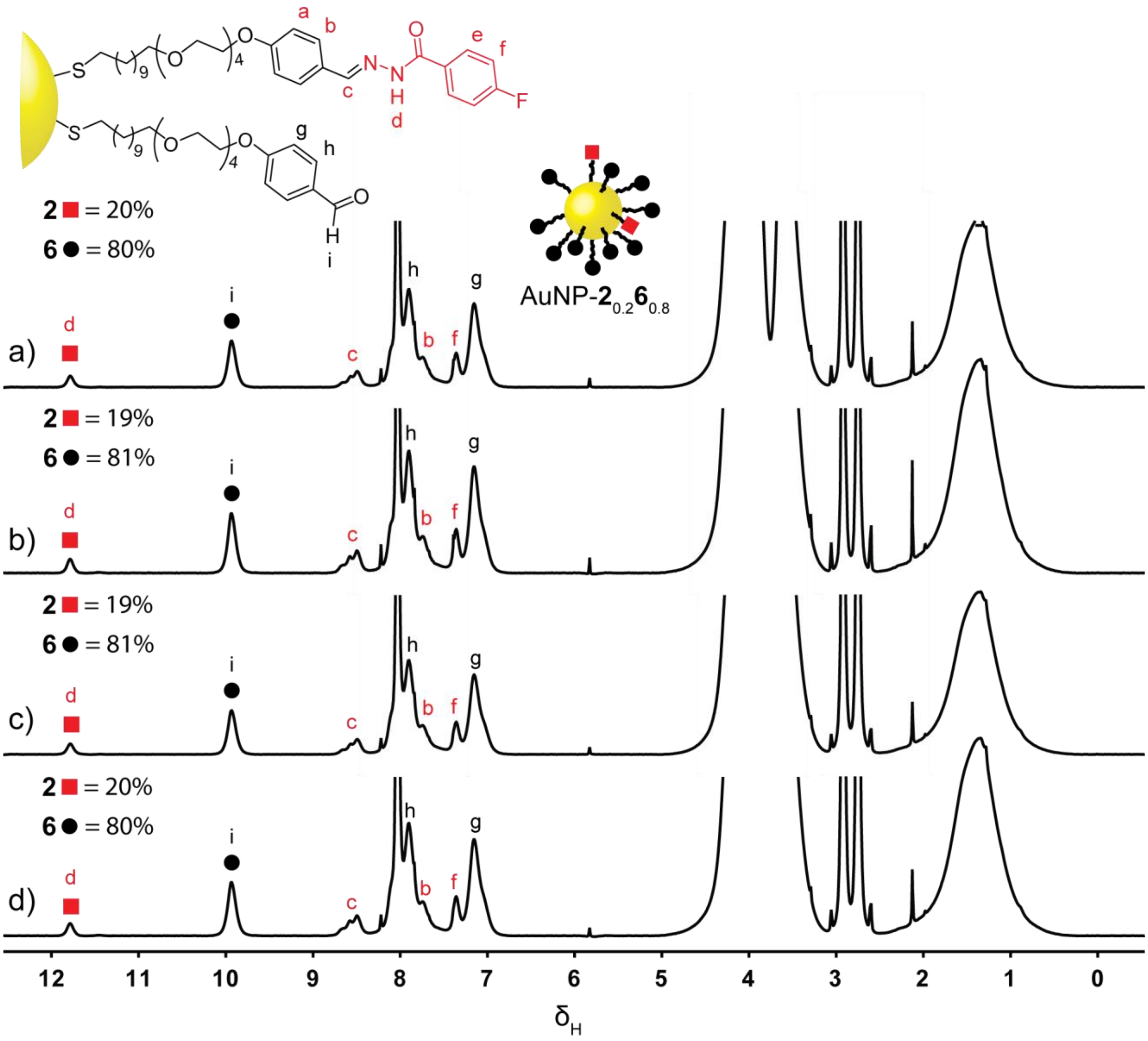

Figure S36. Stability monitoring of AuNP-$\mathbf{2}_{0.2}\mathbf{6}_{0.8}$ surface-bound aldehydes under hydrazone exchange conditions by in situ ^{1}H NMR spectroscopy. Quantitative ^{1}H NMR (500 MHz, [D_7]DMF, D1: 30 s) spectrum of AuNP-**6** recorded before incubating the sample with CF_3CO_2H (20 mM) in [D_7]DMF at 45 °C; b–d) Quantitative ^{1}H NMR (500 MHz, [D_7]DMF, D1: 30 s) spectra recorded 21.0, 92.0, and 116 h after incubating AuNP-$\mathbf{2}_{0.2}\mathbf{6}_{0.8}$ with CF_3CO_2H (20 mM) at 45 °C. No evidence of surface-bound carboxylic acids, nor degradation products or free ligands were detected. Furthermore, close agreement was observed for monolayer compositions determined over time by area deconvolution of broad nanoparticle signals corresponding to surface-bound **2** and **6** (**Table S19**).

Table S19. AuNP-$\mathbf{2}_{0.2}\mathbf{6}_{0.8}$ monolayer compositions determined over time by area deconvolution of broad nanoparticle signals corresponding to surface-bound **2** and **6**, while incubating the sample with 20 mM CF_3CO_2H in [D_7]DMF at 45 °C.

Spectrum **Figure S36**	t / h	Monolayer composition[a] χ_2	Monolayer composition[a] χ_6
a)	0.00	20	80
b)	21.0	19	81
c)	92.0	19	81
d)	116	20	80

[a] Determined by in situ ^{1}H NMR. Calculated by area deconvolution of broad nanoparticle signals corresponding to surface-bound **2** and **3** (**Figure S36**).

8. Kinetic studies of dynamic covalent reactions

8.1 Hydrazone exchange

All hydrazone exchange reactions **R1**, **R2**, **R3** and **R4** were performed at a concentration of ca. 5 mM in starting hydrazone in the presence of a molecular modifier at equimolar concentration, excess acid catalyst (20 mM CF_3CO_2H), and large excess of water (10% *v*/*v* ≈ 1000 equivalents) in DMF. Stock solutions of model compounds or nanoparticles were prepared in 9:1 *v*/*v* DMF/D_2O. Concentrations of each fluorinated species were assessed by ^{19}F NMR relative to 4-fluorotoluene as internal standard, which was added at a known concentration (ca. 5 mM). Then an equimolar amount of molecular modifier (aldehyde or hydrazide) was added and the reaction triggered by addition of CF_3CO_2H. The NMR tube was held at rt and occasionally agitated while recording NMR spectra at intermediate time points to track reaction progress. Each exchange was monitored until equilibrium was reached. The total concentration of fluorinated species measured remained constant throughout each experiment time-course and was always in agreement with the starting amount of hydrazone and exchange unit. Moreover, no additional resonances were observed in the ^{19}F NMR spectra recorded at later time points, indicating the absence of significant side reactions or decomposition processes. Three experimental replicates were performed for each reaction.

Reaction **R4** has only been studied in bulk solution because reacting AuNP-**1** with nucleophilic exchangeable units produces nanoparticles with a significant number of surface-bound free hydrazides, leading to aggregation and precipitation processes. Reaction **R3** has also been investigated in dry [D_7]DMF (**R3**(anhydr.)) and in 9:1 *v*/*v* DMF/D_2O with a reduced concentration of CF_3CO_2H (5 mM **R3**(low [H^+]).

Each reaction was modelled as a reversible process characterised by two bimolecular rate constants, k_{fwd} and k_{rev}, for the forward and reverse reactions, respectively. This simple model assumes all intermediates to be present only at low 'steady state' concentrations, while water (when added) and acid catalyst are present in a constant excess. To estimate the kinetic parameters, non-linear fits were performed using concentration data for all fluorinated species. In order to determine initial rate constants ($k_{init.}$), data points during the linear portion of the reaction profile were fit to an irreversible bimolecular model. Rate constants were independently determined for each experimental replicate then averaged to give the results summarised in **Table S20** (**R1**, **R2**) and **Table S21** (**R3**, R3(anhydr.), **R3**(low[H^+])). **Tables S22-S32** report all results for each experimental replicate. For reactions **R3**(anhydr.) and **R3**(low [H^+]), which were only run once, a conservative estimate of uncertainties in the rate constants of 10% was chosen. Full time-course plots for a representative example of each experiment are shown in **Figure 5** (**R1**, **R2**, **R3**, **R3**(anhydr.)), **Figure S37** (**R3**(low [H^+]) and **Figure S38** (**R4**).

Table S20. Comparison of rate and equilibrium constants estimated for hydrazone exchange reactions with electrophilic molecular modifiers (**R1**, **R2**) performed in bulk solution and nanoparticle-bound environments.[a]

Reaction	k_{fwd} / mM^{-1} h^{-1}	k_{rev} / mM^{-1} h^{-1}	K (k_{fwd} / k_{rev})	K_{eq} (NMR) [b]
**R1** Bulk solution	6.36 (± 0.31) × 10^{-3}	1.72 (± 0.18) × 10^{-3}	3.7	3.3
**R1** NP-bound	3.34 (± 0.03) × 10^{-3}	1.43 (± 0.12) × 10^{-3}	2.3	2.6
NP-bound inhibition [c]	**0.52**	**0.83**		
**R2** Bulk solution	17.8 (± 0.5) × 10^{-3}	1.42 (± 0.05) × 10^{-3}	12.5	11.9
**R2** NP-bound	14.8 (± 0.2) × 10^{-3}	1.58 (± 0.12) × 10^{-3}	9.4	9.5
NP-bound inhibition [c]	**0.83**	**1.11**		

[a] Values reported as the mean of triplicate experiments ± one standard deviation. Kinetic constants for each experimental replicate are reported in **Tables S22–S25**.
[b] Determined using the average of the concentrations from three data points collected at the apparent equilibrium position.
[c] Quantification of the effect of reaction environment (NP-bound versus bulk solution) on reaction kinetics. Inhibition factor = *k*(NP bound) / *k*(bulk solution).

Table S21. Comparison of rate and equilibrium constants estimated for hydrazone exchange reactions with nucleophilic molecular modifiers performed in bulk solution and nanoparticle-bound environments.

Reaction	k_{fwd} / mM^{-1} h^{-1}	k_{rev} / mM^{-1} h^{-1}	K (k_{fwd} / k_{rev})	K_{eq} (NMR) [d]
**R3** [a] Bulk solution	1.56 (± 0.13)	0.722 (± 0.066)	2.2	2.0
**R3** [a] NP-bound	0.482 (± 0.035)	0.256 (± 0.023)	1.9	1.8
NP-bound inhibition [c]	**0.31**	**0.35**		
**R3** (anhydr.) [b] Bulk solution	1.06 (± 0.11)	0.413 (± 0.041)	2.6	2.2
**R3** (anhydr.) [b] NP-bound	0.416 (± 0.041)	0.194 (± 0.019)	2.1	2.2
NP-bound inhibition [c]	**0.39**	**0.47**		
**R3** (Low [H^+]) [b] Bulk solution	1.01 (± 0.10)	0.591 (± 0.059)	1.7	1.6
**R3** (Low [H^+]) [b] NP-bound	0.345 (± 0.035)	0.195 (± 0.019)	1.8	1.7
NP-bound inhibition [c]	**0.34**	**0.33**		

[a] Values reported as the mean of triplicate experiments ± one standard deviation. Kinetic constants for each experimental replicate are reported in **Tables S26, S27**.
[b] Uncertainties estimated at (± 10%)
[c] Quantification of the effect of reaction environment (NP-bound versus bulk solution) on reaction kinetics. Inhibition factor = *k*(NP bound) / *k*(bulk solution).
[d] Determined using the average of the concentrations from three data points collected at the apparent equilibrium position.

Reaction R1

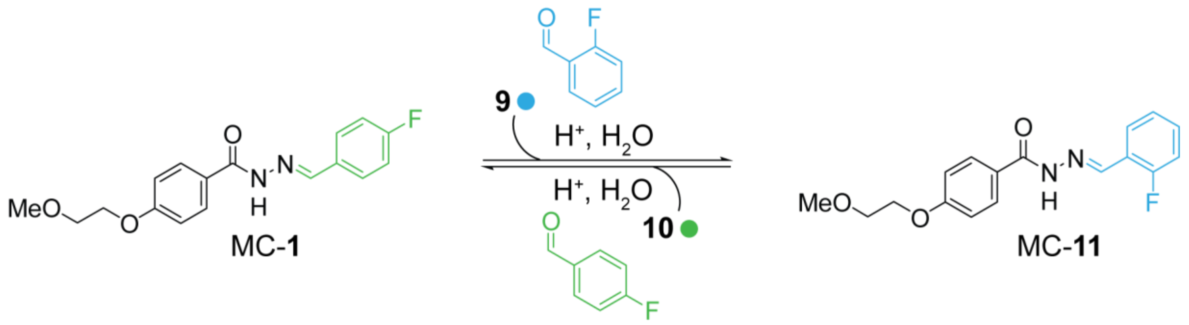

Table S22. Rate constants estimated for hydrazone exchange **R1** performed on MC-**1**.

[MC-**1**]$_0$ /mM	[**9**]$_0$ /mM	[CF_3CO_2H] / mM	k_{fwd} / $mM^{-1}h^{-1}$	k_{rev} / $mM^{-1}h^{-1}$	K (k_{fwd} / k_{rev})	K (NMR)[a]	$k_{init.}$ / $mM^{-1}h^{-1}$
4.74	4.70	20.2	6.09 x 10^{-3}	1.83 x 10^{-3}	3.33	3.01	6.30 x 10^{-3}
4.80	4.73	20.8	6.30 x 10^{-3}	1.51 x 10^{-3}	4.16	3.54	6.41 x 10^{-3}
4.76	4.77	20.8	6.70 x 10^{-3}	1.81 x 10^{-3}	3.71	3.32	6.62 x 10^{-3}
		Mean	6.36 x 10^{-3}	1.72 x 10^{-3}	3.73	3.29	6.44 x 10^{-3}
		SD	± 0.31 x 10^{-3}	± 0.18 x 10^{-3}	± 0.42	± 0.26	± 0.16 x 10^{-3}

[a] Determined using the average of the concentrations from three data points collected at the apparent equilibrium position.

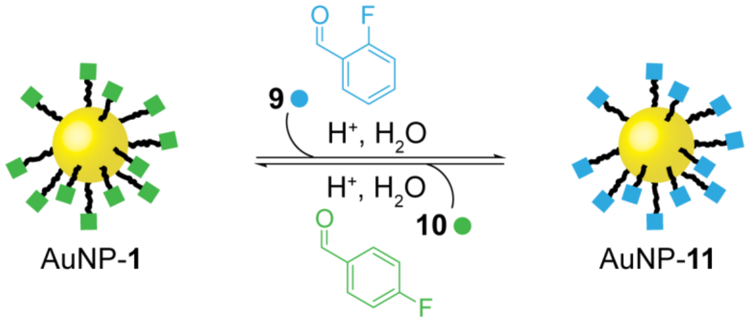

Table S23. Rate constants estimated for hydrazone exchange **R1** performed on AuNP-**1**.

[AuNP-**1**]$_0$ / mM	[**9**]$_0$ /mM	[CF_3CO_2H] / mM	k_{fwd} / $mM^{-1}h^{-1}$	k_{rev} / $mM^{-1}h^{-1}$	K (k_{fwd} / k_{rev})	K (NMR)[a]	$k_{init.}$ / $mM^{-1}h^{-1}$
4.87	4.83	20.7	3.31 x 10^{-3}	1.51 x 10^{-3}	2.19	2.56	3.21 x 10^{-3}
4.81	4.76	20.9	3.33 x 10^{-3}	1.48 x 10^{-3}	2.24	2.49	3.32 x 10^{-3}
4.66	4.68	21.0	3.37 x 10^{-3}	1.30 x 10^{-3}	2.60	2.71	3.43 x 10^{-3}
		Mean	3.34 x 10^{-3}	1.43 x 10^{-3}	2.34	2.59	3.32 x 10^{-3}
		SD	± 0.03 x 10^{-3}	± 0.12 x 10^{-3}	± 0.22	± 0.11	± 0.11 x 10^{-3}

[a] Determined using the average of the concentrations from three data points collected at the apparent equilibrium position.

Reaction R2

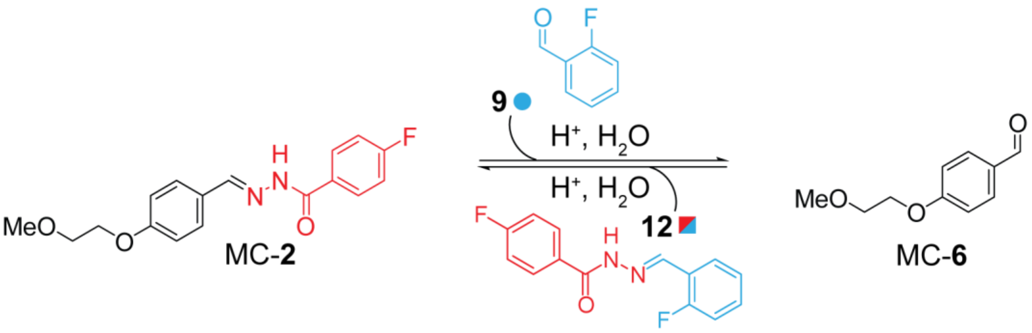

Table S24. Rate constants estimated for hydrazone exchange **R2** performed on MC-**2**.

$[\text{MC-}\mathbf{2}]_0$ /mM	$[\mathbf{9}]_0$ /mM	$[CF_3CO_2H]$ / mM	k_{fwd} / $mM^{-1}h^{-1}$	k_{rev} / $mM^{-1}h^{-1}$	K (k_{fwd} / k_{rev})	K (NMR)[a]	$k_{init.}$ / $mM^{-1}h^{-1}$
4.75	4.77	20.1	18.1×10^{-3}	1.36×10^{-3}	13.3	12.5	1.77×10^{-2}
4.75	4.78	19.9	18.0×10^{-3}	1.45×10^{-3}	12.4	11.7	1.75×10^{-2}
4.83	4.86	20.0	17.3×10^{-3}	1.45×10^{-3}	11.9	11.6	1.73×10^{-2}
		Mean	17.8×10^{-3}	1.42×10^{-3}	12.5	11.9	1.75×10^{-2}
		SD	$\pm 0.4 \times 10^{-3}$	$\pm 0.05 \times 10^{-3}$	± 0.69	± 0.50	$\pm 0.02 \times 10^{-2}$

[a] Determined using the average of the concentrations from three data points collected at the apparent equilibrium position.

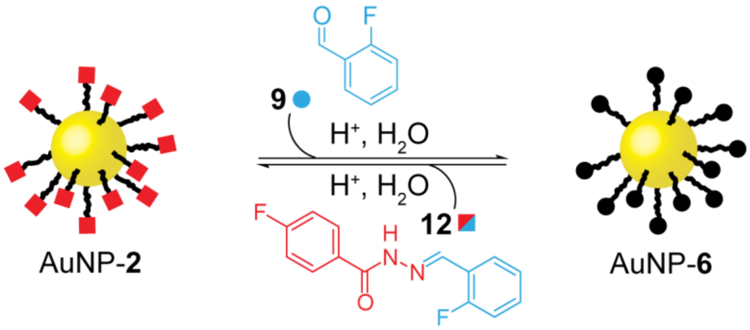

Table S25. Rate constants estimated for hydrazone exchange **R2** performed on AuNP-**2**.

$[\text{AuNP-}\mathbf{2}]_0$ /mM	$[\mathbf{9}]_0$ /mM	$[CF_3CO_2H]$ / mM	k_{fwd} / $mM^{-1}h^{-1}$	k_{rev} / $mM^{-1}h^{-1}$	K (k_{fwd} / k_{rev})	K (NMR)[a]	$k_{init.}$ / $mM^{-1}h^{-1}$
4.93	4.94	20.3	15.0×10^{-3}	1.50×10^{-3}	10.0	10.2	1.58×10^{-2}
4.94	4.95	20.0	14.8×10^{-3}	1.52×10^{-3}	9.7	9.7	1.55×10^{-2}
4.91	4.94	20.0	14.7×10^{-3}	1.71×10^{-3}	8.6	8.5	1.54×10^{-2}
		Mean	14.8×10^{-3}	1.58×10^{-3}	9.44	9.48	1.56×10^{-2}
		SD	$\pm 0.20 \times 10^{-3}$	$\pm 0.12 \times 10^{-3}$	± 0.74	± 0.86	$\pm 0.02 \times 10^{-2}$

[a] Determined using the average of the concentrations from three data points collected at the apparent equilibrium position.

Reaction R3

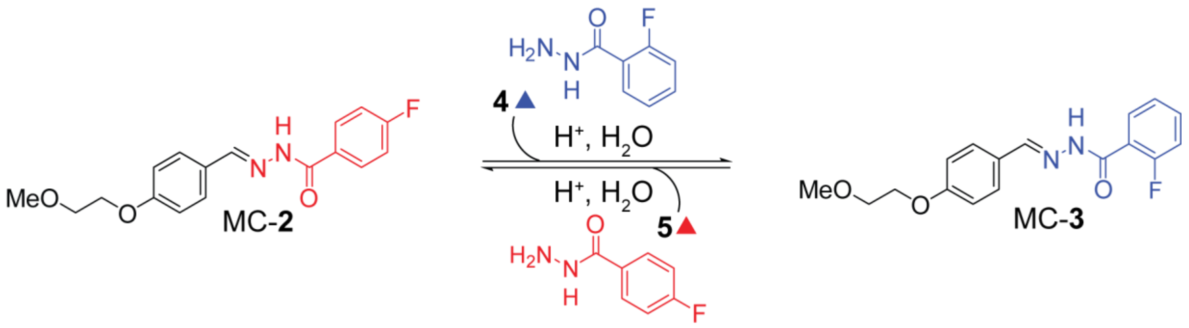

Table S26. Rate constants estimated for hydrazone exchange **R3** performed on MC-**2**.

[MC-**2**]$_0$ /mM	[**4**]$_0$ /mM	[CF_3CO_2H] / mM	k_{fwd} / $mM^{-1}h^{-1}$	k_{rev} / $mM^{-1}h^{-1}$	K (k_{fwd} / k_{rev})	K (NMR)[a]	$k_{init.}$ / $mM^{-1}h^{-1}$
4.82	4.87	20.6	1.66	0.723	2.30	2.12	1.54
4.80	4.84	20.0	1.41	0.656	2.16	1.97	1.19
4.73	4.75	19.9	1.59	0.787	2.02	1.90	1.40
		Mean	1.56	0.722	2.16	2.00	1.38
		SD	± 0.13	± 0.066	± 0.14	± 0.11	± 0.18

[a] Determined using the average of the concentrations from three data points collected at the apparent equilibrium position.

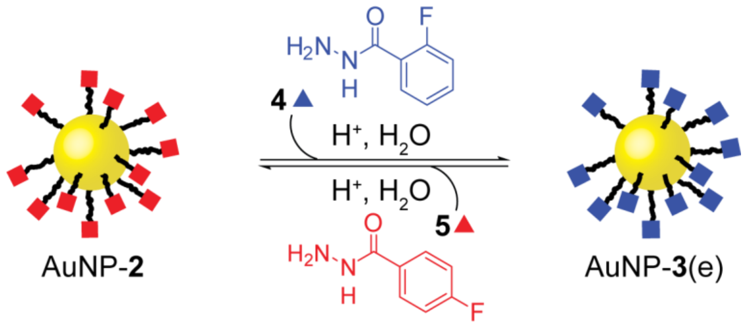

Table S27. Rate constants estimated for hydrazone exchange **R3** performed on AuNP-**2**.

[AuNP-**2**]$_0$ /mM	[**4**]$_0$ /mM	[CF_3CO_2H] / mM	k_{fwd} / $mM^{-1}h^{-1}$	k_{rev} / $mM^{-1}h^{-1}$	K (k_{fwd} / k_{rev})	K (NMR)[a]	$k_{init.}$ / $mM^{-1}h^{-1}$
4.82	4.83	20.5	0.518	0.283	1.83	1.76	0.48
4.89	4.93	20.8	0.478	0.244	1.96	1.86	0.44
4.80	4.78	20.3	0.449	0.241	1.86	1.74	0.41
		Mean	0.482	0.256	1.88	1.78	0.44
		SD	± 0.035	± 0.023	± 0.07	± 0.06	± 0.03

[a] Determined using the average of the concentrations from three data points collected at the apparent equilibrium position.

Reaction R3(anhydr.)

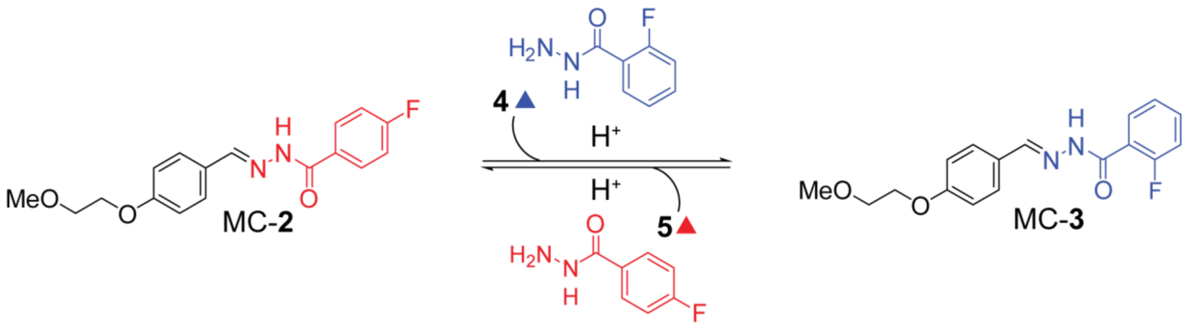

Table S28. Rate constants estimated for hydrazone exchange **R3**(anhydr.) performed on MC-**2**.

[MC-**2**]$_0$ /mM	[**4**]$_0$ /mM	[CF_3CO_2H] / mM	k_{fwd} / $mM^{-1}h^{-1}$	k_{rev} / $mM^{-1}h^{-1}$	K (k_{fwd} / k_{rev})	K (NMR)[a]	k_{init} / $mM^{-1}h^{-1}$
4.81	4.94	20.2	1.06	0.413	2.57	2.23	0.92

[a] Determined using the average of the concentrations from three data points collected at the apparent equilibrium position.

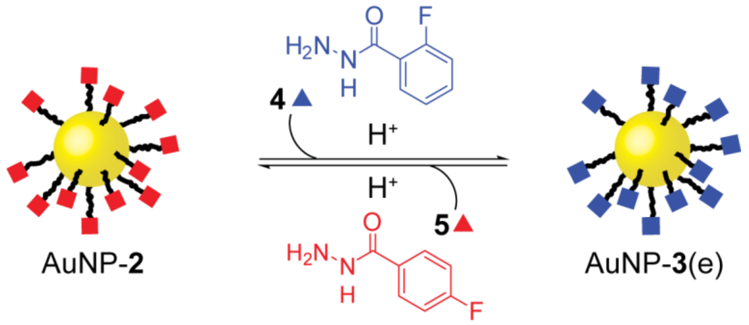

Table S29. Rate constants estimated for hydrazone exchange **R3**(anhydr.) performed on AuNP-**2**

[AuNP-**2**]$_0$ /mM	[**4**]$_0$ /mM	[CF_3CO_2H] / mM	k_{fwd} / $mM^{-1}h^{-1}$	k_{rev} / $mM^{-1}h^{-1}$	K (k_{fwd} / k_{rev})	K (NMR)[a]	k_{init} / $mM^{-1}h^{-1}$
4.89	4.93	20.2	0.416	0.194	2.14	2.20	0.40

[a] Determined using the average of the concentrations from three data points collected at the apparent equilibrium position.

Reaction R3(low [H⁺])

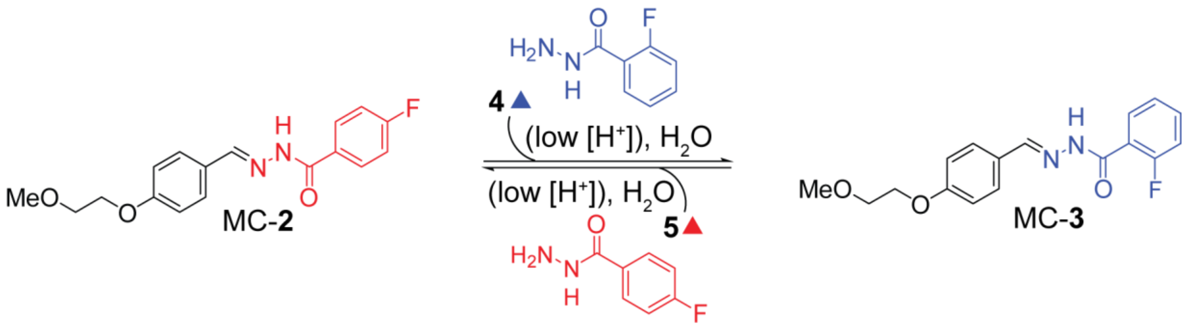

Table S30. Rate constants estimated for hydrazone exchange **R3**(low [H^+]) performed on MC-**2**

[MC-**2**]$_0$ /mM	[**4**]$_0$ /mM	[CF_3CO_2H] / mM	k_{fwd} / $mM^{-1}h^{-1}$	k_{rev} / $mM^{-1}h^{-1}$	K (k_{fwd} / k_{rev})	K (NMR)[a]	k_{init} / $mM^{-1}h^{-1}$
4.96	4.96	5.35	1.01	0.591	1.71	1.60	0.88

[a] Determined using the average of the concentrations from three data points collected at the apparent equilibrium position.

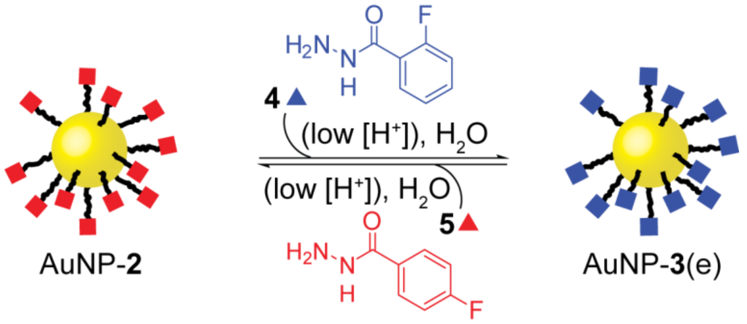

Table S31. Rate constants estimated for hydrazone exchange **R3**(low [H^+]) performed on AuNP-**2**

[AuNP-**2**]$_0$ /mM	[**4**]$_0$ /mM	[CF_3CO_2H] / mM	k_{fwd} / $mM^{-1}h^{-1}$	k_{rev} / $mM^{-1}h^{-1}$	K (k_{fwd} / k_{rev})	K (NMR)[a]	k_{init} / $mM^{-1}h^{-1}$
4.98	4.99	5.13	0.345	0.195	1.77	1.68	0.34

[a] Determined using the average of the concentrations from three data points collected at the apparent equilibrium position.

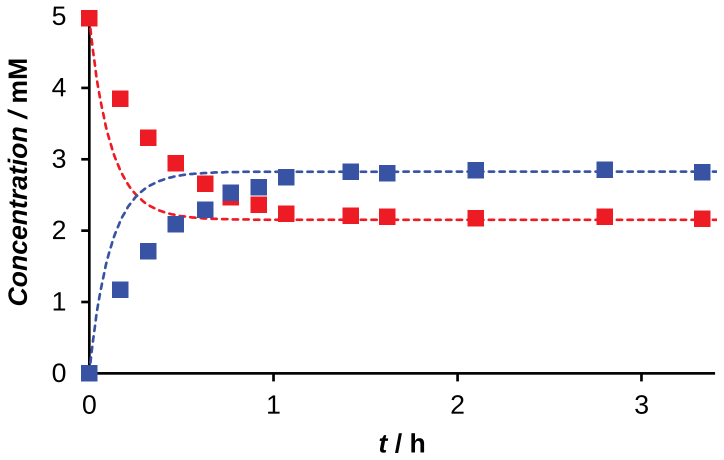

Figure S37. Kinetic profiles for reaction **R3**(low [H^+]). Red squares: nanoparticle-bound-**2**. Dark blue squares: nanoparticle-bound-**3**. Red dashed lines: MC-**2**. Blue dashed lines: MC-**3**. Conditions: hydrazide **4** (1.0 Eq. with respect to AuNP-**2** or MC-**2**), CF_3CO_2H (5 mM), 9:1 *v*/*v* DMF/D_2O, rt.

Reaction R4

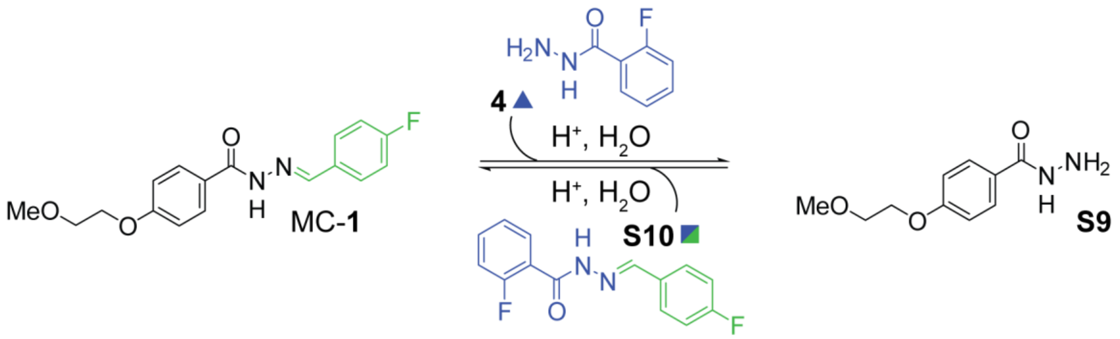

Table S32. Rate constants estimated for hydrazone exchange **R4** performed on MC-**1**.

[MC-**1**]$_0$ /mM	[**4**]$_0$ /mM	[CF_3CO_2H] / mM	k_{fwd} / $mM^{-1}h^{-1}$	k_{rev} / $mM^{-1}h^{-1}$	K (k_{fwd} / k_{rev})	K (NMR)[a]	$k_{init.}$ / $mM^{-1}h^{-1}$
4.78	4.75	20.0	0.363	0.111	3.28	2.81	0.35
4.73	4.81	20.4	0.353	0.103	3.42	2.92	0.34
4.74	4.87	20.4	0.355	0.094	3.76	3.06	0.34
		Mean	0.357	0.103	3.49	2.93	0.34
		SD	± 0.006	± 0.008	± 0.25	± 0.13	± 0.01

[a] Determined using the average of the concentrations from three data points collected at the apparent equilibrium position.

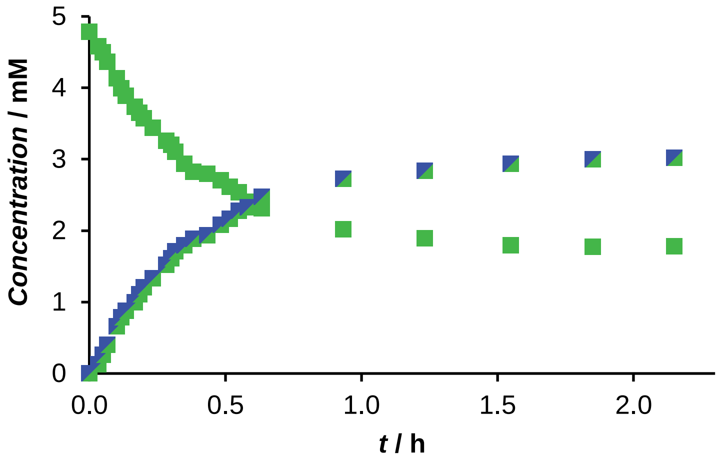

Figure S38. Kinetic profiles for reaction **R4**. Green squares: hydrazone MC-**1**. Green/blue squares: hydrazone **S10**. Conditions: hydrazide **4** (1.0 Eq. with respect to MC-**1**), CF_3CO_2H (20 mM), 9:1 *v*/*v* DMF/D_2O, rt.

8.2 Hydrazone hydrolysis

Hydrazone hydrolysis reactions **H1** and **H2** were performed at a concentration of ca. 5 mM in starting hydrazone, in presence of an excess of acid catalyst (20 mM CF_3CO_2H) and large excess of water (10% *v*/*v* ≈ 1000 equivalents) in DMF. Stock solutions of model compounds or nanoparticles were prepared in 9:1 *v*/*v* DMF/D_2O. Concentrations of each fluorinated species were assessed by ^{19}F NMR relative to 4-fluorotoluene as internal standard, which was added at a known concentration (ca. 5 mM). The reaction was triggered by addition of CF_3CO_2H. The NMR tube was held at rt and occasionally agitated while recording NMR spectra at intermediate time points to track reaction progress. Each hydrolysis was monitored until equilibrium was reached. The total concentration of fluorinated species measured remained constant throughout each experiment time-course and was always in agreement with the starting amount of hydrazone. Moreover, no additional resonances were observed in the ^{19}F NMR spectra recorded at later time points, indicating the absence of significant side reactions or decomposition processes. Three experimental replicates were performed for each reaction.

Each reaction was modelled as a reversible process characterised by a pseudo-first order forward rate constant (k_{hydr}), and second order reverse rate constant (k_{cond}). Rate constants were independently determined for each experimental replicate then averaged to give the results reported in **Tables S33–S36.** Full time-course plots for a representative example of each experiment are shown in **Figures S39–S40.**

Reaction H1

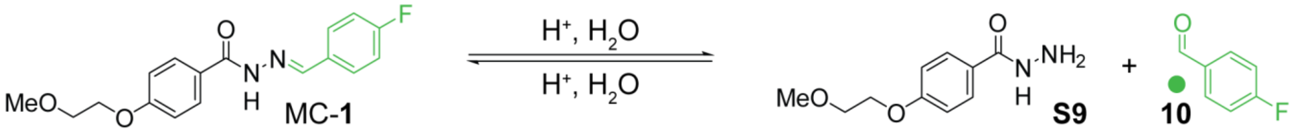

Table S33. Rate constants estimated for hydrazone hydrolysis **H1** performed on MC-**1**.

$[\text{MC-}\mathbf{1}]_0$ /mM	$[CF_3CO_2H]$ / mM	k_{hydr} / h^{-1}	k_{cond} / $mM^{-1}h^{-1}$	K (k_{hydr} / k_{cond})	K (NMR)[a]
4.86	20.5	4.24×10^{-2}	54.1×10^{-2}	7.83×10^{-2}	7.61×10^{-2}
4.80	20.2	4.31×10^{-2}	59.7×10^{-2}	7.21×10^{-2}	6.85×10^{-2}
4.77	19.8	4.11×10^{-2}	57.5×10^{-2}	7.15×10^{-2}	6.69×10^{-2}
	Mean	4.22×10^{-2}	57.1×10^{-2}	7.40×10^{-2}	7.05×10^{-2}
	SD	$\pm 0.10 \times 10^{-2}$	$\pm 2.8 \times 10^{-2}$	$\pm 0.37 \times 10^{-2}$	$\pm 0.49 \times 10^{-2}$

[a] Determined using the average of the concentrations from three data points collected at the apparent equilibrium position.

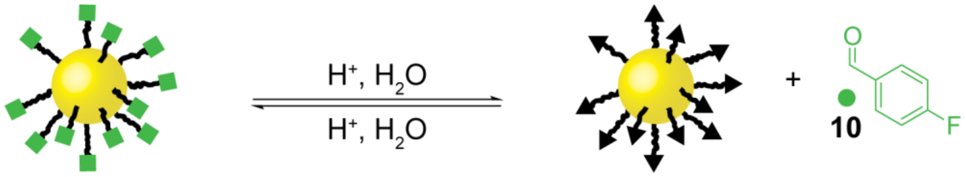

Table S34. Rate constants estimated for hydrazone hydrolysis **H1** performed on AuNP-**1**.

$[\text{AuNP-}\mathbf{1}]_0$ /mM	$[CF_3CO_2H]$ / mM	k_{hydr} / h^{-1}	k_{cond} / $mM^{-1}h^{-1}$	K (k_{hydr} / k_{cond})	K (NMR)[a]
4.77	19.9	1.83×10^{-2}	75.5×10^{-2}	2.42×10^{-2}	2.37×10^{-2}
4.82	19.9	2.18×10^{-2}	94.6×10^{-2}	2.30×10^{-2}	2.34×10^{-2}
4.85	20.0	2.02×10^{-2}	85.9×10^{-2}	2.35×10^{-2}	2.34×10^{-2}
	Mean	2.01×10^{-2}	85.3×10^{-2}	2.36×10^{-2}	2.35×10^{-2}
	SD	$\pm 0.17 \times 10^{-2}$	$\pm 9.6 \times 10^{-2}$	$\pm 0.06 \times 10^{-2}$	$\pm 0.02 \times 10^{-2}$

[a] Determined using the average of the concentrations from three data points collected at the apparent equilibrium position.

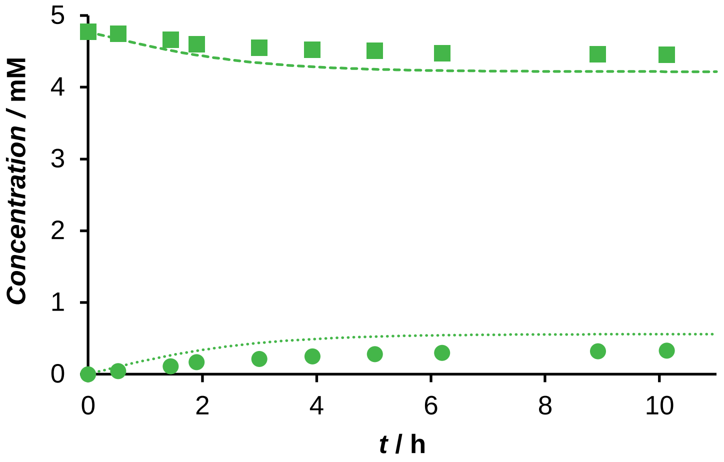

Figure S39. Kinetic profiles for reaction **H1**. Green squares: nanoparticle-bound-**1**. Green circles: aldehyde **10** (from hydrolysis of AuNP-**1**). Green dashed line: MC-**1**. Green dotted line: aldehyde **10** (from hydrolysis of MC-**1**). Conditions: AuNP-**1** or MC-**1** (ca. 5 mM), CF_3CO_2H (20 mM), 9:1 *v*/*v* DMF/D_2O, rt.

Reaction H2

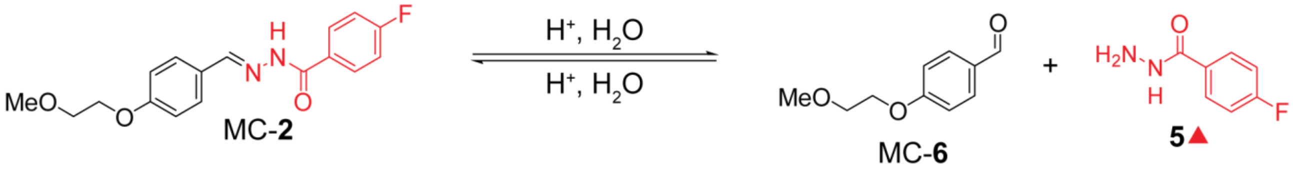

Table S35. Rate constants estimated for hydrazone hydrolysis **H2** performed on MC-**2**.

[MC-**1**]$_0$ /mM	[CF_3CO_2H] / mM	K_{hydr} / h^{-1}	K_{cond} / $mM^{-1}h^{-1}$	K (k_{hydr} / k_{cond})	K (NMR)[a]
4.96	20.4	10.7 x 10^{-2}	69.4 x 10^{-2}	15.4 x 10^{-2}	15.1 x 10^{-2}
4.69	19.9	10.3 x 10^{-2}	80.4 x 10^{-2}	12.9 x 10^{-2}	12.7 x 10^{-2}
4.70	20.3	11.8 x 10^{-2}	89.7 x 10^{-2}	13.1 x 10^{-2}	13.4 x 10^{-2}
	Mean	10.9 x 10^{-2}	79.8 x 10^{-2}	13.8 x 10^{-2}	13.7 x 10^{-2}
	SD	± 0.8 x 10^{-2}	± 10.2 x 10^{-2}	± 1.4 x 10^{-2}	± 1.3 x 10^{-2}

[a] Determined using the average of the concentrations from three data points collected at the apparent equilibrium position.

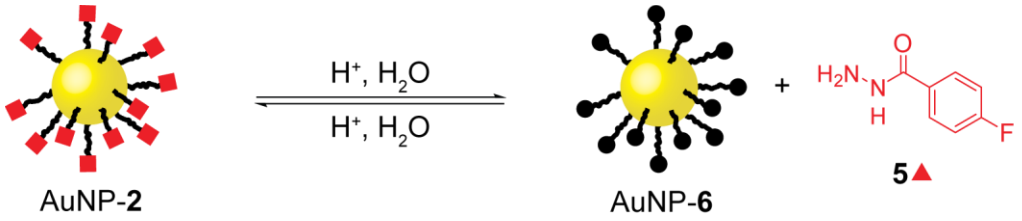

Table S36. Rate constants estimated for hydrazone hydrolysis **H2** performed on AuNP-**2**

[AuNP-**2**]$_0$ /mM	[CF_3CO_2H] / mM	k_{hydr} / h^{-1}	k_{cond} / $mM^{-1}h^{-1}$	K (k_{hydr} / k_{cond})	K (NMR)[a]
4.75	20.1	8.64 x 10^{-2}	75.2 x 10^{-2}	11.5 x 10^{-2}	11.7 x 10^{-2}
4.74	19.9	8.50 x 10^{-2}	74.4 x 10^{-2}	11.4 x 10^{-2}	11.6 x 10^{-2}
4.80	20.1	7.45 x 10^{-2}	66.5 x 10^{-2}	11.2 x 10^{-2}	10.8 x 10^{-2}
	Mean	8.20 x 10^{-1}	72.1 x 10^{-1}	11.4 x 10^{-2}	11.4 x 10^{-2}
	SD	± 0.65 x 10^{-2}	± 4.8 x 10^{-2}	± 0.1 x 10^{-2}	± 0.5 x 10^{-2}

[a] Determined using the average of the concentrations from three data points collected at the apparent equilibrium position.

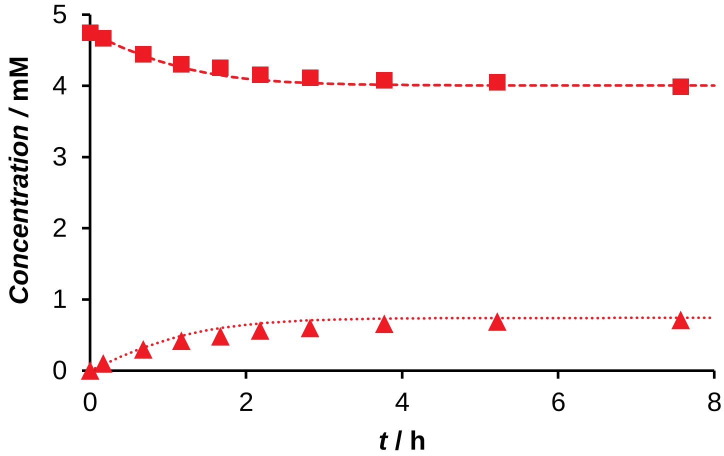

Figure S40. Kinetic profiles for reaction **H2**. Red squares: nanoparticle-bound-**2**. Red triangles: hydrazide **5** (from hydrolysis of AuNP-**2**). Red dashed line: MC-**2**. Red dotted line: hydrazide **5** (from hydrolysis of MC-**2**). Conditions: AuNP-**2** or MC-**2** (ca. 5 mM), CF_3CO_2H (20 mM), 9:1 *v*/*v* DMF/D_2O, rt.

9. Complementary nanoparticle assembly

Sample preparation

Stock solutions of AuNP-**1** and AuNP-**2** were separately prepared by dissolving a portion (ca. 2 mg) of dried nanoparticles in DMF/D_2O (9:1 *v*/*v*, 2 mL). Concentrations of surface-bound hydrazones were assessed by ^{19}F NMR relative to 4-fluorotoluene as internal standard, which was added at a known concentration (ca. 5 mM). Solutions **A** and **B** were then prepared volumetrically to give accurately known concentrations of surface-bound **1** and **2**, respectively:

A. AuNP-**1** in 9:1 *v*/*v* DMF/H_2O (0.15 mM surface-bound **1**).

B. AuNP-**2** in 9:1 *v*/*v* DMF/H_2O (0.15 mM surface-bound **2**).

Solutions **C** and **D** were also prepared volumetrically to give accurately known concentrations of CF_3CO_2H and aldehyde **10**, respectively:

C. CF_3CO_2H in 9:1 *v*/*v* DMF/H_2O (4 M).

D. Aldehyde **10** in 9:1 *v*/*v* DMF/H_2O (1 M).

Each solution was than sonicated for 10 minutes and filtered (Whatman Puradisc 13, polypropylene, 100 nm).

The following mixtures were prepared to contain a final overall concentration of 0.15 mM in terms of surface-bound hydrazones.

Complementary nanoparticle assembly: 0.075 mM AuNP-**1** (**A**, 1.50 mL) + 0.075 mM AuNP-**2** (**B**, 1.50 mL) + 20 mM CF_3CO_2H (**C**, 15.0 µL).

Control 1: 0.075 mM AuNP-**1** (**A**, 1.50 mL) + 0.075 mM AuNP-**2** (**B**, 1.50 mL) (no acid).

Control 2: 0.15 mM AuNP-**1** (**A**, 3.00 mL) + 20 mM CF_3CO_2H (**C**, 15.0 µL).

Control 3: 0.15 mM AuNP-**2** (**B**, 3.00 mL) + 20 mM CF_3CO_2H (**C**, 15.0 µL).

Adaptive nanoparticle assemblies were demonstrated in three independent experiments by adding different concentrations of capping agent (aldehyde **10**) after 5 days.

Capped assembly 1: 0.075 mM AuNP-**1** (**A**, 1.50 mL) + 0.075 mM AuNP-**2** (**B**, 1.50 mL) + 20 mM CF_3CO_2H (**C**, 15.0 µL) + 0.75 mM **10** (**D**, 1.69 µL).

Capped assembly 2: 0.075 mM AuNP-**1** (**A**, 1.50 mL) + 0.075 mM AuNP-**2** (**B**, 1.50 mL) + 20 mM CF_3CO_2H (**C**, 15.0 µL) + 3.0 mM **10** (**D**, 6.75 µL).

Capped assembly 3: 0.075 mM AuNP-**1** (**A**, 1.50 mL) + 0.075 mM AuNP-**2** (**B**, 1.50 mL) + 20 mM CF_3CO_2H (**C**, 15.0 µL) + 6.0 mM **10** (**D**, 13.5 µL).

9.1 Monitoring by UV-Vis absorbance spectroscopy

Changes in the concentration of colloidally stable material were monitored by UV-Vis absorbance spectroscopy without any further dilution. The first absorption spectrum (t = 0) was recorded prior addition of acid catalyst (where required), and subsequent spectra recorded at 24 h intervals. The timeseries of absorption spectra acquired during each experiment are shown in **Figures S41-S47**.

Complementary nanoparticle assembly

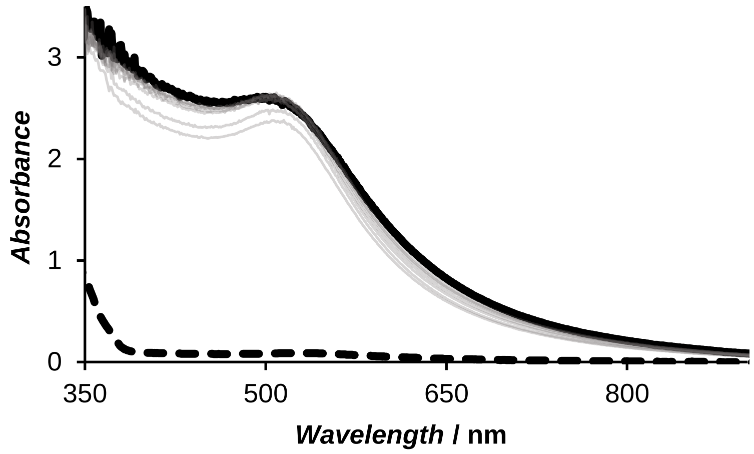

Figure S41. UV-Vis absorption spectra recorded over 8 days during complementary nanoparticle assembly. Conditions: AuNP-**1** (0.075 mM), AuNP-**2** (0.075 mM), CF_3CO_2H (20 mM), 9:1 *v*/*v* DMF/H_2O, 40 °C. Spectrum recorded immediately before addition of acid (black solid line); spectra recorded every 24 hours during the reaction time-course (grey solid lines); spectrum recorded after 8 days (black dashed line).

Control 1

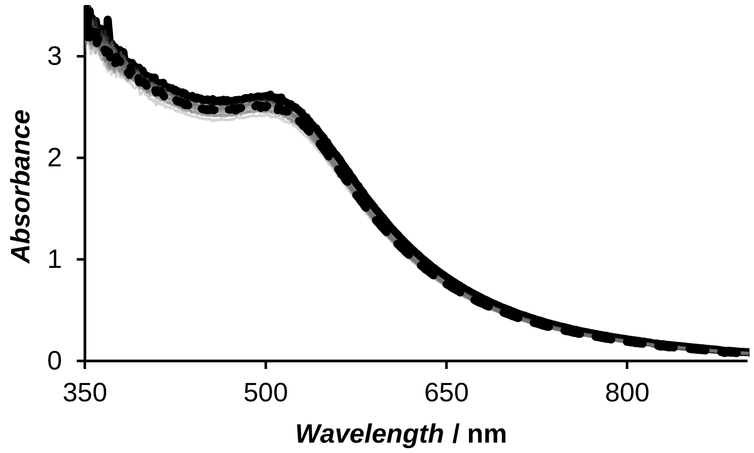

Figure S42. UV-Vis absorption spectra recorded over 25 days during control experiment 1. Conditions: AuNP-**1** (0.075 mM), AuNP-**2** (0.075 mM), 9:1 *v*/*v* DMF/H_2O, 40 °C. Spectrum recorded immediately before incubation at 40 °C (black solid line); spectra recorded every 24 hours during the reaction time-course (grey solid lines); spectrum recorded after 25 days (black dashed line).

Control 2

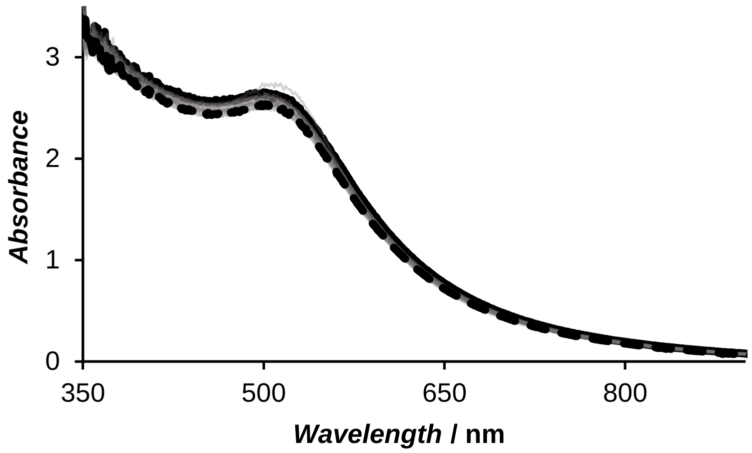

Figure S43. UV-Vis absorption spectra recorded over 25 days during control experiment 2. Conditions: AuNP-**1** (0.15 mM), CF_3CO_2H (20 mM), 9:1 *v*/*v* DMF/H_2O, 40 °C. Spectrum recorded immediately before addition of acid (black solid line); spectra recorded every 24 hours during the reaction time-course (grey solid lines); spectrum recorded after 25 days (black dashed line).

Control 3

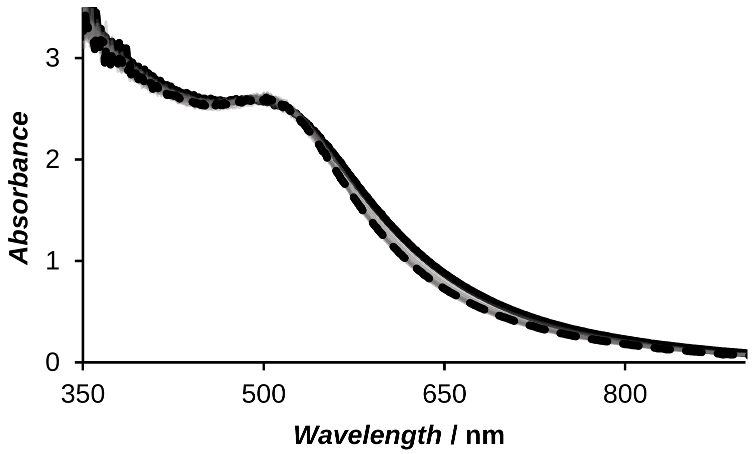

Figure S44. UV-Vis absorption spectra recorded over 25 days during control experiment 3. Conditions: AuNP-**2** (0.15 mM), CF_3CO_2H (20 mM), 9:1 *v*/*v* DMF/H_2O, 40 °C. Spectrum recorded immediately before addition of acid (black solid line); spectra recorded every 24 hours during the reaction time-course (grey solid lines); spectrum recorded after 25 days (black dashed line).

Capped assembly 1

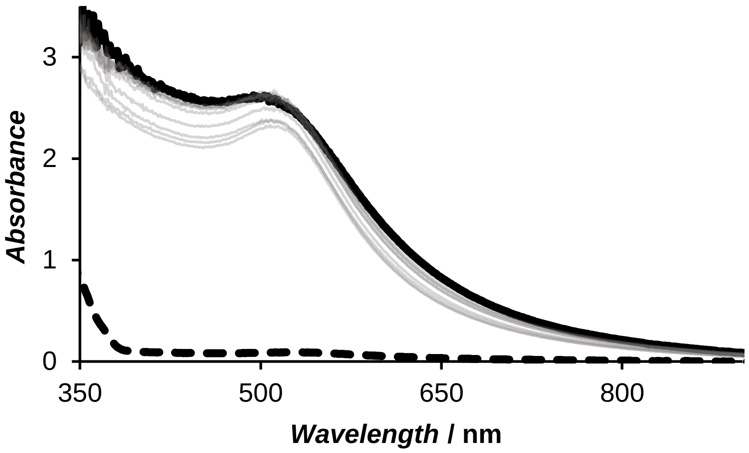

Figure S45. UV-Vis absorption spectra recorded over 10 days during experiment capped assembly 1. Conditions: AuNP-**1** (0.075 mM), AuNP-**2** (0.075 mM) CF_3CO_2H (20 mM), aldehyde **10** (0.75 mM, added after 5 days), 9:1 *v*/*v* DMF/H_2O, 40 °C. Spectrum recorded immediately before addition of acid (black solid line); spectra recorded every 24 hours during the reaction time-course (grey solid lines); spectrum recorded after 10 days (black dashed line).

Capped assembly 2

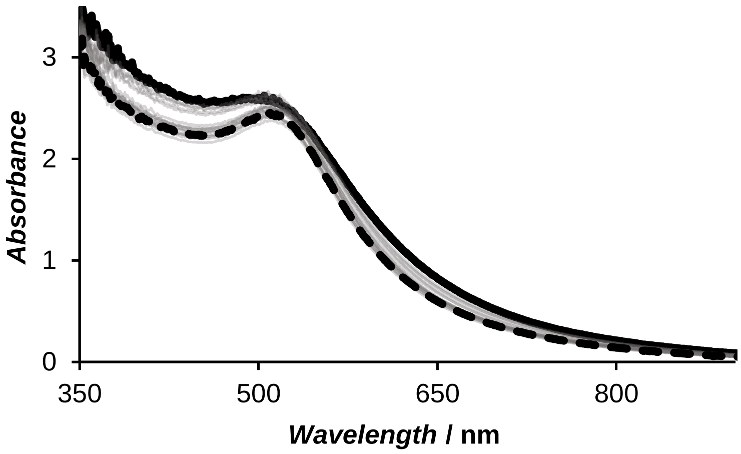

Figure S46. UV-Vis absorption spectra recorded over 25 days during experiment capped assembly 2. Conditions: AuNP-**1** (0.075 mM), AuNP-**2** (0.075 mM) CF_3CO_2H (20 mM), aldehyde **10** (3.0 mM, added after 5 days), 9:1 *v*/*v* DMF/H_2O, 40 °C. Spectrum recorded immediately before addition of acid (black solid line); spectra recorded every 24 hours during the reaction time-course (grey solid lines); spectrum recorded after 25 days (black dashed line).

Capped assembly 3

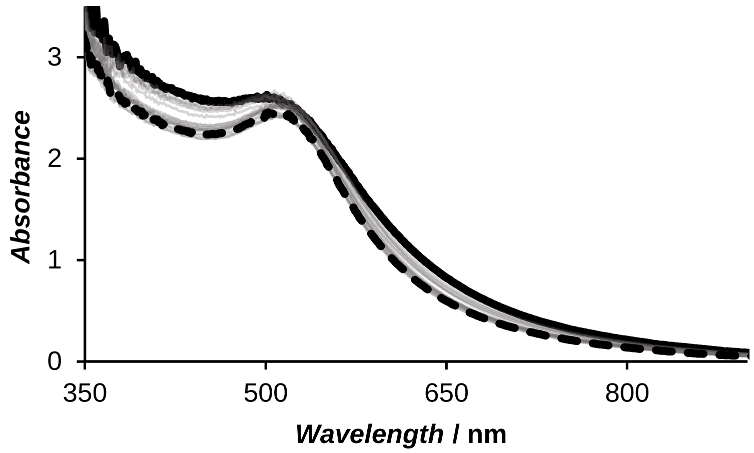

Figure S47. UV-Vis absorption spectra recorded over 25 days during experiment capped assembly 3. Conditions: AuNP-1 (0.075 mM), AuNP-2 (0.075 mM) CF_3CO_2H (20 mM), aldehyde 10 (6.0 mM, added after 5 days), 9:1 *v*/*v* DMF/H_2O, 40 °C. Spectrum recorded immediately before addition of acid (black solid line); spectra recorded every 24 hours during the reaction time-course (grey solid lines); spectrum recorded after 25 days (black dashed line).

9.2 Monitoring by dynamic light scattering

Solvodynamic particle size was monitored by dynamic light scattering. Samples were prepared by collecting 150 µL of solution previously subjected to UV-Vis absorbance spectroscopy, then diluting to a final volume of 2.00 mL, using fresh 9:1 *v*/*v* DMF/H_2O (1.85 mL). For each sample, three independent measurements were made in series, and the results averaged. In turn, each measurement is the average of 10 to 17 sequential scans. The solvodynamic sizes are reported as the mean size for distributions expressed as % number of particles (**Figure 6**). Size distributions were calculated by the instrument from the recorded intensity data. Equations reported in the literature were used to estimate appropriate values for viscosity,[1a] refractive index,[1b] and dielectric constant[1c] of 9:1 *v*/*v* DMF/D_2O from the reported values for the neat solvents at 25 °C.

Expansion of the solvodynamic size variations measured for complementary nanoparticle assembly during the first four days is reported in **Figure S48**. Solvodynamic size variations measured for control experiments 1–3 are reported in **Figures S49–S51**.

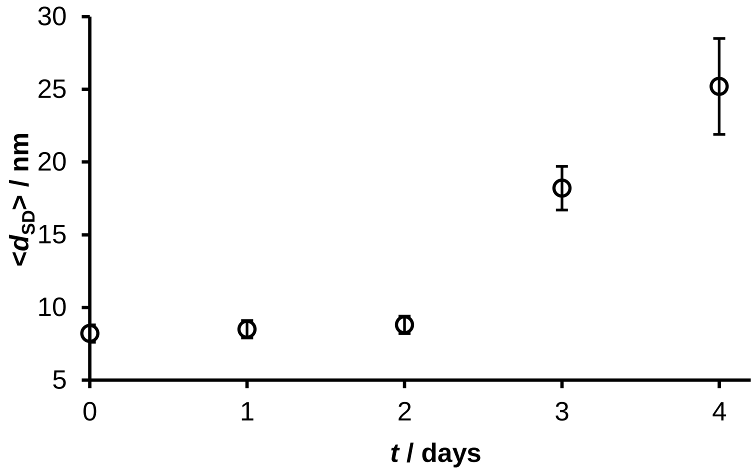

Figure S48. Expansion of the solvodynamic size variations measured by dynamic light scattering for complementary nanoparticle assembly over the first four days. Sizes are the mean of three measurements for distributions expressed in terms of % number of particles; error bars indicate ± 1 standard deviation.

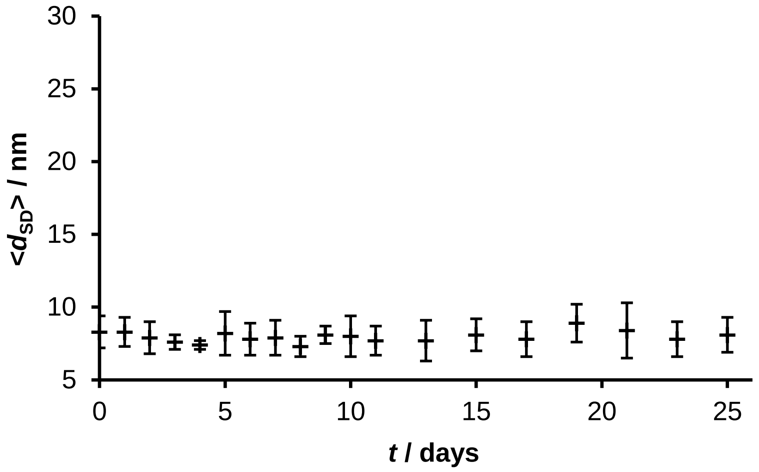

Figure S49. Solvodynamic size variations measured by dynamic light scattering for control experiment 1. Sizes are the mean of three measurements for distributions expressed in terms of number of particles; error bars indicate ± 1 standard deviation.

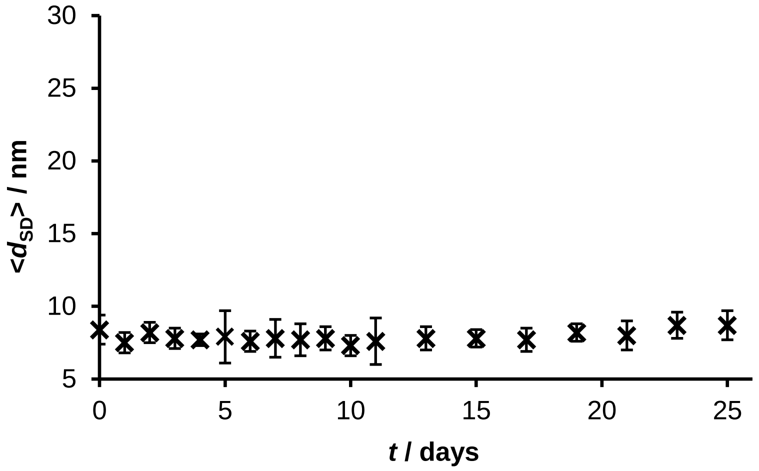

Figure S50. Solvodynamic size variations measured by dynamic light scattering for control experiment 2. Sizes are the mean of three measurements for distributions expressed in terms of number of particles; error bars indicate ± 1 standard deviation.

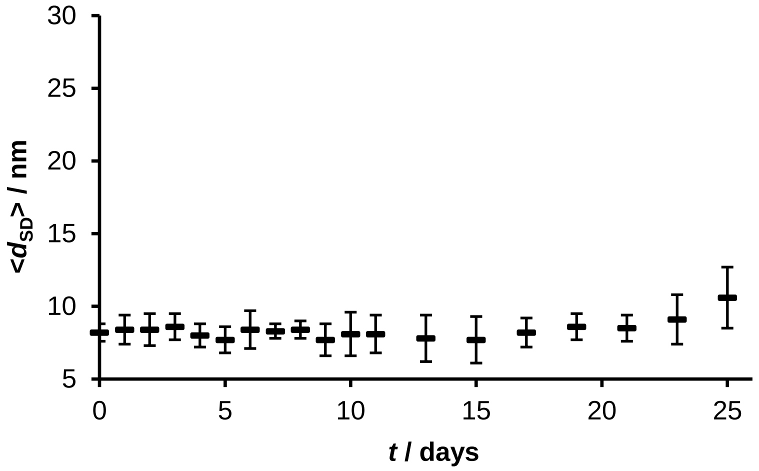

Figure S51. Solvodynamic size variations measured by dynamic light scattering for control experiment 3. Sizes are the mean of three measurements for distributions expressed in terms of number of particles; error bars indicate ± 1 standard deviation.

9.3 Monitoring by transmission electron microscopy

Samples of colloidally stable nanoparticles and aggregates were prepared for electron microscopy by depositing one drop of nanoparticle suspension (diluted as described above) onto a TEM grid sitting on a lint-free tissue. To image insoluble extended aggregates formed as a result of complementary nanoparticle assembly, the sample was first sonicated for 5 min before withdrawing a drop of the resulting suspension. All grids were left to dry at ambient pressure and temperature before imaging.

Complementary nanoparticle assembly (before addition of acid)

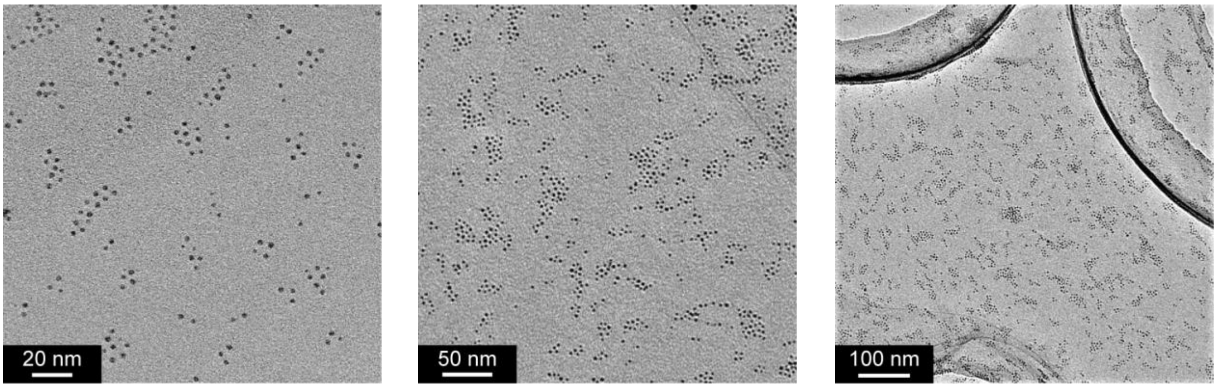

Complementary nanoparticle assembly (Day 5)

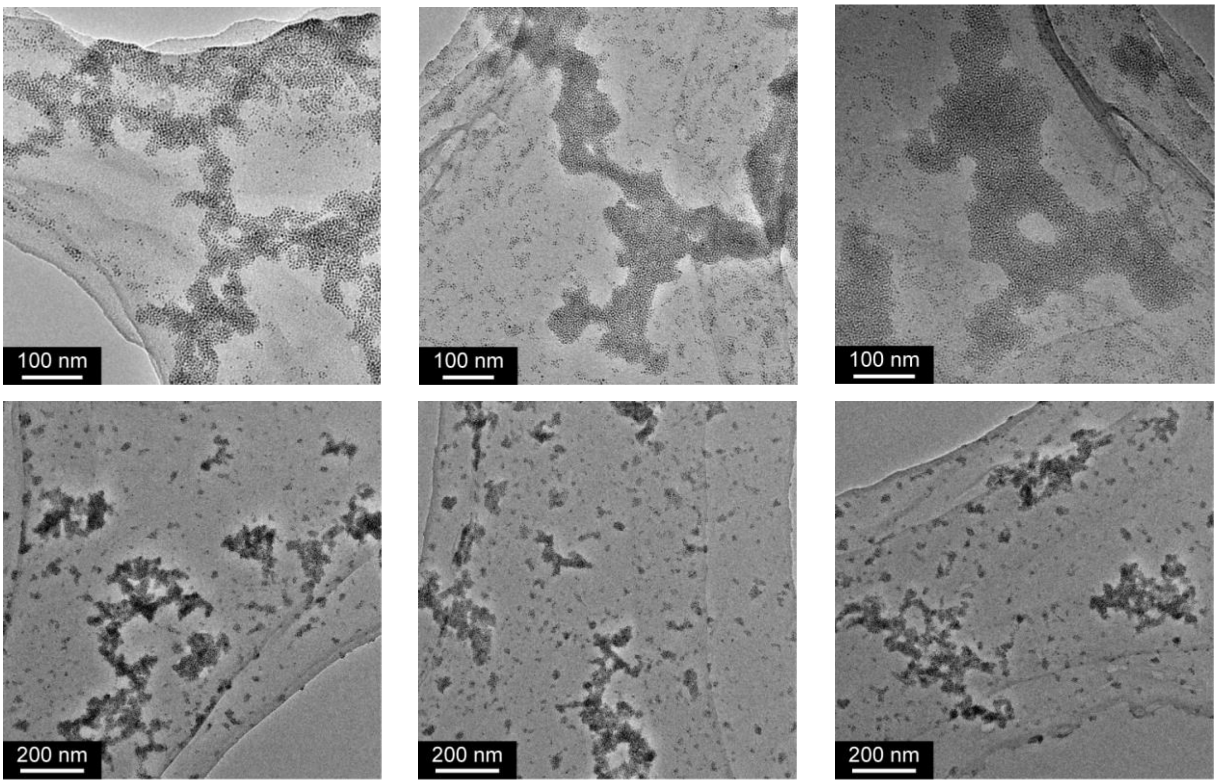

Complementary nanoparticle assembly (Day 8)

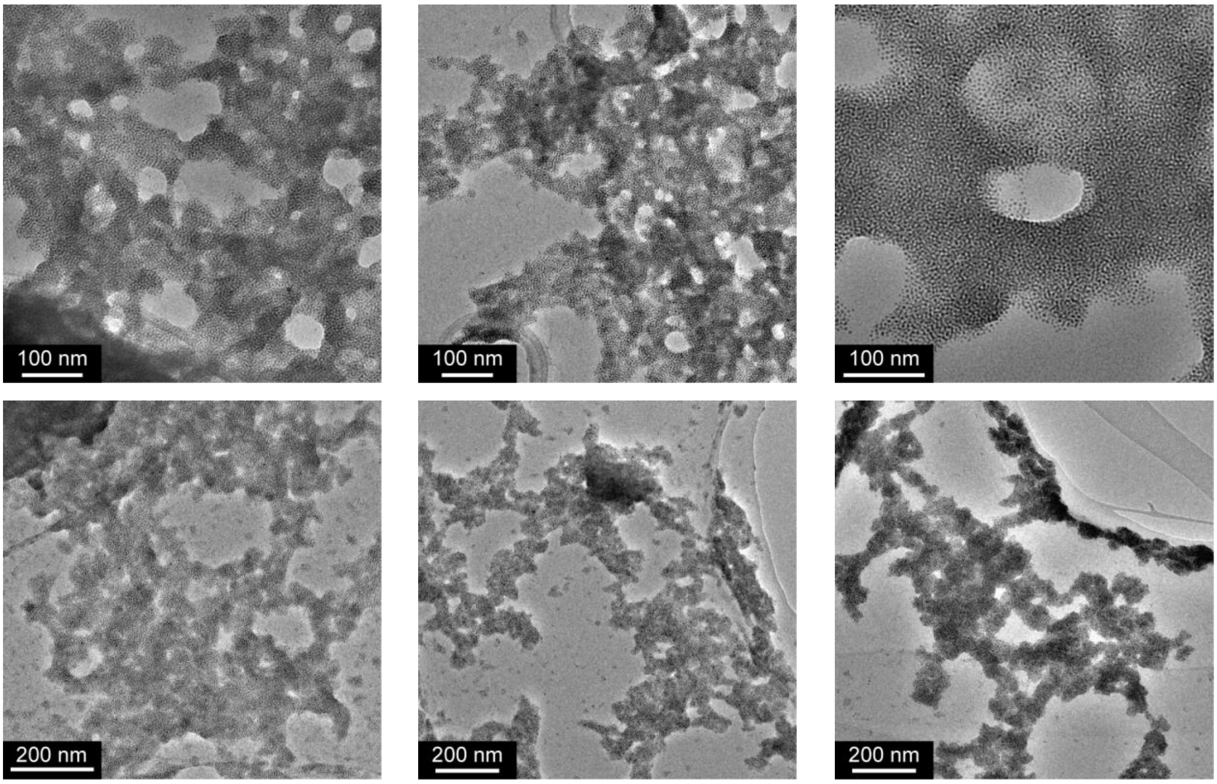

Control 1 (Day 25)

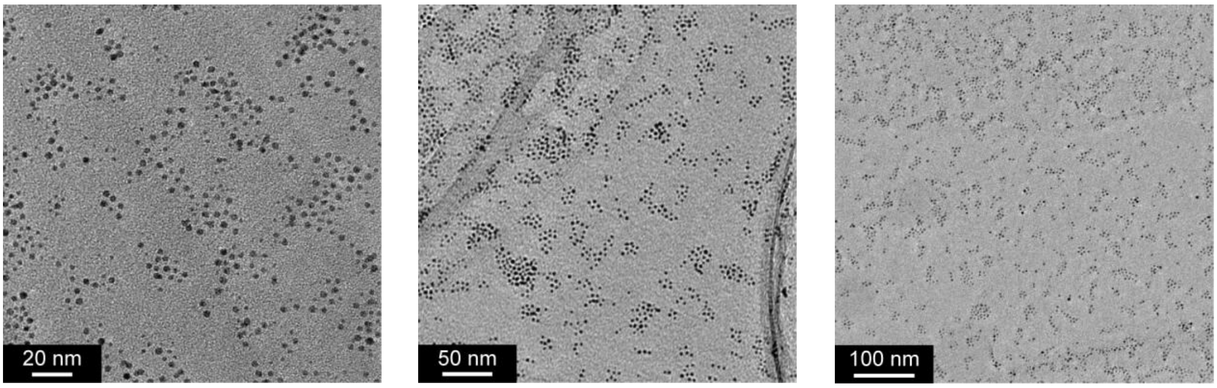

Control 2 (Day 25)

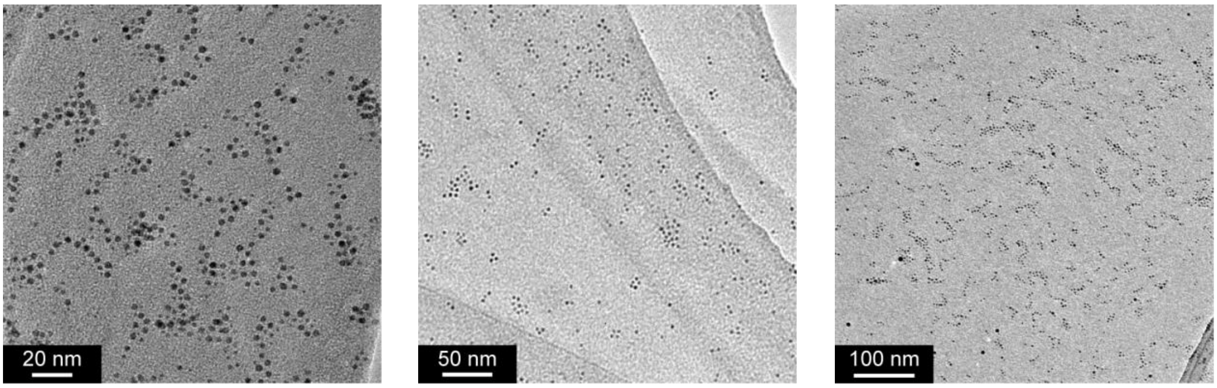

Control 3 (Day 25)

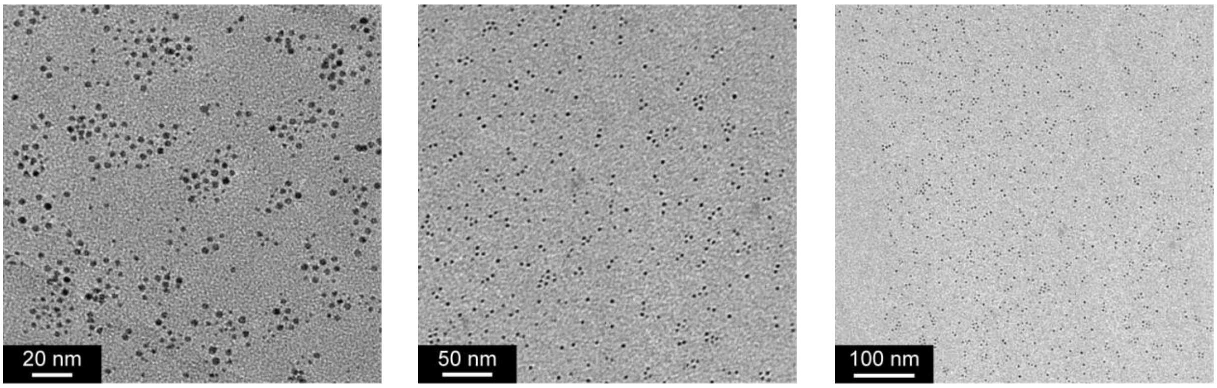

10. ^{1}H, ^{13}C and ^{19}F NMR spectra of organic compounds

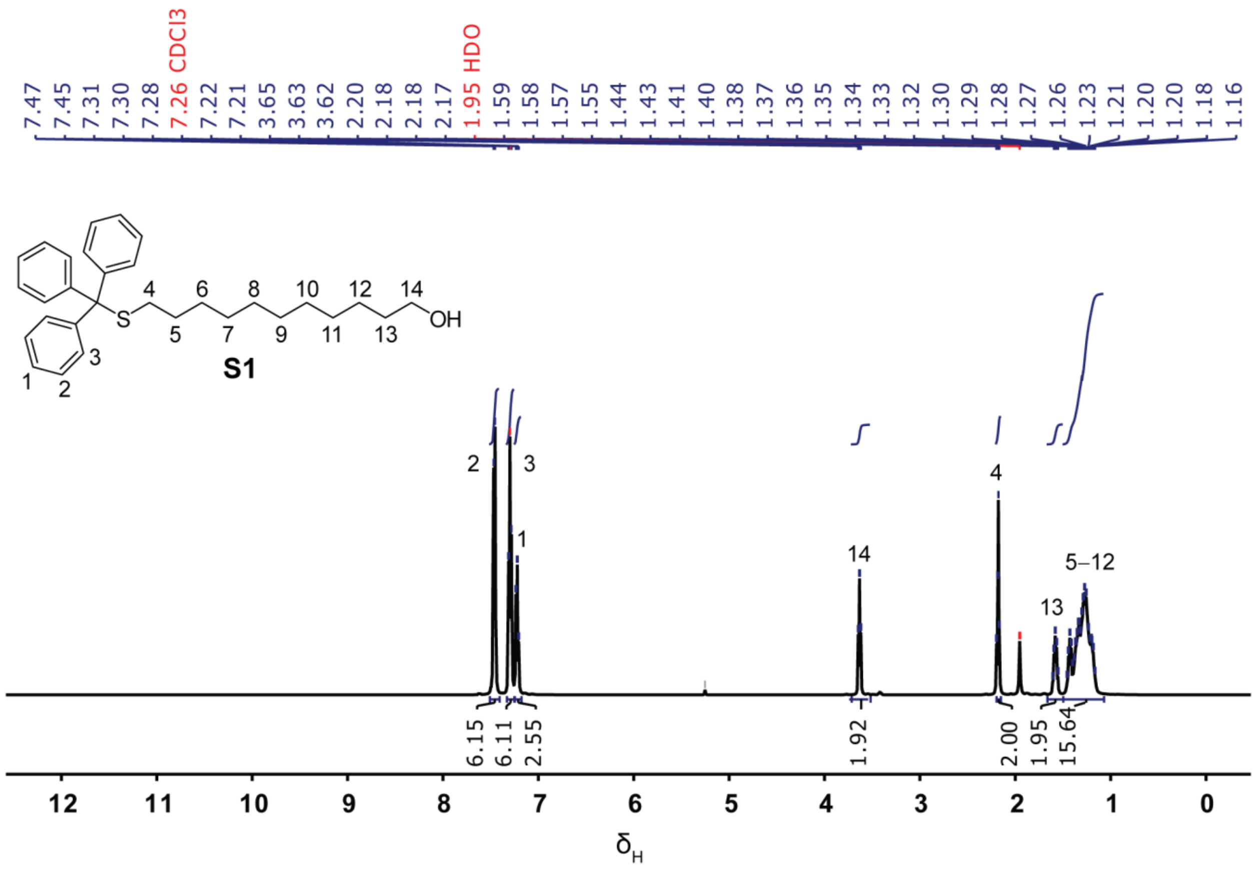

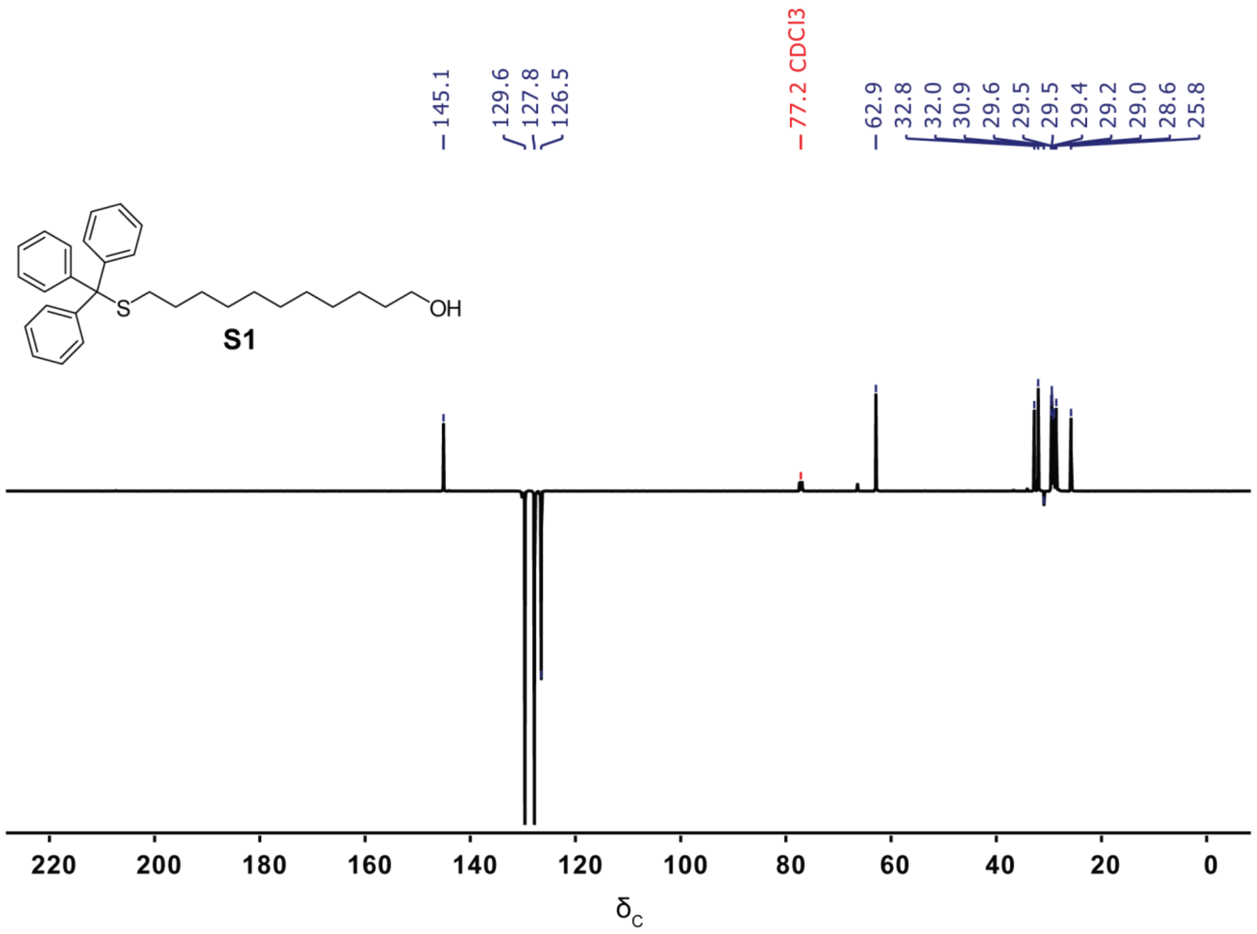

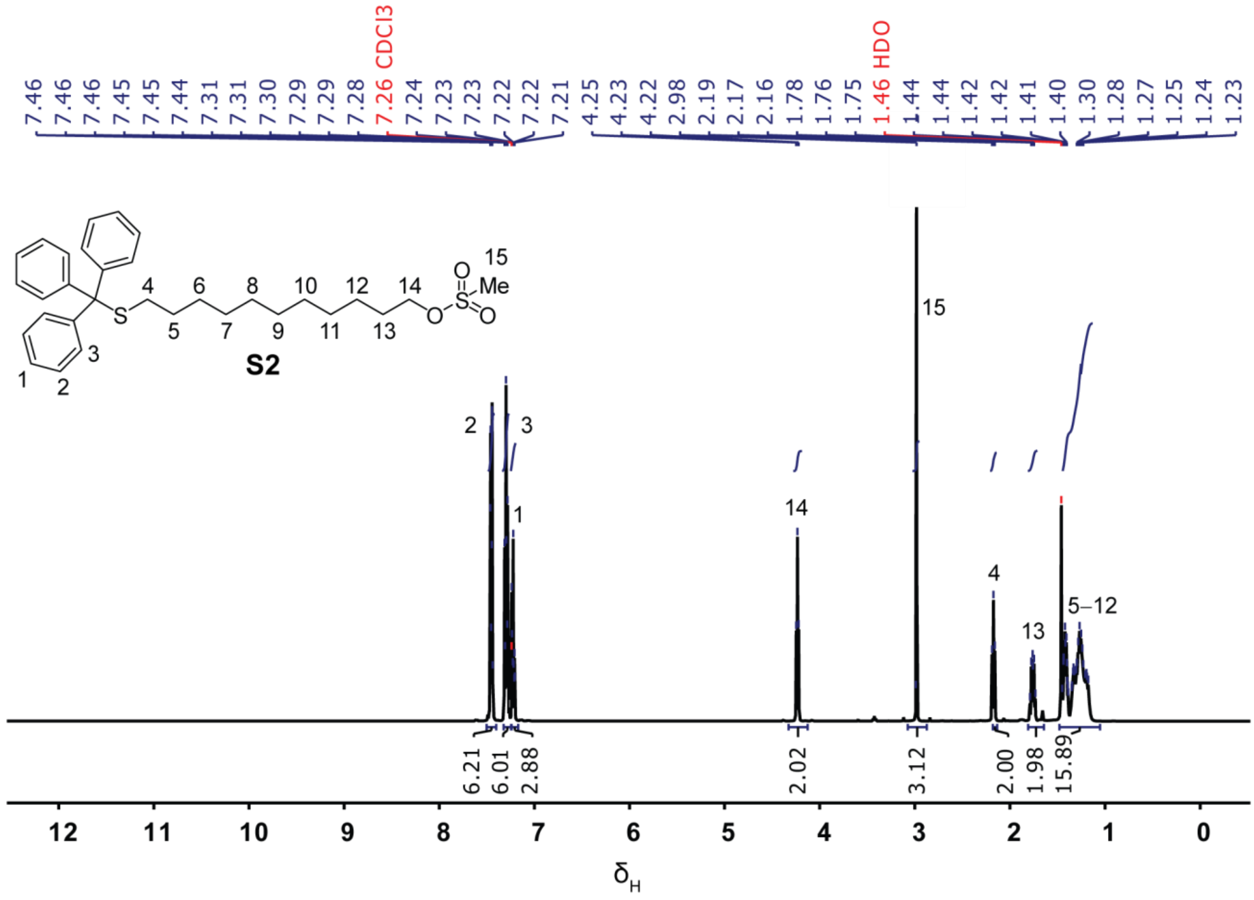
7.46 7.46 7.46 7.45 7.45 7.44 7.31 7.31 7.30 7.29 7.29 7.28 7.26 CDCl3 7.24 7.23 7.23 7.22 7.22 7.21
4.25 4.23 4.22 2.98 2.19 2.17 2.16 1.78 1.76 1.75 1.46 HDO 1.44 1.44 1.42 1.42 1.41 1.40 1.30 1.28 1.27 1.25 1.24 1.23
S2
15
Me
2
3
1
14
4
13
5–12
6.21
6.01
2.88
2.02
3.12
2.00
1.98
15.89
12 11 10 9 8 7 6 5 4 3 2 1 0
δH

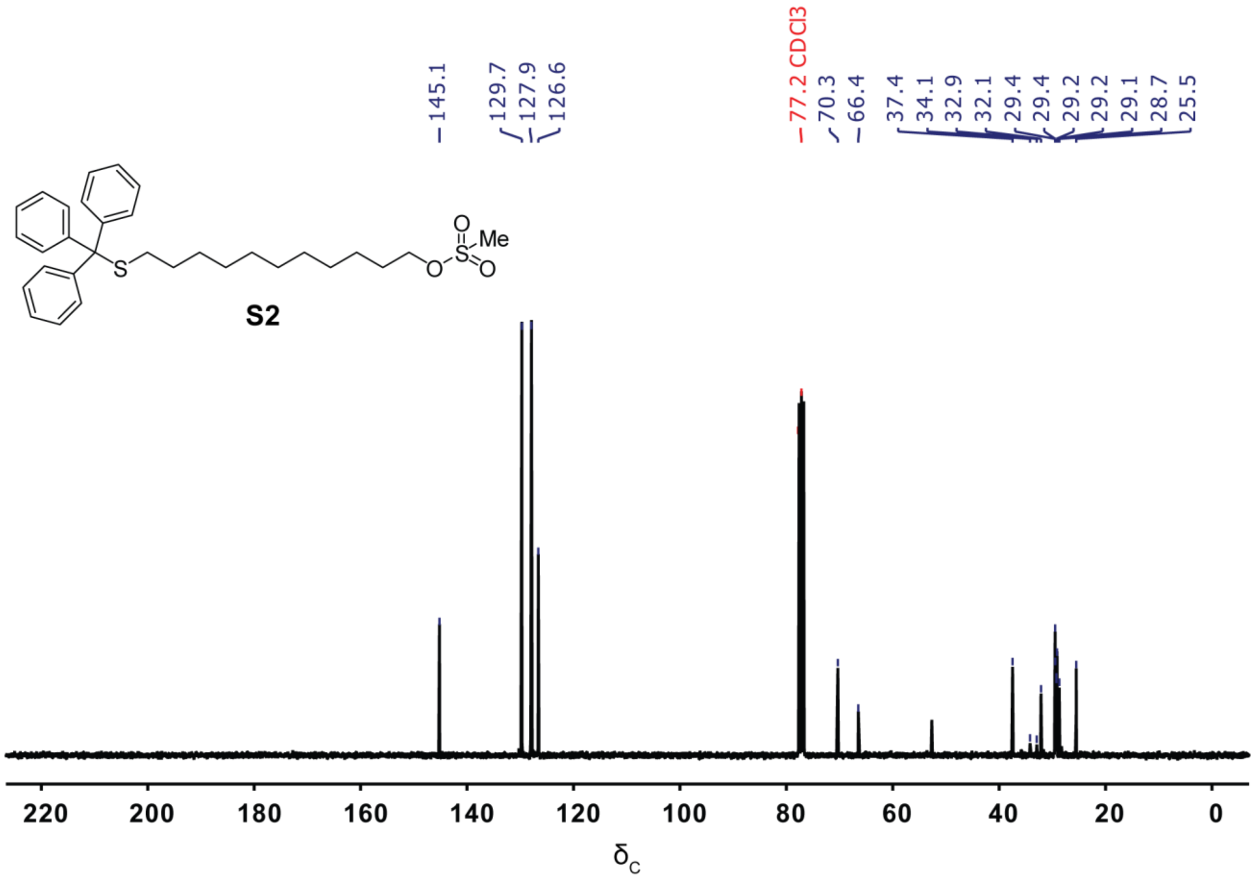
145.1
129.7 127.9 126.6
77.2 CDCl3 70.3 66.4
37.4 34.1 32.9 32.1 29.4 29.4 29.2 29.2 29.1 28.7 25.5
S2
Me
220 200 180 160 140 120 100 80 60 40 20 0
δC

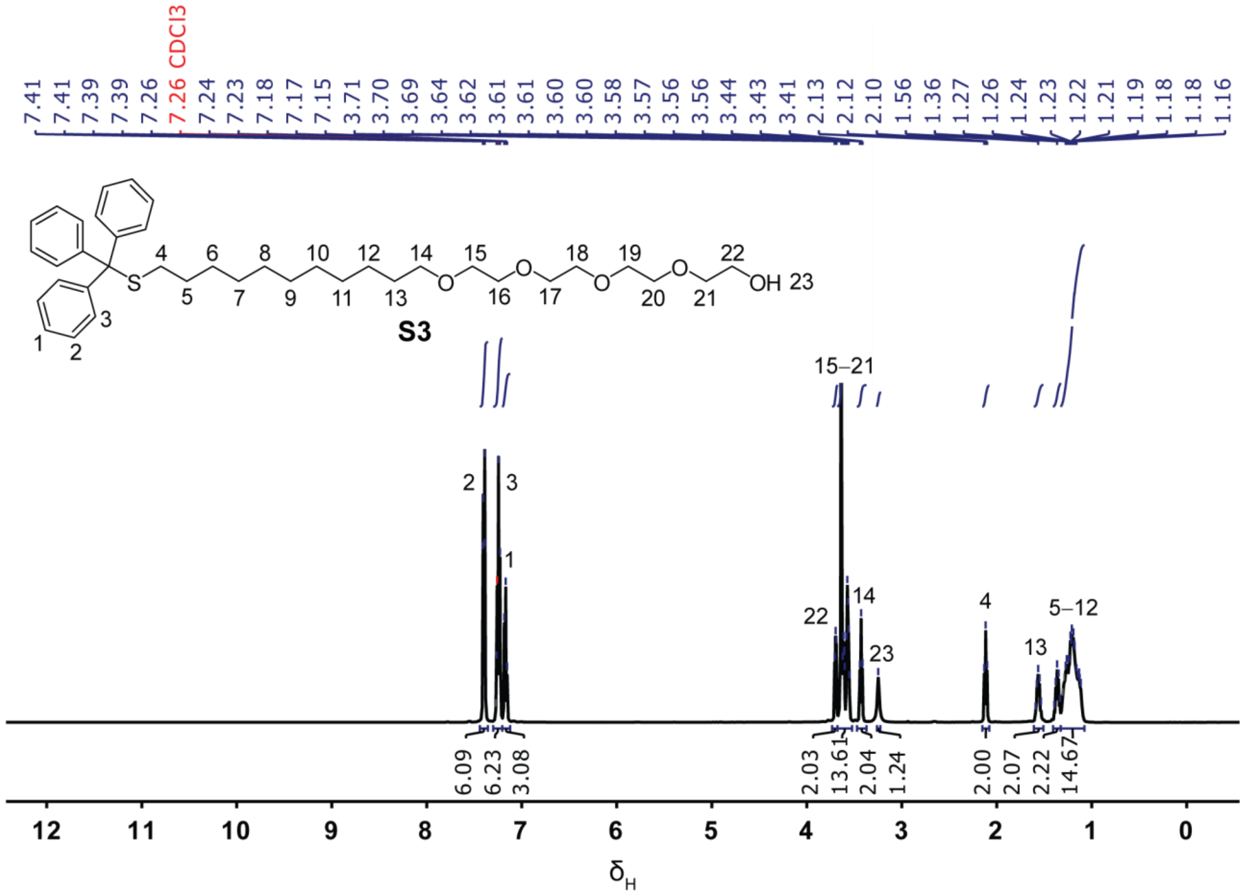
7.41 7.41 7.39 7.39 7.26 7.26 CDCl3 7.24 7.23 7.18 7.17 7.15 3.71 3.70 3.69 3.64 3.62 3.61 3.61 3.60 3.60 3.58 3.57 3.56 3.56 3.44 3.43 3.41 2.13 2.12 2.10 1.56 1.36 1.27 1.26 1.24 1.23 1.22 1.21 1.19 1.18 1.18 1.16
S3
15–21
2
3
1
22
14
23
4
13
5–12
6.09 6.23 3.08 2.03 13.61 2.04 1.24 2.00 2.07 2.22 14.67
12 11 10 9 8 7 6 5 4 3 2 1 0
δH

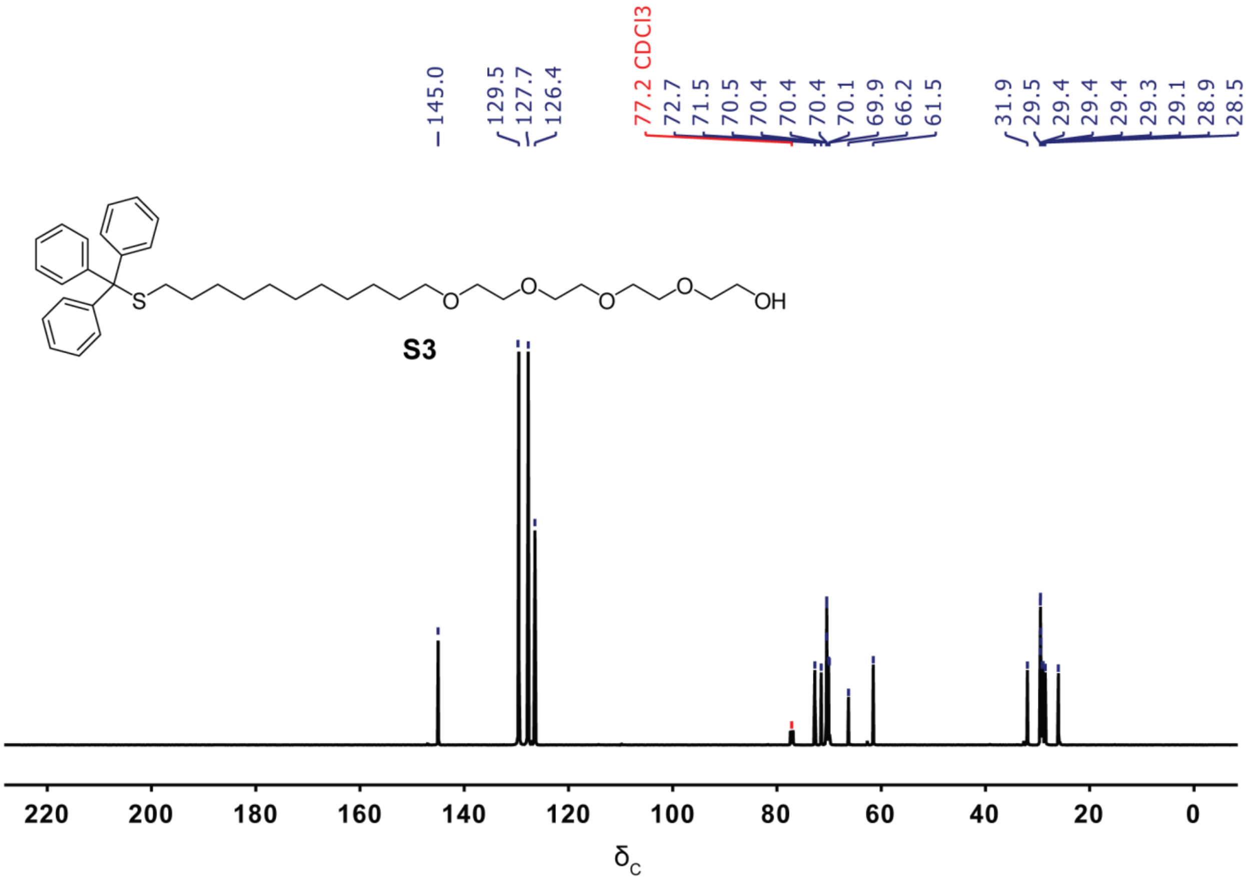
145.0 129.5 127.7 126.4 77.2 CDCl3 72.7 71.5 70.5 70.4 70.4 70.4 70.1 69.9 66.2 61.5 31.9 29.5 29.4 29.4 29.3 29.1 28.9 28.5
S3
220 200 180 160 140 120 100 80 60 40 20 0
δC

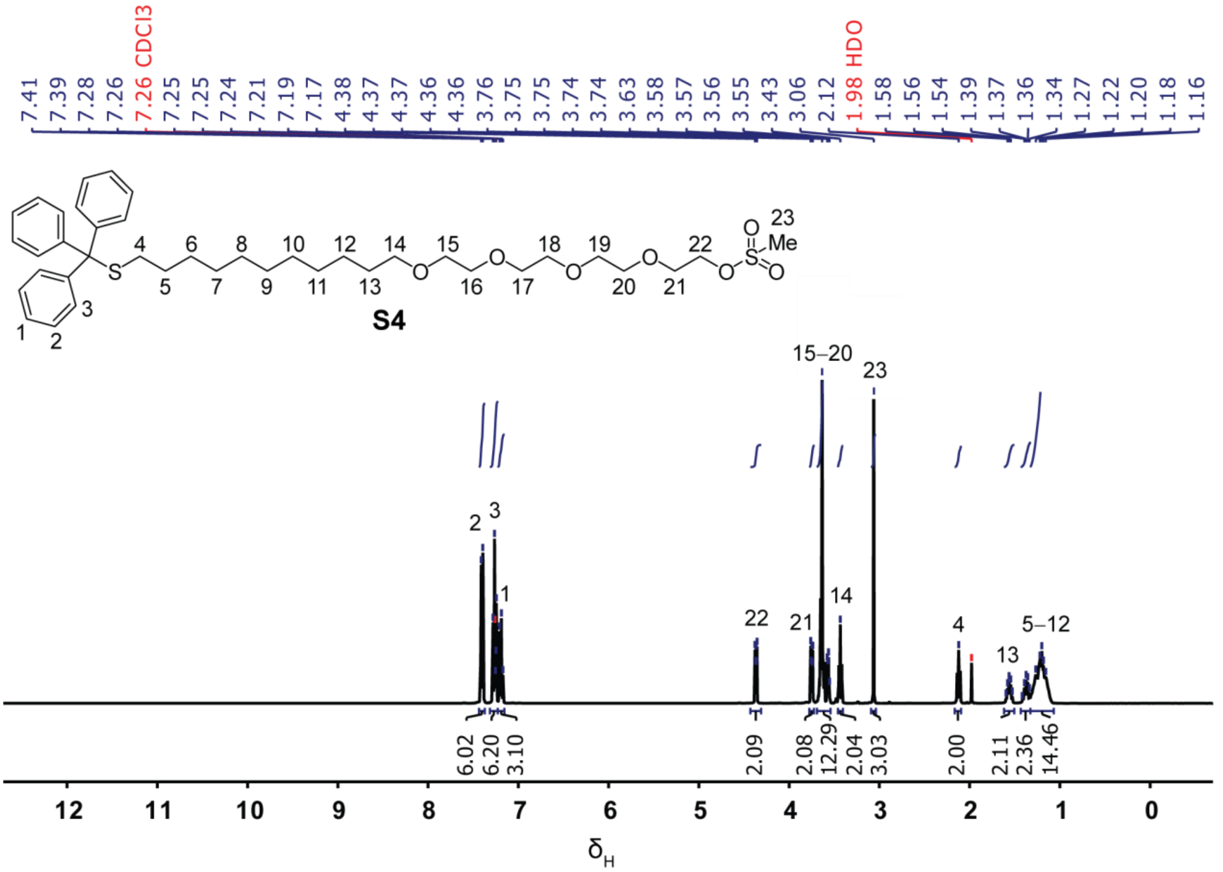
7.41 7.39 7.28 7.26 7.26 CDCl3 7.25 7.25 7.24 7.21 7.19 7.17 4.38 4.37 4.37 4.36 4.36 3.76 3.75 3.75 3.74 3.74 3.63 3.58 3.57 3.56 3.55 3.43 3.06 2.12 1.98 HDO 1.58 1.56 1.54 1.39 1.37 1.36 1.34 1.27 1.22 1.20 1.18 1.16
S4
15–20
23
2
3
1
22
21
14
4
13
5–12
6.02 6.20 3.10 2.09 2.08 12.29 2.04 3.03 2.00 2.11 2.36 14.46
δH

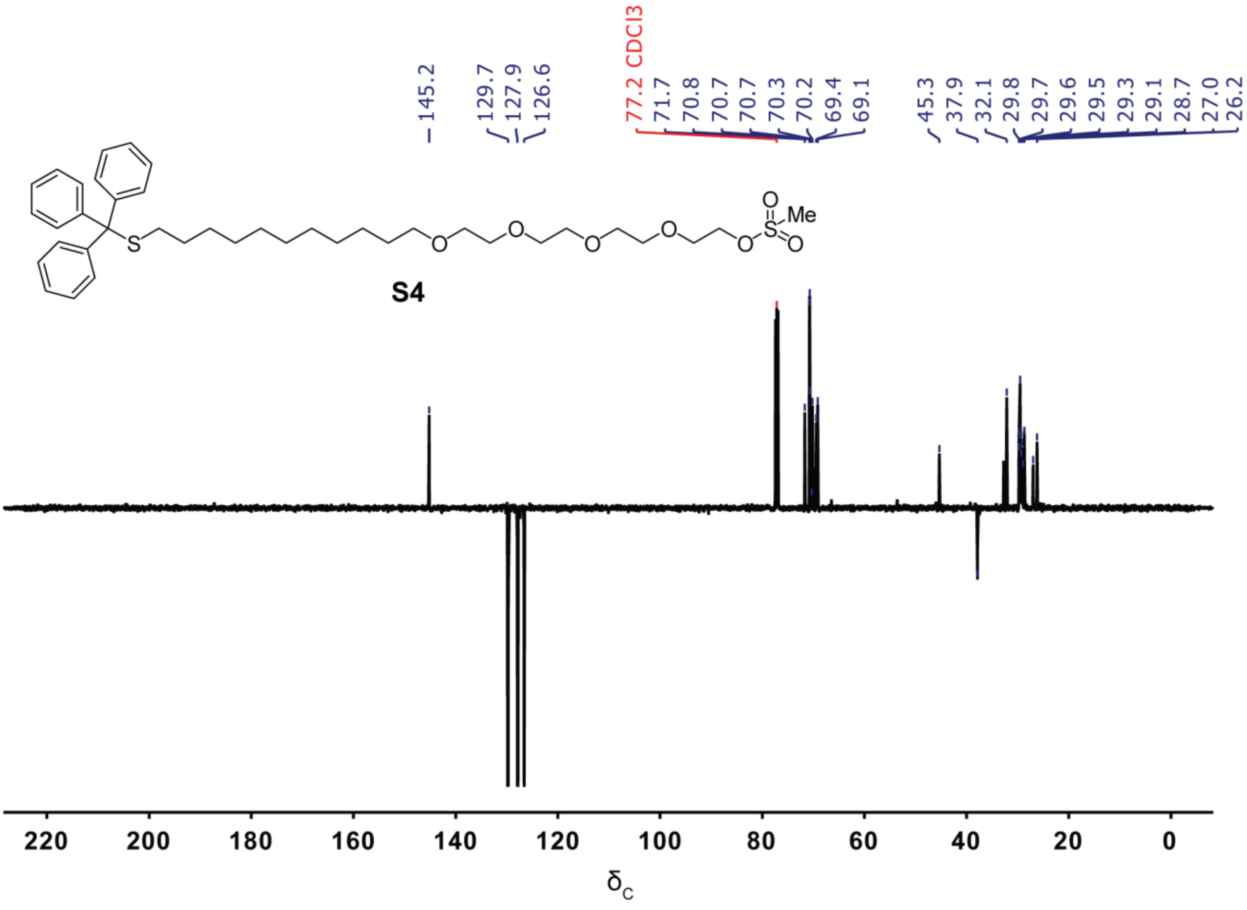
145.2 129.7 127.9 126.6 77.2 CDCl3 71.7 70.8 70.7 70.7 70.3 70.2 69.4 69.1 45.3 37.9 32.1 29.8 29.7 29.6 29.5 29.3 29.1 28.7 27.0 26.2
S4
δC

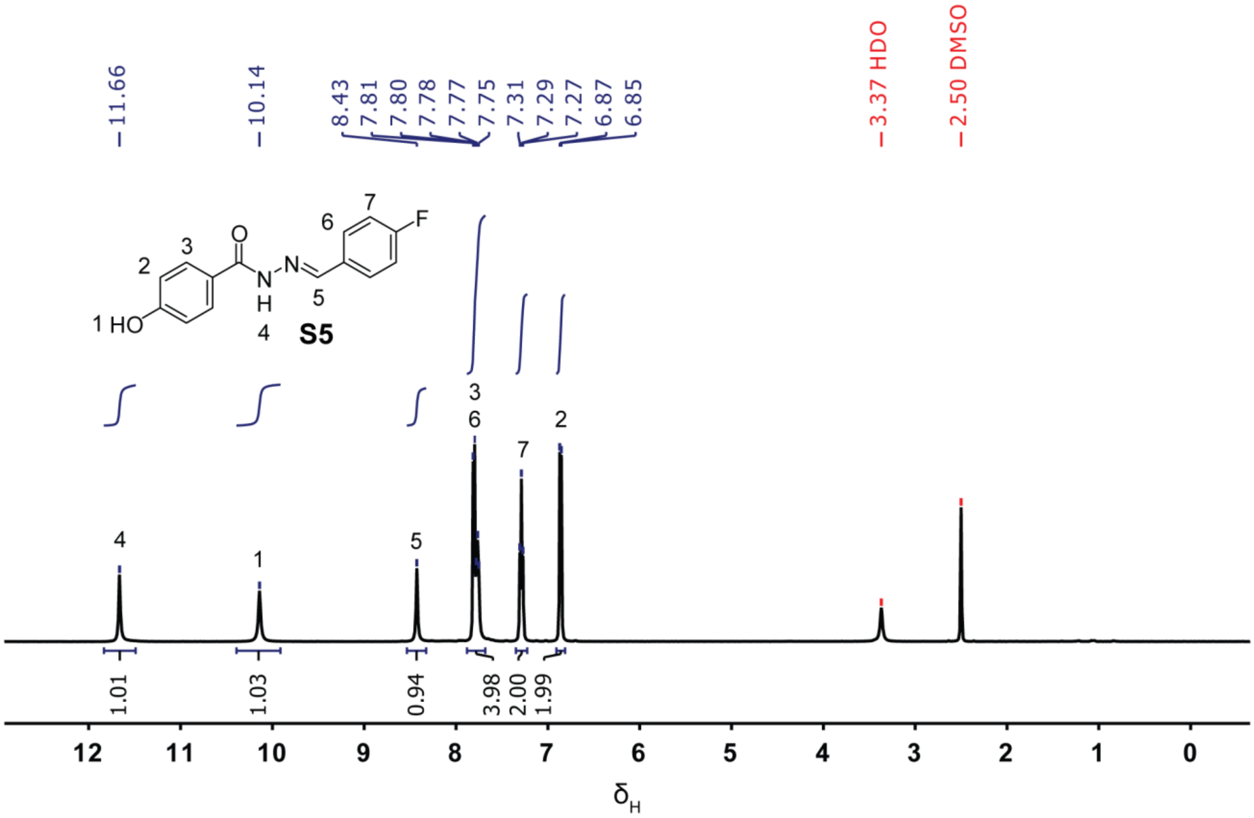
11.66
10.14
8.43
7.81
7.80
7.78
7.77
7.75
7.31
7.29
7.27
6.87
6.85
3.37 HDO
2.50 DMSO
S5
1 HO
2
3
4
5
6
7
F
1.01
1.03
0.94
3.98
2.00
1.99
12 11 10 9 8 7 6 5 4 3 2 1 0
δH

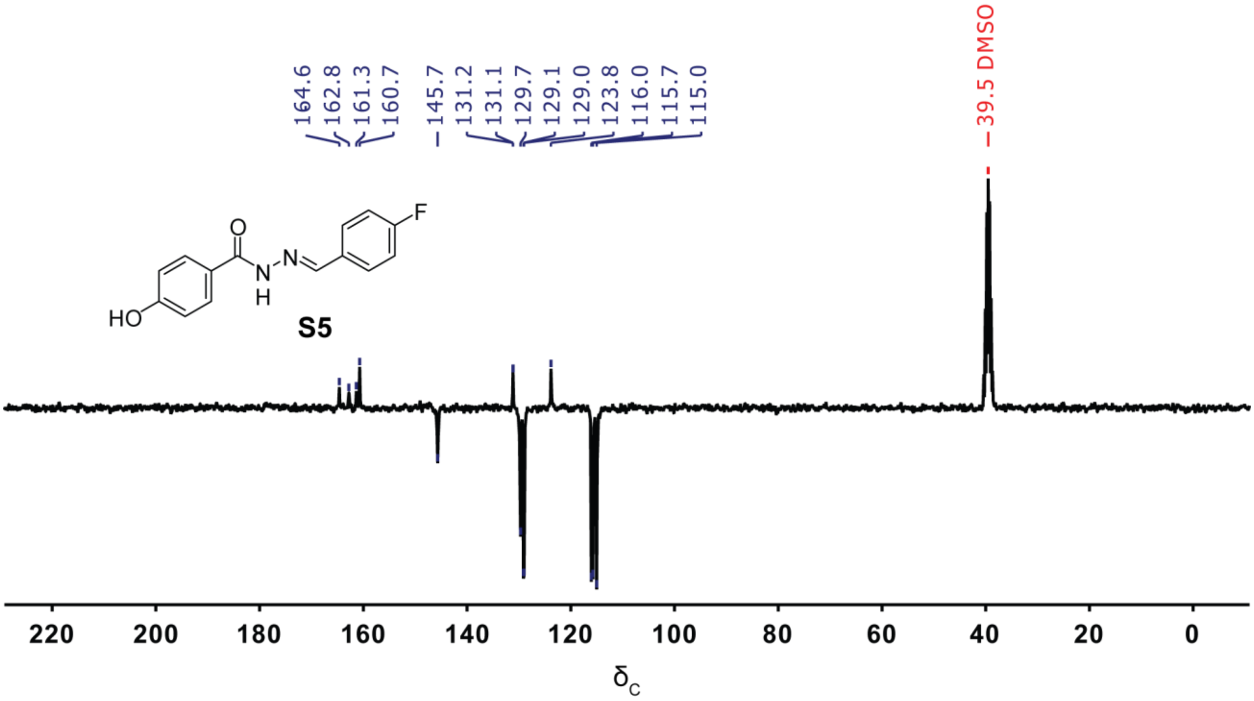
164.6
162.8
161.3
160.7
145.7
131.2
131.1
129.7
129.1
129.0
123.8
116.0
115.7
115.0
39.5 DMSO
S5
HO
F
220 200 180 160 140 120 100 80 60 40 20 0
δC

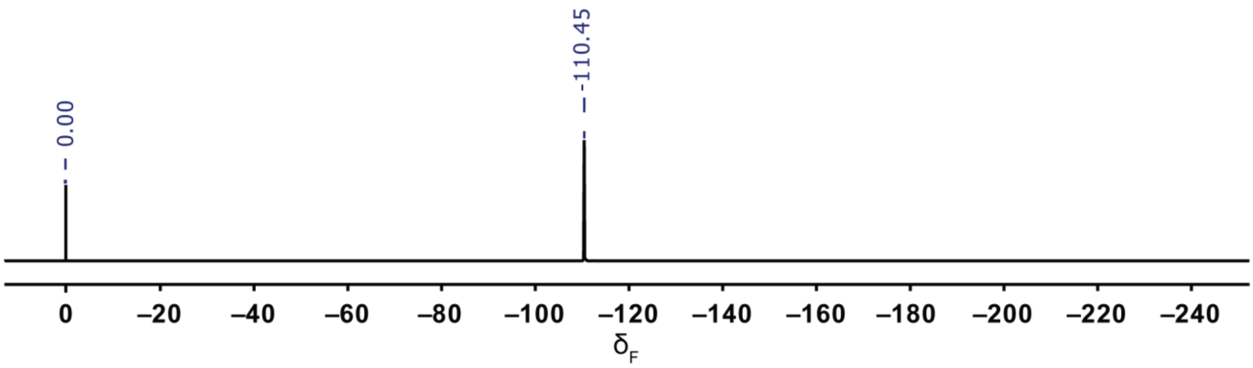
0.00
-110.45
0 −20 −40 −60 −80 −100 −120 −140 −160 −180 −200 −220 −240
δF

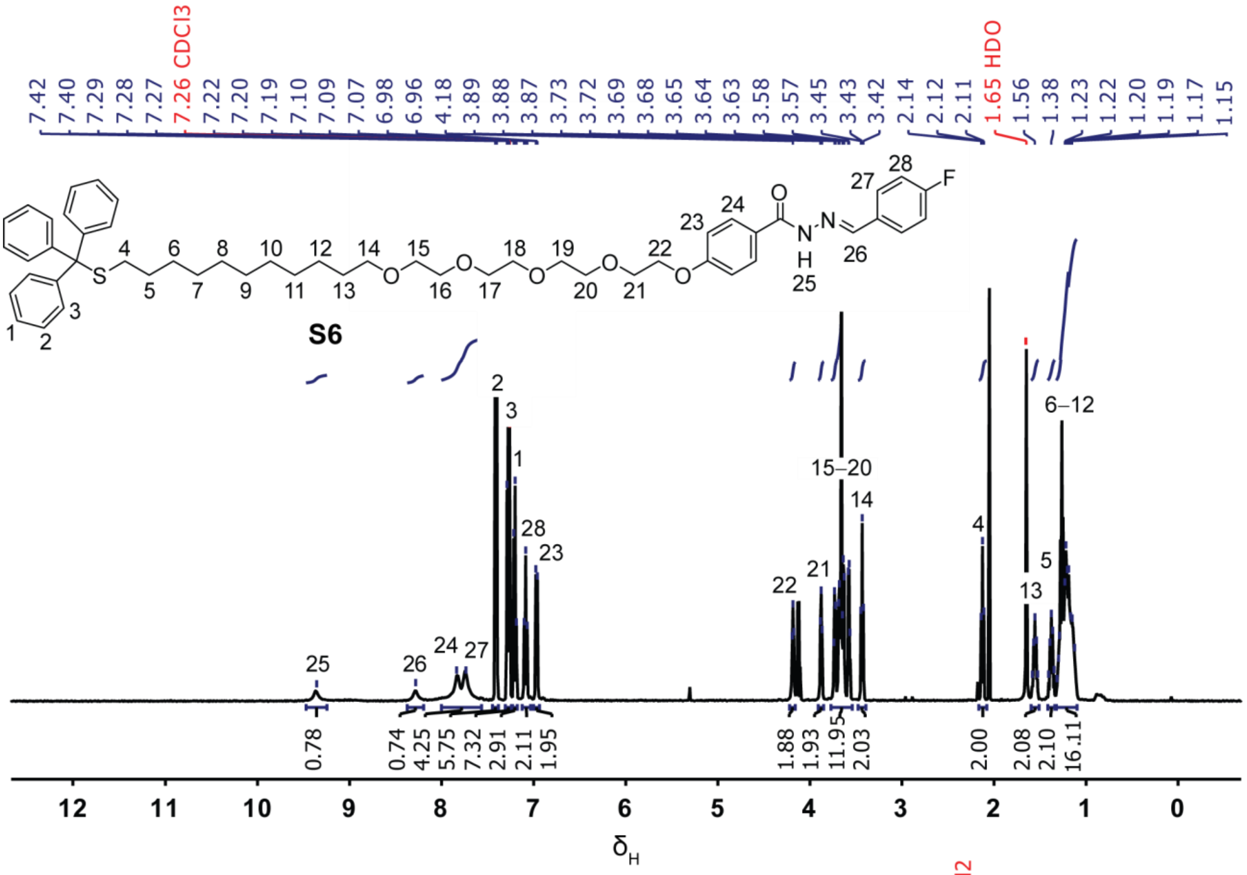
7.42 7.40 7.29 7.28 7.27 7.26 CDCl3 7.22 7.20 7.19 7.10 7.09 7.07 6.98 6.96 4.18 3.89 3.88 3.87 3.73 3.72 3.69 3.68 3.65 3.64 3.63 3.58 3.57 3.45 3.43 3.42 2.14 2.12 2.11 1.65 HDO 1.56 1.38 1.23 1.22 1.20 1.19 1.17 1.15
S6
25
26
24
27
2
3
1
28
23
22
21
15–20
14
4
13
5
6–12
0.78 0.74 4.25 5.75 7.32 2.91 2.11 1.95 1.88 1.93 11.95 2.03 2.00 2.08 2.10 16.11
12 11 10 9 8 7 6 5 4 3 2 1 0
δH

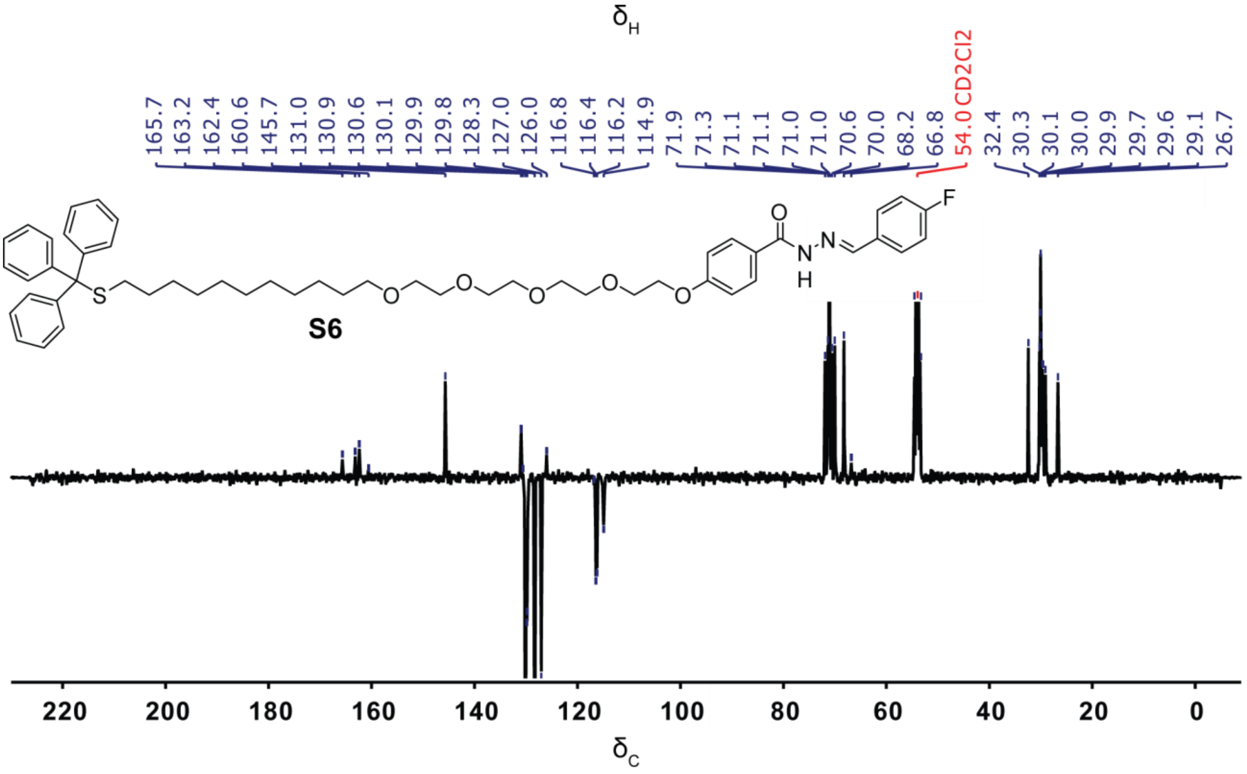
165.7 163.2 162.4 160.6 145.7 131.0 130.9 130.6 130.1 129.9 129.8 128.3 127.0 126.0 116.8 116.4 116.2 114.9 71.9 71.3 71.1 71.1 71.0 71.0 70.6 70.0 68.2 66.8 54.0 CD2Cl2 32.4 30.3 30.1 30.0 29.9 29.7 29.6 29.1 26.7
S6
220 200 180 160 140 120 100 80 60 40 20 0
δC

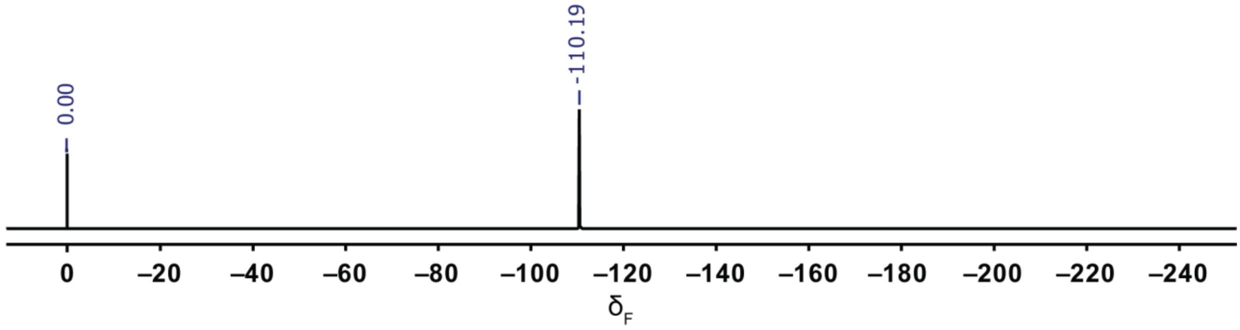
0.00
-110.19
0 −20 −40 −60 −80 −100 −120 −140 −160 −180 −200 −220 −240
δF

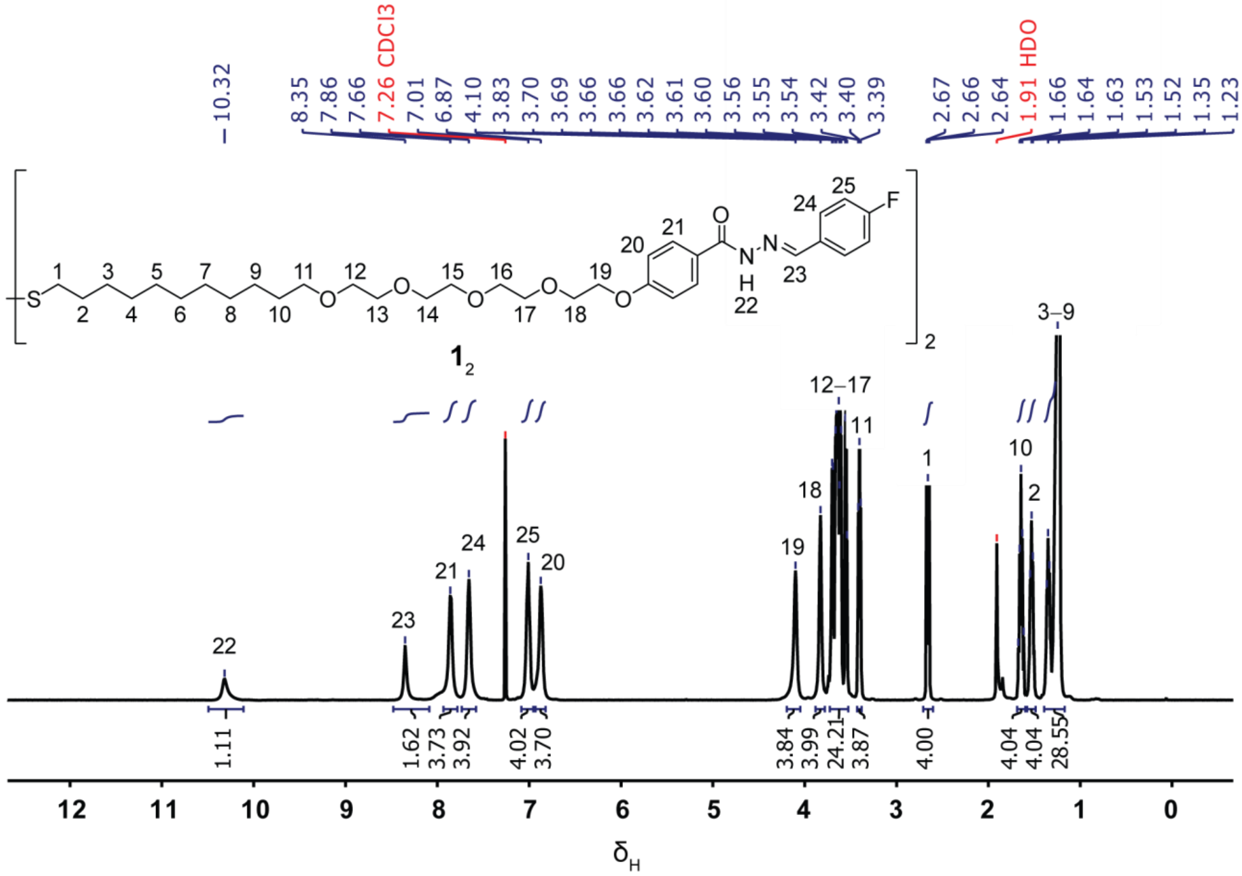
10.32
8.35 7.86 7.66 7.26 CDCl3 7.01 6.87 4.10 3.83 3.70 3.69 3.66 3.66 3.62 3.61 3.60 3.56 3.55 3.54 3.42 3.40 3.39
2.67 2.66 2.64 1.91 HDO 1.66 1.64 1.63 1.53 1.52 1.35 1.23
1₂
22 23 21 24 25 20 19 18 12–17 11 1 10 2 3–9
1.11 1.62 3.73 3.92 4.02 3.70 3.84 3.99 24.21 3.87 4.00 4.04 4.04 28.55
12 11 10 9 8 7 6 5 4 3 2 1 0
δH

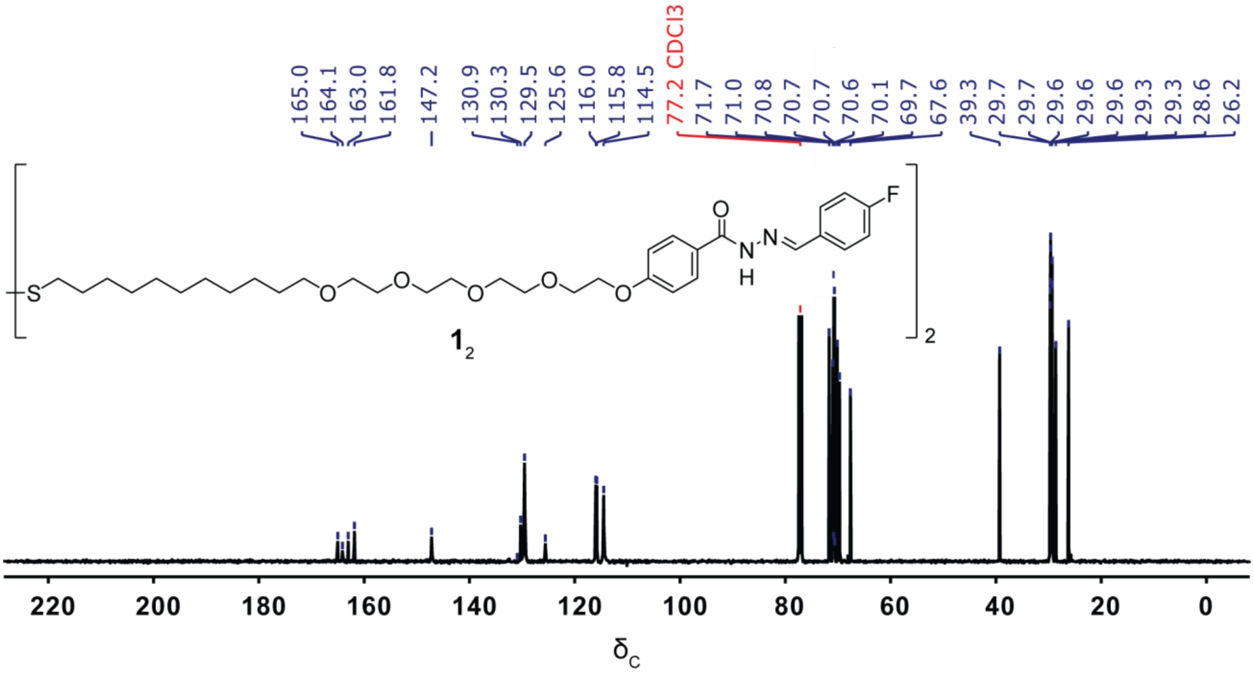
165.0 164.1 163.0 161.8 147.2 130.9 130.3 129.5 125.6 116.0 115.8 114.5 77.2 CDCl3 71.7 71.0 70.8 70.7 70.7 70.6 70.1 69.7 67.6 39.3 29.7 29.7 29.6 29.6 29.6 29.3 29.3 28.6 26.2
1₂
220 200 180 160 140 120 100 80 60 40 20 0
δC

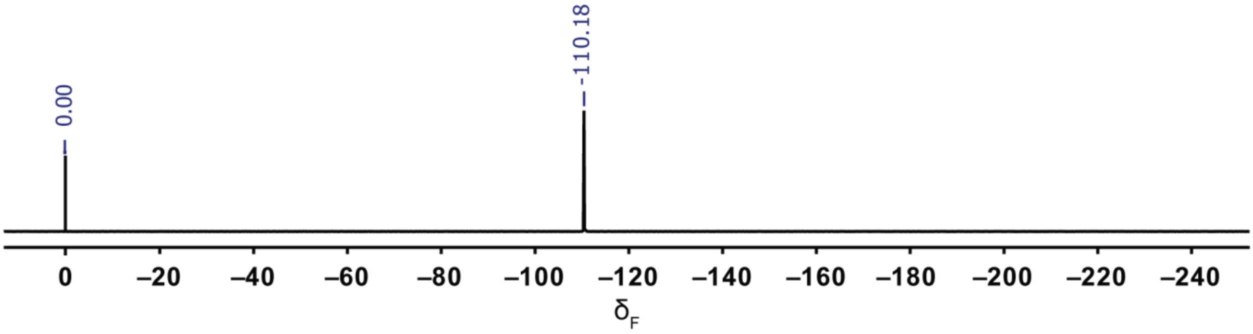
0.00
-110.18
0 −20 −40 −60 −80 −100 −120 −140 −160 −180 −200 −220 −240
δF

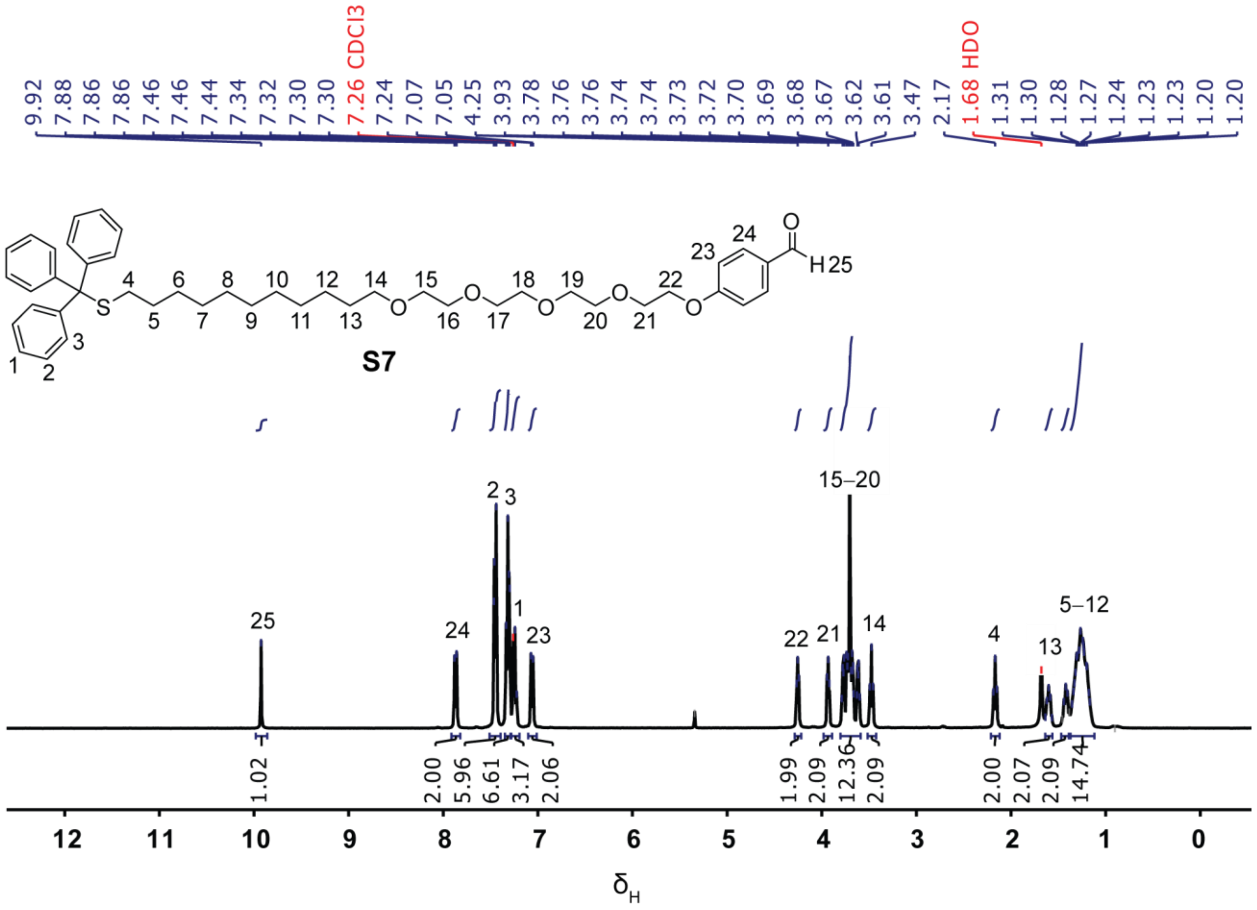
9.92 7.88 7.86 7.86 7.46 7.46 7.44 7.34 7.32 7.30 7.30 7.26 CDCl3 7.24 7.07 7.05 4.25 3.93 3.78 3.76 3.76 3.74 3.74 3.73 3.73 3.72 3.70 3.69 3.68 3.67 3.62 3.61 3.47 2.17 1.68 HDO 1.31 1.30 1.28 1.27 1.24 1.23 1.23 1.20 1.20
S7
25
24
2 3
1
23
22
21
15–20
14
4
13
5–12
1.02 2.00 5.96 6.61 3.17 2.06 1.99 2.09 12.36 2.09 2.00 2.07 2.09 14.74
12 11 10 9 8 7 6 5 4 3 2 1 0
δH

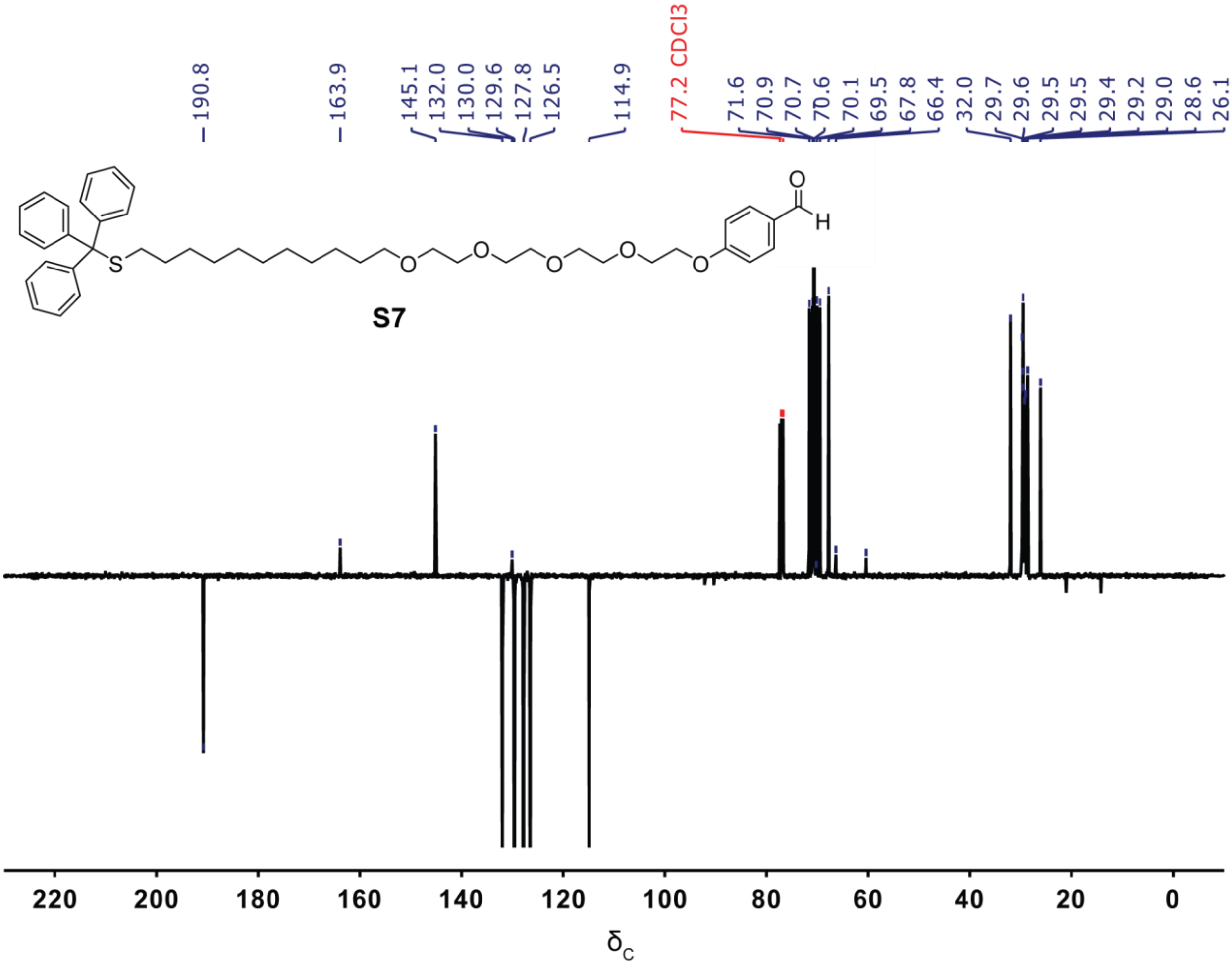
190.8 163.9 145.1 132.0 130.0 129.6 127.8 126.5 114.9 77.2 CDCl3 71.6 70.9 70.7 70.6 70.1 69.5 67.8 66.4 32.0 29.7 29.6 29.5 29.5 29.4 29.2 29.0 28.6 26.1
S7
220 200 180 160 140 120 100 80 60 40 20 0
δC

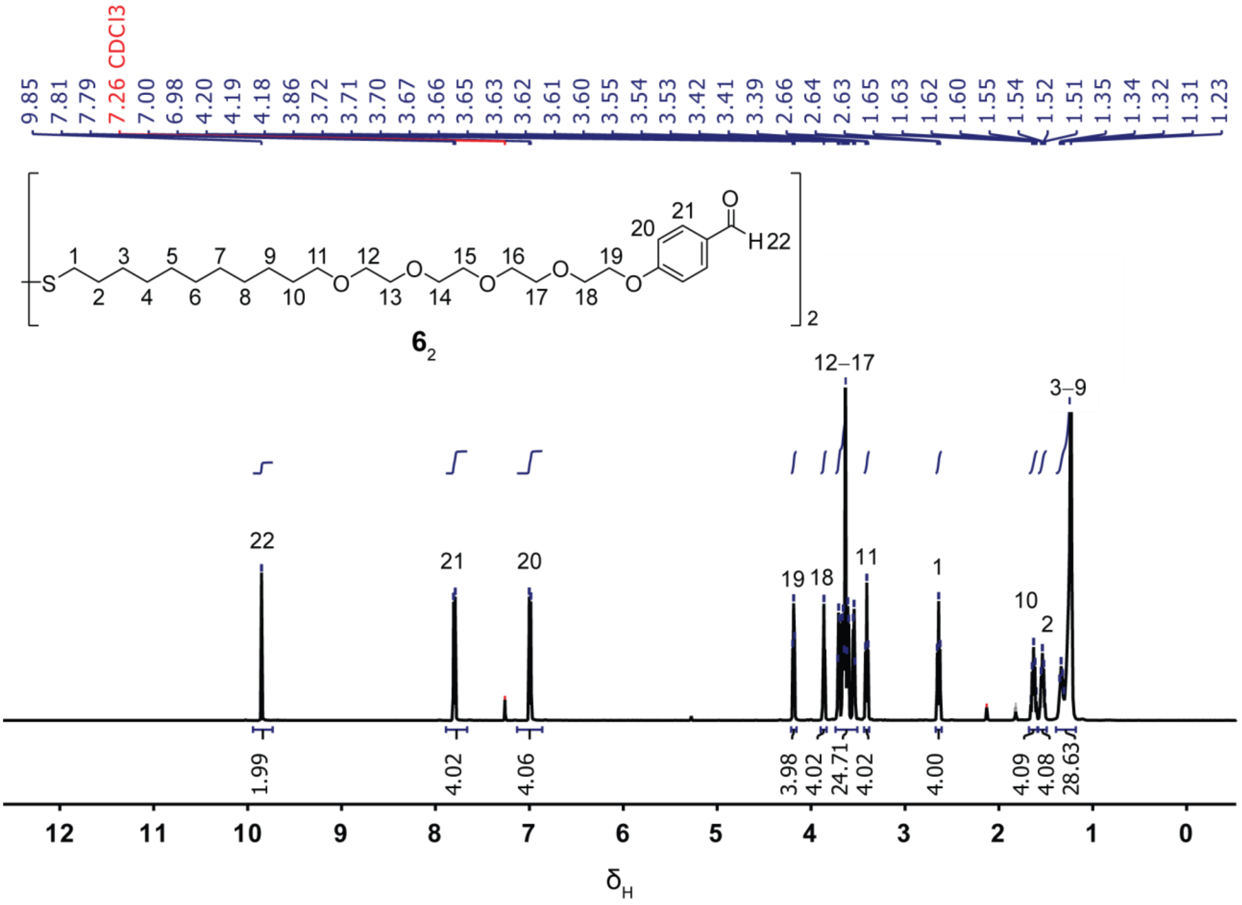
9.85 7.81 7.79 7.26 CDCl3 7.00 6.98 4.20 4.19 4.18 3.86 3.72 3.71 3.70 3.67 3.66 3.65 3.63 3.62 3.61 3.60 3.55 3.54 3.53 3.42 3.41 3.39 2.66 2.64 2.63 1.65 1.63 1.62 1.60 1.55 1.54 1.52 1.51 1.35 1.34 1.32 1.31 1.23
S
O
H
1 2 3 4 5 6 7 8 9 10 11 12 13 14 15 16 17 18 19 20 21 22
6
2
12–17
3–9
22
21
20
19 18
11
1
10
2
1.99
4.02
4.06
3.98
4.02
24.71
4.02
4.00
4.09
4.08
28.63
12 11 10 9 8 7 6 5 4 3 2 1 0
δH

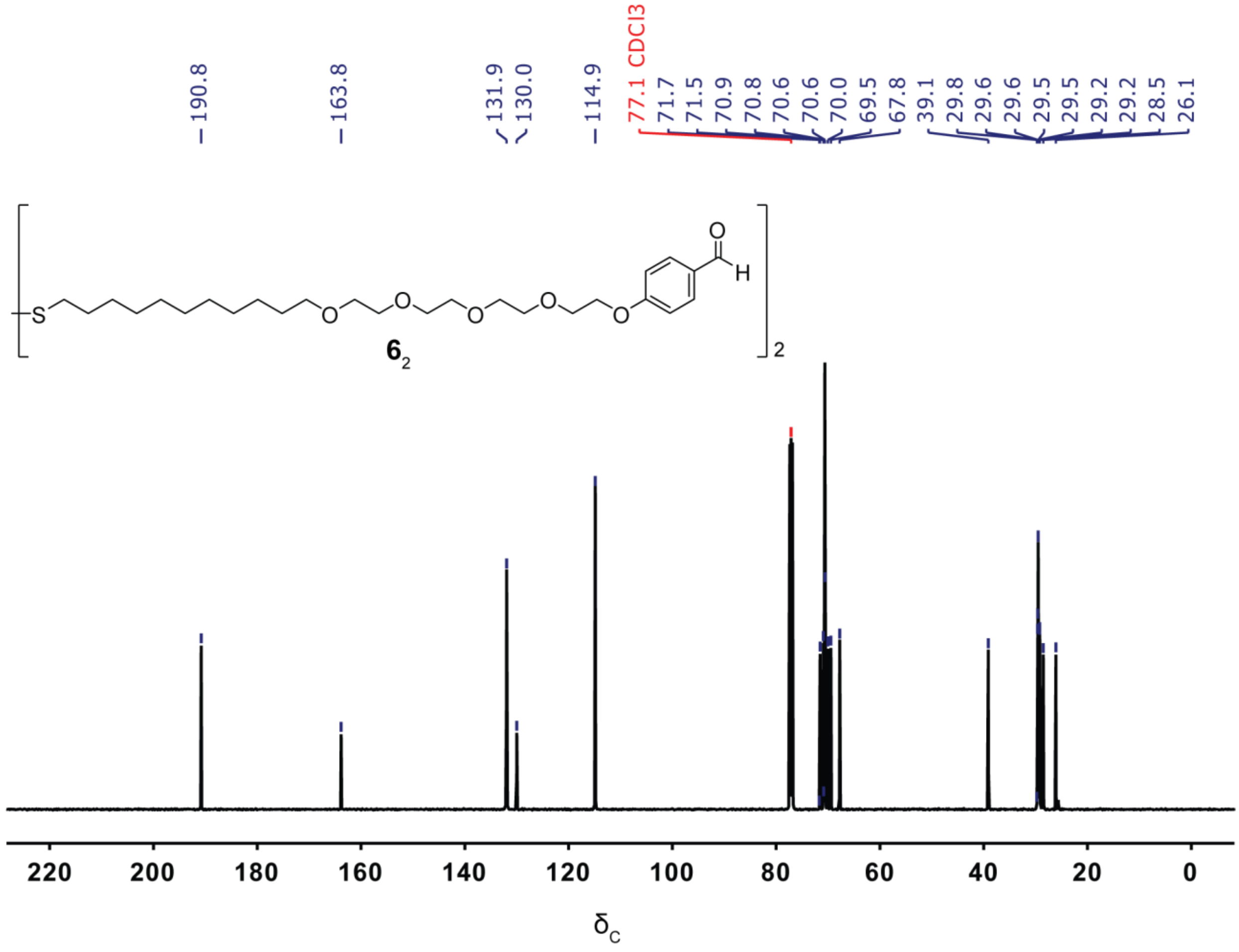
190.8
163.8
131.9
130.0
114.9
77.1 CDCl3
71.7 71.5 70.9 70.8 70.6 70.6 70.0 69.5 67.8 39.1 29.8 29.6 29.6 29.5 29.5 29.2 29.2 28.5 26.1
S
O
H
6
2
220 200 180 160 140 120 100 80 60 40 20 0
δC

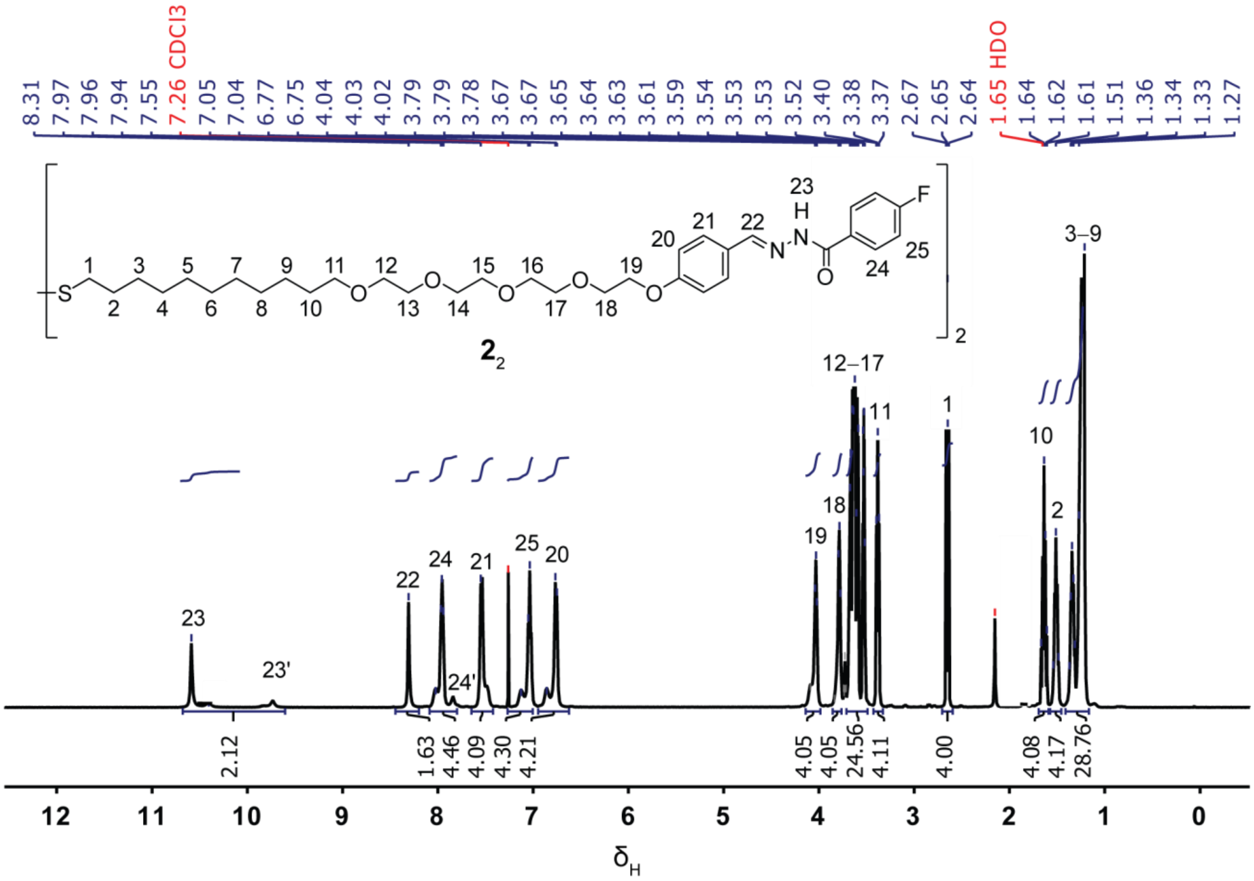
8.31 7.97 7.96 7.94 7.55 7.26 CDCl3 7.05 7.04 6.77 6.75 4.04 4.03 4.02 3.79 3.79 3.78 3.67 3.67 3.65 3.64 3.63 3.61 3.59 3.54 3.53 3.53 3.52 3.40 3.38 3.37 2.67 2.65 2.64 1.65 HDO 1.64 1.62 1.61 1.51 1.36 1.34 1.33 1.27
2₂
23
22
24
21
25
20
19
18
12–17
11
1
10
2
3–9
23'
24'
2.12
1.63
4.46
4.09
4.30
4.21
4.05
4.05
24.56
4.11
4.00
4.08
4.17
28.76
δH

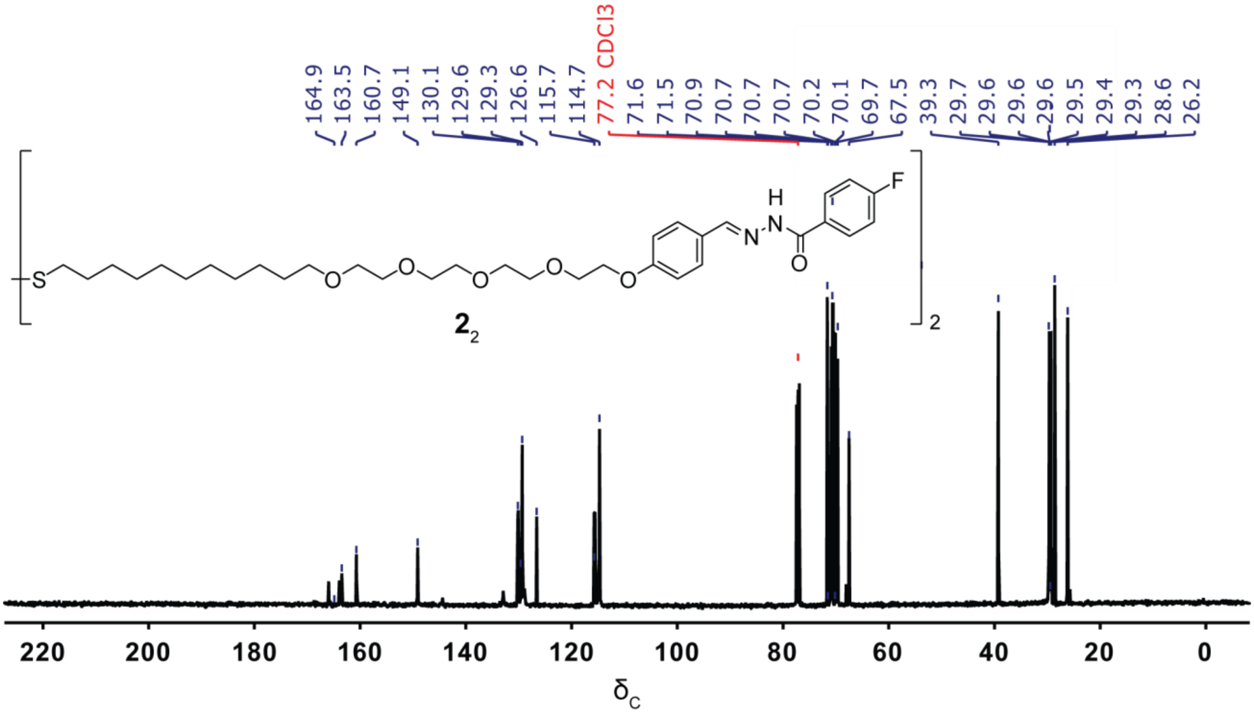
164.9 163.5 160.7 149.1 130.1 129.6 129.3 126.6 115.7 114.7 77.2 CDCl3 71.6 71.5 70.9 70.7 70.7 70.7 70.2 70.1 69.7 67.5 39.3 29.7 29.6 29.6 29.6 29.5 29.4 29.3 28.6 26.2
2₂
δC

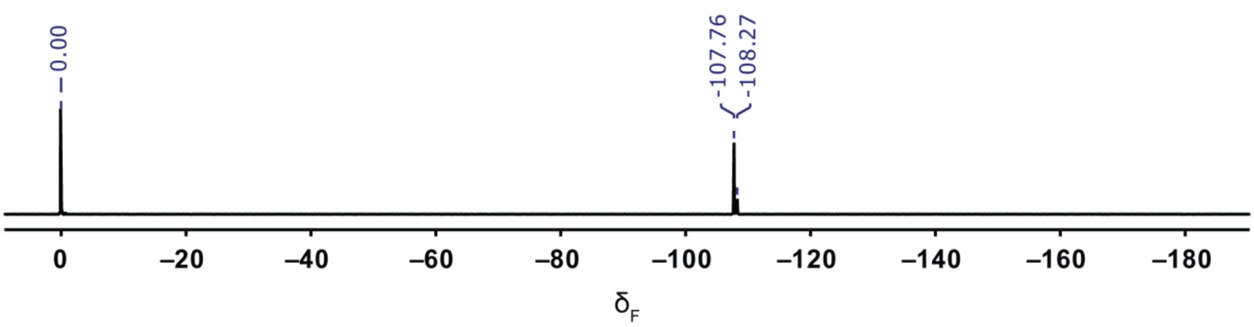
0.00
-107.76
-108.27
δF

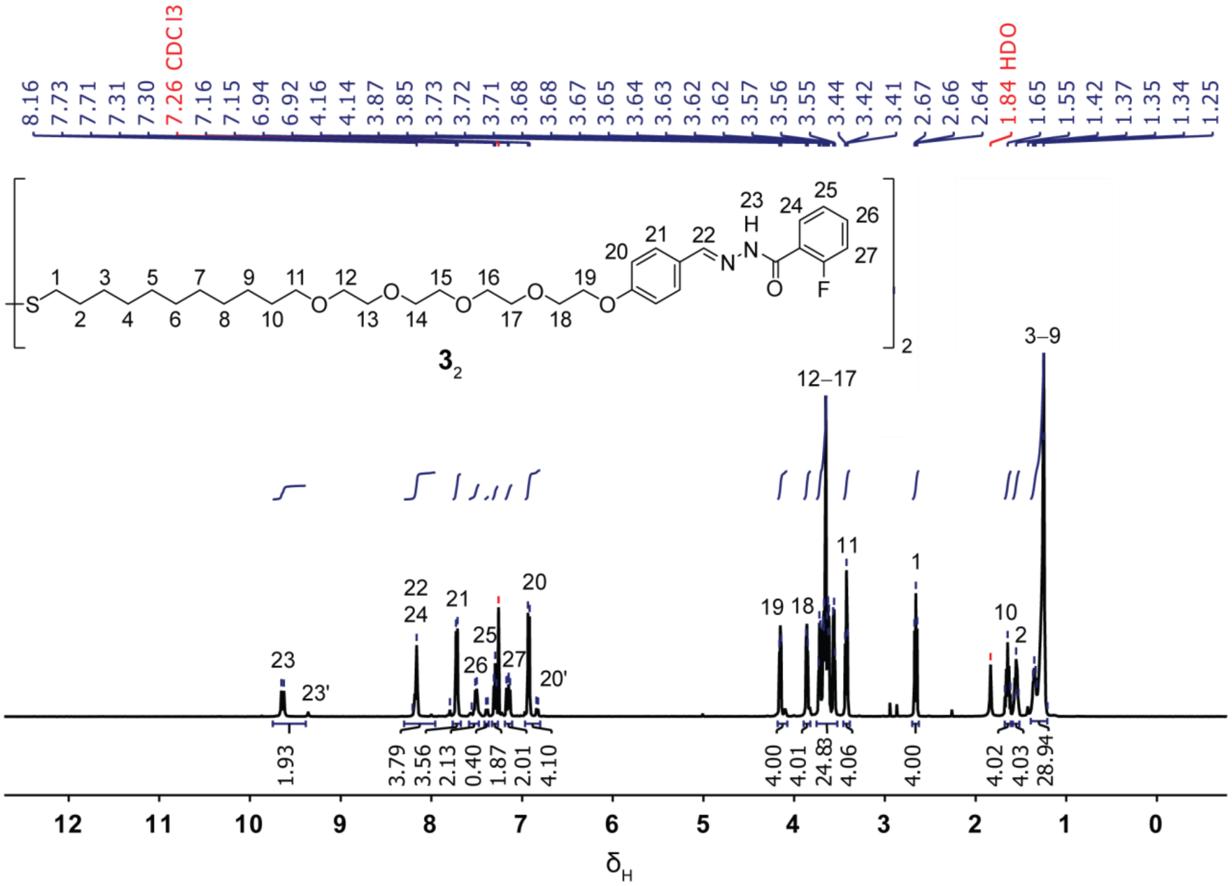
8.16 7.73 7.71 7.31 7.30 7.26 CDCl3 7.16 7.15 6.94 6.92 4.16 4.14 3.87 3.85 3.73 3.72 3.71 3.68 3.68 3.67 3.65 3.64 3.63 3.62 3.62 3.57 3.56 3.55 3.44 3.42 3.41 2.67 2.66 2.64 1.84 HDO 1.65 1.55 1.42 1.37 1.35 1.34 1.25
3_2
1.93 3.79 3.56 2.13 0.40 1.87 2.01 4.10 4.00 4.01 24.83 4.06 4.00 4.02 4.03 28.94
δH

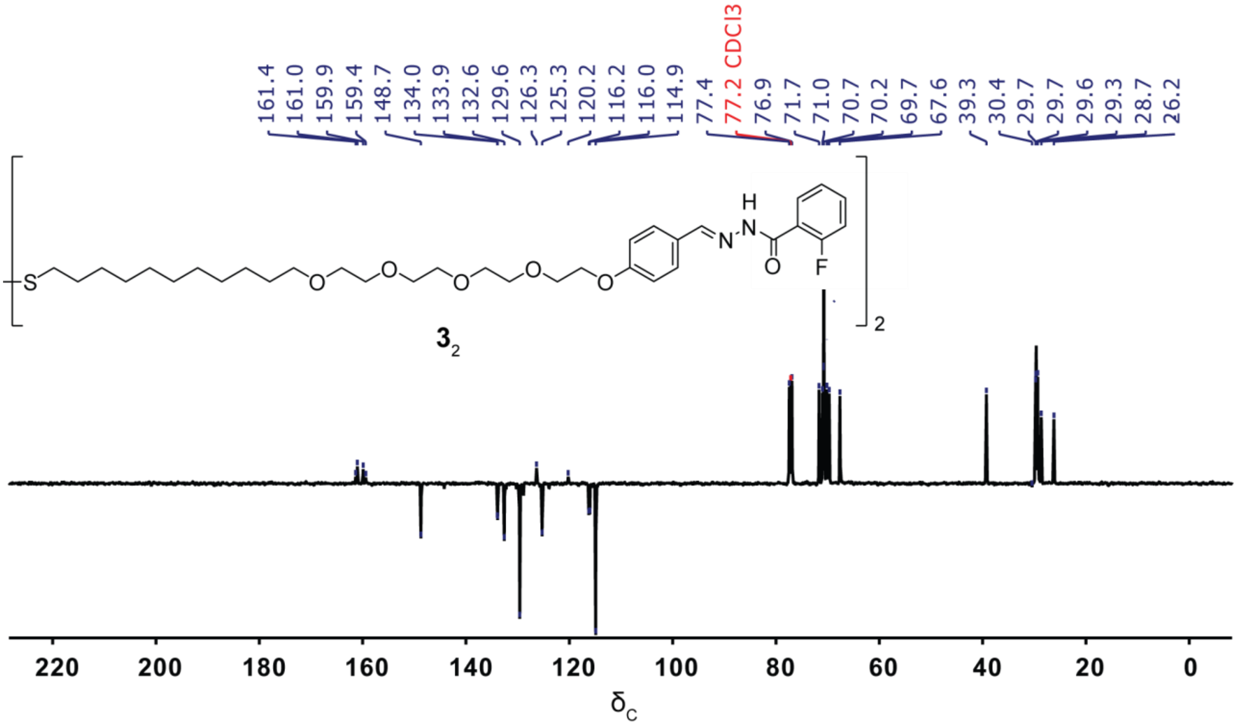
161.4 161.0 159.9 159.4 148.7 134.0 133.9 132.6 129.6 126.3 125.3 120.2 116.2 116.0 114.9 77.4 77.2 CDCl3 76.9 71.7 71.0 70.7 70.2 69.7 67.6 39.3 30.4 29.7 29.7 29.6 29.3 28.7 26.2
3_2
δC

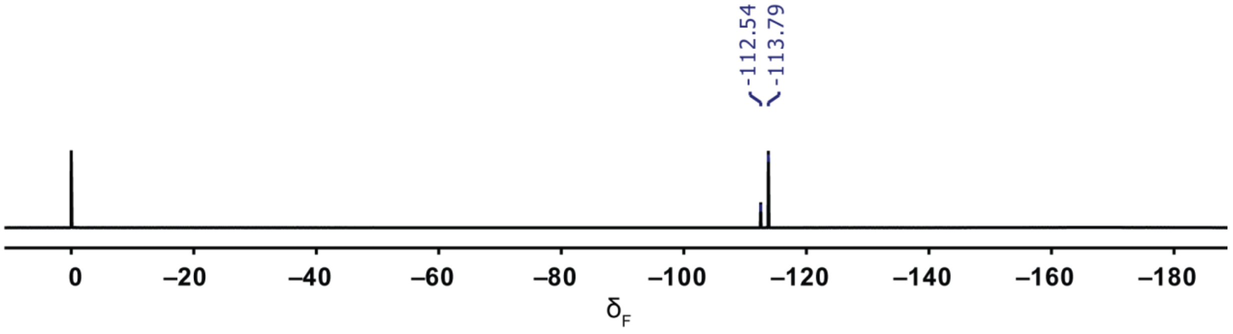
-112.54
-113.79
δF

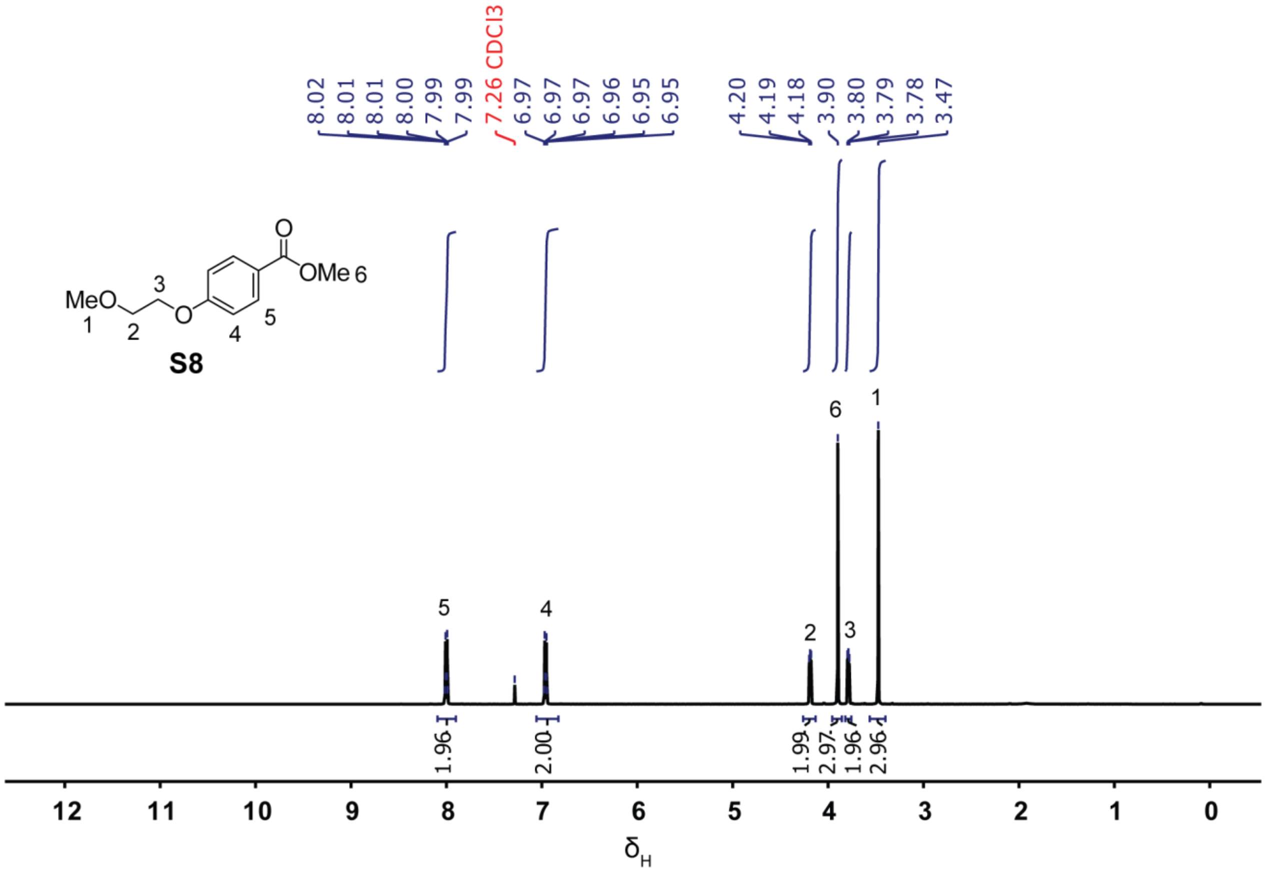
8.02
8.01
8.01
8.00
7.99
7.99
7.26 CDCl3
6.97
6.97
6.97
6.96
6.95
6.95
4.20
4.19
4.18
3.90
3.80
3.79
3.78
3.47
MeO
OMe 6
O
1
2
3
4
5
S8
1.96
2.00
1.99
2.97
1.96
2.96
12
11
10
9
8
7
6
5
4
3
2
1
0
δH

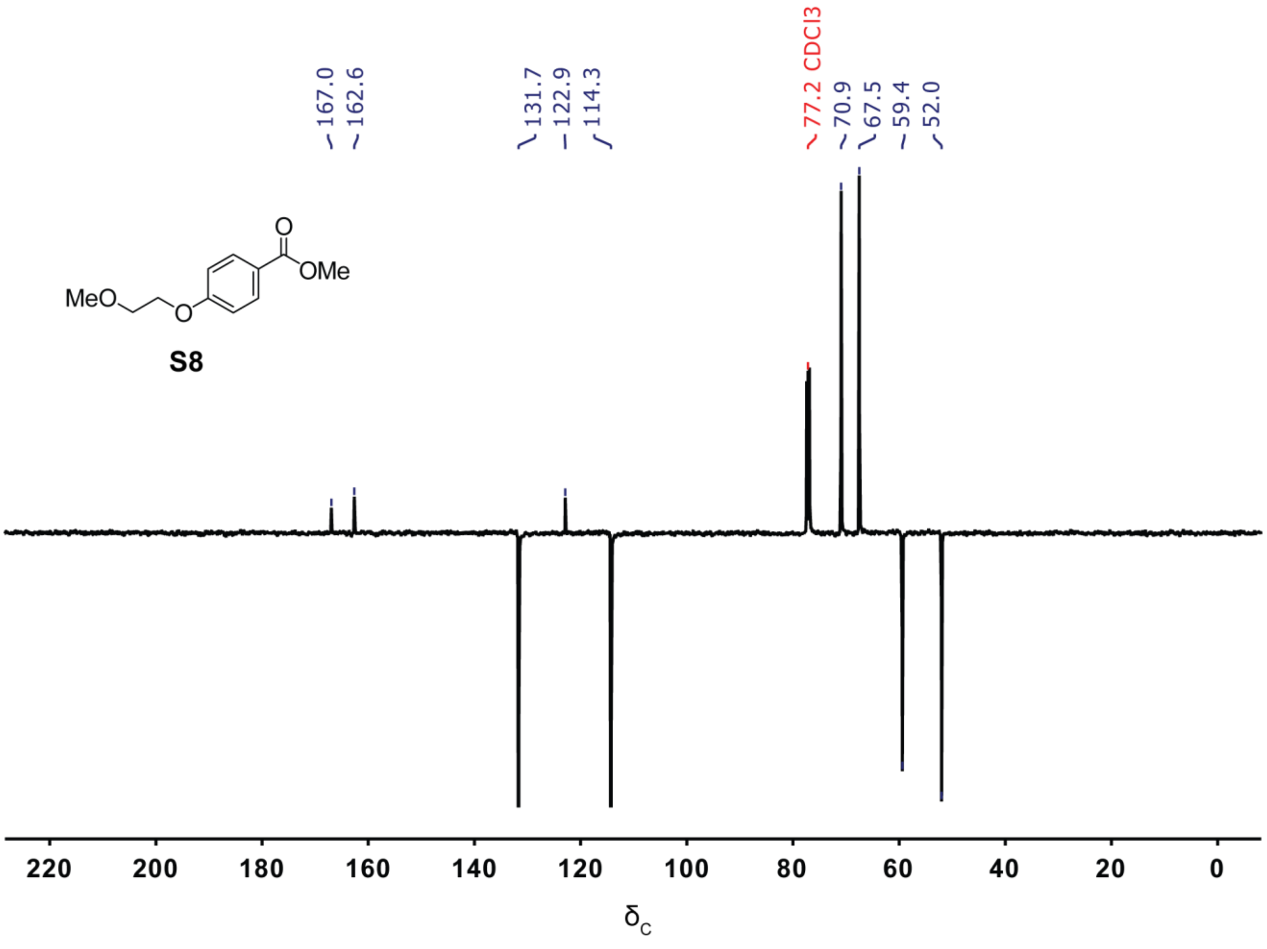
167.0
162.6
131.7
122.9
114.3
77.2 CDCl3
70.9
67.5
59.4
52.0
MeO
OMe
O
S8
220
200
180
160
140
120
100
80
60
40
20
0
δC

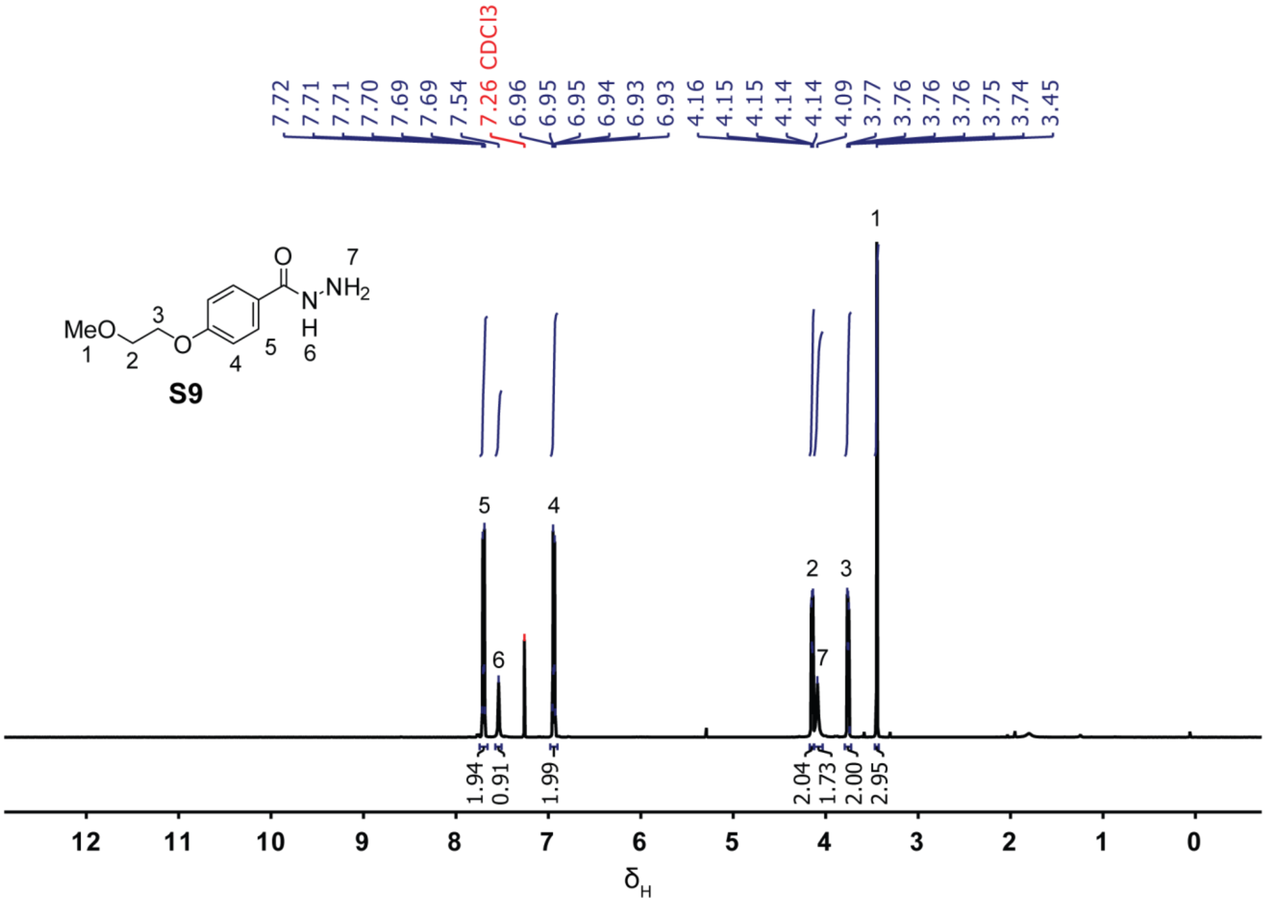
7.72
7.71
7.71
7.70
7.69
7.69
7.54
7.26 CDCl3
6.96
6.95
6.95
6.94
6.93
6.93
4.16
4.15
4.15
4.14
4.14
4.09
3.77
3.76
3.76
3.76
3.75
3.74
3.45
MeO
O
NH2
N
H
S9
1.94
0.91
1.99
2.04
1.73
2.00
2.95
12
11
10
9
8
7
6
5
4
3
2
1
0
δH

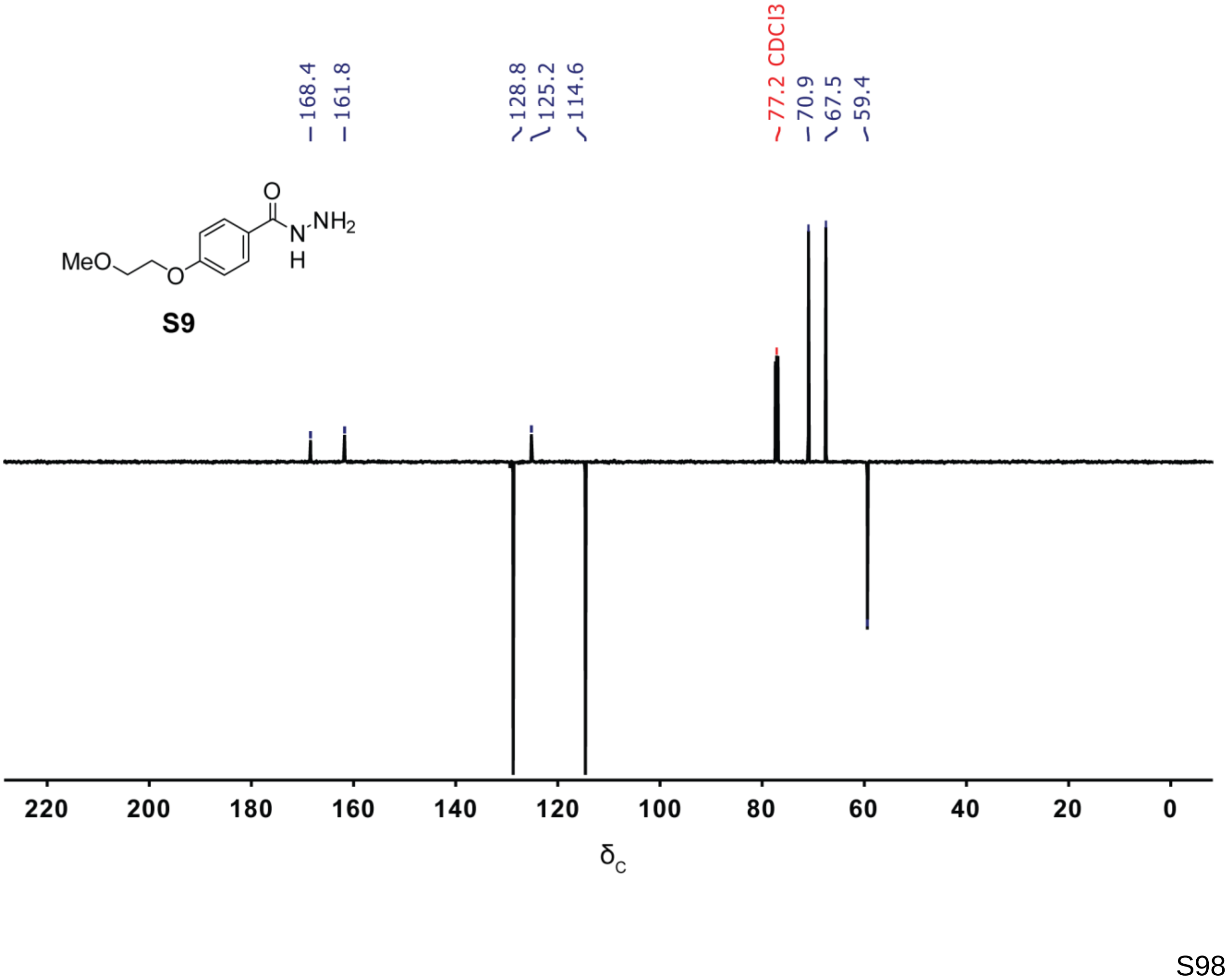
168.4
161.8
128.8
125.2
114.6
77.2 CDCl3
70.9
67.5
59.4
MeO
O
NH2
N
H
S9
220
200
180
160
140
120
100
80
60
40
20
0
δC

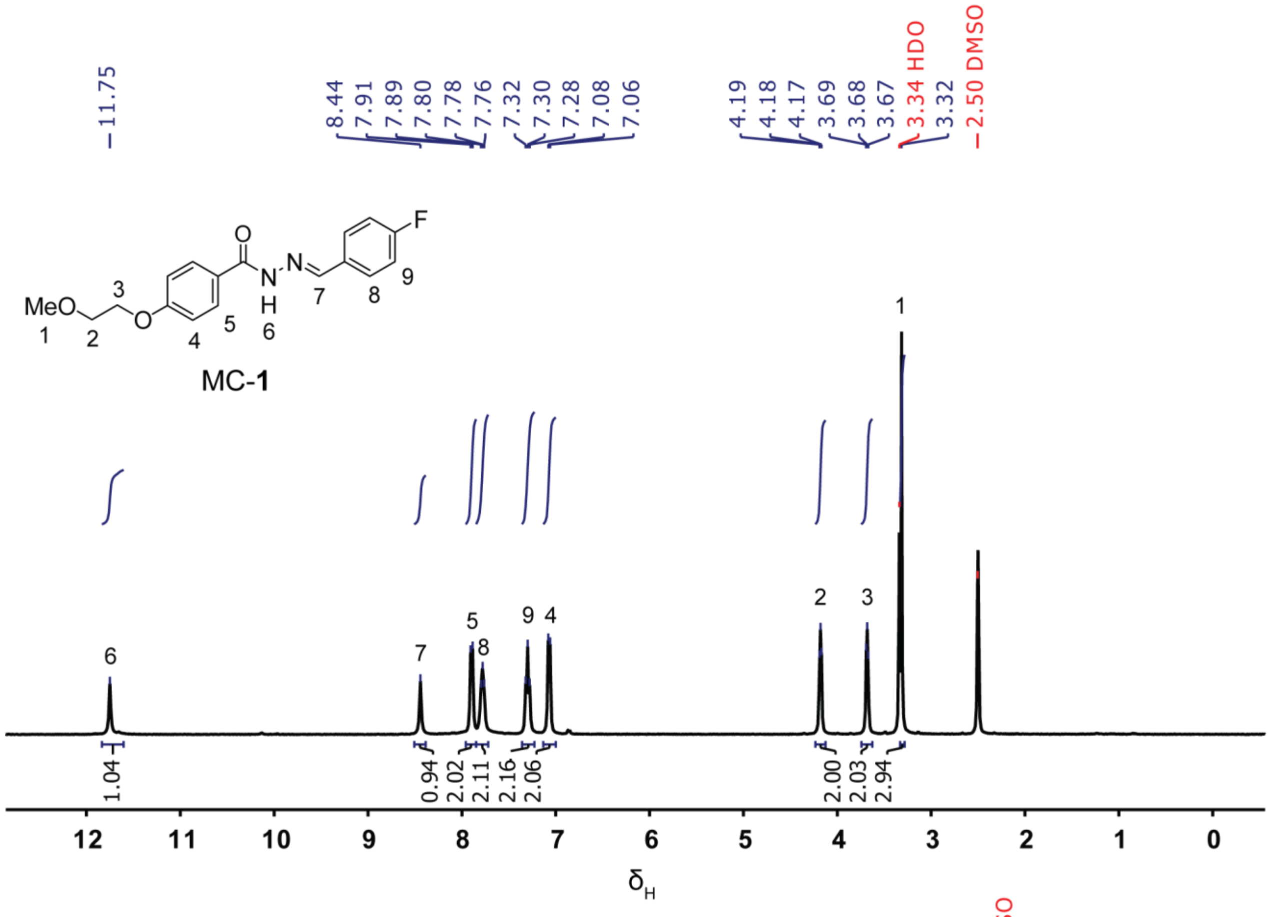
11.75
8.44 7.91 7.89 7.80 7.78 7.76 7.32 7.30 7.28 7.08 7.06
4.19 4.18 4.17 3.69 3.68 3.67 3.34 HDO 3.32 2.50 DMSO
MC-1
1.04 0.94 2.02 2.11 2.16 2.06 2.00 2.03 2.94
δH

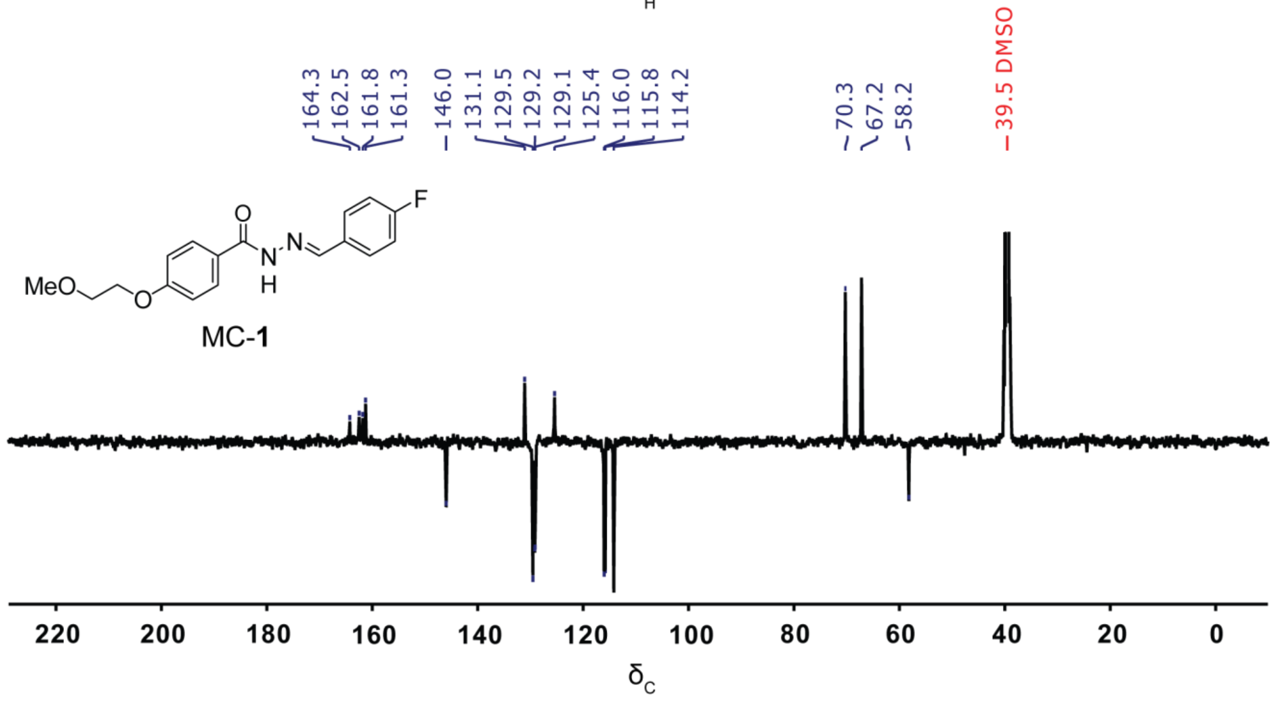
164.3 162.5 161.8 161.3 146.0 131.1 129.5 129.2 129.1 125.4 116.0 115.8 114.2
70.3 67.2 58.2 39.5 DMSO
MC-1
δC

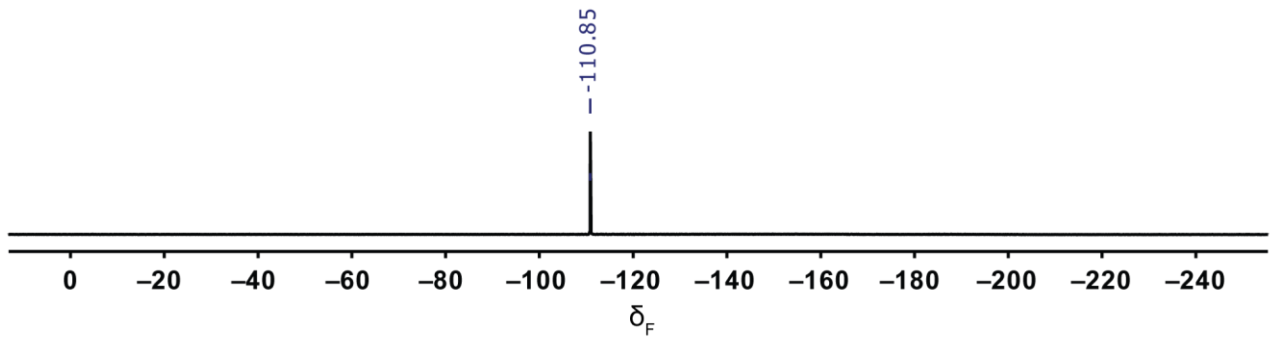
-110.85
δF

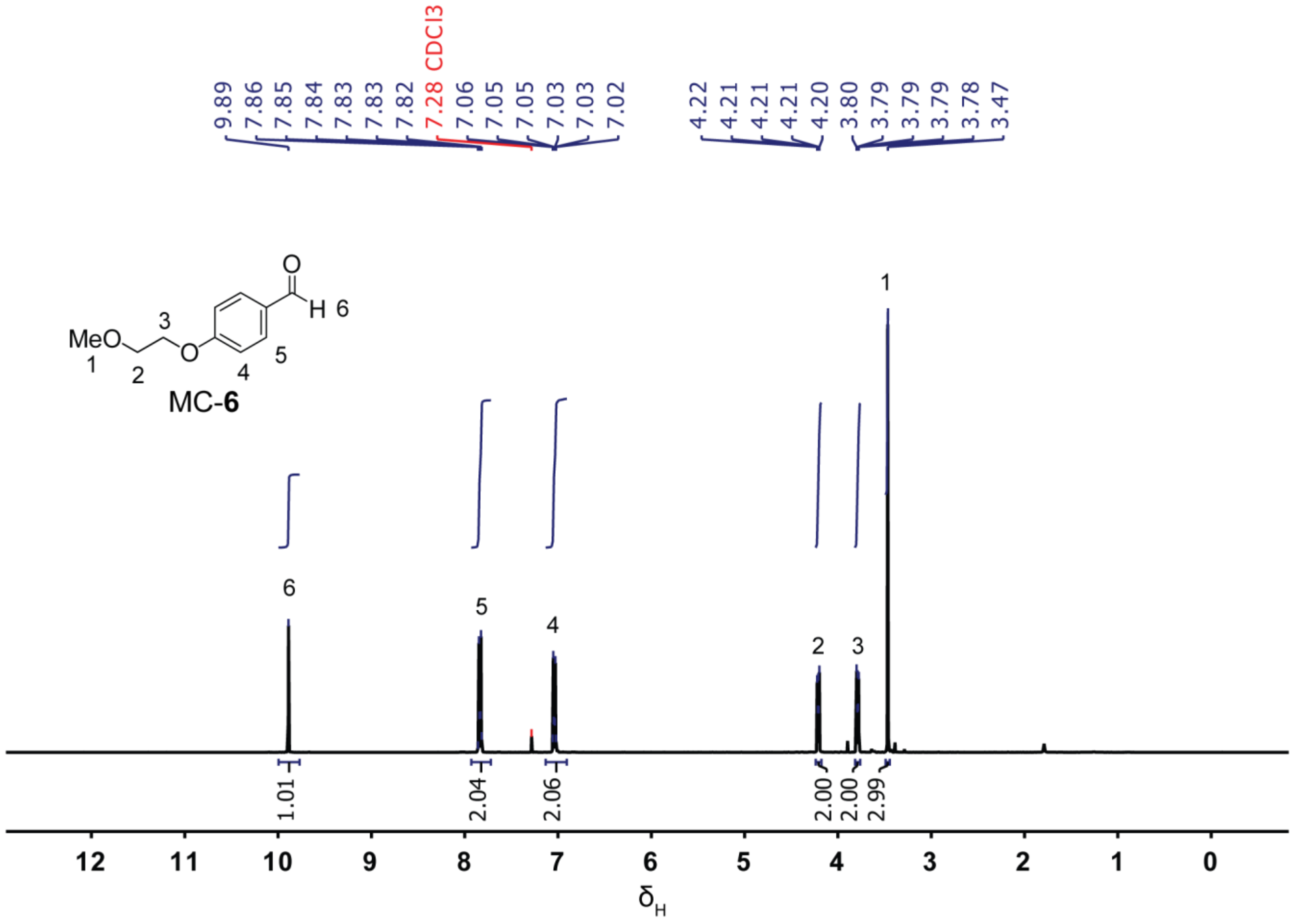
9.89
7.86
7.85
7.84
7.83
7.83
7.82
7.28 CDCl3
7.06
7.05
7.05
7.03
7.03
7.02
4.22
4.21
4.21
4.21
4.20
3.80
3.79
3.79
3.79
3.78
3.47
MeO
H 6
MC-6
1.01
2.04
2.06
2.00
2.00
2.99
12
11
10
9
8
7
6
5
4
3
2
1
0
δ_H

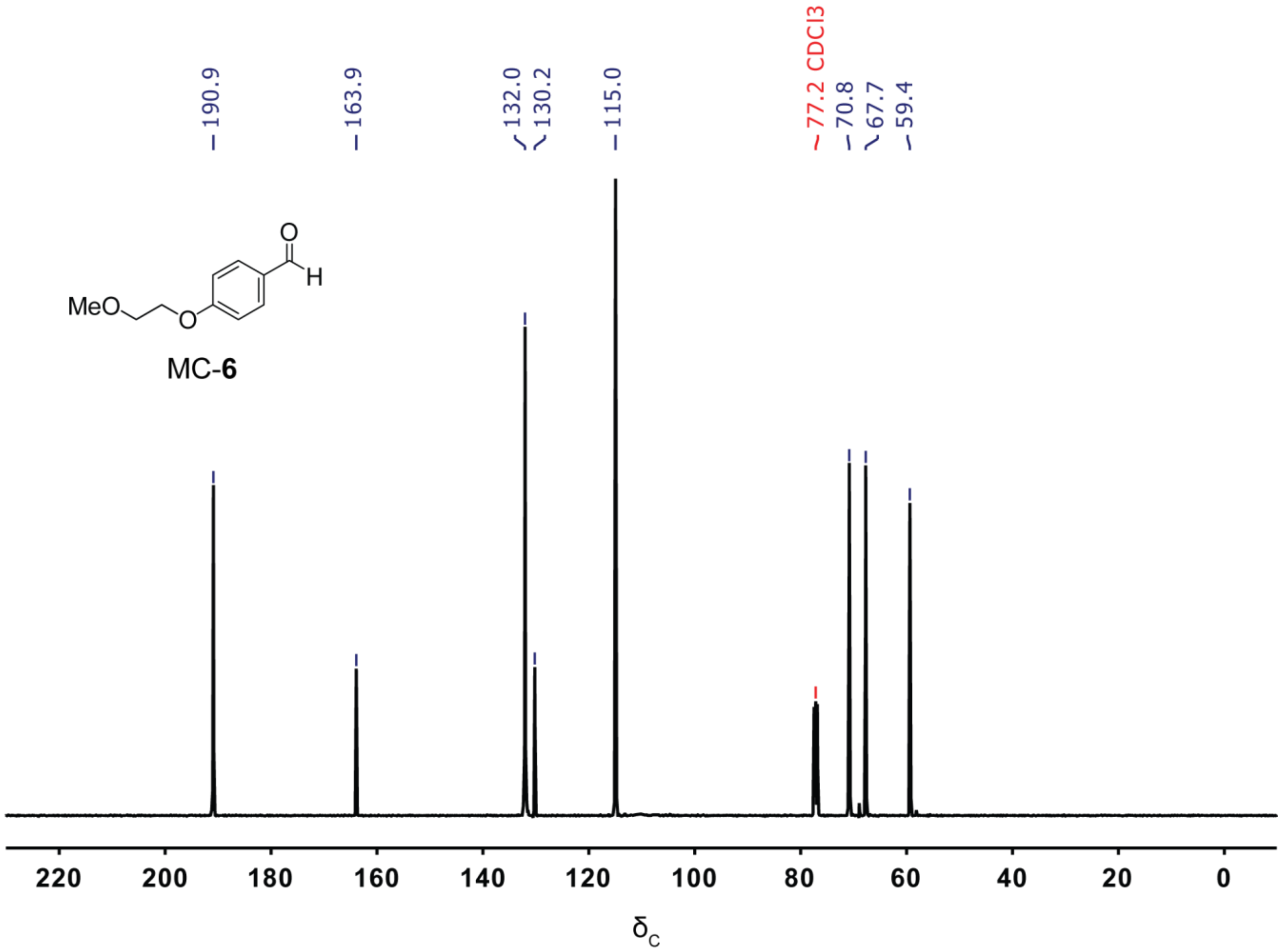
190.9
163.9
132.0
130.2
115.0
77.2 CDCl3
70.8
67.7
59.4
MeO
H
MC-6
220
200
180
160
140
120
100
80
60
40
20
0
δ_C

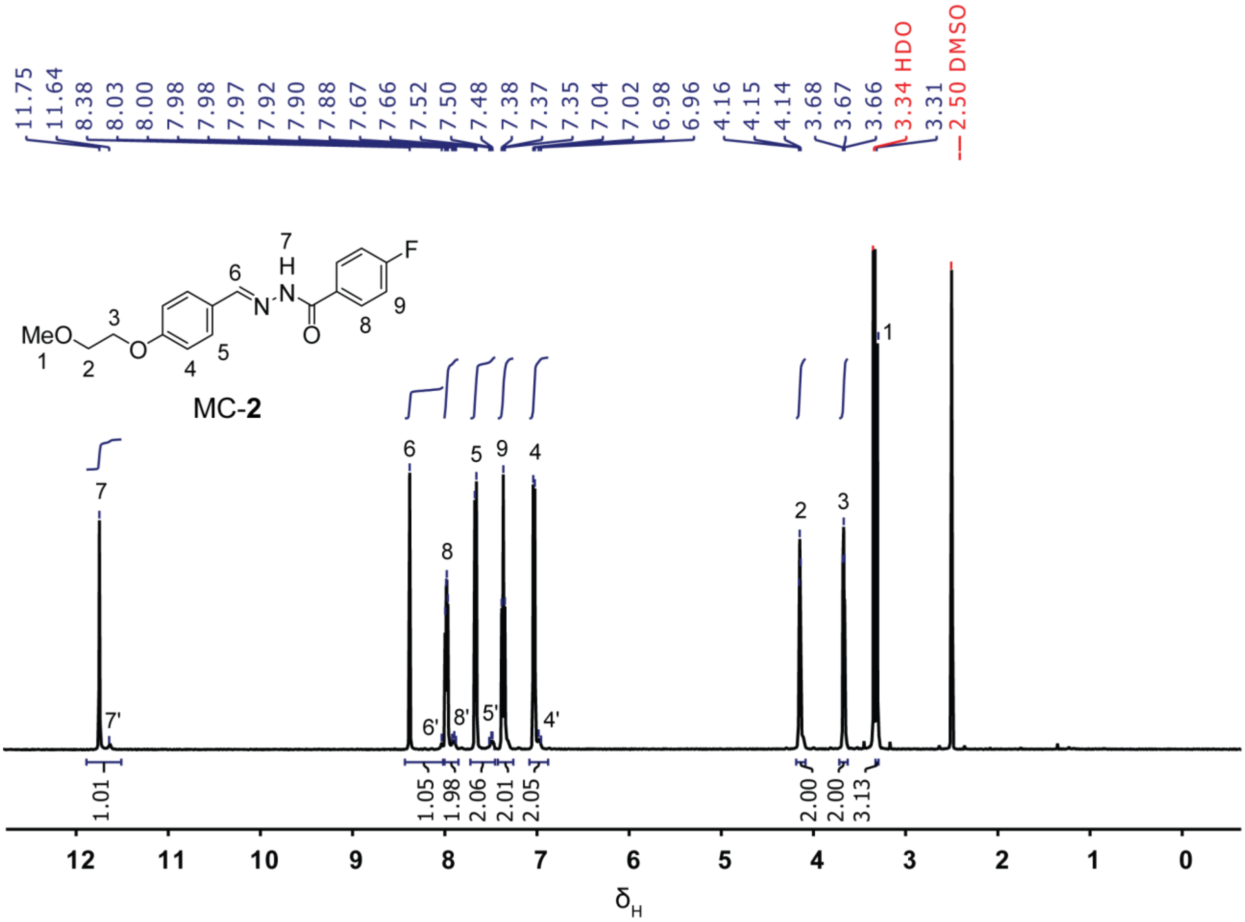
11.75 11.64 8.38 8.03 8.00 7.98 7.98 7.97 7.92 7.90 7.88 7.67 7.66 7.52 7.50 7.48 7.38 7.37 7.35 7.04 7.02 6.98 6.96 4.16 4.15 4.14 3.68 3.67 3.66 3.34 HDO 3.31 2.50 DMSO
MeO
F
MC-2
1.01 1.05 1.98 2.06 2.01 2.05 2.00 2.00 3.13
12 11 10 9 8 7 6 5 4 3 2 1 0
δH

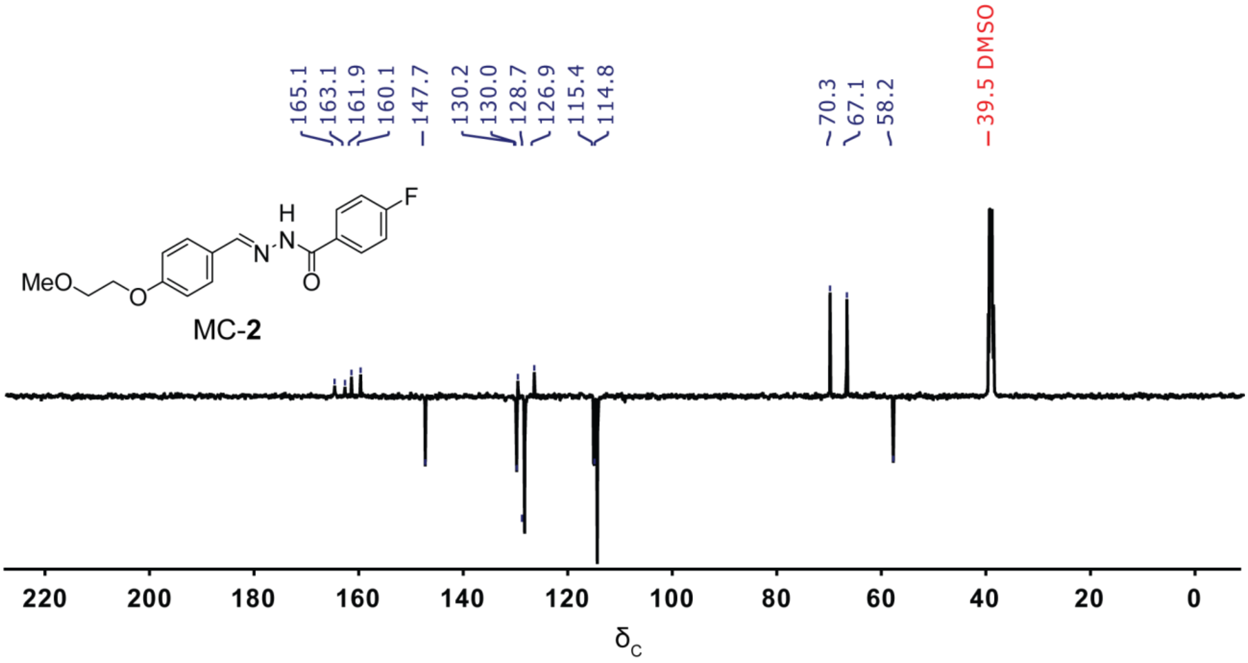
165.1 163.1 161.9 160.1 147.7 130.2 130.0 128.7 126.9 115.4 114.8 70.3 67.1 58.2 39.5 DMSO
MeO
F
MC-2
220 200 180 160 140 120 100 80 60 40 20 0
δC

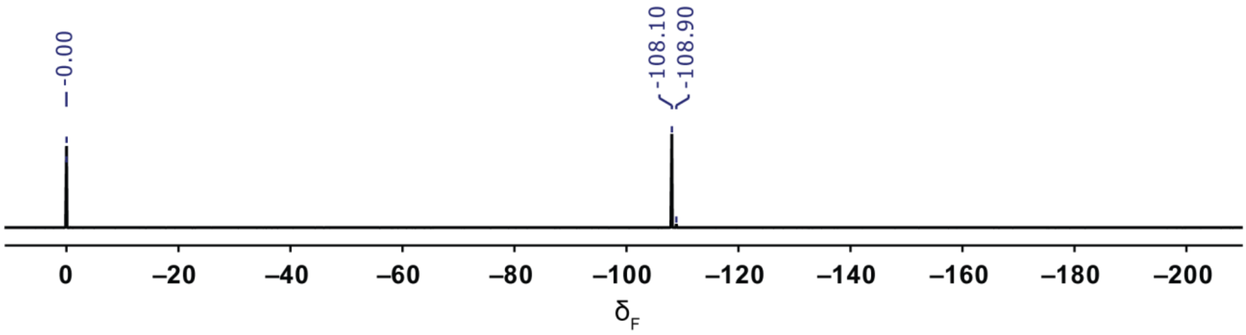
-0.00
-108.10 -108.90
0 −20 −40 −60 −80 −100 −120 −140 −160 −180 −200
δF

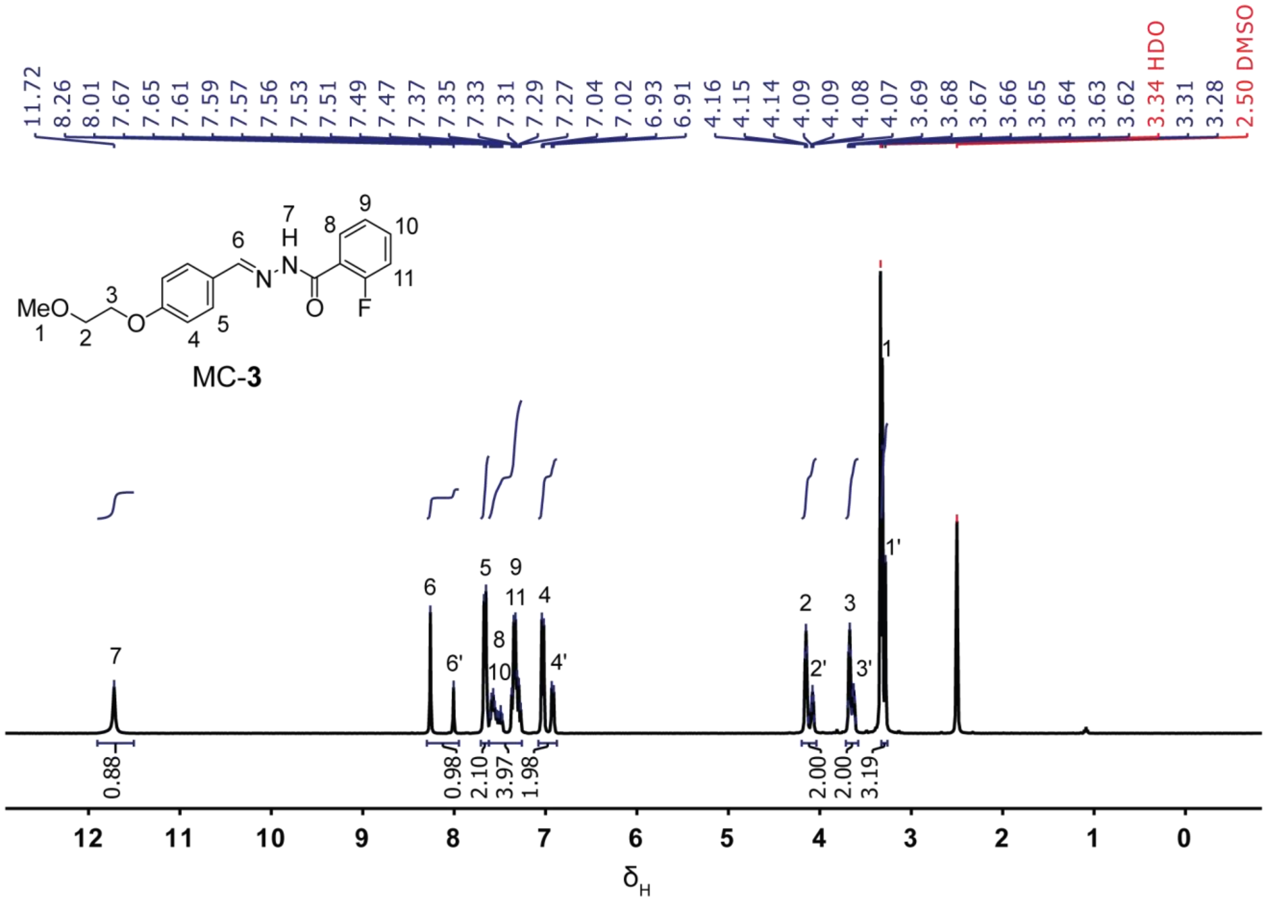
11.72
8.26
8.01
7.67
7.65
7.61
7.59
7.57
7.56
7.53
7.51
7.49
7.47
7.37
7.35
7.33
7.31
7.29
7.27
7.04
7.02
6.93
6.91
4.16
4.15
4.14
4.09
4.09
4.08
4.07
3.69
3.68
3.67
3.66
3.65
3.64
3.63
3.62
3.34 HDO
3.31
3.28
2.50 DMSO
MeO
O
H
N
N
O
F
MC-3
0.88
0.98
2.10
3.97
1.98
2.00
2.00
3.19
12 11 10 9 8 7 6 5 4 3 2 1 0
δH

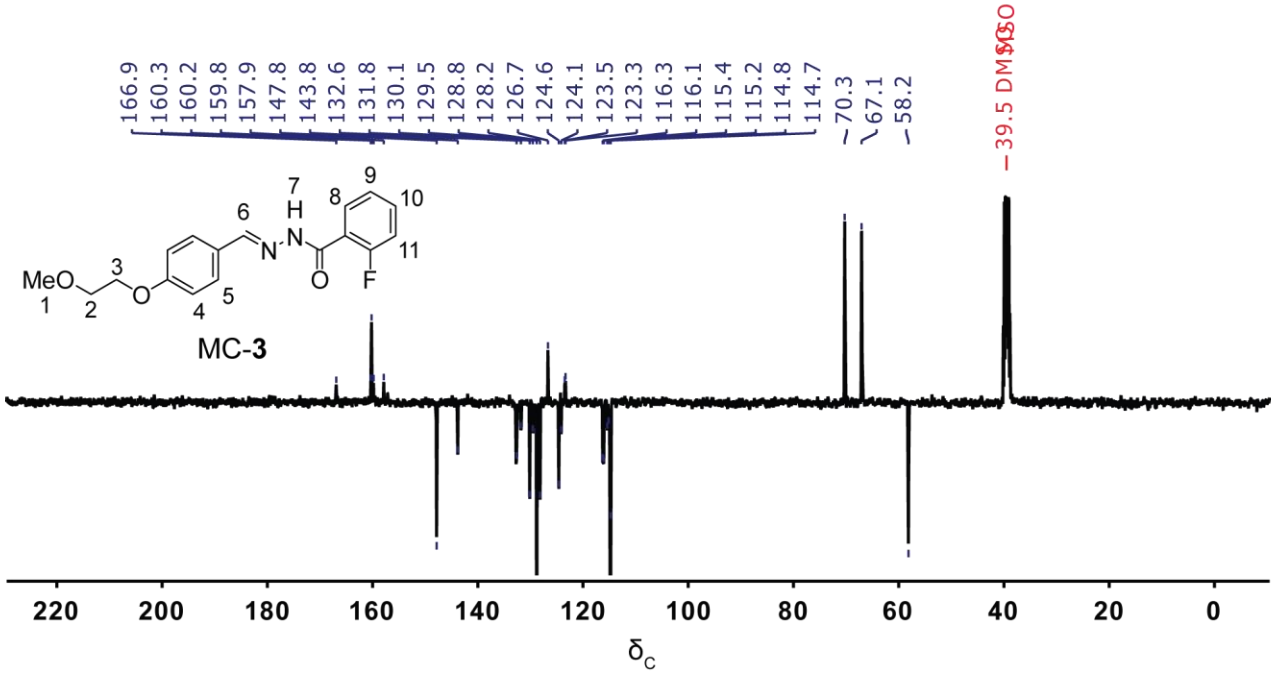
166.9
160.3
160.2
159.8
157.9
147.8
143.8
132.6
131.8
130.1
129.5
128.8
128.2
126.7
124.6
124.1
123.5
123.3
116.3
116.1
115.4
115.2
114.8
114.7
70.3
67.1
58.2
39.5 DMSO
MC-3
220 200 180 160 140 120 100 80 60 40 20 0
δC

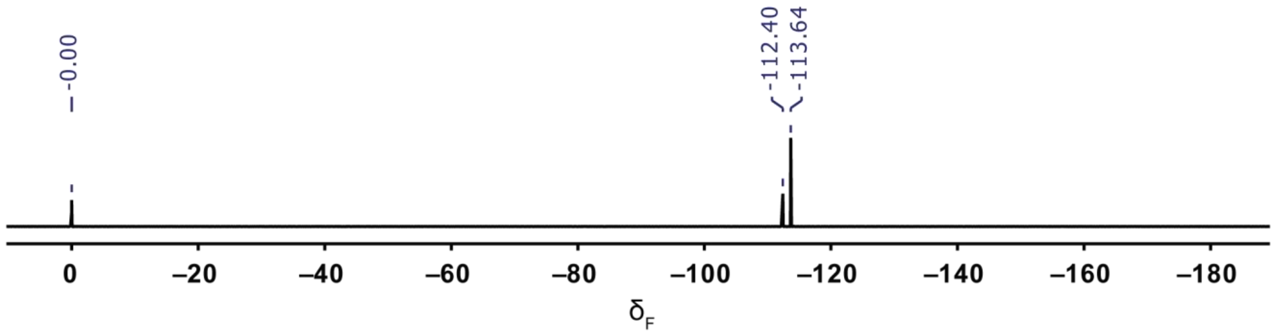
-0.00
-112.40
-113.64
0 −20 −40 −60 −80 −100 −120 −140 −160 −180
δF

11. References

1. (a) V. A. Bloomfield and R. K. Dewan, *J. Phys. Chem.*, 1971, **75**, 3113-3119; (b) W. Heller, *J. Phys. Chem.*, 1965, **69**, 1123-1129; (c) A. Jouyban, S. Soltanpour and H. K. Chan, *Int. J. Pharm.*, 2004, **269**, 353-360.
2. G. R. Fulmer, A. J. M. Miller, N. H. Sherden, H. E. Gottlieb, A. Nudelman, B. M. Stoltz, J. E. Bercaw and K. I. Goldberg, *Organometallics*, 2010, **29**, 2176-2179.
3. F. della Sala and E. R. Kay, *Angew. Chem. Int. Ed.*, 2015, **54**, 4187-4191.
4. J. D. Menczel and R. B. Prime, eds., *Thermal Analysis of Polymers. Fundamentals and applications.*, Wiley, Hoboken, New Jersey, 2009.
5. F. Rastrelli, S. Jha and F. Mancin, *J. Am. Chem. Soc.*, 2009, **131**, 14222-14224.
6. W. Edwards, N. Marro, G. Turner and E. R. Kay, *Chem. Sci.*, 2018, **9**, 125-133.
7. B. Yan, Z.-J. Zhu, O. R. Miranda, A. Chompoosor, V. M. Rotello and R. W. Vachet, *Anal. Bioanal. Chem.*, 2010, **396**, 1025-1035.
